# Evaluation of the accuracy and repeatability of Deepseek V3, Doubao, and Kimi1.5 in answering knowledge-related queries about chronic non-bacterial osteitis

**DOI:** 10.3389/frai.2025.1629149

**Published:** 2025-09-29

**Authors:** Zhenxing Zhu, Jun Xie, Longxin Zhou, Chaoran Yang, Feng Li

**Affiliations:** ^1^Department of Rehabilitation Medicine, Ganzhou People's Hospital, Ganzhou, China; ^2^Department of Obstetrics and Gynecology, Dayu County Maternal and Child Health Hospital, Ganzhou University, Ganzhou, China

**Keywords:** chronic non-bacterial osteitis, Chinese AI chatbots, knowledge retrieval, Deepseek V3, Doubao, Kimi1.5

## Abstract

**Background:**

There are significant differences in the diagnosis and treatment of chronic non-bacterial osteitis (CNO), and there is an urgent need for health education efforts to enhance awareness of this condition. Deepseek V3, Doubao, and Kimi1.5 are highly popular language models in China that can provide knowledge related to diseases. This article aims to investigate the accuracy and reproducibility of the responses provided by these three artificial intelligence (AI) language models in answering questions about CNO.

**Methods:**

According to the latest expert consensus, 16 questions related to CNO were collected. The three AI language models were separately asked these questions at three different times. The answers were independently evaluated by two orthopedic experts.

**Results:**

Among the responses of the three AI models to 16 CNO-related questions across three rounds of testing, only Doubao received “Completely incorrect” ratings (accounting for 6.25%) in the third round of scoring by Reviewer 2. During the answering process, Doubao had the shortest response time and provided the most words in its answers. In the first and third rounds of scoring by the first expert, Kimi scored the highest (3.938 ± 0.342, 3.875 ± 0.873), while in the second round, Doubao scored the highest (3.875 ± 0.5). In the second round of scoring by the second expert, Doubao received the highest score (3.812 ± 0.403). In the first and third rounds, Kimi1.5 received the highest score (3.812 ± 0.602, 3.812 ± 0.704).

**Conclusion:**

Deepseek V3, Doubao, and Kimi1.5 are capable of answering most questions related to CNO with good accuracy and reproducibility, showing no significant differences.

## 1 Introduction

Chronic non-bacterial osteitis (CNO) is an autoinflammatory bone disease that commonly affects children and adolescents (Zhao et al., 2021). CNO can lead to severe complications, including bone pain and bone damage. Its pathophysiological characteristics are marked by increased inflammasome assembly and imbalanced cytokine expression. Treatment medications include nonsteroidal anti-inflammatory drugs (NSAIDs), corticosteroids, etc. (Hedrich et al., 2023). Though reliable epidemiological data are lacking, CNO is likely to be one of the most common autoinflammatory syndromes (Schnabel et al., 2016). In Germany, incidence rates were estimated at 0.4 per 100.000 children/year (Jansson et al., 2011). However, despite recent advances in our understanding of CNO, the number of detected cases has increased in recent years (Lenert and Ferguson, 2020). Nevertheless, there are considerable practice variations in the marking, diagnosis, and treatment of CNO currently (Winter et al., 2025). Therefore, there is an urgent need for health education efforts to enhance awareness of CNO.

Artificial intelligence (AI) is a system’s capacity to analyze data. It uses computers and machines to boost humans’ decision - making, problem - solving, and tech innovation capabilities (Tang et al., 2022). Currently, AI is becoming increasingly popular in China and even worldwide, and it is being applied in various fields (Wei et al., 2022). With the aging of the population, the global demand for high-quality healthcare has increased, while artificial intelligence (AI) has been widely applied in modern medicine—for instance, in diagnosis and treatment (Higuchi et al., 2024; Chadebecq et al., 2023). These AI tools can assist clinicians in different fields to make more informed decisions (Kulkarni and Singh, 2023). In the field of oncology, AI can be used for cancer screening and diagnosis (Abbasi, 2020). In cardiovascular medicine, it can integrate various forms of patient data to aid doctors in treatment (Lüscher et al., 2024). Even in surgical procedures, AI can improve multiple aspects such as preoperative, intraoperative, and postoperative care (Varghese et al., 2024). The rapid development of AI has provided convenience for the entire medical industry. In daily life, it offers both patients and doctors channels to access disease-related knowledge. However, people are easily misled due to inaccurate and outdated information.

In clinical practice, it is common to encounter patients using these artificial intelligence language models to inquire about disease-related knowledge. Previous studies have evaluated the accuracy and reproducibility of ChatGPT in answering questions related to *Helicobacter pylori* (Lai et al., 2024). The results showed that ChatGPT could provide correct answers to most queries related to *Helicobacter pylori*, demonstrating good accuracy and reproducibility.

Deepseek V3, Doubao, and Kimi1.5 are the three most popular and freely accessible artificial intelligence language models in China. They have been widely applied in the country and are used by a large number of people, so it is necessary to assess the accuracy and reproducibility of these three language models in answering disease-related questions.

## 2 Methods

### 2.1 Data source

In order to evaluate the accuracy of Deepseek V3 (https://chat.deepseek.com), Doubao (https://www.doubao.com), and Kimi1.5 (https://kimi.moonshot.cn) in answering questions related to CNO, we selected highly scored statements from the latest expert consensus on CNO diseases. This guideline was jointly released in 2025 by over 40 medical experts and served as the source for the questionnaire in this article (Winter et al., 2025). To ensure that the selection process not only guarantees the clinical representativeness of the questions (aligning with patients’ actual needs) but also highlights professional guidance (addressing complex diagnosis and treatment challenges), a core question list that combines practicality and professionalism was finally developed.

We have proposed a total of 16 questions, covering the definition (Question 1), clinical manifestations (Questions 3 and 4), diagnosis (Questions 2, 5, and 6–8), differential diagnosis (Questions 9–11), treatment (Questions 13–16), and treatment of the disease. These questions include those frequently asked by patients, such as *“What are the most common manifestations of adult chronic nonbacterial osteomyelitis?,”* as well as highly complex ones, such as *“What is the first-line treatment option for adult chronic nonbacterial osteomyelitis?.”* Furthermore, only questions based on highly recommended or consensus-reached outcomes were selected; those involving controversial recommendations were excluded.

**Figure 1:**
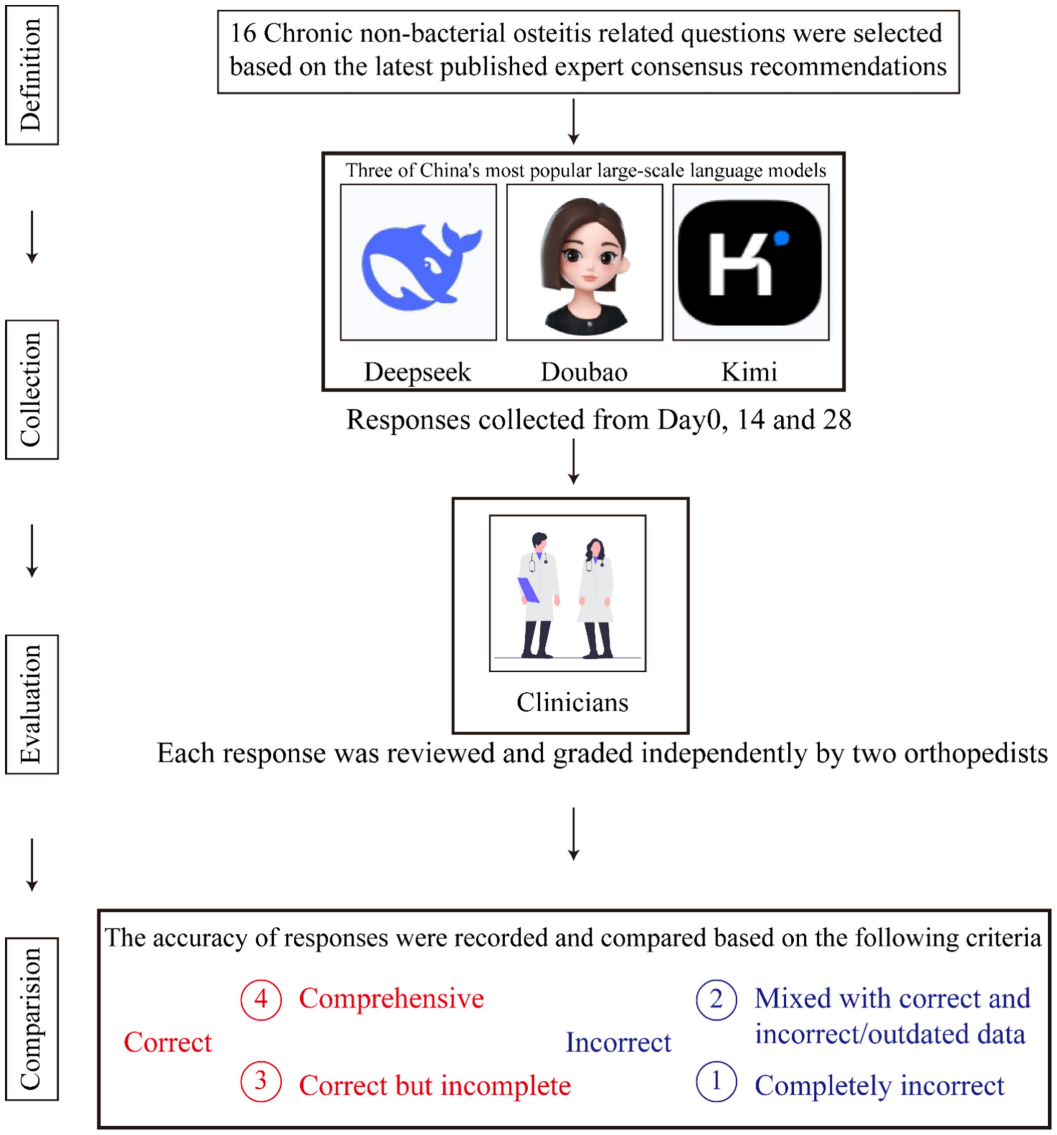
The flow chart of this study.

The language of the questions affects both model performance and the applicability of results to other linguistic or regional contexts (Nezhad et al., 2024). Therefore, all questions in this study were posed in Chinese. Figure 1 provides a comprehensive overview of the screening process.

### 2.2 Response generation

Deepseek V3, Doubao, and Kimi1.5 are the three most popular AI language large models in China, all of which can be used for free. In order to evaluate the usefulness of these AI models in answering questions related to CNO, each question was independently inputted, and each model was allowed to provide only one response. We directly input the organized questions for inquiry without using any additional prompts. Nor did we standardize the AI “system messages” or context windows.

The response time taken to answer the question and the number of characters in the answer were recorded. To assess their reliability in responding to CNO-related queries, independent tests were conducted at three different time points within a 28-day period. All data collection time is April 2025. The counting object of this article is Chinese characters, and the counting tool is Microsoft Word document.

### 2.3 Assessment and grading

Each question was independently reviewed and scored by two orthopedic surgeons, and the reviewers were unaware of the source of the answer corresponding to each question. For questions where there were discrepancies in scoring, they were referred back to a more experienced expert, who was asked to judge which reviewer’s scoring for these questions was more reasonable. To assess accuracy, each question was evaluated individually based on statements recommended in the latest expert consensus, using the following scoring system (Hu et al., 2024; Lai et al., 2024): (1) Comprehensive (4 points); (2) correct but incomplete (3 points); (3) mixed with correct and incorrect/outdated data (2 points); and (4) completely incorrect (1 point).

### 2.4 Term definitions

1. Accuracy: Evaluated based on the scores of the three AI models across three rounds of testing for the 16 CNO-related questions.
2. Reproducibility: Evaluated based on the score variations of the three AI models across three rounds of testing when answering the 16 CNO-related questions.
3. Comprehensiveness: Evaluated based on the degree of consistency between the responses of the three AI models to the 16 CNO-related questions and the recommendations in the expert consensus.

### 2.5 Statistical analysis

The evaluators’ assessments of the AI’s responses related to CNO were expressed as median and interquartile range (IQR), and non-parametric tests were used to evaluate the differences between groups. Bonferroni correction was applied to the comparison of the three groups.*p* < 0.05 was considered statistically significant. All analyses were conducted statistically using R (version 4.4.3) and visualized through GraphPad Prism (version 9.3.0) and OriginPro 2024. Cohen’s kappa was used to assess the level of inter-rater agreement between the two reviewers, with calculations performed using IBM SPSS Statistics 27. The results showed that the Kappa value was 0.336, and *p* < 0.01.

## 3 Results

### 3.1 The basic characteristics of AI’S responses to CNO-related questions

**Figure 2:**
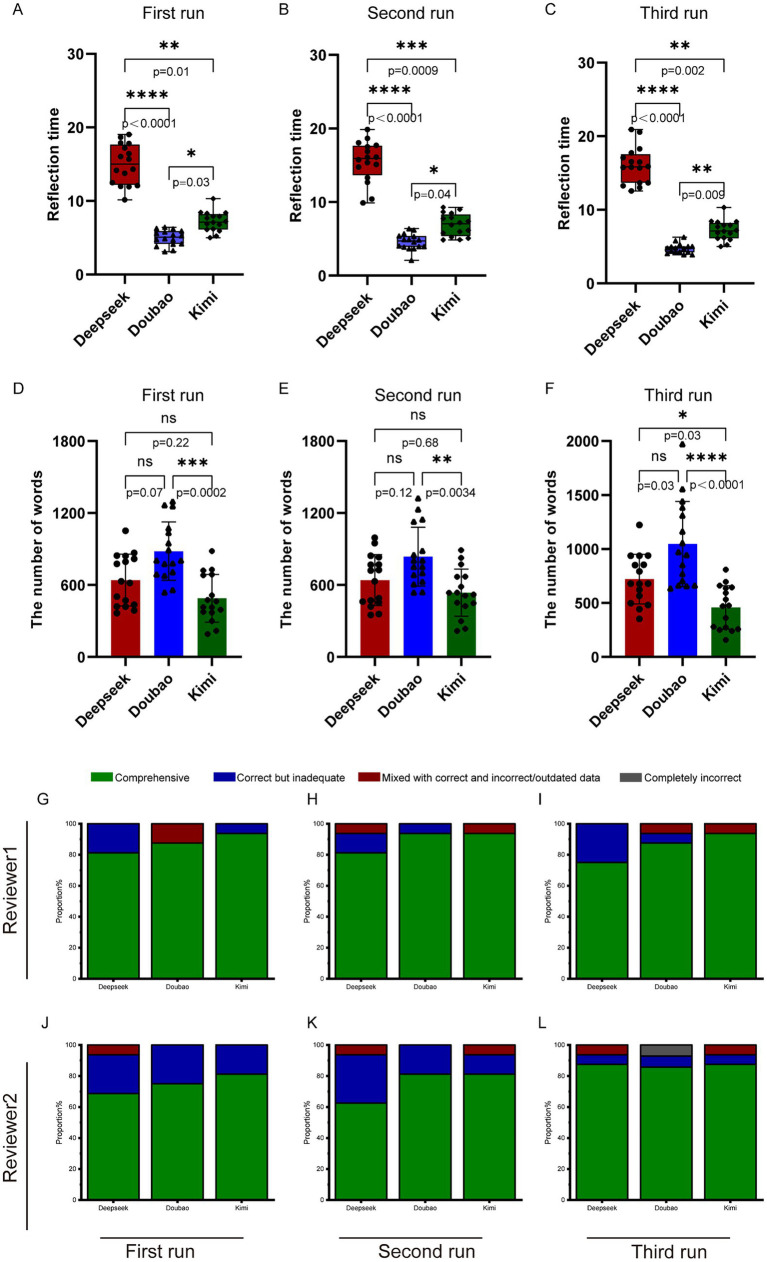
The basic characteristics of AI’s responses to CNO-related questions. **(A–C)** Three sets of running results regarding the response time of different types of artificial intelligence to CNO-related questions. **(D–F)** Three sets of running results regarding the word counts of different types of artificial intelligence to CNO-related questions. **(G–I)** The results of three trials conducted by Reviewer 1 in inquiring about CNO-related questions to these three AI models. **(J–L)** The results of three trials conducted by Reviewer 2 in inquiring about CNO-related questions to these three AI models. Statistical analysis was performed using non-parametric tests. ns, nonsignificant; *p < 0.05; **p < 0.01; ***p < 0.001; ****p < 0.0001.

Based on the latest expert consensus, we proposed 16 questions related to CNO, covering basic knowledge, diagnosis, differential diagnosis, examination, and treatment. As can be seen from Figures 2A–C and Table 1, Doubao took the shortest time to answer questions in all three rounds of Q&A, with response times of 5.09 (4.18, 5.84)s, 4.73 (3.94, 5.36)s, and 4.78 (4.26, 5.01)s, respectively. Kimi came next, with response times of 7.28 (5.99, 8.19)s, 7.05 (5.65, 8.04)s, and 7.15 (6.17, 8.02)s for the three rounds. Deepseek V3 took the longest time to answer these questions, with response times of 15.01 (12.39, 17.36)s, 15.93 (14.40, 17.57)s, and 15.84 (13.67, 17.24)s, respectively. From Figures 2D,E and Table 2, it can be seen that in the three rounds of answers to CNO-related questions, Doubao provided the longest responses, with word counts of 804.5 (703.2, 1044.5), 778.5 (670.8,946.0), and 958.5 (694.5, 1329.2), respectively. Deepseek V3 ranked second, with word counts of 624.0 (432.0, 824.0), 675.5 (459.0, 785.2), and 679.0 (548.2, 874.8). Kimi1.5 had the shortest responses, with word counts of 439.0 (374.2, 683.5), 526.5 (441.8, 668.2), and 473.0 (268.5, 638.0).

**Table 1:** Comparison of the response times of three AIs to CNO-related questions.

Response times	Deepseek V3	Doubao	Kimi 1.5
First run, median (IQR)	15.01 (12.39,17.36)	5.09 (4.18,5.84)	7.28 (5.99,8.19)
Second run, median (IQR)	15.93 (14.40,17.57)	4.73 (3.94,5.36)	7.05 (5.65,8.04)
Third run, median (IQR)	15.84 (13.67,17.24)	4.78 (4.26,5.01)	7.15 (6.17,8.02)

**Table 2:** Comparison of the word counts of the answers given by three AIs to CNO-related questions.

Word counts	Deepseek V3	Doubao	Kimi 1.5
First run, median (IQR)	624.0 (432.0,824.0)	804.5 (703.2,1044.5)	439.0 (374.2,683.5)
Second run, Median (IQR)	675.5 (459.0,785.2)	778.5 (670.8,946.0)	526.5 (441.8,668.2)
Third run, median (IQR)	679.0 (548.2,874.8)	958.5 (694.5,1329.2)	473.0 (268.5,638.0)

In the first round of scoring by Reviewer 1 (Figure 2G): For Deepseek V3’s scores, Correct but Inadequate and Comprehensive account for 18.75 and 81.25% respectively; For Doubao’s scores, Mixed with Correct and Incorrect/Outdated Data and Comprehensive account for 12.5 and 87.5% respectively; For Kimi1.5’s scores, Correct but Inadequate and Comprehensive account for 6.25 and 93.75%, respectively. In the second round of scoring by Reviewer 1 (Figure 2H): For Deepseek V3’s scores, Mixed with Correct and Incorrect/Outdated Data, Correct but Inadequate, and Comprehensive account for 6.25, 12.5, and 81.25% respectively; For Doubao’s scores, Correct but Inadequate and Comprehensive account for 6.25 and 93.75% respectively; For Kimi1.5’s scores, Mixed with Correct and Incorrect/Outdated Data and Comprehensive account for 6.25 and 93.75%, respectively. In the third round of scoring by Reviewer 1 (Figure 2I): For Deepseek V3’s scores, Correct but Inadequate and Comprehensive account for 25 and 75% respectively; For Doubao’s scores, Mixed with Correct and Incorrect/Outdated Data, Correct but Inadequate, and Comprehensive account for 6.25, 6.25, and 87.5% respectively; For Kimi1.5’s scores, Mixed with Correct and Incorrect/Outdated Data and Comprehensive account for 6.25 and 93.75%, respectively.

In the first round of scoring by Reviewer 2: For Deepseek V3’s scores, Mixed with Correct and Incorrect/Outdated Data, Correct but Inadequate, and Comprehensive account for 6.25, 25, and 68.75% respectively; For Doubao’s scores, Correct but Inadequate and Comprehensive account for 25 and 75% respectively; For Kimi1.5’s scores, Correct but Inadequate and Comprehensive account for 18.75 and 81.25%, respectively. In the second round of scoring by Reviewer 2: For Deepseek V3’s scores, Mixed with Correct and Incorrect/Outdated Data, Correct but Inadequate, and Comprehensive account for 6.25, 31.25, and 62.5% respectively; For Doubao’s scores, Correct but Inadequate and Comprehensive account for 18.75 and 81.25% respectively; For Kimi1.5’s scores, Mixed with Correct and Incorrect/Outdated Data, Correct but Inadequate, and Comprehensive account for 6.25, 12.5, and 81.25%, respectively. In the third round of scoring by Reviewer 2: For Deepseek V3’s scores, Mixed with Correct and Incorrect/Outdated Data, Correct but Inadequate, and Comprehensive account for 6.25, 6.25, and 87.5% respectively; For Doubao’s scores, Completely Incorrect, Correct but Inadequate, and Comprehensive account for 6.25, 6.25, and 75% respectively; For Kimi1.5’s scores, Mixed with Correct and Incorrect/Outdated Data, Correct but Inadequate, and Comprehensive account for 6.25, 6.25, and 87.5%, respectively.

### 3.2 Comparison of ratings between two reviewers

**Figure 3:**
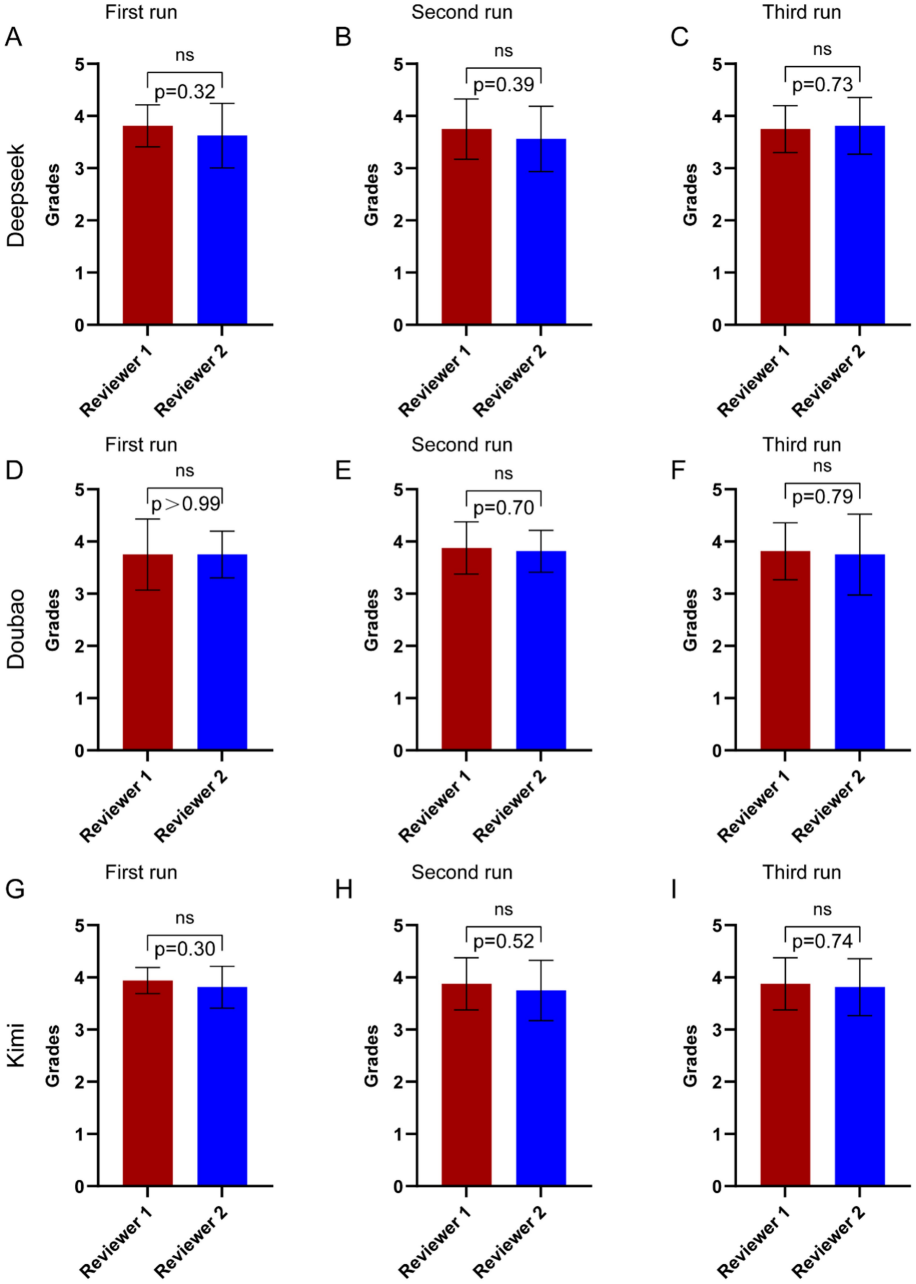
Comparison of ratings between two reviewers by the three AI language models to questions related to CNO. **(A–C)** The results of three trials conducted by two reviewers in asking CNO-related questions to Deepseek V3. (D-F) The results of three trials conducted by two reviewers in asking CNO-related questions to Doubao. **(G–I)** The results of three trials conducted by two reviewers in asking CNO-related questions to Kimi 1.5. Statistical analysis was performed using *t* test. ns, not significant.

The questions answered by the three AI models were evaluated by two orthopedic doctors based on the expert consensus. When there were disagreements between the two doctors, a more experienced expert conducted a re-evaluation. As can be seen in Figure 3A–C, in the first two rounds of Deepseek V3’s operation, Reviewer 1 gave slightly higher scores than Reviewer 2, while in the third round, Reviewer 2 gave higher scores. However, there were no significant differences in the scores across the three rounds. From Figure 3D–F, it can be seen that Reviewer 1 and Reviewer 2 gave similar scores to Doubao’s answers, with no significant differences. In Figure 3G–I, it can be observed that in the first round, Reviewer 1 gave a higher score than Reviewer 2, while in the subsequent two rounds, the scores were similar, with no significant differences across the three rounds.

### 3.3 The score distribution of Deepseek V3, Doubao, and Kimi1.5 in answering CNO-related questions

**Figure 4:**
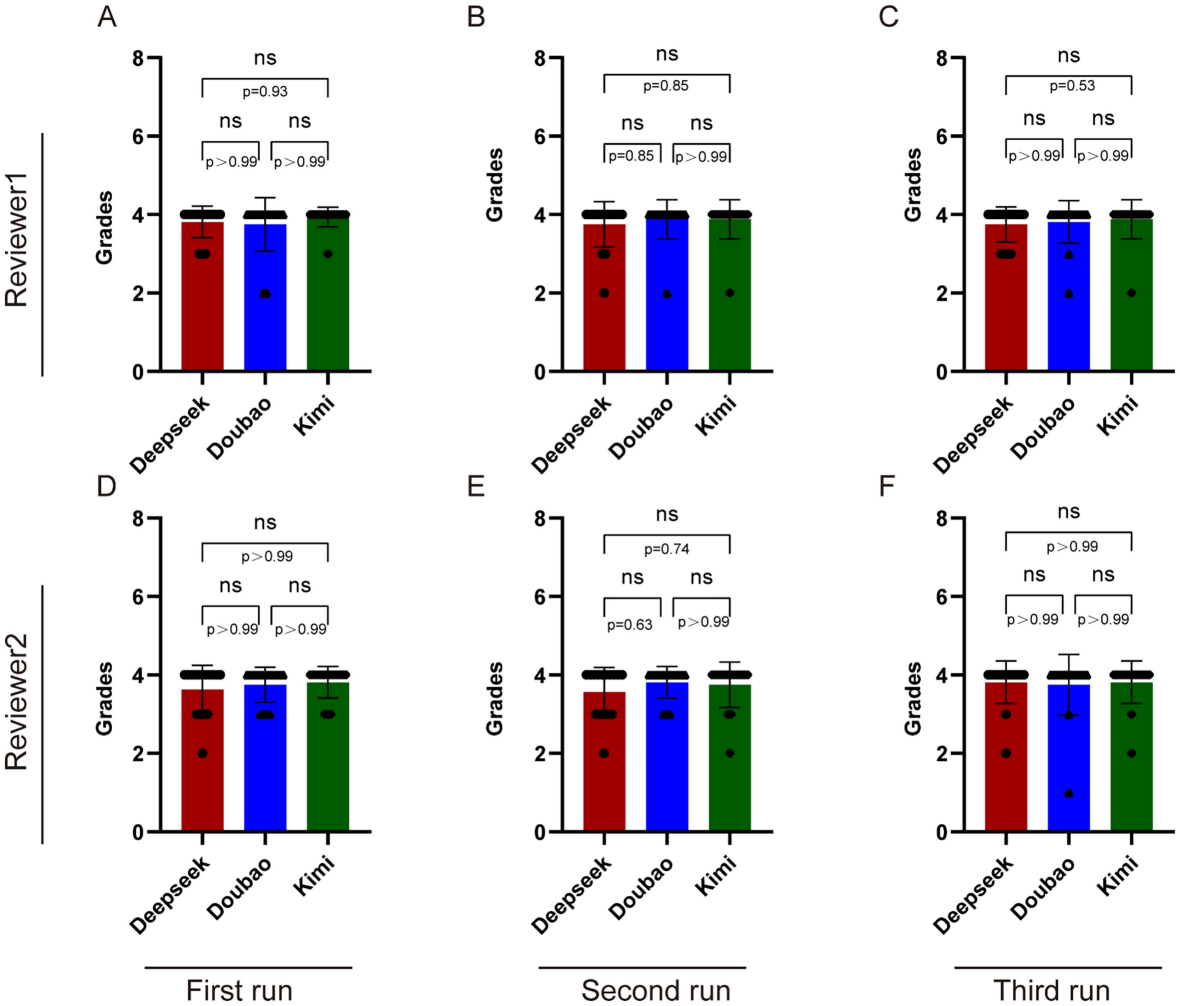
Comparison among Deepseek V3, Doubao, and Kimi 1.5 in answering CNO-related questions. **(A–C)** The results of three trials conducted by Reviewer 1 in inquiring about CNO-related questions to these three AI models. **(D–F)** The results of three trials conducted by Reviewer 2 in inquiring about CNO-related questions to these three AI models. Statistical analysis was performed using Kruskal-Wallis. ns, nonsignificant.

Supplementary Tables S1–S3 present the scores given by the two reviewers. From Supplementary Table S4 and Figure 4, it can be seen that Reviewer 1’s score distributions for Deepseek V3, Doubao, and Kimi1.5 in the first and second rounds were all 4.00 (4.00, 4.00) (Figures 4A,B). In the third round of scoring, the scores for Deepseek V3, Doubao, and Kimi1.5 were 4.00 (3.75, 4.00), 4.00 (4.00, 4.00), and 4.00 (4.00, 4.00), respectively (Figure 4C).

For Reviewer 2, the score distributions for Deepseek V3, Doubao, and Kimi1.5 in the first round were 4.00 (3.00, 4.00), 4.00 (3.75, 4.00), and 4.00 (4.00, 4.00), respectively (Figure 4D). In the second round, their score distributions were 4.00 (3.00, 4.00), 4.00 (4.00, 4.00), and 4.00 (4.00, 4.00), respectively (Figure 4E). In the third round, all their score distributions were 4.00 (4.00, 4.00) (Figure 4F).

From Table 3, it can be observed that in the first round of scoring by Reviewer 1, Kimi1.5 received the highest average score of 3.938 ± 0.342, followed by Deepseek V3 (3.812 ± 0.403), with Doubao (3.75 ± 0.683) receiving the lowest score. In the second round of scoring, Doubao, Kimi1.5, and Deepseek V3 achieved scores of 3.875 ± 0.5, 3.875 ± 0.683, and 3.75 ± 0.557, respectively. In the third round of scoring, Doubao, Kimi1.5, and Deepseek V3 obtained scores of 3.812 ± 0.544, 3.875 ± 0.873, and 3.75 ± 0.447, respectively. It can be seen that in the first rounds of scoring by Reviewer 2, Kimi1.5 received the highest scores (3.812 ± 0.602), but in the second round, Doubao received the highest scores (3.812 ± 0.403). In the third round of scoring, Kimi1.5 received the highest score of 3.812 ± 0.704.

**Table 3:** The results of three trials conducted by two reviewers in asking CNO-related questions to the three AI language models.

Reviewers’ grades		DeepseekV3	Doubao	Kimi 1.5
Reviewer 1 grades	First run, mean±SD	3.812 ± 0.403	3.75 ± 0.683	3.938 ± 0.342
	Second run, mean±SD	3.75 ± 0.557	3.875 ± 0.5	3.875 ± 0.683
	Third run, mean±SD	3.75 ± 0.447	3.812 ± 0.544	3.875 ± 0.873
Reviewer 2 grades	First run, mean±SD	3.625 ± 0.619	3.75 ± 0.447	3.812 ± 0.602
	Second run, mean±SD	3.562 ± 0.629	3.812 ± 0.403	3.75 ± 0.602
	Third run, mean±SD	3.812 ± 0.544	3.75 ± 0.775	3.812 ± 0.704

## 4 Discussion

In the field of patient care, AI can serve as a “highly efficient auxiliary tool” for clinicians: On one hand, when doctors in primary hospitals treat patients with suspected CNO, AI can quickly generate an initial diagnosis differential checklist and examination recommendations based on the patients’ symptoms (Apornvirat et al., 2024). This helps shorten the time for diagnostic decision-making and reduce missed diagnoses. On the other hand, when patients report side effects during follow-ups, AI can immediately provide “directions for preliminary treatment plan adjustments” to assist doctors in responding quickly (Hartman-Kenzler et al., 2024). This avoids risks caused by long intervals between follow-up visits and indirectly improves the efficiency and safety of patient care. In the field of patient education, AI can provide “personalized and accessible health guidance”: Traditional patient education mostly relies on general manuals, which are difficult to adapt to individual conditions (Apornvirat et al., 2024). In contrast, AI can integrate a patient’s medical history (e.g., a history of diabetes) (Davis et al., 2023) and treatment plan (e.g., hormone medication) (Natchagande et al., 2024), while also issuing warnings about “signals requiring emergency medical attention” (e.g., sudden vision loss, worsening eye pain). This helps patients understand the logic of diagnosis and treatment, improve treatment adherence, and reduce treatment interruptions or neglect of risks caused by cognitive biases.

In summary, in this study, among Deepseek V3, Doubao, and Kimi1.5, Deepseek V3 took the longest time to think when answering questions, while Doubao responded the fastest. Based on the recommendations outlined in clinical expert guidelines, both reviewers confirmed that an answer to each question could be found in the expert consensus. Since each expert conducted independent evaluations, the majority of the scores were consistent. However, individual cognitive characteristics of experts—such as personal clinical experience and attention allocation—may also lead to discrepancies in scoring. This is an inherent attribute of human subjective judgment and constitutes a bias that is controllable but cannot be completely eliminated. Additionally, among all the answers, Doubao provided the most words in its responses, whereas Kimi1.5 provided the fewest. There were no significant differences in the scores given by the two reviewers for the answers provided by these three AIs, and the scores all fell between 3 and 4, indicating the accuracy of the AI’s responses is highly satisfactory. More importantly, the high scores were maintained across all three rounds, demonstrating good repeatability in the AI’s responses. However, for the fourth question (“Which parts of the body are most frequently affected by chronic non-bacterial osteitis in adults?”), none of the three AIs received a score of 4 and Doubao provided an incorrect answer to the fourth question in the third round. Regarding this issue, the latest guidelines suggest that in adults, CNO predominantly manifests in the anterior chest wall, including the clavicle, upper ribs, and sternum, which are the most commonly affected sites (Winter et al., 2025).

Currently, there have been numerous studies on the application of ChatGPT in medicine, as well as comparative analyses of its research performance in the medical field against different AI models (Bradshaw, 2023). Ozan Yazıcı et al. compared ChatGPT and Perplexity in terms of treatment response and reliability assessment for rectal cancer, finding that Perplexity had higher accuracy than ChatGPT (Yazici et al., 2023). Zhou et al. (2025) evaluated the performance of ChatGPT and DeepSeek in generating educational materials for patients undergoing spinal surgery, discovering that DeepSeek-R1 produced the most credible answers, although the AI models generally received moderate DISCERN scores. Even within the same AI model, differences can exist between versions. Hu et al. examined two versions of ChatGPT in relation to questions about *Helicobacter pylori*, finding that both versions had high accuracy, with ChatGPT 3.5 performing well in the areas of indications, treatment, and gut microbiota, and ChatGPT 4 excelling in diagnosis, gastric cancer, and prevention (Hu et al., 2024). Additionally, researchers have found that the fusion of emotion-aware embeddings in large language models (including Flan-T5, Llama 2, DeepSeek-R1, and ChatGPT 4) is applied to intelligent response generation (Rasool et al., 2025). In this study, when answering relevant questions, we can observe that each AI model has its own characteristics. Deepseek V3 reminds users after each answer that the response is for reference only. Doubao expands on other related questions after answering the initial query. Kimi1.5 not only expands on related questions but also displays the answer sources on the right-hand side.

Although these three AIs scored relatively high, they all have limitations. (1) their sources are not based on clinical evidence but rather on internet sources, many of which are not professional literature guidelines. (2) When new clinical guidelines are updated, AIs do not provide answers according to the latest clinical guidelines or expert consensus, which can easily lead to outdated information and incorrect answers. Furthermore, when AI models are updated, their accuracy also changes. In future analyses, we will also include re-testing after major model updates. (3) The conclusions of this study are only applicable to Chinese Q&A scenarios. Due to the variations in treatment guidelines across different specialties and regions worldwide, giving priority to the guidelines of those regions poses a significant challenge for AI. (4) This study only based its inquiries on 16 questions. Though these questions cover such aspects as disease definition, diagnosis, and treatment, the number is relatively limited. Moreover, the grading system is based on a 4-point scale. Though it has been used in relevant literature, the non-refined scale has the drawback of missing minor errors. (5) Semantic similarity-based evaluation indicators such as BERTScore have not been applied in this study, yet these indicators are an important factor for conducting objective comparisons between models and driving model performance improvement.

Furthermore, patient inquiries in the real world may differ from expert-developed questions in terms of complexity and wording. In future studies, the number of questions should be further increased, and the wording used by patients when they ask questions via AI should be collected. Nowadays, the use frequency of AI in clinical work is increasing. Future research can shift from static Q&A to dynamic clinical reasoning by designing case-based scenarios—requiring models to interpret symptoms, order tests, and propose treatment plans, thereby moving from factual retrieval to diagnostic reasoning (Rasool et al., 2020). Therefore, we suggest that in the future, AI-generated medical answers should undergo appropriate certification and regular review. This approach can provide patients with more accurate disease information, enhance their understanding of the disease, and improve their management of the condition.

## 5 Conclusion

Overall, through this study, we found that these three AI models demonstrate relatively high accuracy and reproducibility when answering CNO-related static questions based on their training data. With no significant differences observed. We believe that with the continuous development of AI prediction models, Deepseek V3, Doubao, and Kimi1.5 have the potential to serve as supplementary tools in addition to expert consensus and guidelines.

## Data Availability

The original contributions presented in the study are included in the article/Supplementary material, further inquiries can be directed to the corresponding author/s.
