# Supplementary material for: Evaluation of the accuracy and repeatability of Deepseek V3, Doubao, and Kimi1.5 in answering knowledge-related queries about chronic non-bacterial osteitis
Source: Front Artif Intell. 2025 Sep 29;8:1629149. doi: 10.3389/frai.2025.1629149 (PMC12515971; doi:10.3389/frai.2025.1629149)
Supplement: Supplementary file 1 [file Data_Sheet_1.zip › day1/day1-Deepseek.docx]

1成人慢性非细菌性骨炎的定义是什么？


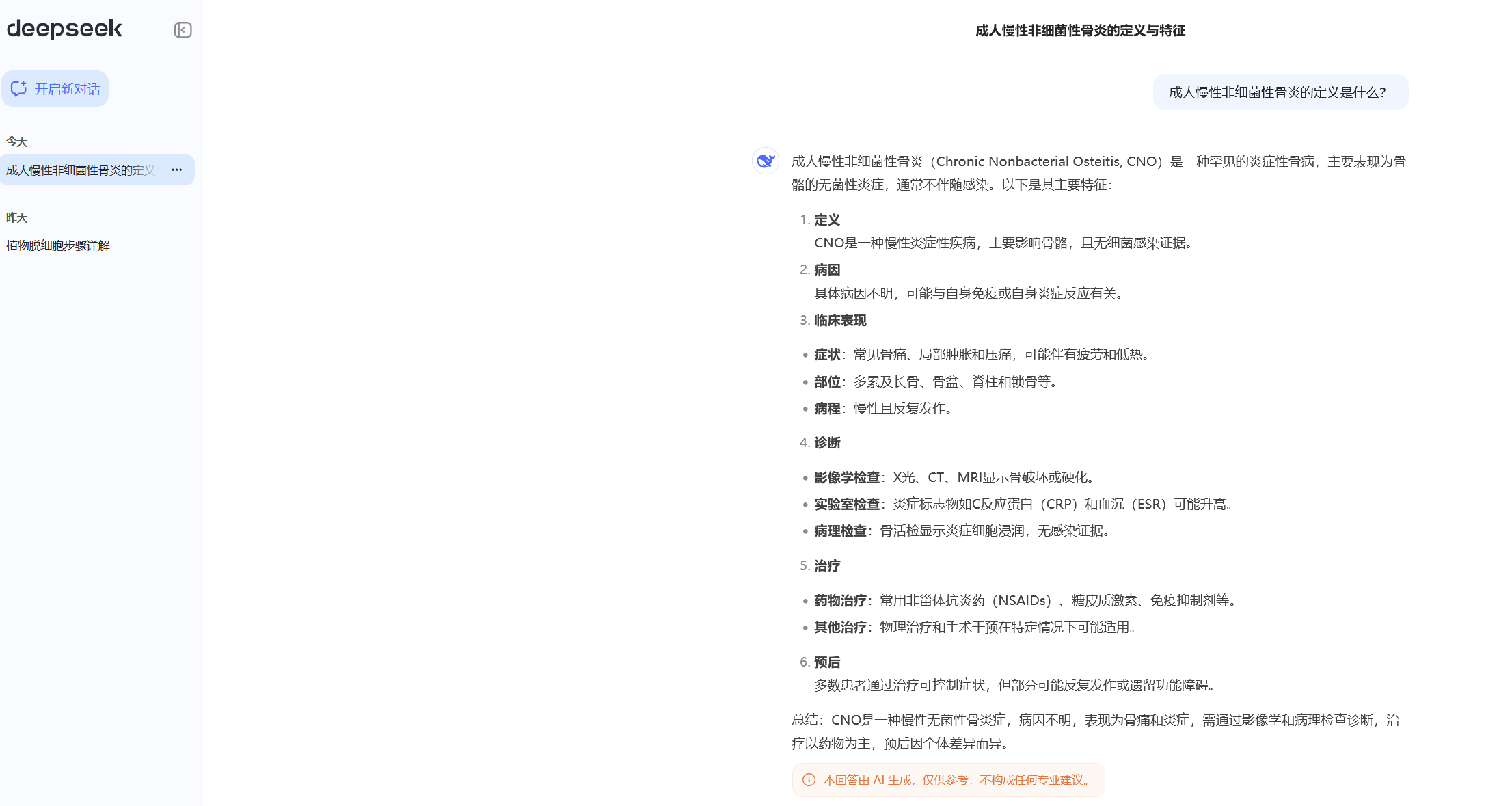


成人慢性非细菌性骨炎（Chronic Nonbacterial Osteitis, CNO）是一种罕见的炎症性骨病，主要表现为骨骼的无菌性炎症，通常不伴随感染。以下是其主要特征：

1. **定义**

CNO是一种慢性炎症性疾病，主要影响骨骼，且无细菌感染证据。

2. **病因**

具体病因不明，可能与自身免疫或自身炎症反应有关。

3. **临床表现**

- **症状**：常见骨痛、局部肿胀和压痛，可能伴有疲劳和低热。

- **部位**：多累及长骨、骨盆、脊柱和锁骨等。

- **病程**：慢性且反复发作。

4. **诊断**

- **影像学检查**：X光、CT、MRI显示骨破坏或硬化。

- **实验室检查**：炎症标志物如C反应蛋白（CRP）和血沉（ESR）可能升高。

- **病理检查**：骨活检显示炎症细胞浸润，无感染证据。

5. **治疗**

- **药物治疗**：常用非甾体抗炎药（NSAIDs）、糖皮质激素、免疫抑制剂等。

- **其他治疗**：物理治疗和手术干预在特定情况下可能适用。

6. **预后**

多数患者通过治疗可控制症状，但部分可能反复发作或遗留功能障碍。

总结：CNO是一种慢性无菌性骨炎症，病因不明，表现为骨痛和炎症，需通过影像学和病理检查诊断，治疗以药物为主，预后因个体差异而异。

2成人慢性非细菌性骨炎的典型影像学特征是什么？


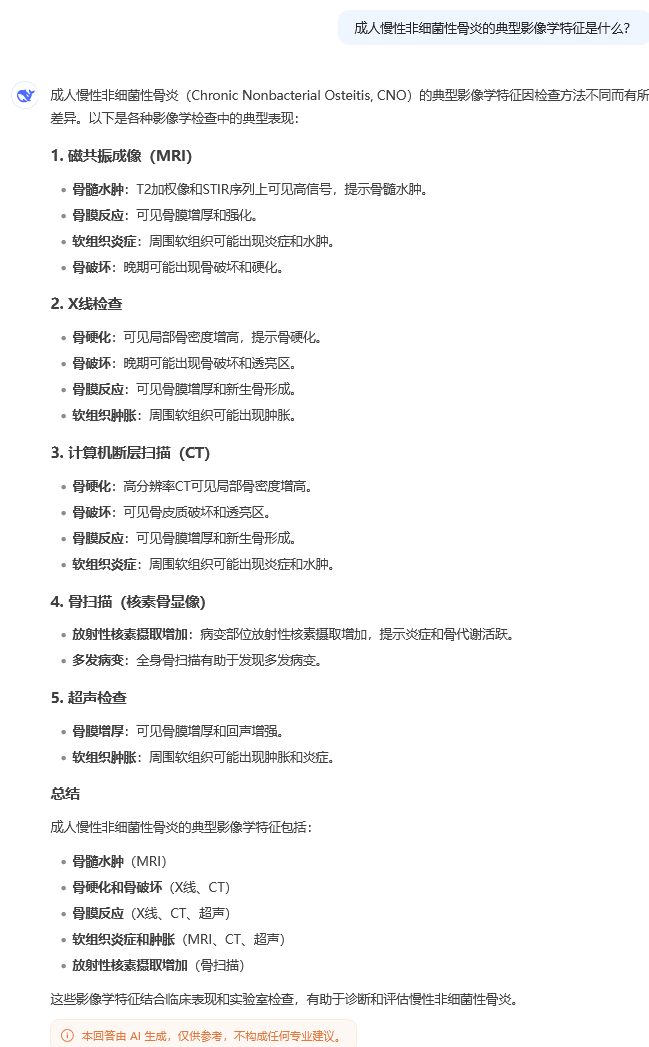


成人慢性非细菌性骨炎（Chronic Nonbacterial Osteitis, CNO）的典型影像学特征因检查方法不同而有所差异。以下是各种影像学检查中的典型表现：

### 1. **磁共振成像（MRI）**

- **骨髓水肿**：T2加权像和STIR序列上可见高信号，提示骨髓水肿。

- **骨膜反应**：可见骨膜增厚和强化。

- **软组织炎症**：周围软组织可能出现炎症和水肿。

- **骨破坏**：晚期可能出现骨破坏和硬化。

### 2. **X线检查**

- **骨硬化**：可见局部骨密度增高，提示骨硬化。

- **骨破坏**：晚期可能出现骨破坏和透亮区。

- **骨膜反应**：可见骨膜增厚和新生骨形成。

- **软组织肿胀**：周围软组织可能出现肿胀。

### 3. **计算机断层扫描（CT）**

- **骨硬化**：高分辨率CT可见局部骨密度增高。

- **骨破坏**：可见骨皮质破坏和透亮区。

- **骨膜反应**：可见骨膜增厚和新生骨形成。

- **软组织炎症**：周围软组织可能出现炎症和水肿。

### 4. **骨扫描（核素骨显像）**

- **放射性核素摄取增加**：病变部位放射性核素摄取增加，提示炎症和骨代谢活跃。

- **多发病变**：全身骨扫描有助于发现多发病变。

### 5. **超声检查**

- **骨膜增厚**：可见骨膜增厚和回声增强。

- **软组织肿胀**：周围软组织可能出现肿胀和炎症。

### 总结

成人慢性非细菌性骨炎的典型影像学特征包括：

- **骨髓水肿**（MRI）

- **骨硬化和骨破坏**（X线、CT）

- **骨膜反应**（X线、CT、超声）

- **软组织炎症和肿胀**（MRI、CT、超声）

- **放射性核素摄取增加**（骨扫描）

这些影像学特征结合临床表现和实验室检查，有助于诊断和评估慢性非细菌性骨炎。

3成人慢性非细菌性骨炎最常见的表现是什么？


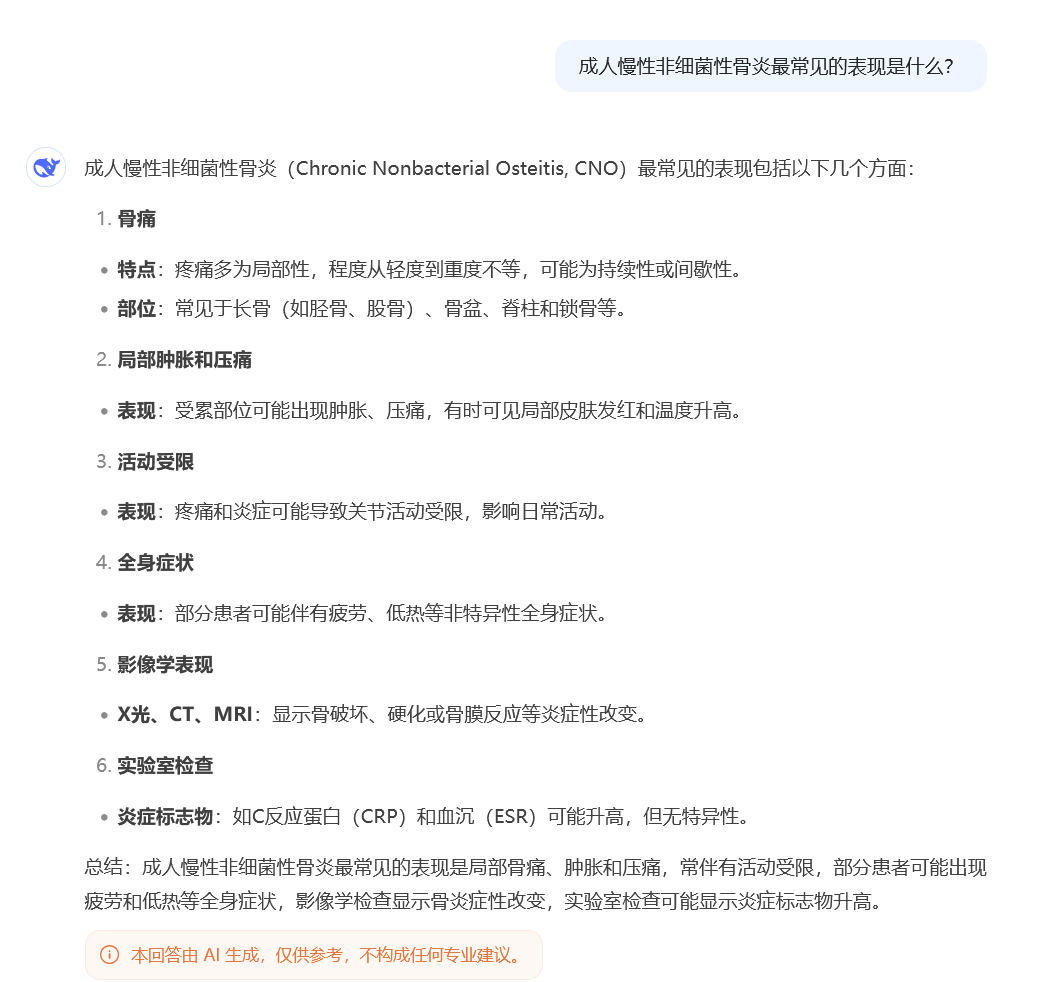


成人慢性非细菌性骨炎（Chronic Nonbacterial Osteitis, CNO）最常见的表现包括以下几个方面：

1. **骨痛**

- **特点**：疼痛多为局部性，程度从轻度到重度不等，可能为持续性或间歇性。

- **部位**：常见于长骨（如胫骨、股骨）、骨盆、脊柱和锁骨等。

2. **局部肿胀和压痛**

- **表现**：受累部位可能出现肿胀、压痛，有时可见局部皮肤发红和温度升高。

3. **活动受限**

- **表现**：疼痛和炎症可能导致关节活动受限，影响日常活动。

4. **全身症状**

- **表现**：部分患者可能伴有疲劳、低热等非特异性全身症状。

5. **影像学表现**

- **X光、CT、MRI**：显示骨破坏、硬化或骨膜反应等炎症性改变。

6. **实验室检查**

- **炎症标志物**：如C反应蛋白（CRP）和血沉（ESR）可能升高，但无特异性。

总结：成人慢性非细菌性骨炎最常见的表现是局部骨痛、肿胀和压痛，常伴有活动受限，部分患者可能出现疲劳和低热等全身症状，影像学检查显示骨炎症性改变，实验室检查可能显示炎症标志物升高。

4成人慢性非细菌性骨炎最常受累的部位是那些？


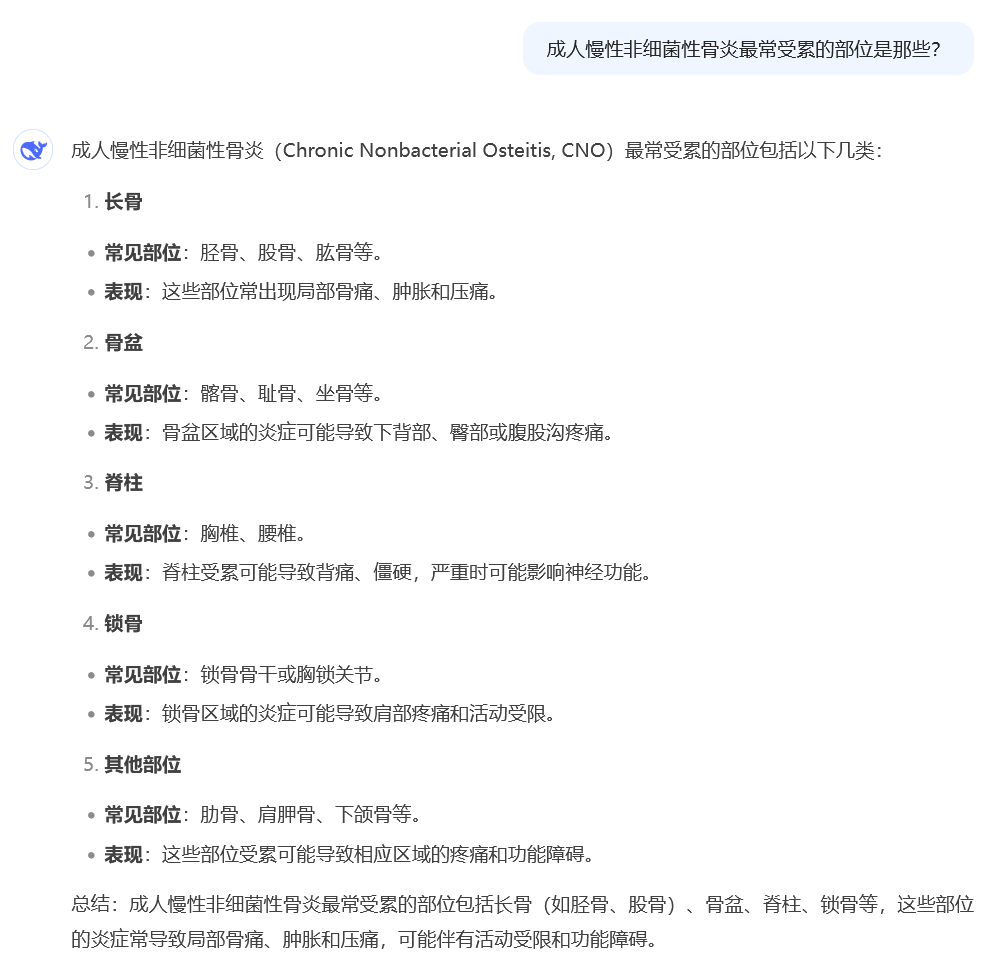


成人慢性非细菌性骨炎（Chronic Nonbacterial Osteitis, CNO）最常受累的部位包括以下几类：

1. **长骨**

- **常见部位**：胫骨、股骨、肱骨等。

- **表现**：这些部位常出现局部骨痛、肿胀和压痛。

2. **骨盆**

- **常见部位**：髂骨、耻骨、坐骨等。

- **表现**：骨盆区域的炎症可能导致下背部、臀部或腹股沟疼痛。

3. **脊柱**

- **常见部位**：胸椎、腰椎。

- **表现**：脊柱受累可能导致背痛、僵硬，严重时可能影响神经功能。

4. **锁骨**

- **常见部位**：锁骨骨干或胸锁关节。

- **表现**：锁骨区域的炎症可能导致肩部疼痛和活动受限。

5. **其他部位**

- **常见部位**：肋骨、肩胛骨、下颌骨等。

- **表现**：这些部位受累可能导致相应区域的疼痛和功能障碍。

总结：成人慢性非细菌性骨炎最常受累的部位包括长骨（如胫骨、股骨）、骨盆、脊柱、锁骨等，这些部位的炎症常导致局部骨痛、肿胀和压痛，可能伴有活动受限和功能障碍。

5成人慢性非细菌性骨炎有没有特异性的炎症标志物？


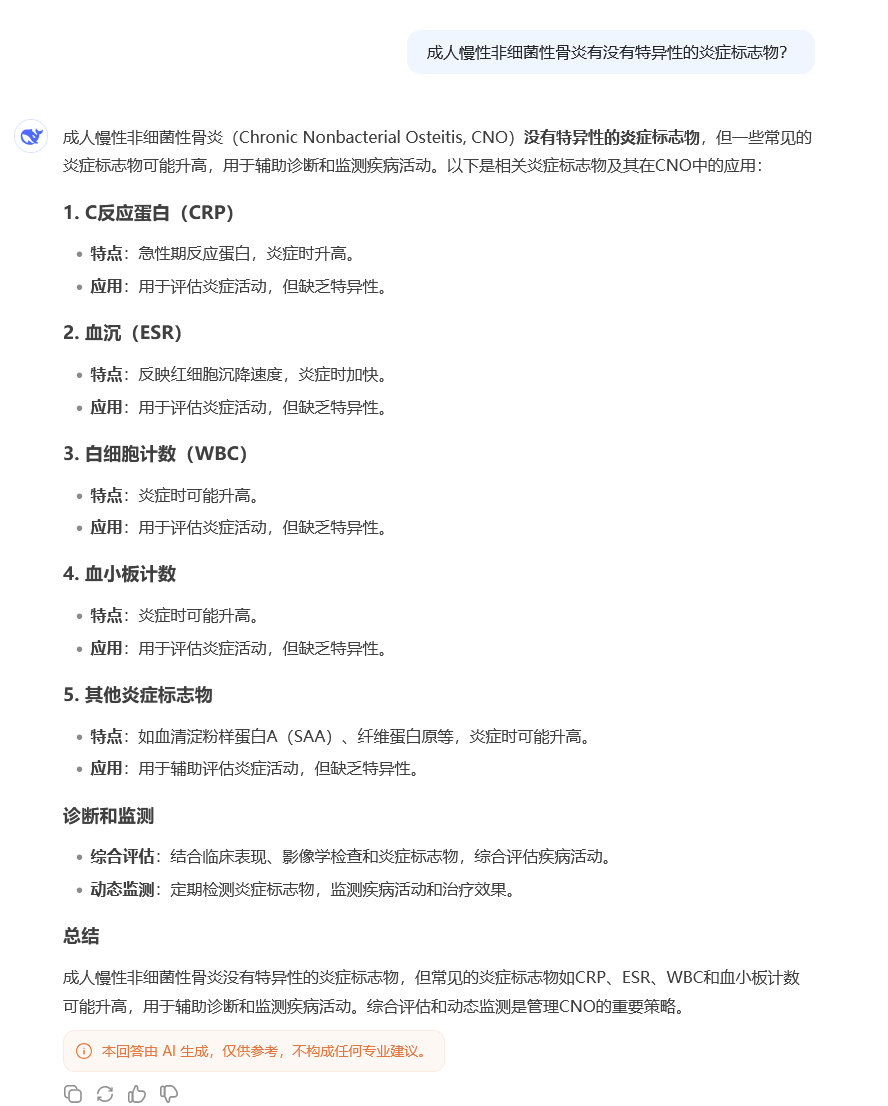


成人慢性非细菌性骨炎（Chronic Nonbacterial Osteitis, CNO）**没有特异性的炎症标志物**，但一些常见的炎症标志物可能升高，用于辅助诊断和监测疾病活动。以下是相关炎症标志物及其在CNO中的应用：

### 1. **C反应蛋白（CRP）**

- **特点**：急性期反应蛋白，炎症时升高。

- **应用**：用于评估炎症活动，但缺乏特异性。

### 2. **血沉（ESR）**

- **特点**：反映红细胞沉降速度，炎症时加快。

- **应用**：用于评估炎症活动，但缺乏特异性。

### 3. **白细胞计数（WBC）**

- **特点**：炎症时可能升高。

- **应用**：用于评估炎症活动，但缺乏特异性。

### 4. **血小板计数**

- **特点**：炎症时可能升高。

- **应用**：用于评估炎症活动，但缺乏特异性。

### 5. **其他炎症标志物**

- **特点**：如血清淀粉样蛋白A（SAA）、纤维蛋白原等，炎症时可能升高。

- **应用**：用于辅助评估炎症活动，但缺乏特异性。

### 诊断和监测

- **综合评估**：结合临床表现、影像学检查和炎症标志物，综合评估疾病活动。

- **动态监测**：定期检测炎症标志物，监测疾病活动和治疗效果。

### 总结

成人慢性非细菌性骨炎没有特异性的炎症标志物，但常见的炎症标志物如CRP、ESR、WBC和血小板计数可能升高，用于辅助诊断和监测疾病活动。综合评估和动态监测是管理CNO的重要策略。

6慢性非细菌性骨炎首选的影像学检查是什么？


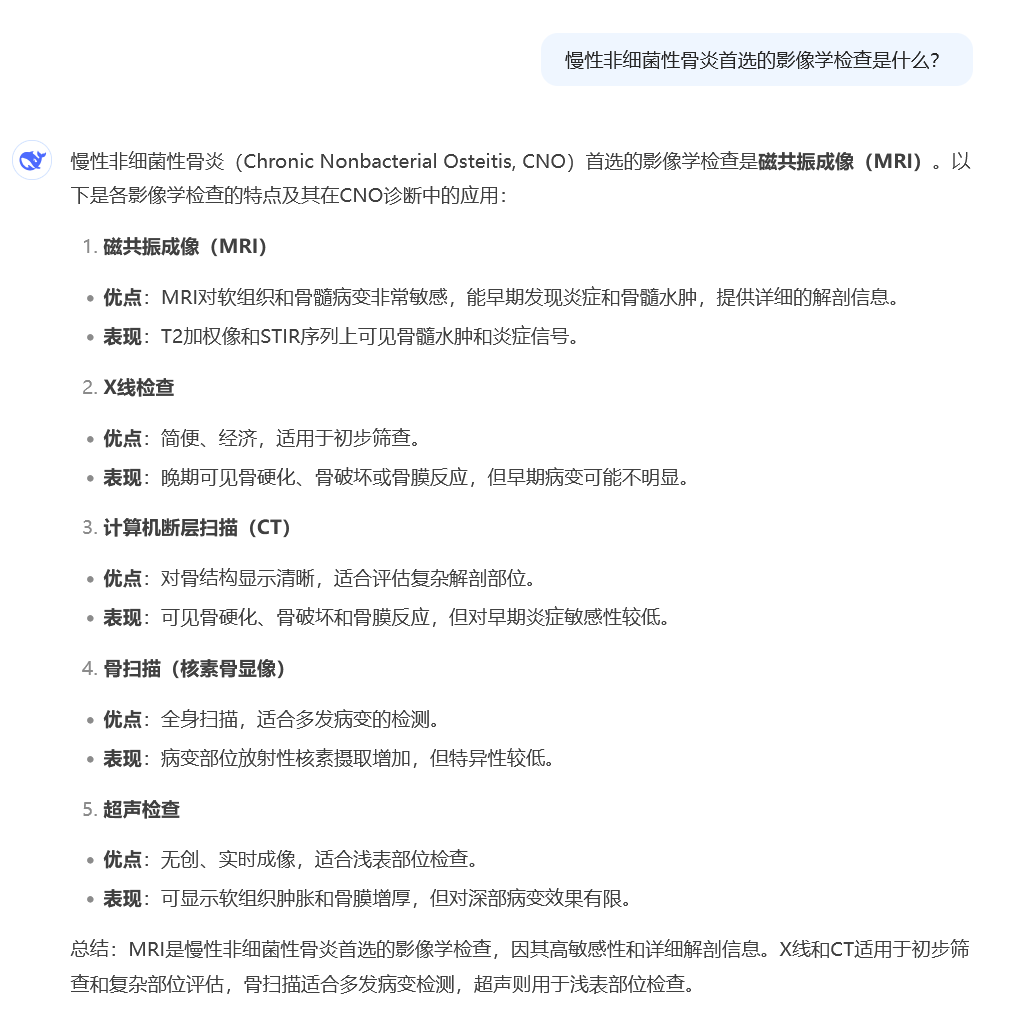


慢性非细菌性骨炎（Chronic Nonbacterial Osteitis, CNO）首选的影像学检查是**磁共振成像（MRI）**。以下是各影像学检查的特点及其在CNO诊断中的应用：

1. **磁共振成像（MRI）**

- **优点**：MRI对软组织和骨髓病变非常敏感，能早期发现炎症和骨髓水肿，提供详细的解剖信息。

- **表现**：T2加权像和STIR序列上可见骨髓水肿和炎症信号。

2. **X线检查**

- **优点**：简便、经济，适用于初步筛查。

- **表现**：晚期可见骨硬化、骨破坏或骨膜反应，但早期病变可能不明显。

3. **计算机断层扫描（CT）**

- **优点**：对骨结构显示清晰，适合评估复杂解剖部位。

- **表现**：可见骨硬化、骨破坏和骨膜反应，但对早期炎症敏感性较低。

4. **骨扫描（核素骨显像）**

- **优点**：全身扫描，适合多发病变的检测。

- **表现**：病变部位放射性核素摄取增加，但特异性较低。

5. **超声检查**

- **优点**：无创、实时成像，适合浅表部位检查。

- **表现**：可显示软组织肿胀和骨膜增厚，但对深部病变效果有限。

总结：MRI是慢性非细菌性骨炎首选的影像学检查，因其高敏感性和详细解剖信息。X线和CT适用于初步筛查和复杂部位评估，骨扫描适合多发病变检测，超声则用于浅表部位检查。

7全身成像检查是不是诊断成人慢性非细菌性骨炎的先决条件？


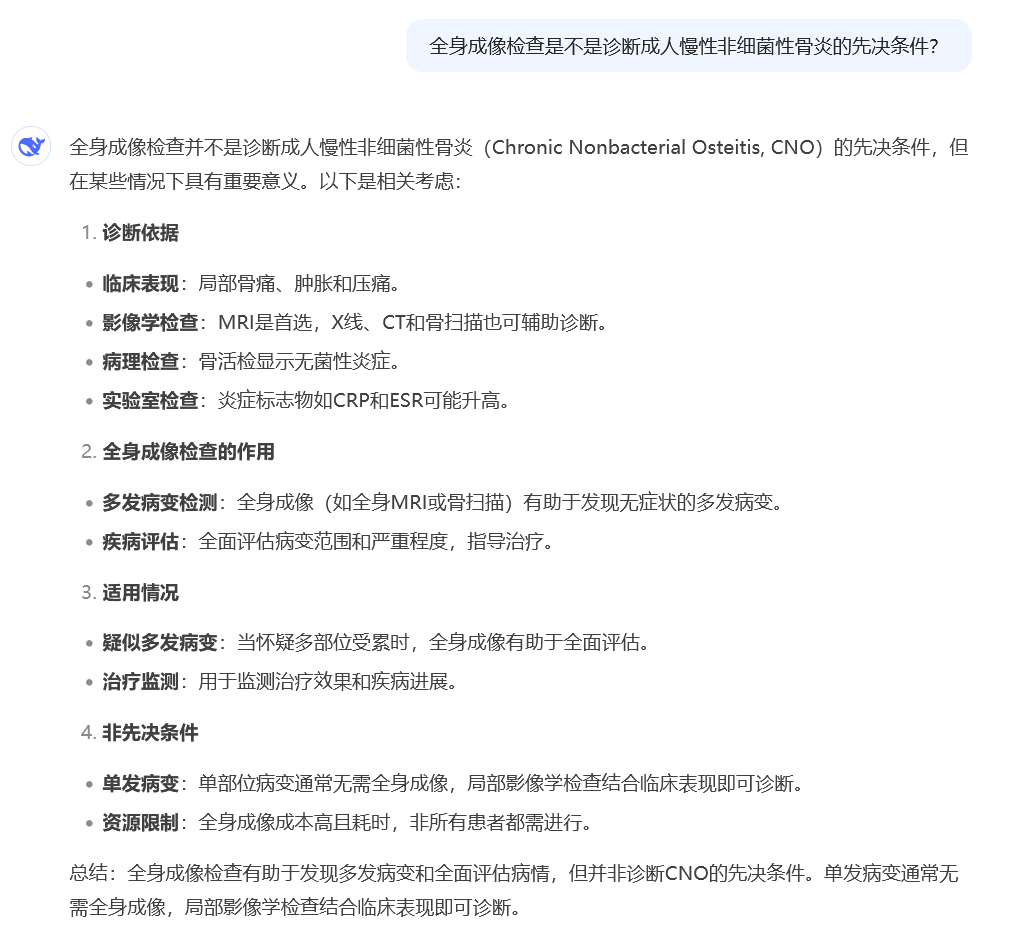


全身成像检查并不是诊断成人慢性非细菌性骨炎（Chronic Nonbacterial Osteitis, CNO）的先决条件，但在某些情况下具有重要意义。以下是相关考虑：

1. **诊断依据**

- **临床表现**：局部骨痛、肿胀和压痛。

- **影像学检查**：MRI是首选，X线、CT和骨扫描也可辅助诊断。

- **病理检查**：骨活检显示无菌性炎症。

- **实验室检查**：炎症标志物如CRP和ESR可能升高。

2. **全身成像检查的作用**

- **多发病变检测**：全身成像（如全身MRI或骨扫描）有助于发现无症状的多发病变。

- **疾病评估**：全面评估病变范围和严重程度，指导治疗。

3. **适用情况**

- **疑似多发病变**：当怀疑多部位受累时，全身成像有助于全面评估。

- **治疗监测**：用于监测治疗效果和疾病进展。

4. **非先决条件**

- **单发病变**：单部位病变通常无需全身成像，局部影像学检查结合临床表现即可诊断。

- **资源限制**：全身成像成本高且耗时，非所有患者都需进行。

总结：全身成像检查有助于发现多发病变和全面评估病情，但并非诊断CNO的先决条件。单发病变通常无需全身成像，局部影像学检查结合临床表现即可诊断。

8诊断成人慢性非细菌性骨炎有必要常规骨活检吗？


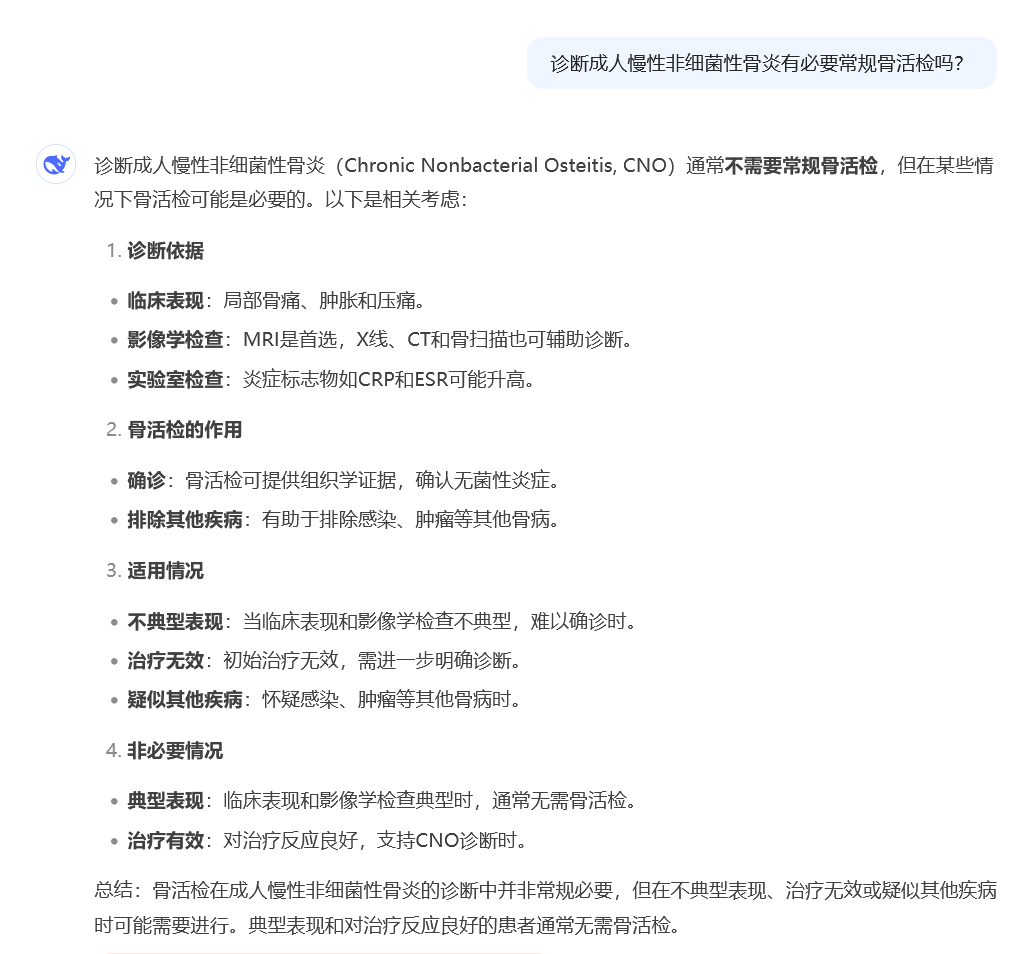


诊断成人慢性非细菌性骨炎（Chronic Nonbacterial Osteitis, CNO）通常**不需要常规骨活检**，但在某些情况下骨活检可能是必要的。以下是相关考虑：

1. **诊断依据**

- **临床表现**：局部骨痛、肿胀和压痛。

- **影像学检查**：MRI是首选，X线、CT和骨扫描也可辅助诊断。

- **实验室检查**：炎症标志物如CRP和ESR可能升高。

2. **骨活检的作用**

- **确诊**：骨活检可提供组织学证据，确认无菌性炎症。

- **排除其他疾病**：有助于排除感染、肿瘤等其他骨病。

3. **适用情况**

- **不典型表现**：当临床表现和影像学检查不典型，难以确诊时。

- **治疗无效**：初始治疗无效，需进一步明确诊断。

- **疑似其他疾病**：怀疑感染、肿瘤等其他骨病时。

4. **非必要情况**

- **典型表现**：临床表现和影像学检查典型时，通常无需骨活检。

- **治疗有效**：对治疗反应良好，支持CNO诊断时。

总结：骨活检在成人慢性非细菌性骨炎的诊断中并非常规必要，但在不典型表现、治疗无效或疑似其他疾病时可能需要进行。典型表现和对治疗反应良好的患者通常无需骨活检。

9慢性非细菌性骨炎如何与恶性骨肿瘤鉴别？


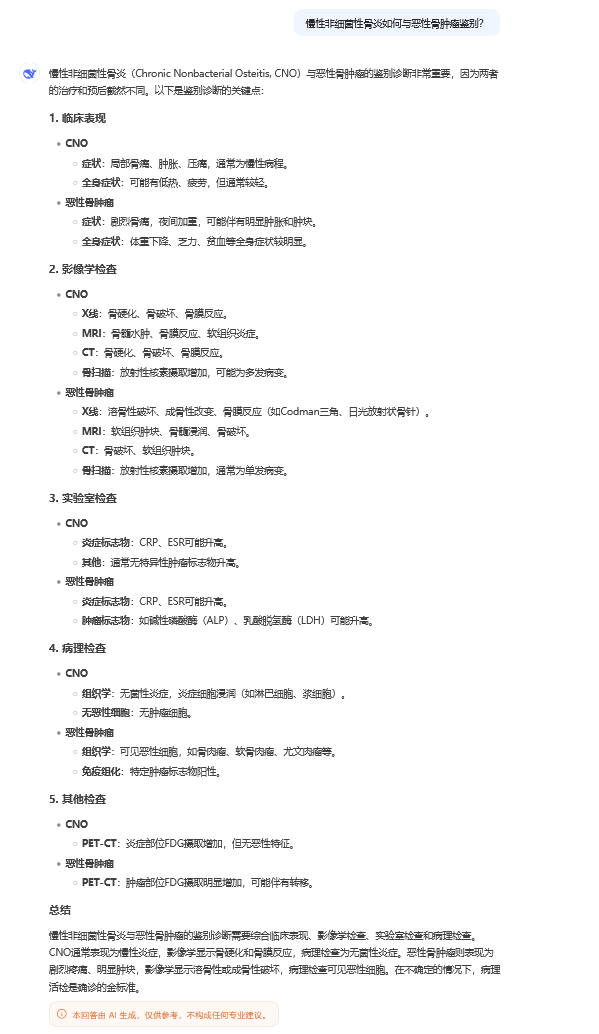


慢性非细菌性骨炎（Chronic Nonbacterial Osteitis, CNO）与恶性骨肿瘤的鉴别诊断非常重要，因为两者的治疗和预后截然不同。以下是鉴别诊断的关键点：

### 1. **临床表现**

- **CNO**

- **症状**：局部骨痛、肿胀、压痛，通常为慢性病程。

- **全身症状**：可能有低热、疲劳，但通常较轻。

- **恶性骨肿瘤**

- **症状**：剧烈骨痛，夜间加重，可能伴有明显肿胀和肿块。

- **全身症状**：体重下降、乏力、贫血等全身症状较明显。

### 2. **影像学检查**

- **CNO**

- **X线**：骨硬化、骨破坏、骨膜反应。

- **MRI**：骨髓水肿、骨膜反应、软组织炎症。

- **CT**：骨硬化、骨破坏、骨膜反应。

- **骨扫描**：放射性核素摄取增加，可能为多发病变。

- **恶性骨肿瘤**

- **X线**：溶骨性破坏、成骨性改变、骨膜反应（如Codman三角、日光放射状骨针）。

- **MRI**：软组织肿块、骨髓浸润、骨破坏。

- **CT**：骨破坏、软组织肿块。

- **骨扫描**：放射性核素摄取增加，通常为单发病变。

### 3. **实验室检查**

- **CNO**

- **炎症标志物**：CRP、ESR可能升高。

- **其他**：通常无特异性肿瘤标志物升高。

- **恶性骨肿瘤**

- **炎症标志物**：CRP、ESR可能升高。

- **肿瘤标志物**：如碱性磷酸酶（ALP）、乳酸脱氢酶（LDH）可能升高。

### 4. **病理检查**

- **CNO**

- **组织学**：无菌性炎症，炎症细胞浸润（如淋巴细胞、浆细胞）。

- **无恶性细胞**：无肿瘤细胞。

- **恶性骨肿瘤**

- **组织学**：可见恶性细胞，如骨肉瘤、软骨肉瘤、尤文肉瘤等。

- **免疫组化**：特定肿瘤标志物阳性。

### 5. **其他检查**

- **CNO**

- **PET-CT**：炎症部位FDG摄取增加，但无恶性特征。

- **恶性骨肿瘤**

- **PET-CT**：肿瘤部位FDG摄取明显增加，可能伴有转移。

### 总结

慢性非细菌性骨炎与恶性骨肿瘤的鉴别诊断需要综合临床表现、影像学检查、实验室检查和病理检查。CNO通常表现为慢性炎症，影像学显示骨硬化和骨膜反应，病理检查为无菌性炎症。恶性骨肿瘤则表现为剧烈疼痛、明显肿块，影像学显示溶骨性或成骨性破坏，病理检查可见恶性细胞。在不确定的情况下，病理活检是确诊的金标准。

10慢性非细菌性骨炎如何与骨关节炎鉴别？


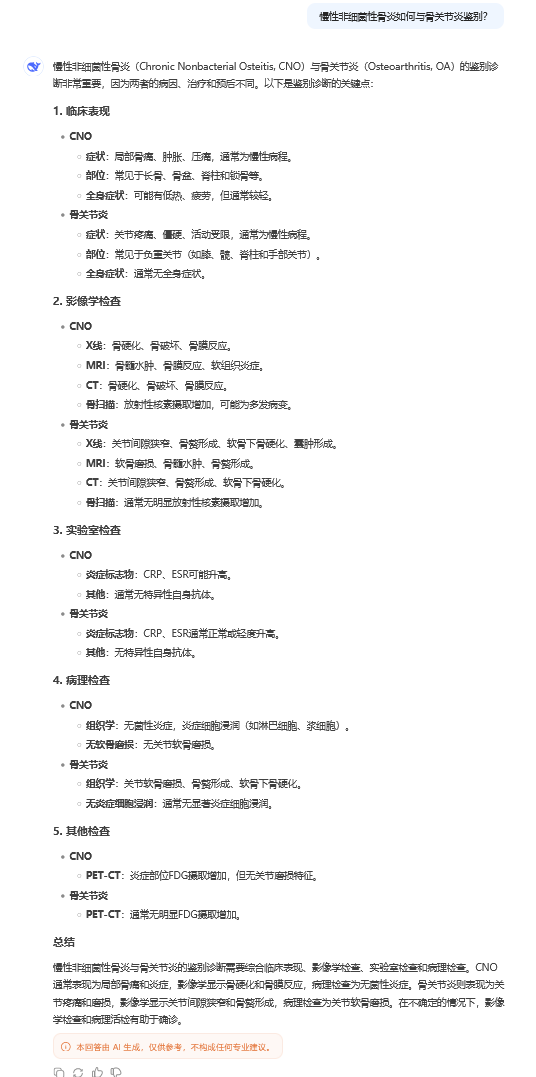


慢性非细菌性骨炎（Chronic Nonbacterial Osteitis, CNO）与骨关节炎（Osteoarthritis, OA）的鉴别诊断非常重要，因为两者的病因、治疗和预后不同。以下是鉴别诊断的关键点：

### 1. **临床表现**

- **CNO**

- **症状**：局部骨痛、肿胀、压痛，通常为慢性病程。

- **部位**：常见于长骨、骨盆、脊柱和锁骨等。

- **全身症状**：可能有低热、疲劳，但通常较轻。

- **骨关节炎**

- **症状**：关节疼痛、僵硬、活动受限，通常为慢性病程。

- **部位**：常见于负重关节（如膝、髋、脊柱和手部关节）。

- **全身症状**：通常无全身症状。

### 2. **影像学检查**

- **CNO**

- **X线**：骨硬化、骨破坏、骨膜反应。

- **MRI**：骨髓水肿、骨膜反应、软组织炎症。

- **CT**：骨硬化、骨破坏、骨膜反应。

- **骨扫描**：放射性核素摄取增加，可能为多发病变。

- **骨关节炎**

- **X线**：关节间隙狭窄、骨赘形成、软骨下骨硬化、囊肿形成。

- **MRI**：软骨磨损、骨髓水肿、骨赘形成。

- **CT**：关节间隙狭窄、骨赘形成、软骨下骨硬化。

- **骨扫描**：通常无明显放射性核素摄取增加。

### 3. **实验室检查**

- **CNO**

- **炎症标志物**：CRP、ESR可能升高。

- **其他**：通常无特异性自身抗体。

- **骨关节炎**

- **炎症标志物**：CRP、ESR通常正常或轻度升高。

- **其他**：无特异性自身抗体。

### 4. **病理检查**

- **CNO**

- **组织学**：无菌性炎症，炎症细胞浸润（如淋巴细胞、浆细胞）。

- **无软骨磨损**：无关节软骨磨损。

- **骨关节炎**

- **组织学**：关节软骨磨损、骨赘形成、软骨下骨硬化。

- **无炎症细胞浸润**：通常无显著炎症细胞浸润。

### 5. **其他检查**

- **CNO**

- **PET-CT**：炎症部位FDG摄取增加，但无关节磨损特征。

- **骨关节炎**

- **PET-CT**：通常无明显FDG摄取增加。

### 总结

慢性非细菌性骨炎与骨关节炎的鉴别诊断需要综合临床表现、影像学检查、实验室检查和病理检查。CNO通常表现为局部骨痛和炎症，影像学显示骨硬化和骨膜反应，病理检查为无菌性炎症。骨关节炎则表现为关节疼痛和磨损，影像学显示关节间隙狭窄和骨赘形成，病理检查为关节软骨磨损。在不确定的情况下，影像学检查和病理活检有助于确诊。

11慢性非细菌性骨炎如何与纤维发育不良鉴别？


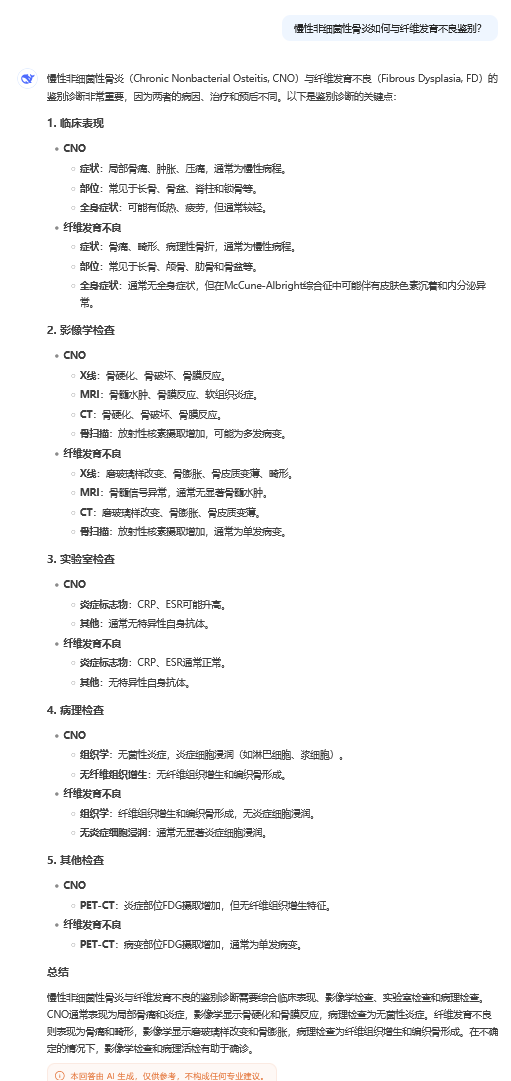


慢性非细菌性骨炎（Chronic Nonbacterial Osteitis, CNO）与纤维发育不良（Fibrous Dysplasia, FD）的鉴别诊断非常重要，因为两者的病因、治疗和预后不同。以下是鉴别诊断的关键点：

### 1. **临床表现**

- **CNO**

- **症状**：局部骨痛、肿胀、压痛，通常为慢性病程。

- **部位**：常见于长骨、骨盆、脊柱和锁骨等。

- **全身症状**：可能有低热、疲劳，但通常较轻。

- **纤维发育不良**

- **症状**：骨痛、畸形、病理性骨折，通常为慢性病程。

- **部位**：常见于长骨、颅骨、肋骨和骨盆等。

- **全身症状**：通常无全身症状，但在McCune-Albright综合征中可能伴有皮肤色素沉着和内分泌异常。

### 2. **影像学检查**

- **CNO**

- **X线**：骨硬化、骨破坏、骨膜反应。

- **MRI**：骨髓水肿、骨膜反应、软组织炎症。

- **CT**：骨硬化、骨破坏、骨膜反应。

- **骨扫描**：放射性核素摄取增加，可能为多发病变。

- **纤维发育不良**

- **X线**：磨玻璃样改变、骨膨胀、骨皮质变薄、畸形。

- **MRI**：骨髓信号异常，通常无显著骨髓水肿。

- **CT**：磨玻璃样改变、骨膨胀、骨皮质变薄。

- **骨扫描**：放射性核素摄取增加，通常为单发病变。

### 3. **实验室检查**

- **CNO**

- **炎症标志物**：CRP、ESR可能升高。

- **其他**：通常无特异性自身抗体。

- **纤维发育不良**

- **炎症标志物**：CRP、ESR通常正常。

- **其他**：无特异性自身抗体。

### 4. **病理检查**

- **CNO**

- **组织学**：无菌性炎症，炎症细胞浸润（如淋巴细胞、浆细胞）。

- **无纤维组织增生**：无纤维组织增生和编织骨形成。

- **纤维发育不良**

- **组织学**：纤维组织增生和编织骨形成，无炎症细胞浸润。

- **无炎症细胞浸润**：通常无显著炎症细胞浸润。

### 5. **其他检查**

- **CNO**

- **PET-CT**：炎症部位FDG摄取增加，但无纤维组织增生特征。

- **纤维发育不良**

- **PET-CT**：病变部位FDG摄取增加，通常为单发病变。

### 总结

慢性非细菌性骨炎与纤维发育不良的鉴别诊断需要综合临床表现、影像学检查、实验室检查和病理检查。CNO通常表现为局部骨痛和炎症，影像学显示骨硬化和骨膜反应，病理检查为无菌性炎症。纤维发育不良则表现为骨痛和畸形，影像学显示磨玻璃样改变和骨膨胀，病理检查为纤维组织增生和编织骨形成。在不确定的情况下，影像学检查和病理活检有助于确诊。

12在治慢性非细菌性骨炎过程中应该选择短疗程的糖皮质激素治疗还是长期的糖皮质激素治疗？？


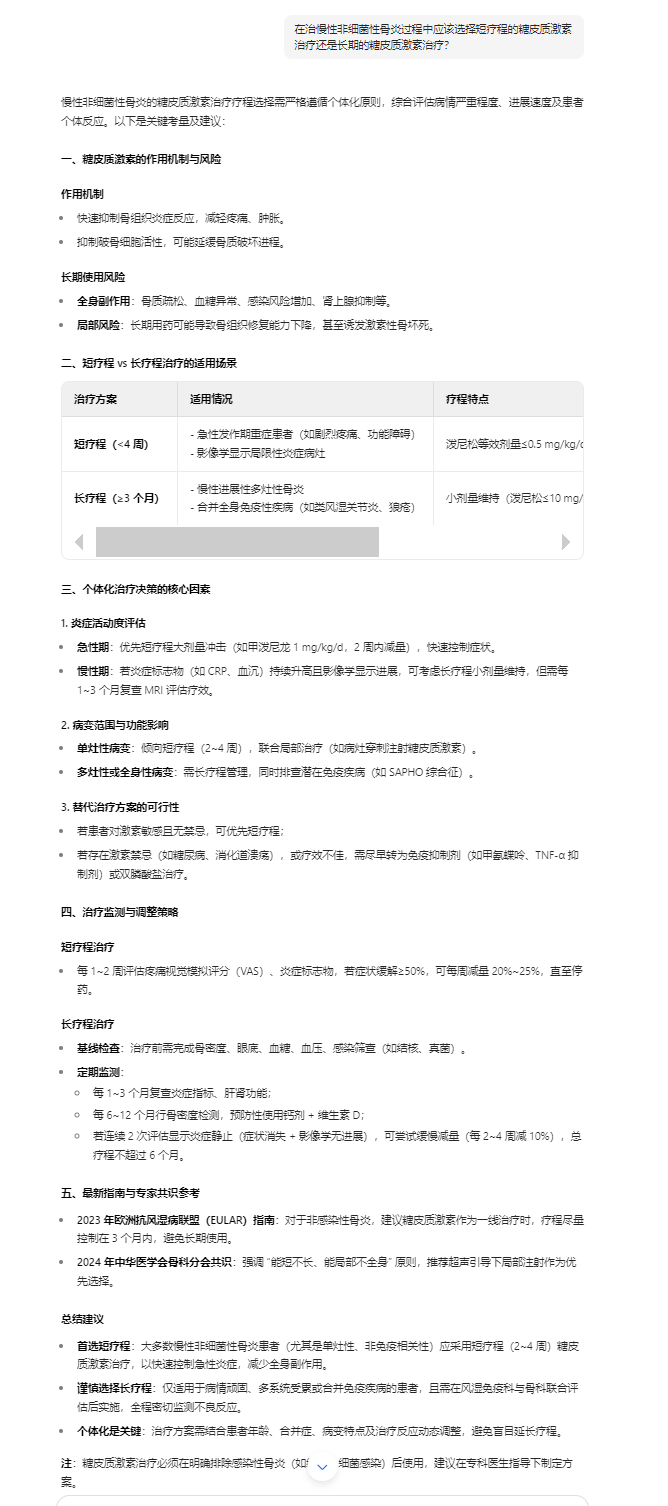


慢性非细菌性骨炎的糖皮质激素治疗疗程选择需严格遵循个体化原则，综合评估病情严重程度、进展速度及患者个体反应。以下是关键考量及建议：

### **一、糖皮质激素的作用机制与风险**

#### **作用机制**

- 快速抑制骨组织炎症反应，减轻疼痛、肿胀。

- 抑制破骨细胞活性，可能延缓骨质破坏进程。

#### **长期使用风险**

- **全身副作用**：骨质疏松、血糖异常、感染风险增加、肾上腺抑制等。

- **局部风险**：长期用药可能导致骨组织修复能力下降，甚至诱发激素性骨坏死。

### **二、短疗程 vs 长疗程治疗的适用场景**

| **治疗方案** | **适用情况** | **疗程特点** | **注意事项** |

|--------------------|-----------------------------------------------------------------------------|---------------------------------------|-------------------------------------------|

| **短疗程（<4周）** | - 急性发作期重症患者（如剧烈疼痛、功能障碍）<br>- 影像学显示局限性炎症病灶 | 泼尼松等效剂量≤0.5 mg/kg/d，快速减量 | 需联合非甾体抗炎药（NSAIDs）或物理治疗 |

| **长疗程（≥3个月）** | - 慢性进展性多灶性骨炎<br>- 合并全身免疫性疾病（如类风湿关节炎、狼疮） | 小剂量维持（泼尼松≤10 mg/d），定期评估 | 必须严格监测骨密度、血糖、感染指标等 |

### **三、个体化治疗决策的核心因素**

#### **1. 炎症活动度评估**

- **急性期**：优先短疗程大剂量冲击（如甲泼尼龙1 mg/kg/d，2周内减量），快速控制症状。

- **慢性期**：若炎症标志物（如CRP、血沉）持续升高且影像学显示进展，可考虑长疗程小剂量维持，但需每1~3个月复查MRI评估疗效。

#### **2. 病变范围与功能影响**

- **单灶性病变**：倾向短疗程（2~4周），联合局部治疗（如病灶穿刺注射糖皮质激素）。

- **多灶性或全身性病变**：需长疗程管理，同时排查潜在免疫疾病（如SAPHO综合征）。

#### **3. 替代治疗方案的可行性**

- 若患者对激素敏感且无禁忌，可优先短疗程；

- 若存在激素禁忌（如糖尿病、消化道溃疡），或疗效不佳，需尽早转为免疫抑制剂（如甲氨蝶呤、TNF-α抑制剂）或双膦酸盐治疗。

### **四、治疗监测与调整策略**

#### **短疗程治疗**

- 每1~2周评估疼痛视觉模拟评分（VAS）、炎症标志物，若症状缓解≥50%，可每周减量20%~25%，直至停药。

#### **长疗程治疗**

- **基线检查**：治疗前需完成骨密度、眼底、血糖、血压、感染筛查（如结核、真菌）。

- **定期监测**：

- 每1~3个月复查炎症指标、肝肾功能；

- 每6~12个月行骨密度检测，预防性使用钙剂+维生素D；

- 若连续2次评估显示炎症静止（症状消失+影像学无进展），可尝试缓慢减量（每2~4周减10%），总疗程不超过6个月。

### **五、最新指南与专家共识参考**

- **2023年欧洲抗风湿病联盟（EULAR）指南**：对于非感染性骨炎，建议糖皮质激素作为一线治疗时，疗程尽量控制在3个月内，避免长期使用。

- **2024年中华医学会骨科分会共识**：强调“能短不长、能局部不全身”原则，推荐超声引导下局部注射作为优先选择。

### **总结建议**

- **首选短疗程**：大多数慢性非细菌性骨炎患者（尤其是单灶性、非免疫相关性）应采用短疗程（2~4周）糖皮质激素治疗，以快速控制急性炎症，减少全身副作用。

- **谨慎选择长疗程**：仅适用于病情顽固、多系统受累或合并免疫疾病的患者，且需在风湿免疫科与骨科联合评估后实施，全程密切监测不良反应。

- **个体化是关键**：治疗方案需结合患者年龄、合并症、病变特点及治疗反应动态调整，避免盲目延长疗程。

**注**：糖皮质激素治疗必须在明确排除感染性骨炎（如结核、细菌感染）后使用，建议在专科医生指导下制定方案。

13成人慢性非细菌性骨炎一线治疗方案是什么？


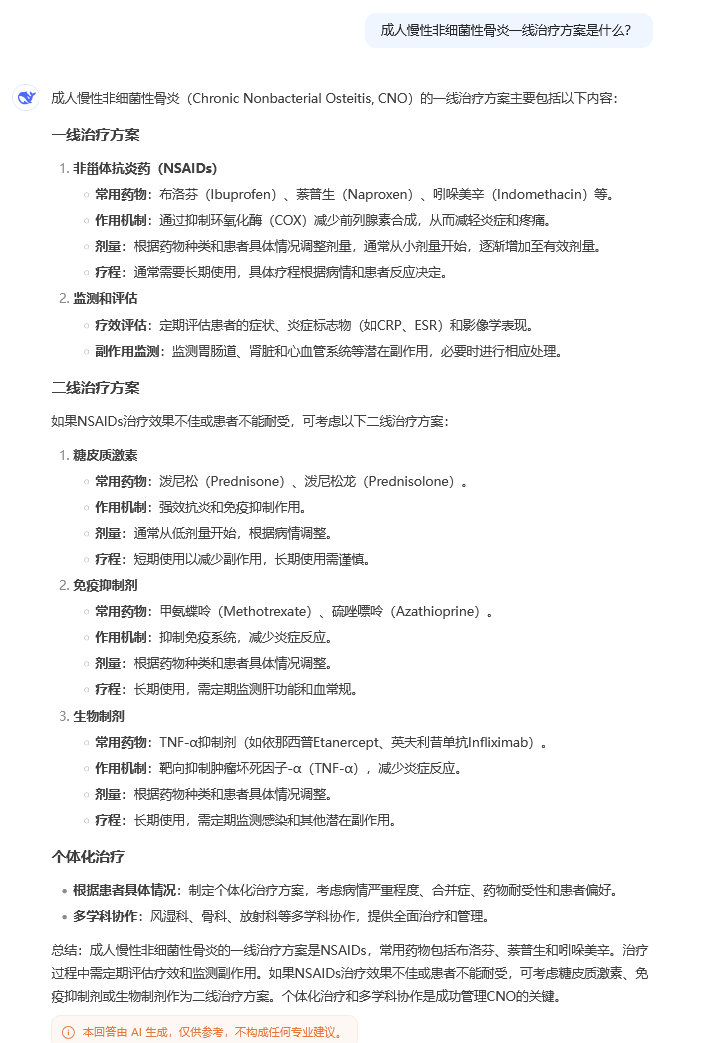


成人慢性非细菌性骨炎（Chronic Nonbacterial Osteitis, CNO）的一线治疗方案主要包括以下内容：

### 一线治疗方案

1. **非甾体抗炎药（NSAIDs）**

- **常用药物**：布洛芬（Ibuprofen）、萘普生（Naproxen）、吲哚美辛（Indomethacin）等。

- **作用机制**：通过抑制环氧化酶（COX）减少前列腺素合成，从而减轻炎症和疼痛。

- **剂量**：根据药物种类和患者具体情况调整剂量，通常从小剂量开始，逐渐增加至有效剂量。

- **疗程**：通常需要长期使用，具体疗程根据病情和患者反应决定。

2. **监测和评估**

- **疗效评估**：定期评估患者的症状、炎症标志物（如CRP、ESR）和影像学表现。

- **副作用监测**：监测胃肠道、肾脏和心血管系统等潜在副作用，必要时进行相应处理。

### 二线治疗方案

如果NSAIDs治疗效果不佳或患者不能耐受，可考虑以下二线治疗方案：

1. **糖皮质激素**

- **常用药物**：泼尼松（Prednisone）、泼尼松龙（Prednisolone）。

- **作用机制**：强效抗炎和免疫抑制作用。

- **剂量**：通常从低剂量开始，根据病情调整。

- **疗程**：短期使用以减少副作用，长期使用需谨慎。

2. **免疫抑制剂**

- **常用药物**：甲氨蝶呤（Methotrexate）、硫唑嘌呤（Azathioprine）。

- **作用机制**：抑制免疫系统，减少炎症反应。

- **剂量**：根据药物种类和患者具体情况调整。

- **疗程**：长期使用，需定期监测肝功能和血常规。

3. **生物制剂**

- **常用药物**：TNF-α抑制剂（如依那西普Etanercept、英夫利昔单抗Infliximab）。

- **作用机制**：靶向抑制肿瘤坏死因子-α（TNF-α），减少炎症反应。

- **剂量**：根据药物种类和患者具体情况调整。

- **疗程**：长期使用，需定期监测感染和其他潜在副作用。

### 个体化治疗

- **根据患者具体情况**：制定个体化治疗方案，考虑病情严重程度、合并症、药物耐受性和患者偏好。

- **多学科协作**：风湿科、骨科、放射科等多学科协作，提供全面治疗和管理。

总结：成人慢性非细菌性骨炎的一线治疗方案是NSAIDs，常用药物包括布洛芬、萘普生和吲哚美辛。治疗过程中需定期评估疗效和监测副作用。如果NSAIDs治疗效果不佳或患者不能耐受，可考虑糖皮质激素、免疫抑制剂或生物制剂作为二线治疗方案。个体化治疗和多学科协作是成功管理CNO的关键。

14无临床症状，也没有放射学疾病活动的慢性非细菌性骨炎的病人需要治疗吗？


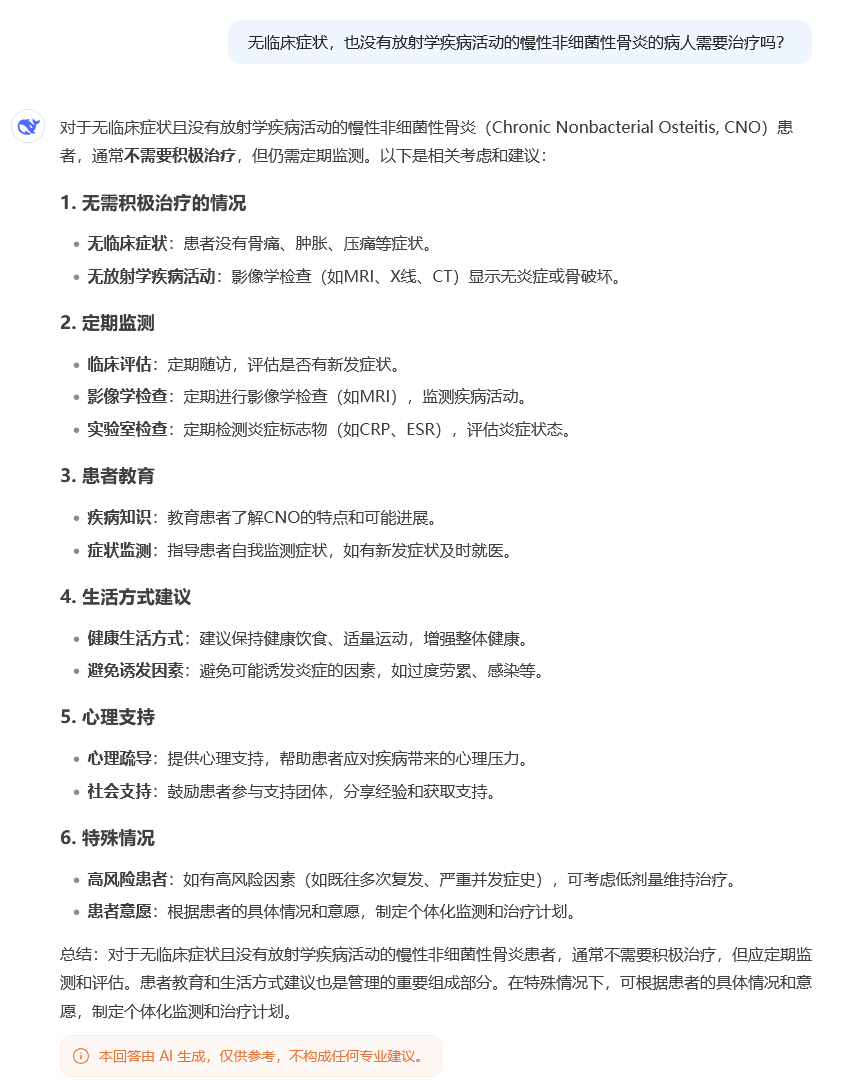


对于无临床症状且没有放射学疾病活动的慢性非细菌性骨炎（Chronic Nonbacterial Osteitis, CNO）患者，通常**不需要积极治疗**，但仍需定期监测。以下是相关考虑和建议：

### 1. **无需积极治疗的情况**

- **无临床症状**：患者没有骨痛、肿胀、压痛等症状。

- **无放射学疾病活动**：影像学检查（如MRI、X线、CT）显示无炎症或骨破坏。

### 2. **定期监测**

- **临床评估**：定期随访，评估是否有新发症状。

- **影像学检查**：定期进行影像学检查（如MRI），监测疾病活动。

- **实验室检查**：定期检测炎症标志物（如CRP、ESR），评估炎症状态。

### 3. **患者教育**

- **疾病知识**：教育患者了解CNO的特点和可能进展。

- **症状监测**：指导患者自我监测症状，如有新发症状及时就医。

### 4. **生活方式建议**

- **健康生活方式**：建议保持健康饮食、适量运动，增强整体健康。

- **避免诱发因素**：避免可能诱发炎症的因素，如过度劳累、感染等。

### 5. **心理支持**

- **心理疏导**：提供心理支持，帮助患者应对疾病带来的心理压力。

- **社会支持**：鼓励患者参与支持团体，分享经验和获取支持。

### 6. **特殊情况**

- **高风险患者**：如有高风险因素（如既往多次复发、严重并发症史），可考虑低剂量维持治疗。

- **患者意愿**：根据患者的具体情况和意愿，制定个体化监测和治疗计划。

总结：对于无临床症状且没有放射学疾病活动的慢性非细菌性骨炎患者，通常不需要积极治疗，但应定期监测和评估。患者教育和生活方式建议也是管理的重要组成部分。在特殊情况下，可根据患者的具体情况和意愿，制定个体化监测和治疗计划。

15有临床症状没有放射学疾病活动的慢性非细菌性骨炎的病人如何处理？


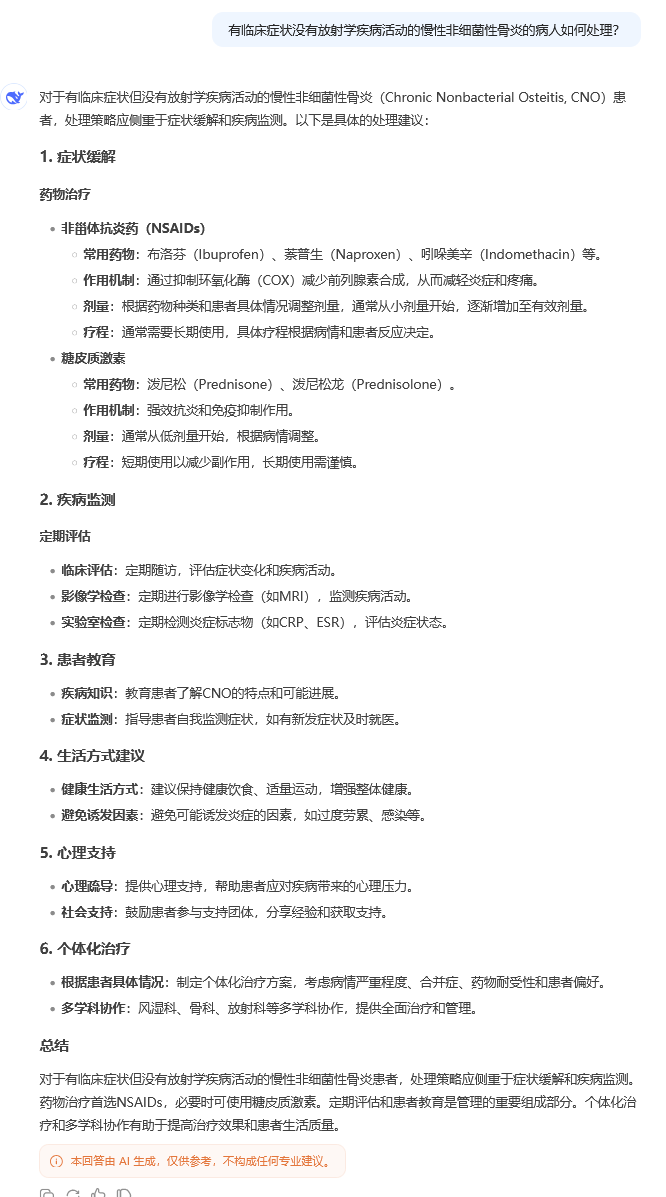


对于有临床症状但没有放射学疾病活动的慢性非细菌性骨炎（Chronic Nonbacterial Osteitis, CNO）患者，处理策略应侧重于症状缓解和疾病监测。以下是具体的处理建议：

### 1. **症状缓解**

#### 药物治疗

- **非甾体抗炎药（NSAIDs）**

- **常用药物**：布洛芬（Ibuprofen）、萘普生（Naproxen）、吲哚美辛（Indomethacin）等。

- **作用机制**：通过抑制环氧化酶（COX）减少前列腺素合成，从而减轻炎症和疼痛。

- **剂量**：根据药物种类和患者具体情况调整剂量，通常从小剂量开始，逐渐增加至有效剂量。

- **疗程**：通常需要长期使用，具体疗程根据病情和患者反应决定。

- **糖皮质激素**

- **常用药物**：泼尼松（Prednisone）、泼尼松龙（Prednisolone）。

- **作用机制**：强效抗炎和免疫抑制作用。

- **剂量**：通常从低剂量开始，根据病情调整。

- **疗程**：短期使用以减少副作用，长期使用需谨慎。

### 2. **疾病监测**

#### 定期评估

- **临床评估**：定期随访，评估症状变化和疾病活动。

- **影像学检查**：定期进行影像学检查（如MRI），监测疾病活动。

- **实验室检查**：定期检测炎症标志物（如CRP、ESR），评估炎症状态。

### 3. **患者教育**

- **疾病知识**：教育患者了解CNO的特点和可能进展。

- **症状监测**：指导患者自我监测症状，如有新发症状及时就医。

### 4. **生活方式建议**

- **健康生活方式**：建议保持健康饮食、适量运动，增强整体健康。

- **避免诱发因素**：避免可能诱发炎症的因素，如过度劳累、感染等。

### 5. **心理支持**

- **心理疏导**：提供心理支持，帮助患者应对疾病带来的心理压力。

- **社会支持**：鼓励患者参与支持团体，分享经验和获取支持。

### 6. **个体化治疗**

- **根据患者具体情况**：制定个体化治疗方案，考虑病情严重程度、合并症、药物耐受性和患者偏好。

- **多学科协作**：风湿科、骨科、放射科等多学科协作，提供全面治疗和管理。

### 总结

对于有临床症状但没有放射学疾病活动的慢性非细菌性骨炎患者，处理策略应侧重于症状缓解和疾病监测。药物治疗首选NSAIDs，必要时可使用糖皮质激素。定期评估和患者教育是管理的重要组成部分。个体化治疗和多学科协作有助于提高治疗效果和患者生活质量。

16有椎体塌陷风险的脊柱骨病变的慢性非细菌性骨炎患者和出现明显骨骼累积损伤的慢性非细菌性骨炎患者如何治疗？


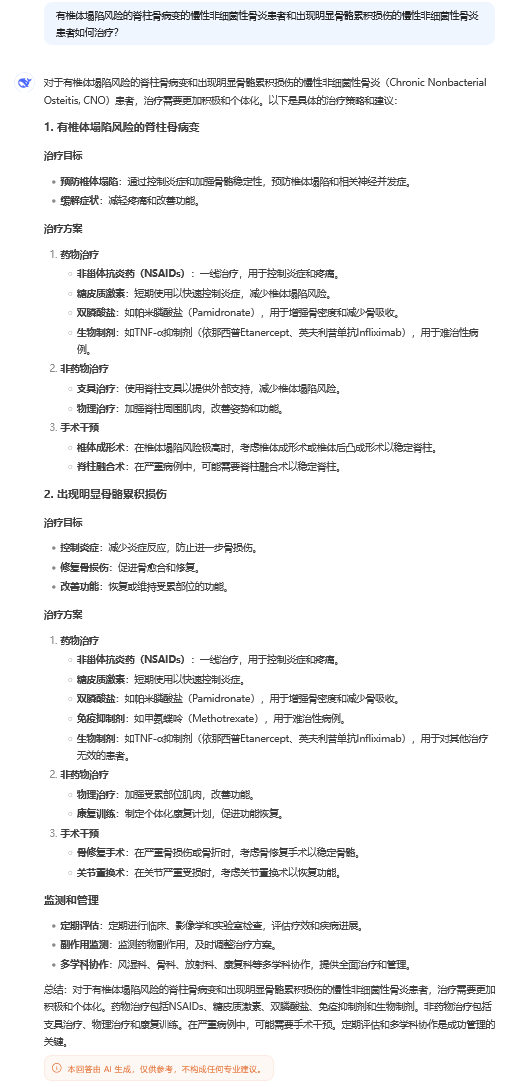


对于有椎体塌陷风险的脊柱骨病变和出现明显骨骼累积损伤的慢性非细菌性骨炎（Chronic Nonbacterial Osteitis, CNO）患者，治疗需要更加积极和个体化。以下是具体的治疗策略和建议：

### 1. **有椎体塌陷风险的脊柱骨病变**

#### 治疗目标

- **预防椎体塌陷**：通过控制炎症和加强骨骼稳定性，预防椎体塌陷和相关神经并发症。

- **缓解症状**：减轻疼痛和改善功能。

#### 治疗方案

1. **药物治疗**

- **非甾体抗炎药（NSAIDs）**：一线治疗，用于控制炎症和疼痛。

- **糖皮质激素**：短期使用以快速控制炎症，减少椎体塌陷风险。

- **双膦酸盐**：如帕米膦酸盐（Pamidronate），用于增强骨密度和减少骨吸收。

- **生物制剂**：如TNF-α抑制剂（依那西普Etanercept、英夫利昔单抗Infliximab），用于难治性病例。

2. **非药物治疗**

- **支具治疗**：使用脊柱支具以提供外部支持，减少椎体塌陷风险。

- **物理治疗**：加强脊柱周围肌肉，改善姿势和功能。

3. **手术干预**

- **椎体成形术**：在椎体塌陷风险极高时，考虑椎体成形术或椎体后凸成形术以稳定脊柱。

- **脊柱融合术**：在严重病例中，可能需要脊柱融合术以稳定脊柱。

### 2. **出现明显骨骼累积损伤**

#### 治疗目标

- **控制炎症**：减少炎症反应，防止进一步骨损伤。

- **修复骨损伤**：促进骨愈合和修复。

- **改善功能**：恢复或维持受累部位的功能。

#### 治疗方案

1. **药物治疗**

- **非甾体抗炎药（NSAIDs）**：一线治疗，用于控制炎症和疼痛。

- **糖皮质激素**：短期使用以快速控制炎症。

- **双膦酸盐**：如帕米膦酸盐（Pamidronate），用于增强骨密度和减少骨吸收。

- **免疫抑制剂**：如甲氨蝶呤（Methotrexate），用于难治性病例。

- **生物制剂**：如TNF-α抑制剂（依那西普Etanercept、英夫利昔单抗Infliximab），用于对其他治疗无效的患者。

2. **非药物治疗**

- **物理治疗**：加强受累部位肌肉，改善功能。

- **康复训练**：制定个体化康复计划，促进功能恢复。

3. **手术干预**

- **骨修复手术**：在严重骨损伤或骨折时，考虑骨修复手术以稳定骨骼。

- **关节置换术**：在关节严重受损时，考虑关节置换术以恢复功能。

### 监测和管理

- **定期评估**：定期进行临床、影像学和实验室检查，评估疗效和疾病进展。

- **副作用监测**：监测药物副作用，及时调整治疗方案。

- **多学科协作**：风湿科、骨科、放射科、康复科等多学科协作，提供全面治疗和管理。

总结：对于有椎体塌陷风险的脊柱骨病变和出现明显骨骼累积损伤的慢性非细菌性骨炎患者，治疗需要更加积极和个体化。药物治疗包括NSAIDs、糖皮质激素、双膦酸盐、免疫抑制剂和生物制剂。非药物治疗包括支具治疗、物理治疗和康复训练。在严重病例中，可能需要手术干预。定期评估和多学科协作是成功管理的关键。
