# Supplementary material for: Evaluation of the accuracy and repeatability of Deepseek V3, Doubao, and Kimi1.5 in answering knowledge-related queries about chronic non-bacterial osteitis
Source: Front Artif Intell. 2025 Sep 29;8:1629149. doi: 10.3389/frai.2025.1629149 (PMC12515971; doi:10.3389/frai.2025.1629149)
Supplement: Supplementary file 1 [file Data_Sheet_1.zip › day1/day1-Doubao.docx]

1成人慢性非细菌性骨炎的定义是什么？


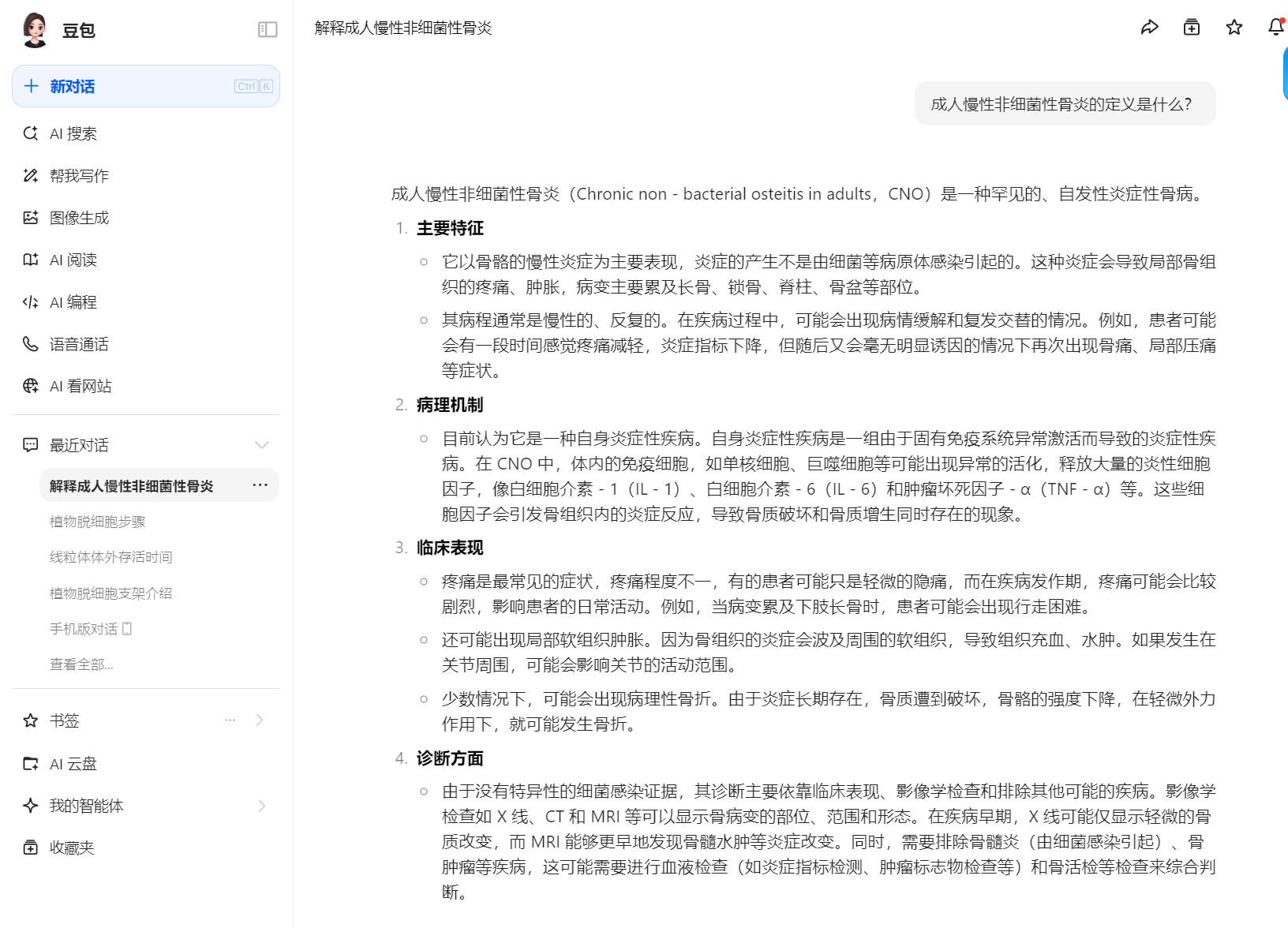


成人慢性非细菌性骨炎（Chronic non - bacterial osteitis in adults，CNO）是一种罕见的、自发性炎症性骨病。

1. **主要特征**

- 它以骨骼的慢性炎症为主要表现，炎症的产生不是由细菌等病原体感染引起的。这种炎症会导致局部骨组织的疼痛、肿胀，病变主要累及长骨、锁骨、脊柱、骨盆等部位。

- 其病程通常是慢性的、反复的。在疾病过程中，可能会出现病情缓解和复发交替的情况。例如，患者可能会有一段时间感觉疼痛减轻，炎症指标下降，但随后又会毫无明显诱因的情况下再次出现骨痛、局部压痛等症状。

2. **病理机制**

- 目前认为它是一种自身炎症性疾病。自身炎症性疾病是一组由于固有免疫系统异常激活而导致的炎症性疾病。在CNO中，体内的免疫细胞，如单核细胞、巨噬细胞等可能出现异常的活化，释放大量的炎性细胞因子，像白细胞介素 - 1（IL - 1）、白细胞介素 - 6（IL - 6）和肿瘤坏死因子 - α（TNF - α）等。这些细胞因子会引发骨组织内的炎症反应，导致骨质破坏和骨质增生同时存在的现象。

3. **临床表现**

- 疼痛是最常见的症状，疼痛程度不一，有的患者可能只是轻微的隐痛，而在疾病发作期，疼痛可能会比较剧烈，影响患者的日常活动。例如，当病变累及下肢长骨时，患者可能会出现行走困难。

- 还可能出现局部软组织肿胀。因为骨组织的炎症会波及周围的软组织，导致组织充血、水肿。如果发生在关节周围，可能会影响关节的活动范围。

- 少数情况下，可能会出现病理性骨折。由于炎症长期存在，骨质遭到破坏，骨骼的强度下降，在轻微外力作用下，就可能发生骨折。

4. **诊断方面**

- 由于没有特异性的细菌感染证据，其诊断主要依靠临床表现、影像学检查和排除其他可能的疾病。影像学检查如X线、CT和MRI等可以显示骨病变的部位、范围和形态。在疾病早期，X线可能仅显示轻微的骨质改变，而MRI能够更早地发现骨髓水肿等炎症改变。同时，需要排除骨髓炎（由细菌感染引起）、骨肿瘤等疾病，这可能需要进行血液检查（如炎症指标检测、肿瘤标志物检查等）和骨活检等检查来综合判断。

2成人慢性非细菌性骨炎的典型影像学特征是什么？


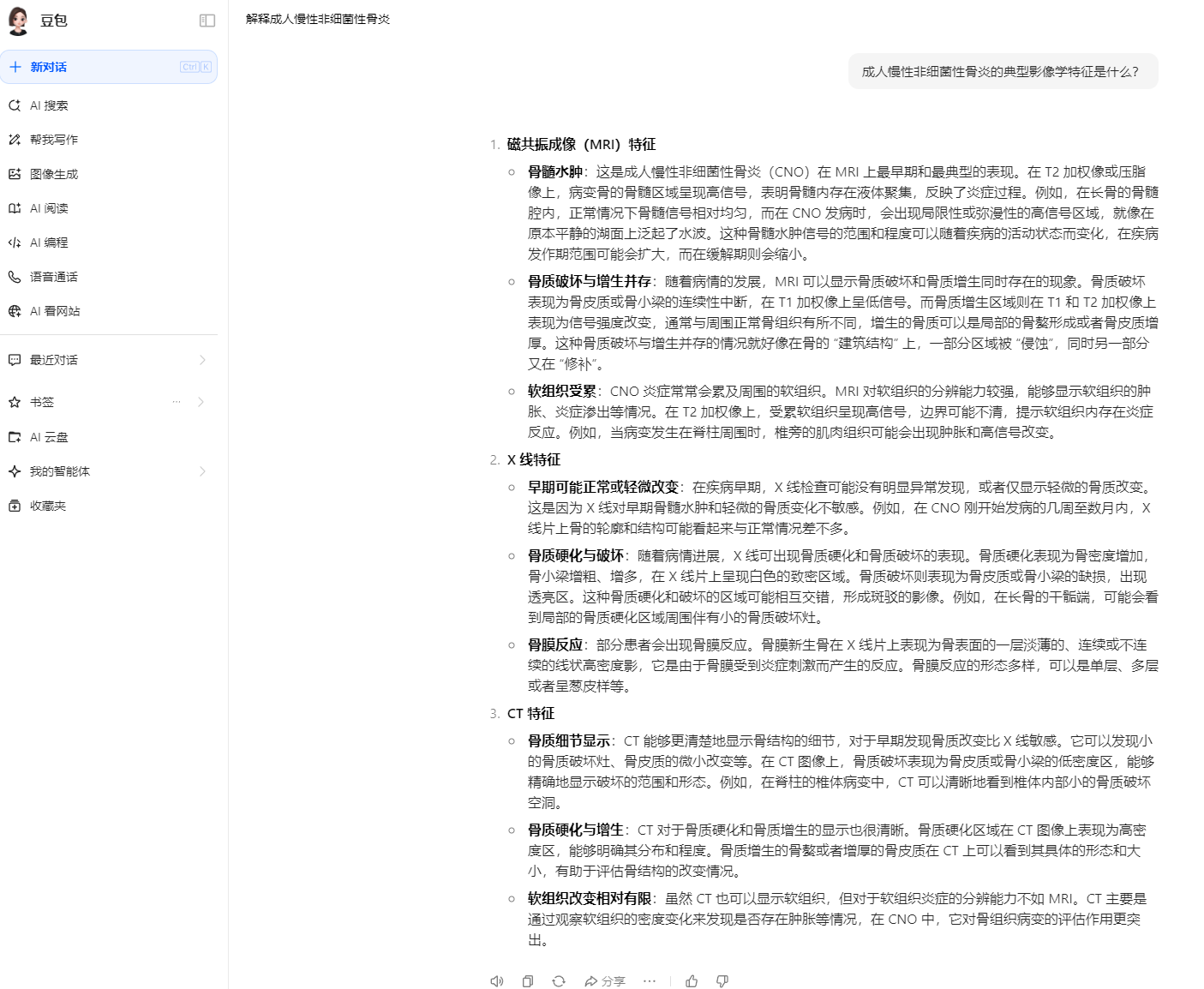
1. **磁共振成像（MRI）特征**

- **骨髓水肿**：这是成人慢性非细菌性骨炎（CNO）在MRI上最早期和最典型的表现。在T2加权像或压脂像上，病变骨的骨髓区域呈现高信号，表明骨髓内存在液体聚集，反映了炎症过程。例如，在长骨的骨髓腔内，正常情况下骨髓信号相对均匀，而在CNO发病时，会出现局限性或弥漫性的高信号区域，就像在原本平静的湖面上泛起了水波。这种骨髓水肿信号的范围和程度可以随着疾病的活动状态而变化，在疾病发作期范围可能会扩大，而在缓解期则会缩小。

- **骨质破坏与增生并存**：随着病情的发展，MRI可以显示骨质破坏和骨质增生同时存在的现象。骨质破坏表现为骨皮质或骨小梁的连续性中断，在T1加权像上呈低信号。而骨质增生区域则在T1和T2加权像上表现为信号强度改变，通常与周围正常骨组织有所不同，增生的骨质可以是局部的骨赘形成或者骨皮质增厚。这种骨质破坏与增生并存的情况就好像在骨的“建筑结构”上，一部分区域被“侵蚀”，同时另一部分又在“修补”。

- **软组织受累**：CNO炎症常常会累及周围的软组织。MRI对软组织的分辨能力较强，能够显示软组织的肿胀、炎症渗出等情况。在T2加权像上，受累软组织呈现高信号，边界可能不清，提示软组织内存在炎症反应。例如，当病变发生在脊柱周围时，椎旁的肌肉组织可能会出现肿胀和高信号改变。

2. **X线特征**

- **早期可能正常或轻微改变**：在疾病早期，X线检查可能没有明显异常发现，或者仅显示轻微的骨质改变。这是因为X线对早期骨髓水肿和轻微的骨质变化不敏感。例如，在CNO刚开始发病的几周至数月内，X线片上骨的轮廓和结构可能看起来与正常情况差不多。

- **骨质硬化与破坏**：随着病情进展，X线可出现骨质硬化和骨质破坏的表现。骨质硬化表现为骨密度增加，骨小梁增粗、增多，在X线片上呈现白色的致密区域。骨质破坏则表现为骨皮质或骨小梁的缺损，出现透亮区。这种骨质硬化和破坏的区域可能相互交错，形成斑驳的影像。例如，在长骨的干骺端，可能会看到局部的骨质硬化区域周围伴有小的骨质破坏灶。

- **骨膜反应**：部分患者会出现骨膜反应。骨膜新生骨在X线片上表现为骨表面的一层淡薄的、连续或不连续的线状高密度影，它是由于骨膜受到炎症刺激而产生的反应。骨膜反应的形态多样，可以是单层、多层或者呈葱皮样等。

3. **CT特征**

- **骨质细节显示**：CT能够更清楚地显示骨结构的细节，对于早期发现骨质改变比X线敏感。它可以发现小的骨质破坏灶、骨皮质的微小改变等。在CT图像上，骨质破坏表现为骨皮质或骨小梁的低密度区，能够精确地显示破坏的范围和形态。例如，在脊柱的椎体病变中，CT可以清晰地看到椎体内部小的骨质破坏空洞。

- **骨质硬化与增生**：CT对于骨质硬化和骨质增生的显示也很清晰。骨质硬化区域在CT图像上表现为高密度区，能够明确其分布和程度。骨质增生的骨赘或者增厚的骨皮质在CT上可以看到其具体的形态和大小，有助于评估骨结构的改变情况。

- **软组织改变相对有限**：虽然CT也可以显示软组织，但对于软组织炎症的分辨能力不如MRI。CT主要是通过观察软组织的密度变化来发现是否存在肿胀等情况，在CNO中，它对骨组织病变的评估作用更突出。

3成人慢性非细菌性骨炎最常见的表现是什么？


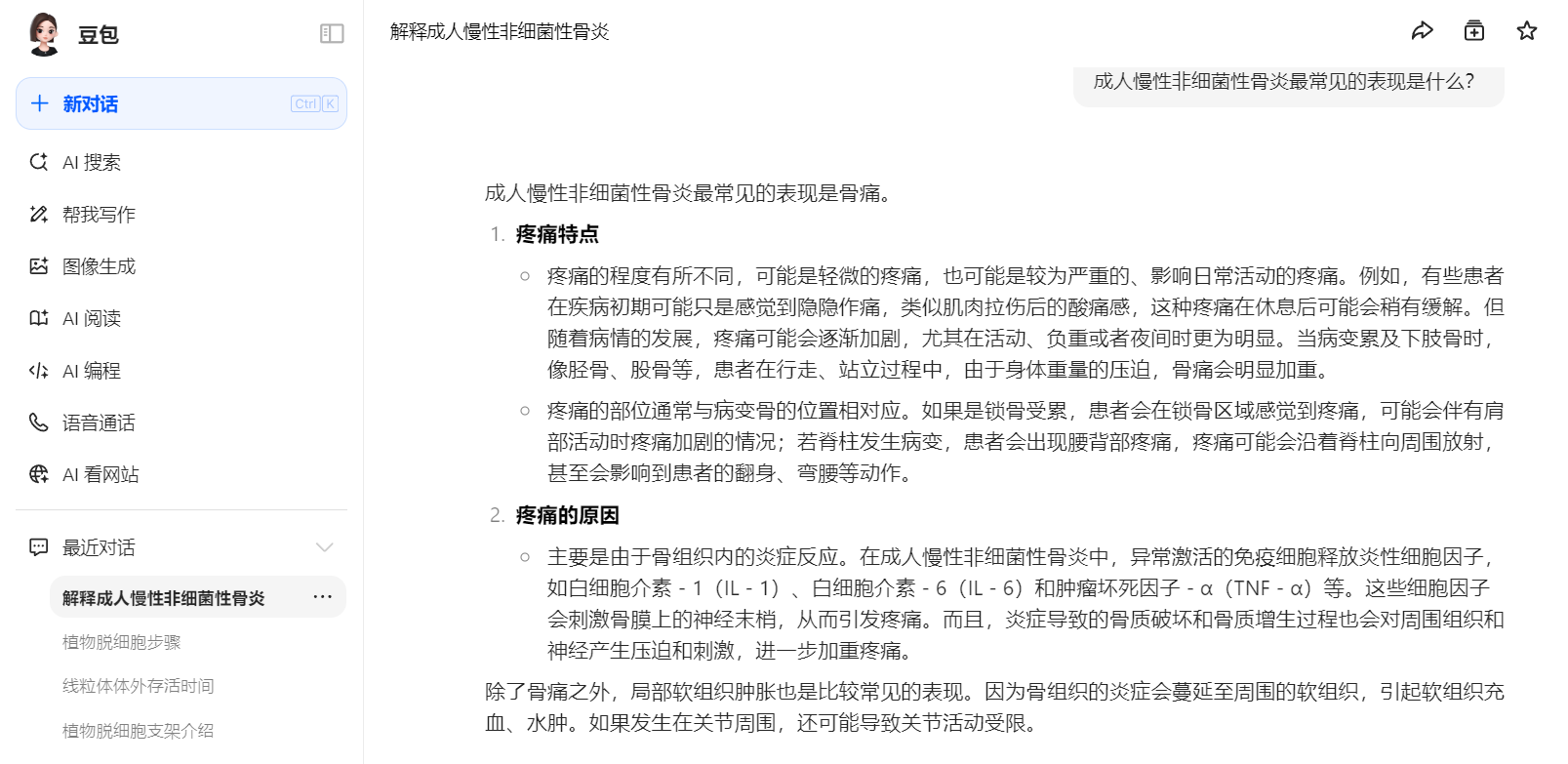
成人慢性非细菌性骨炎最常见的表现是骨痛。

1. **疼痛特点**

- 疼痛的程度有所不同，可能是轻微的疼痛，也可能是较为严重的、影响日常活动的疼痛。例如，有些患者在疾病初期可能只是感觉到隐隐作痛，类似肌肉拉伤后的酸痛感，这种疼痛在休息后可能会稍有缓解。但随着病情的发展，疼痛可能会逐渐加剧，尤其在活动、负重或者夜间时更为明显。当病变累及下肢骨时，像胫骨、股骨等，患者在行走、站立过程中，由于身体重量的压迫，骨痛会明显加重。

- 疼痛的部位通常与病变骨的位置相对应。如果是锁骨受累，患者会在锁骨区域感觉到疼痛，可能会伴有肩部活动时疼痛加剧的情况；若脊柱发生病变，患者会出现腰背部疼痛，疼痛可能会沿着脊柱向周围放射，甚至会影响到患者的翻身、弯腰等动作。

2. **疼痛的原因**

- 主要是由于骨组织内的炎症反应。在成人慢性非细菌性骨炎中，异常激活的免疫细胞释放炎性细胞因子，如白细胞介素 - 1（IL - 1）、白细胞介素 - 6（IL - 6）和肿瘤坏死因子 - α（TNF - α）等。这些细胞因子会刺激骨膜上的神经末梢，从而引发疼痛。而且，炎症导致的骨质破坏和骨质增生过程也会对周围组织和神经产生压迫和刺激，进一步加重疼痛。

除了骨痛之外，局部软组织肿胀也是比较常见的表现。因为骨组织的炎症会蔓延至周围的软组织，引起软组织充血、水肿。如果发生在关节周围，还可能导致关节活动受限。

4成人慢性非细菌性骨炎最常受累的部位是那些？


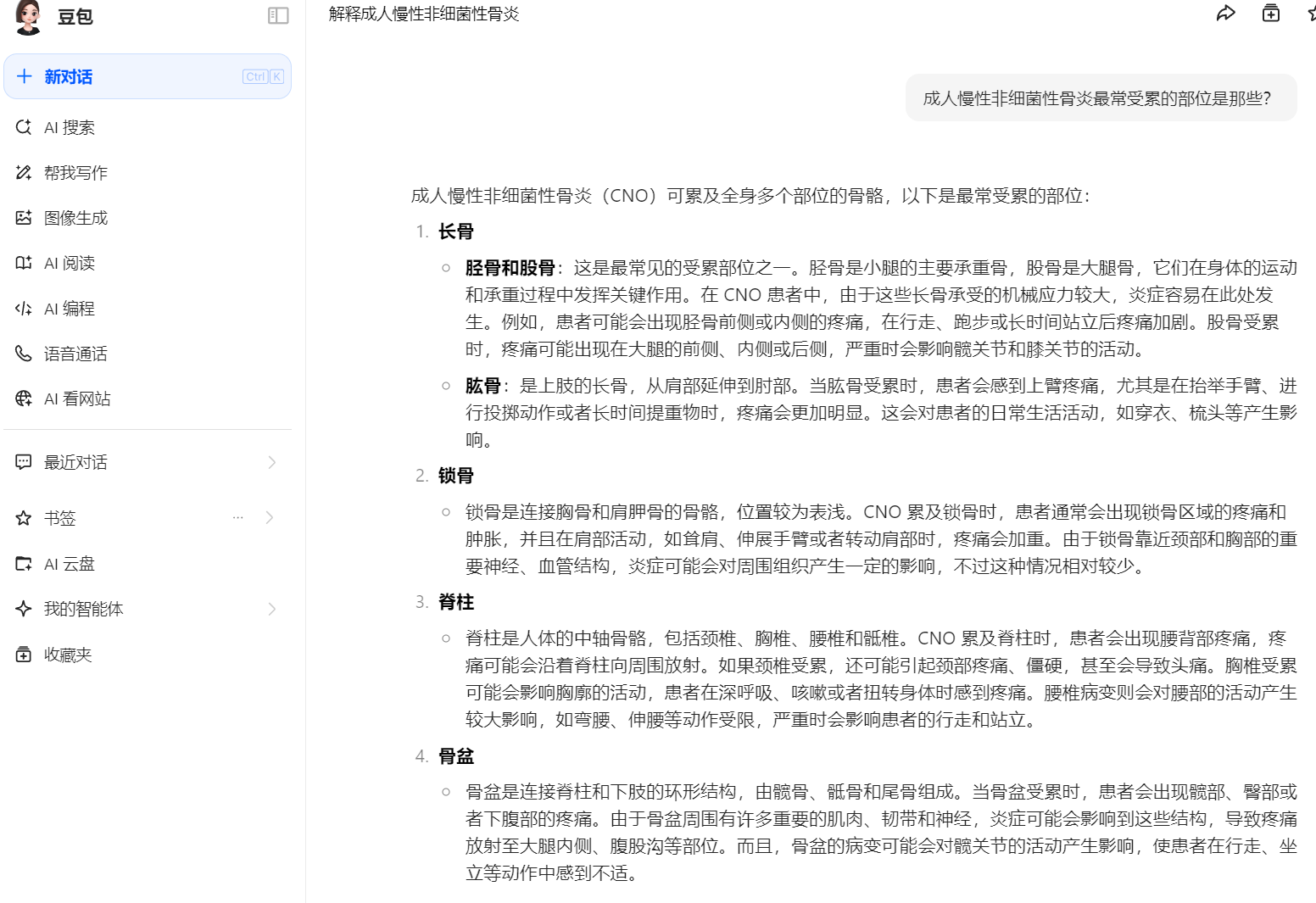
成人慢性非细菌性骨炎（CNO）可累及全身多个部位的骨骼，以下是最常受累的部位：

1. **长骨**

- **胫骨和股骨**：这是最常见的受累部位之一。胫骨是小腿的主要承重骨，股骨是大腿骨，它们在身体的运动和承重过程中发挥关键作用。在CNO患者中，由于这些长骨承受的机械应力较大，炎症容易在此处发生。例如，患者可能会出现胫骨前侧或内侧的疼痛，在行走、跑步或长时间站立后疼痛加剧。股骨受累时，疼痛可能出现在大腿的前侧、内侧或后侧，严重时会影响髋关节和膝关节的活动。

- **肱骨**：是上肢的长骨，从肩部延伸到肘部。当肱骨受累时，患者会感到上臂疼痛，尤其是在抬举手臂、进行投掷动作或者长时间提重物时，疼痛会更加明显。这会对患者的日常生活活动，如穿衣、梳头等产生影响。

2. **锁骨**

- 锁骨是连接胸骨和肩胛骨的骨骼，位置较为表浅。CNO累及锁骨时，患者通常会出现锁骨区域的疼痛和肿胀，并且在肩部活动，如耸肩、伸展手臂或者转动肩部时，疼痛会加重。由于锁骨靠近颈部和胸部的重要神经、血管结构，炎症可能会对周围组织产生一定的影响，不过这种情况相对较少。

3. **脊柱**

- 脊柱是人体的中轴骨骼，包括颈椎、胸椎、腰椎和骶椎。CNO累及脊柱时，患者会出现腰背部疼痛，疼痛可能会沿着脊柱向周围放射。如果颈椎受累，还可能引起颈部疼痛、僵硬，甚至会导致头痛。胸椎受累可能会影响胸廓的活动，患者在深呼吸、咳嗽或者扭转身体时感到疼痛。腰椎病变则会对腰部的活动产生较大影响，如弯腰、伸腰等动作受限，严重时会影响患者的行走和站立。

4. **骨盆**

- 骨盆是连接脊柱和下肢的环形结构，由髋骨、骶骨和尾骨组成。当骨盆受累时，患者会出现髋部、臀部或者下腹部的疼痛。由于骨盆周围有许多重要的肌肉、韧带和神经，炎症可能会影响到这些结构，导致疼痛放射至大腿内侧、腹股沟等部位。而且，骨盆的病变可能会对髋关节的活动产生影响，使患者在行走、坐立等动作中感到不适。

5成人慢性非细菌性骨炎有没有特异性的炎症标志物？


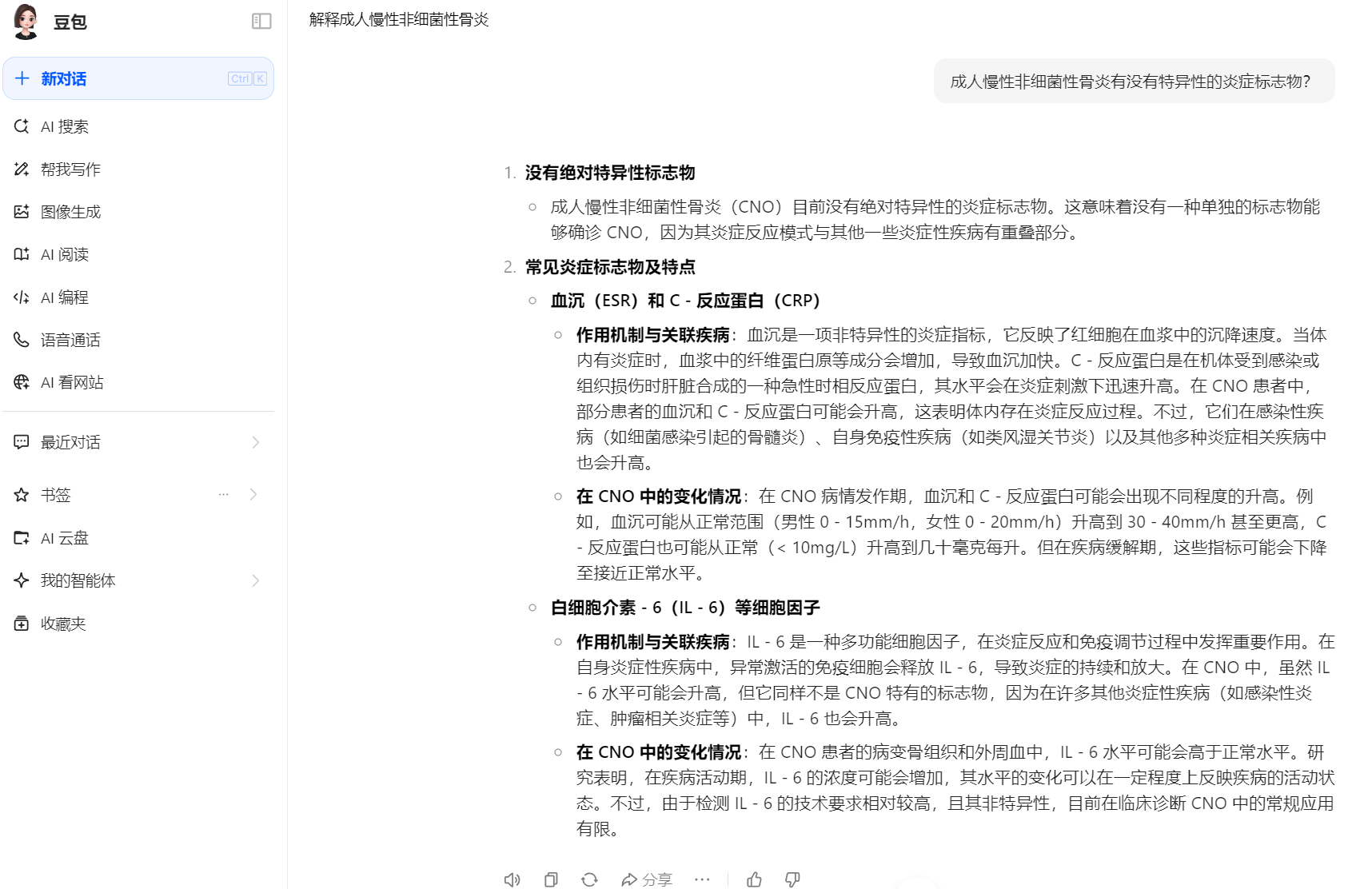
1. **没有绝对特异性标志物**

- 成人慢性非细菌性骨炎（CNO）目前没有绝对特异性的炎症标志物。这意味着没有一种单独的标志物能够确诊CNO，因为其炎症反应模式与其他一些炎症性疾病有重叠部分。

2. **常见炎症标志物及特点**

- **血沉（ESR）和C -反应蛋白（CRP）**

- **作用机制与关联疾病**：血沉是一项非特异性的炎症指标，它反映了红细胞在血浆中的沉降速度。当体内有炎症时，血浆中的纤维蛋白原等成分会增加，导致血沉加快。C -反应蛋白是在机体受到感染或组织损伤时肝脏合成的一种急性时相反应蛋白，其水平会在炎症刺激下迅速升高。在CNO患者中，部分患者的血沉和C -反应蛋白可能会升高，这表明体内存在炎症反应过程。不过，它们在感染性疾病（如细菌感染引起的骨髓炎）、自身免疫性疾病（如类风湿关节炎）以及其他多种炎症相关疾病中也会升高。

- **在CNO中的变化情况**：在CNO病情发作期，血沉和C -反应蛋白可能会出现不同程度的升高。例如，血沉可能从正常范围（男性0 - 15mm/h，女性0 - 20mm/h）升高到30 - 40mm/h甚至更高，C -反应蛋白也可能从正常（< 10mg/L）升高到几十毫克每升。但在疾病缓解期，这些指标可能会下降至接近正常水平。

- **白细胞介素 - 6（IL - 6）等细胞因子**

- **作用机制与关联疾病**：IL - 6是一种多功能细胞因子，在炎症反应和免疫调节过程中发挥重要作用。在自身炎症性疾病中，异常激活的免疫细胞会释放IL - 6，导致炎症的持续和放大。在CNO中，虽然IL - 6水平可能会升高，但它同样不是CNO特有的标志物，因为在许多其他炎症性疾病（如感染性炎症、肿瘤相关炎症等）中，IL - 6也会升高。

- **在CNO中的变化情况**：在CNO患者的病变骨组织和外周血中，IL - 6水平可能会高于正常水平。研究表明，在疾病活动期，IL - 6的浓度可能会增加，其水平的变化可以在一定程度上反映疾病的活动状态。不过，由于检测IL - 6的技术要求相对较高，且其非特异性，目前在临床诊断CNO中的常规应用有限。

6慢性非细菌性骨炎首选的影像学检查是什么？


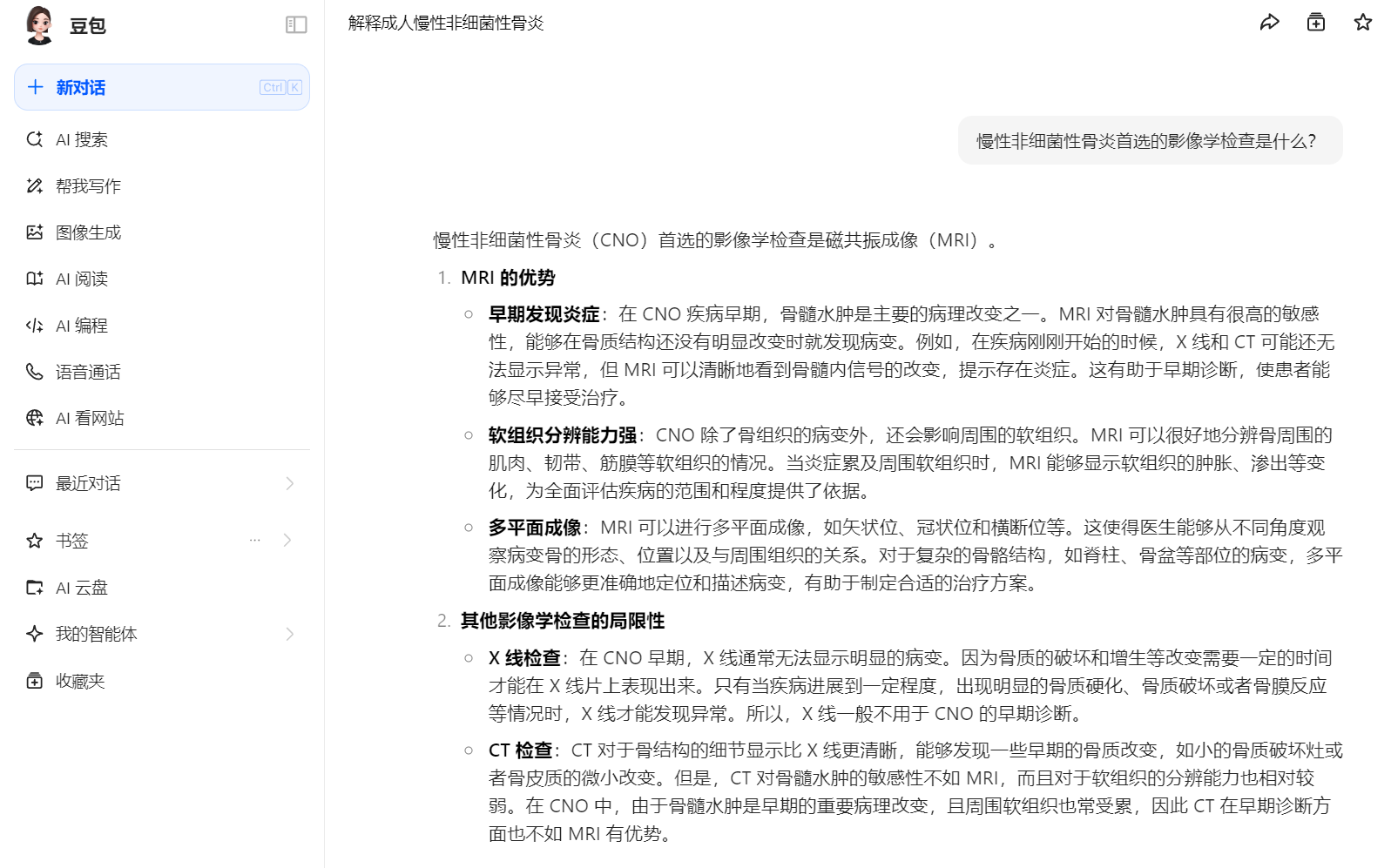
 慢性非细菌性骨炎（CNO）首选的影像学检查是磁共振成像（MRI）。

1. **MRI的优势**

- **早期发现炎症**：在CNO疾病早期，骨髓水肿是主要的病理改变之一。MRI对骨髓水肿具有很高的敏感性，能够在骨质结构还没有明显改变时就发现病变。例如，在疾病刚刚开始的时候，X线和CT可能还无法显示异常，但MRI可以清晰地看到骨髓内信号的改变，提示存在炎症。这有助于早期诊断，使患者能够尽早接受治疗。

- **软组织分辨能力强**：CNO除了骨组织的病变外，还会影响周围的软组织。MRI可以很好地分辨骨周围的肌肉、韧带、筋膜等软组织的情况。当炎症累及周围软组织时，MRI能够显示软组织的肿胀、渗出等变化，为全面评估疾病的范围和程度提供了依据。

- **多平面成像**：MRI可以进行多平面成像，如矢状位、冠状位和横断位等。这使得医生能够从不同角度观察病变骨的形态、位置以及与周围组织的关系。对于复杂的骨骼结构，如脊柱、骨盆等部位的病变，多平面成像能够更准确地定位和描述病变，有助于制定合适的治疗方案。

2. **其他影像学检查的局限性**

- **X线检查**：在CNO早期，X线通常无法显示明显的病变。因为骨质的破坏和增生等改变需要一定的时间才能在X线片上表现出来。只有当疾病进展到一定程度，出现明显的骨质硬化、骨质破坏或者骨膜反应等情况时，X线才能发现异常。所以，X线一般不用于CNO的早期诊断。

- **CT检查**：CT对于骨结构的细节显示比X线更清晰，能够发现一些早期的骨质改变，如小的骨质破坏灶或者骨皮质的微小改变。但是，CT对骨髓水肿的敏感性不如MRI，而且对于软组织的分辨能力也相对较弱。在CNO中，由于骨髓水肿是早期的重要病理改变，且周围软组织也常受累，因此CT在早期诊断方面也不如MRI有优势。

7全身成像检查是不是诊断成人慢性非细菌性骨炎的先决条件？


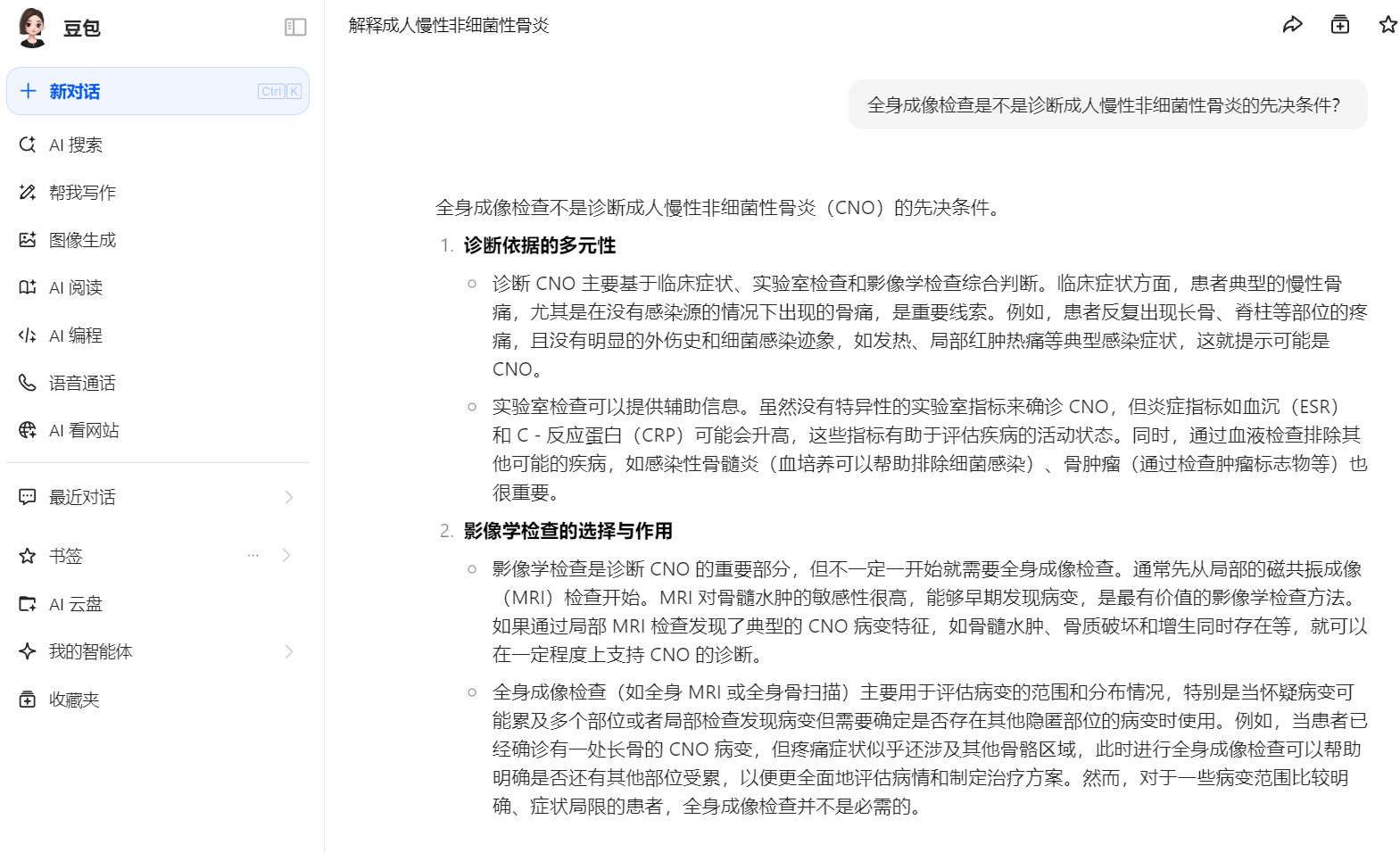
全身成像检查不是诊断成人慢性非细菌性骨炎（CNO）的先决条件。

1. **诊断依据的多元性**

- 诊断CNO主要基于临床症状、实验室检查和影像学检查综合判断。临床症状方面，患者典型的慢性骨痛，尤其是在没有感染源的情况下出现的骨痛，是重要线索。例如，患者反复出现长骨、脊柱等部位的疼痛，且没有明显的外伤史和细菌感染迹象，如发热、局部红肿热痛等典型感染症状，这就提示可能是CNO。

- 实验室检查可以提供辅助信息。虽然没有特异性的实验室指标来确诊CNO，但炎症指标如血沉（ESR）和C - 反应蛋白（CRP）可能会升高，这些指标有助于评估疾病的活动状态。同时，通过血液检查排除其他可能的疾病，如感染性骨髓炎（血培养可以帮助排除细菌感染）、骨肿瘤（通过检查肿瘤标志物等）也很重要。

2. **影像学检查的选择与作用**

- 影像学检查是诊断CNO的重要部分，但不一定一开始就需要全身成像检查。通常先从局部的磁共振成像（MRI）检查开始。MRI对骨髓水肿的敏感性很高，能够早期发现病变，是最有价值的影像学检查方法。如果通过局部MRI检查发现了典型的CNO病变特征，如骨髓水肿、骨质破坏和增生同时存在等，就可以在一定程度上支持CNO的诊断。

- 全身成像检查（如全身MRI或全身骨扫描）主要用于评估病变的范围和分布情况，特别是当怀疑病变可能累及多个部位或者局部检查发现病变但需要确定是否存在其他隐匿部位的病变时使用。例如，当患者已经确诊有一处长骨的CNO病变，但疼痛症状似乎还涉及其他骨骼区域，此时进行全身成像检查可以帮助明确是否还有其他部位受累，以便更全面地评估病情和制定治疗方案。然而，对于一些病变范围比较明确、症状局限的患者，全身成像检查并不是必需的。

8诊断成人慢性非细菌性骨炎有必要常规骨活检吗？


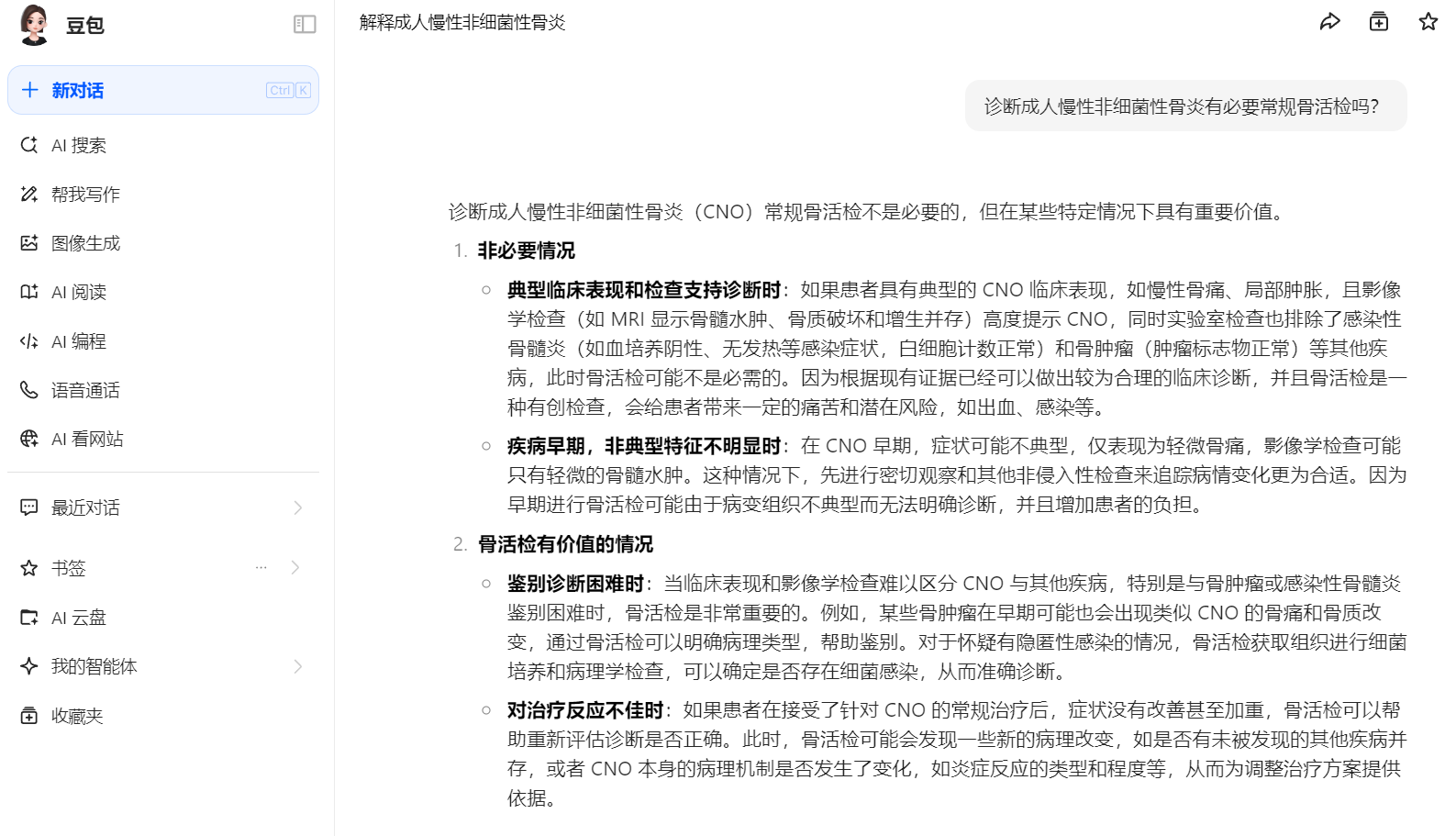
 诊断成人慢性非细菌性骨炎（CNO）常规骨活检不是必要的，但在某些特定情况下具有重要价值。

1. **非必要情况**

- **典型临床表现和检查支持诊断时**：如果患者具有典型的CNO临床表现，如慢性骨痛、局部肿胀，且影像学检查（如MRI显示骨髓水肿、骨质破坏和增生并存）高度提示CNO，同时实验室检查也排除了感染性骨髓炎（如血培养阴性、无发热等感染症状，白细胞计数正常）和骨肿瘤（肿瘤标志物正常）等其他疾病，此时骨活检可能不是必需的。因为根据现有证据已经可以做出较为合理的临床诊断，并且骨活检是一种有创检查，会给患者带来一定的痛苦和潜在风险，如出血、感染等。

- **疾病早期，非典型特征不明显时**：在CNO早期，症状可能不典型，仅表现为轻微骨痛，影像学检查可能只有轻微的骨髓水肿。这种情况下，先进行密切观察和其他非侵入性检查来追踪病情变化更为合适。因为早期进行骨活检可能由于病变组织不典型而无法明确诊断，并且增加患者的负担。

2. **骨活检有价值的情况**

- **鉴别诊断困难时**：当临床表现和影像学检查难以区分CNO与其他疾病，特别是与骨肿瘤或感染性骨髓炎鉴别困难时，骨活检是非常重要的。例如，某些骨肿瘤在早期可能也会出现类似CNO的骨痛和骨质改变，通过骨活检可以明确病理类型，帮助鉴别。对于怀疑有隐匿性感染的情况，骨活检获取组织进行细菌培养和病理学检查，可以确定是否存在细菌感染，从而准确诊断。

- **对治疗反应不佳时**：如果患者在接受了针对CNO的常规治疗后，症状没有改善甚至加重，骨活检可以帮助重新评估诊断是否正确。此时，骨活检可能会发现一些新的病理改变，如是否有未被发现的其他疾病并存，或者CNO本身的病理机制是否发生了变化，如炎症反应的类型和程度等，从而为调整治疗方案提供依据。

9慢性非细菌性骨炎如何与恶性骨肿瘤鉴别？


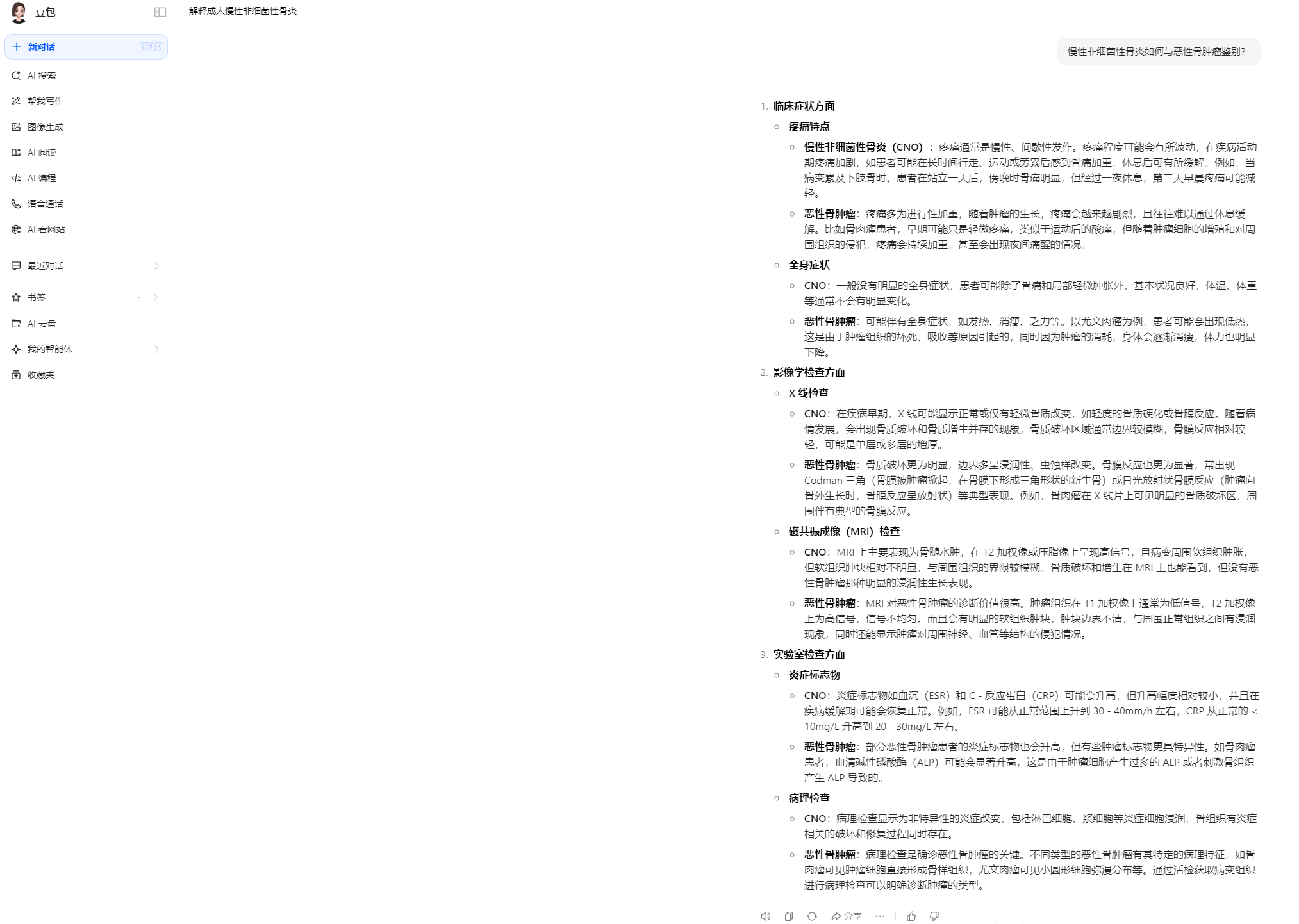
 1. **临床症状方面**

- **疼痛特点**

- **慢性非细菌性骨炎（CNO）**：疼痛通常是慢性、间歇性发作。疼痛程度可能会有所波动，在疾病活动期疼痛加剧，如患者可能在长时间行走、运动或劳累后感到骨痛加重，休息后可有所缓解。例如，当病变累及下肢骨时，患者在站立一天后，傍晚时骨痛明显，但经过一夜休息，第二天早晨疼痛可能减轻。

- **恶性骨肿瘤**：疼痛多为进行性加重，随着肿瘤的生长，疼痛会越来越剧烈，且往往难以通过休息缓解。比如骨肉瘤患者，早期可能只是轻微疼痛，类似于运动后的酸痛，但随着肿瘤细胞的增殖和对周围组织的侵犯，疼痛会持续加重，甚至会出现夜间痛醒的情况。

- **全身症状**

- **CNO**：一般没有明显的全身症状，患者可能除了骨痛和局部轻微肿胀外，基本状况良好，体温、体重等通常不会有明显变化。

- **恶性骨肿瘤**：可能伴有全身症状，如发热、消瘦、乏力等。以尤文肉瘤为例，患者可能会出现低热，这是由于肿瘤组织的坏死、吸收等原因引起的，同时因为肿瘤的消耗，身体会逐渐消瘦，体力也明显下降。

2. **影像学检查方面**

- **X线检查**

- **CNO**：在疾病早期，X线可能显示正常或仅有轻微骨质改变，如轻度的骨质硬化或骨膜反应。随着病情发展，会出现骨质破坏和骨质增生并存的现象，骨质破坏区域通常边界较模糊，骨膜反应相对较轻，可能是单层或多层的增厚。

- **恶性骨肿瘤**：骨质破坏更为明显，边界多呈浸润性、虫蚀样改变。骨膜反应也更为显著，常出现Codman三角（骨膜被肿瘤掀起，在骨膜下形成三角形状的新生骨）或日光放射状骨膜反应（肿瘤向骨外生长时，骨膜反应呈放射状）等典型表现。例如，骨肉瘤在X线片上可见明显的骨质破坏区，周围伴有典型的骨膜反应。

- **磁共振成像（MRI）检查**

- **CNO**：MRI上主要表现为骨髓水肿，在T2加权像或压脂像上呈现高信号，且病变周围软组织肿胀，但软组织肿块相对不明显，与周围组织的界限较模糊。骨质破坏和增生在MRI上也能看到，但没有恶性骨肿瘤那种明显的浸润性生长表现。

- **恶性骨肿瘤**：MRI对恶性骨肿瘤的诊断价值很高。肿瘤组织在T1加权像上通常为低信号，T2加权像上为高信号，信号不均匀。而且会有明显的软组织肿块，肿块边界不清，与周围正常组织之间有浸润现象，同时还能显示肿瘤对周围神经、血管等结构的侵犯情况。

3. **实验室检查方面**

- **炎症标志物**

- **CNO**：炎症标志物如血沉（ESR）和C -反应蛋白（CRP）可能会升高，但升高幅度相对较小，并且在疾病缓解期可能会恢复正常。例如，ESR可能从正常范围上升到30 - 40mm/h左右，CRP从正常的< 10mg/L升高到20 - 30mg/L左右。

- **恶性骨肿瘤**：部分恶性骨肿瘤患者的炎症标志物也会升高，但有些肿瘤标志物更具特异性。如骨肉瘤患者，血清碱性磷酸酶（ALP）可能会显著升高，这是由于肿瘤细胞产生过多的ALP或者刺激骨组织产生ALP导致的。

- **病理检查**

- **CNO**：病理检查显示为非特异性的炎症改变，包括淋巴细胞、浆细胞等炎症细胞浸润，骨组织有炎症相关的破坏和修复过程同时存在。

- **恶性骨肿瘤**：病理检查是确诊恶性骨肿瘤的关键。不同类型的恶性骨肿瘤有其特定的病理特征，如骨肉瘤可见肿瘤细胞直接形成骨样组织，尤文肉瘤可见小圆形细胞弥漫分布等。通过活检获取病变组织进行病理检查可以明确诊断肿瘤的类型。

10慢性非细菌性骨炎如何与骨关节炎鉴别？


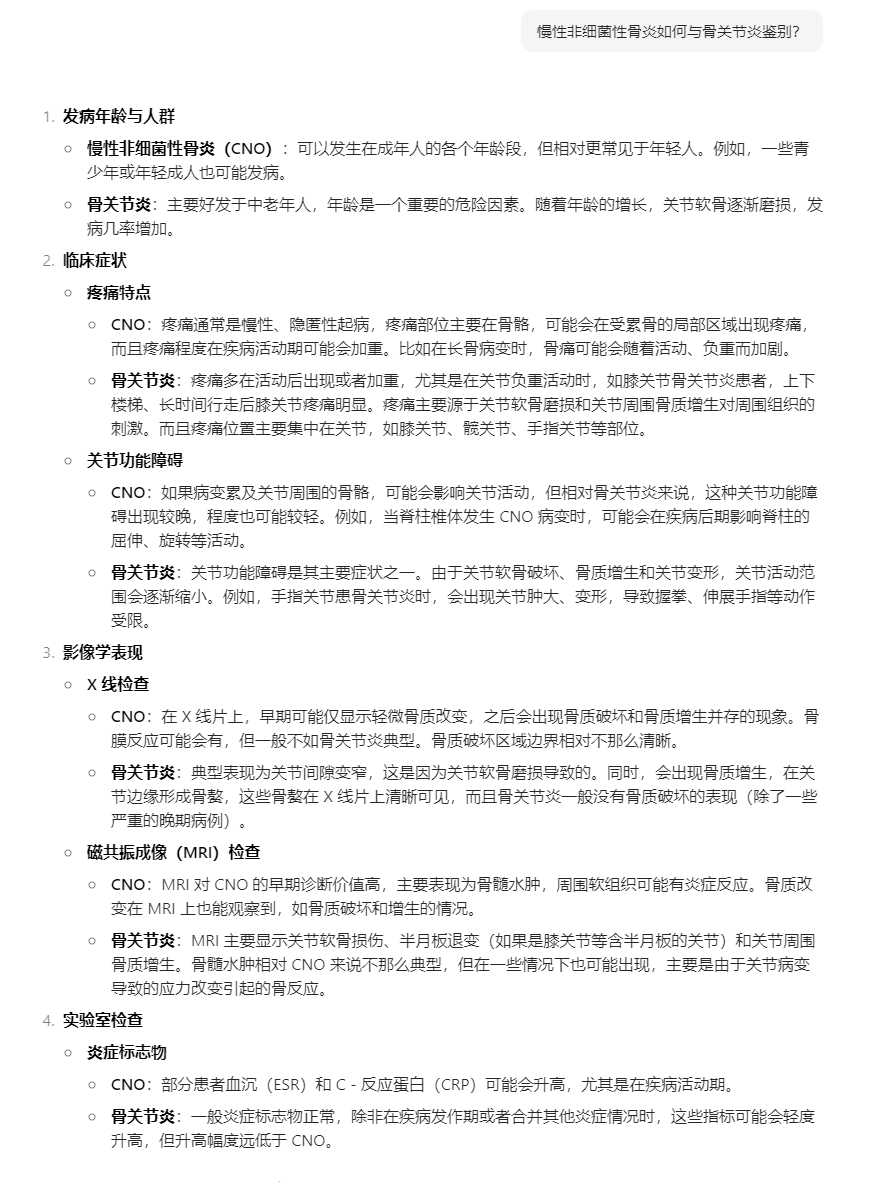
 1. **发病年龄与人群**

- **慢性非细菌性骨炎（CNO）**：可以发生在成年人的各个年龄段，但相对更常见于年轻人。例如，一些青少年或年轻成人也可能发病。

- **骨关节炎**：主要好发于中老年人，年龄是一个重要的危险因素。随着年龄的增长，关节软骨逐渐磨损，发病几率增加。

2. **临床症状**

- **疼痛特点**

- **CNO**：疼痛通常是慢性、隐匿性起病，疼痛部位主要在骨骼，可能会在受累骨的局部区域出现疼痛，而且疼痛程度在疾病活动期可能会加重。比如在长骨病变时，骨痛可能会随着活动、负重而加剧。

- **骨关节炎**：疼痛多在活动后出现或者加重，尤其是在关节负重活动时，如膝关节骨关节炎患者，上下楼梯、长时间行走后膝关节疼痛明显。疼痛主要源于关节软骨磨损和关节周围骨质增生对周围组织的刺激。而且疼痛位置主要集中在关节，如膝关节、髋关节、手指关节等部位。

- **关节功能障碍**

- **CNO**：如果病变累及关节周围的骨骼，可能会影响关节活动，但相对骨关节炎来说，这种关节功能障碍出现较晚，程度也可能较轻。例如，当脊柱椎体发生CNO病变时，可能会在疾病后期影响脊柱的屈伸、旋转等活动。

- **骨关节炎**：关节功能障碍是其主要症状之一。由于关节软骨破坏、骨质增生和关节变形，关节活动范围会逐渐缩小。例如，手指关节患骨关节炎时，会出现关节肿大、变形，导致握拳、伸展手指等动作受限。

3. **影像学表现**

- **X线检查**

- **CNO**：在X线片上，早期可能仅显示轻微骨质改变，之后会出现骨质破坏和骨质增生并存的现象。骨膜反应可能会有，但一般不如骨关节炎典型。骨质破坏区域边界相对不那么清晰。

- **骨关节炎**：典型表现为关节间隙变窄，这是因为关节软骨磨损导致的。同时，会出现骨质增生，在关节边缘形成骨赘，这些骨赘在X线片上清晰可见，而且骨关节炎一般没有骨质破坏的表现（除了一些严重的晚期病例）。

- **磁共振成像（MRI）检查**

- **CNO**：MRI对CNO的早期诊断价值高，主要表现为骨髓水肿，周围软组织可能有炎症反应。骨质改变在MRI上也能观察到，如骨质破坏和增生的情况。

- **骨关节炎**：MRI主要显示关节软骨损伤、半月板退变（如果是膝关节等含半月板的关节）和关节周围骨质增生。骨髓水肿相对CNO来说不那么典型，但在一些情况下也可能出现，主要是由于关节病变导致的应力改变引起的骨反应。

4. **实验室检查**

- **炎症标志物**

- **CNO**：部分患者血沉（ESR）和C -反应蛋白（CRP）可能会升高，尤其是在疾病活动期。

- **骨关节炎**：一般炎症标志物正常，除非在疾病发作期或者合并其他炎症情况时，这些指标可能会轻度升高，但升高幅度远低于CNO。

11慢性非细菌性骨炎如何与纤维发育不良鉴别？


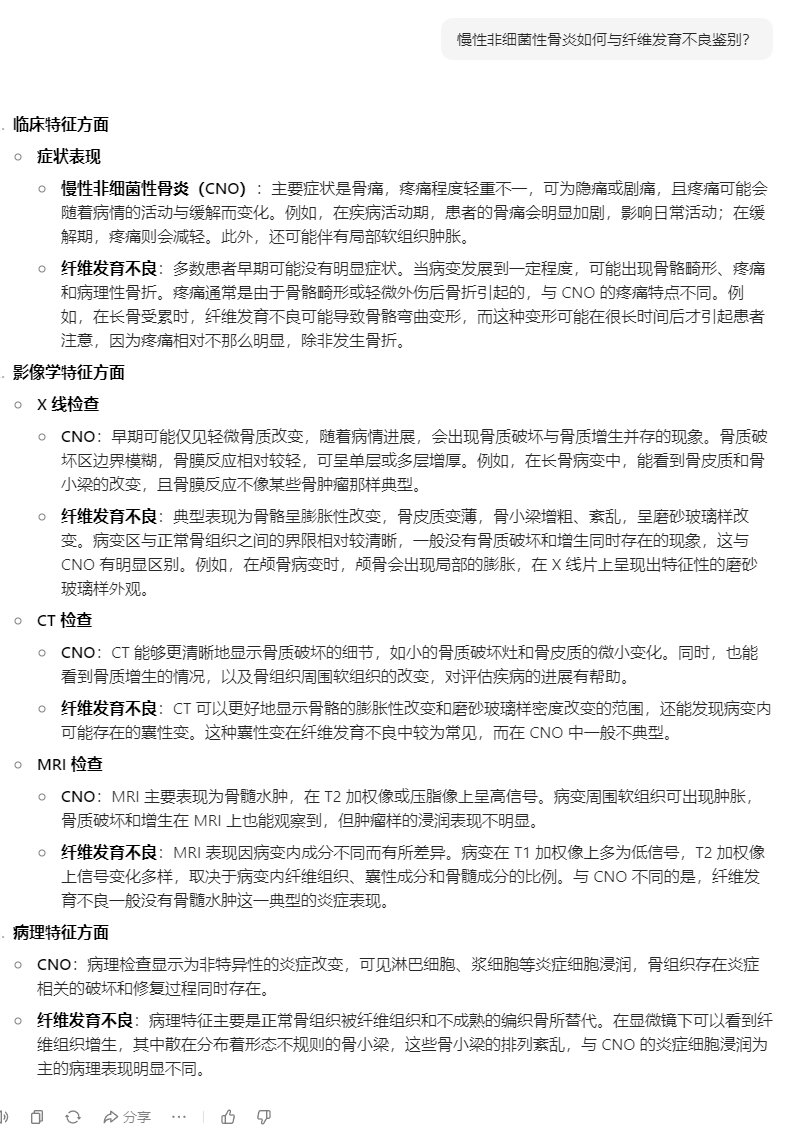
1. **临床特征方面**

- **症状表现**

- **慢性非细菌性骨炎（CNO）**：主要症状是骨痛，疼痛程度轻重不一，可为隐痛或剧痛，且疼痛可能会随着病情的活动与缓解而变化。例如，在疾病活动期，患者的骨痛会明显加剧，影响日常活动；在缓解期，疼痛则会减轻。此外，还可能伴有局部软组织肿胀。

- **纤维发育不良**：多数患者早期可能没有明显症状。当病变发展到一定程度，可能出现骨骼畸形、疼痛和病理性骨折。疼痛通常是由于骨骼畸形或轻微外伤后骨折引起的，与CNO的疼痛特点不同。例如，在长骨受累时，纤维发育不良可能导致骨骼弯曲变形，而这种变形可能在很长时间后才引起患者注意，因为疼痛相对不那么明显，除非发生骨折。

2. **影像学特征方面**

- **X线检查**

- **CNO**：早期可能仅见轻微骨质改变，随着病情进展，会出现骨质破坏与骨质增生并存的现象。骨质破坏区边界模糊，骨膜反应相对较轻，可呈单层或多层增厚。例如，在长骨病变中，能看到骨皮质和骨小梁的改变，且骨膜反应不像某些骨肿瘤那样典型。

- **纤维发育不良**：典型表现为骨骼呈膨胀性改变，骨皮质变薄，骨小梁增粗、紊乱，呈磨砂玻璃样改变。病变区与正常骨组织之间的界限相对较清晰，一般没有骨质破坏和增生同时存在的现象，这与CNO有明显区别。例如，在颅骨病变时，颅骨会出现局部的膨胀，在X线片上呈现出特征性的磨砂玻璃样外观。

- **CT检查**

- **CNO**：CT能够更清晰地显示骨质破坏的细节，如小的骨质破坏灶和骨皮质的微小变化。同时，也能看到骨质增生的情况，以及骨组织周围软组织的改变，对评估疾病的进展有帮助。

- **纤维发育不良**：CT可以更好地显示骨骼的膨胀性改变和磨砂玻璃样密度改变的范围，还能发现病变内可能存在的囊性变。这种囊性变在纤维发育不良中较为常见，而在CNO中一般不典型。

- **MRI检查**

- **CNO**：MRI主要表现为骨髓水肿，在T2加权像或压脂像上呈高信号。病变周围软组织可出现肿胀，骨质破坏和增生在MRI上也能观察到，但肿瘤样的浸润表现不明显。

- **纤维发育不良**：MRI表现因病变内成分不同而有所差异。病变在T1加权像上多为低信号，T2加权像上信号变化多样，取决于病变内纤维组织、囊性成分和骨髓成分的比例。与CNO不同的是，纤维发育不良一般没有骨髓水肿这一典型的炎症表现。

3. **病理特征方面**

- **CNO**：病理检查显示为非特异性的炎症改变，可见淋巴细胞、浆细胞等炎症细胞浸润，骨组织存在炎症相关的破坏和修复过程同时存在。

- **纤维发育不良**：病理特征主要是正常骨组织被纤维组织和不成熟的编织骨所替代。在显微镜下可以看到纤维组织增生，其中散在分布着形态不规则的骨小梁，这些骨小梁的排列紊乱，与CNO的炎症细胞浸润为主的病理表现明显不同。

12在治慢性非细菌性骨炎过程中应该选择短效糖皮质激素还是长效糖皮质激素？


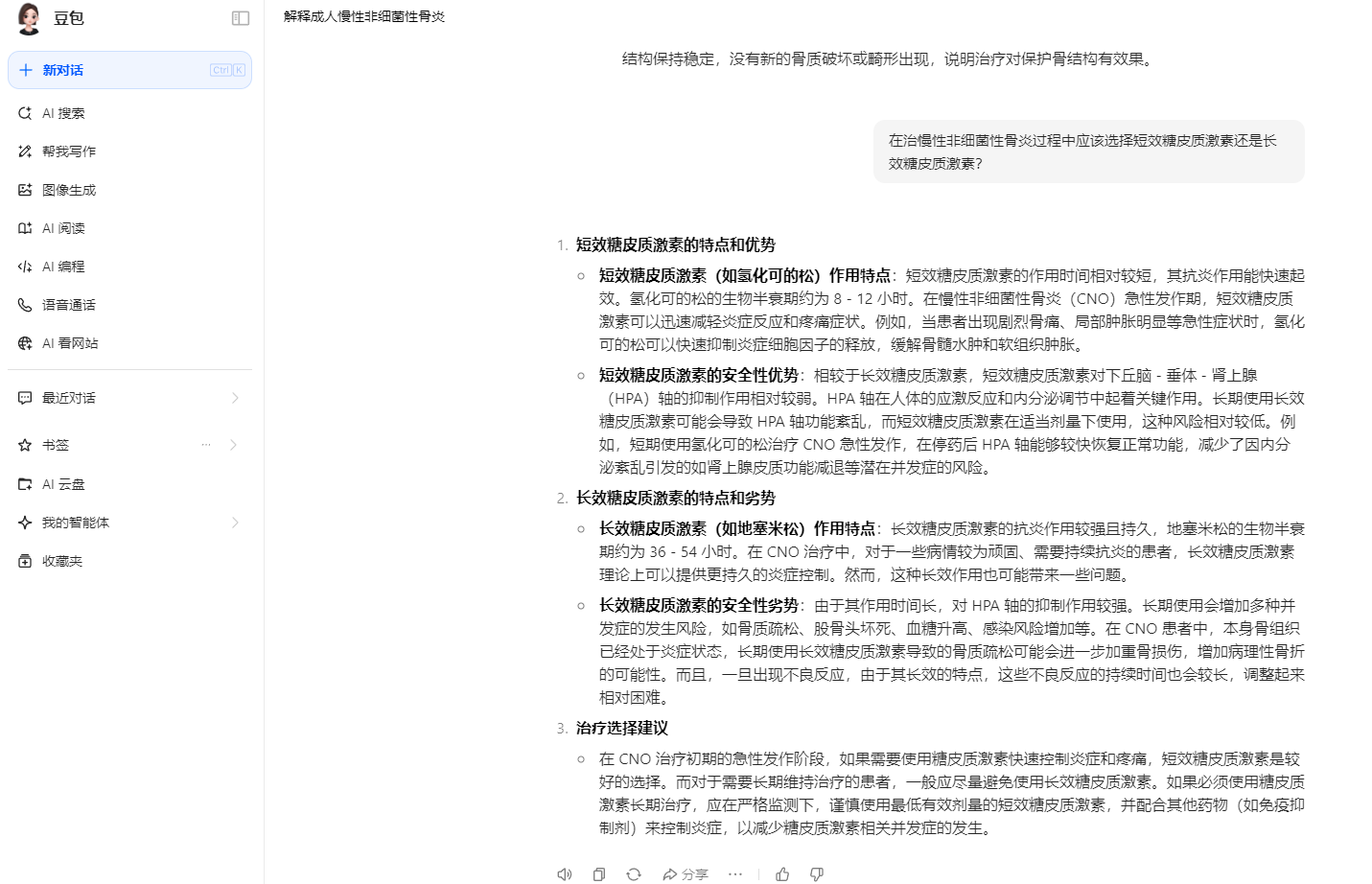
1. **短效糖皮质激素的特点和优势**

- **短效糖皮质激素（如氢化可的松）作用特点**：短效糖皮质激素的作用时间相对较短，其抗炎作用能快速起效。氢化可的松的生物半衰期约为8 - 12小时。在慢性非细菌性骨炎（CNO）急性发作期，短效糖皮质激素可以迅速减轻炎症反应和疼痛症状。例如，当患者出现剧烈骨痛、局部肿胀明显等急性症状时，氢化可的松可以快速抑制炎症细胞因子的释放，缓解骨髓水肿和软组织肿胀。

- **短效糖皮质激素的安全性优势**：相较于长效糖皮质激素，短效糖皮质激素对下丘脑 - 垂体 - 肾上腺（HPA）轴的抑制作用相对较弱。HPA轴在人体的应激反应和内分泌调节中起着关键作用。长期使用长效糖皮质激素可能会导致HPA轴功能紊乱，而短效糖皮质激素在适当剂量下使用，这种风险相对较低。例如，短期使用氢化可的松治疗CNO急性发作，在停药后HPA轴能够较快恢复正常功能，减少了因内分泌紊乱引发的如肾上腺皮质功能减退等潜在并发症的风险。

2. **长效糖皮质激素的特点和劣势**

- **长效糖皮质激素（如地塞米松）作用特点**：长效糖皮质激素的抗炎作用较强且持久，地塞米松的生物半衰期约为36 - 54小时。在CNO治疗中，对于一些病情较为顽固、需要持续抗炎的患者，长效糖皮质激素理论上可以提供更持久的炎症控制。然而，这种长效作用也可能带来一些问题。

- **长效糖皮质激素的安全性劣势**：由于其作用时间长，对HPA轴的抑制作用较强。长期使用会增加多种并发症的发生风险，如骨质疏松、股骨头坏死、血糖升高、感染风险增加等。在CNO患者中，本身骨组织已经处于炎症状态，长期使用长效糖皮质激素导致的骨质疏松可能会进一步加重骨损伤，增加病理性骨折的可能性。而且，一旦出现不良反应，由于其长效的特点，这些不良反应的持续时间也会较长，调整起来相对困难。

3. **治疗选择建议**

- 在CNO治疗初期的急性发作阶段，如果需要使用糖皮质激素快速控制炎症和疼痛，短效糖皮质激素是较好的选择。而对于需要长期维持治疗的患者，一般应尽量避免使用长效糖皮质激素。如果必须使用糖皮质激素长期治疗，应在严格监测下，谨慎使用最低有效剂量的短效糖皮质激素，并配合其他药物（如免疫抑制剂）来控制炎症，以减少糖皮质激素相关并发症的发生。

13成人慢性非细菌性骨炎一线治疗方案是什么？


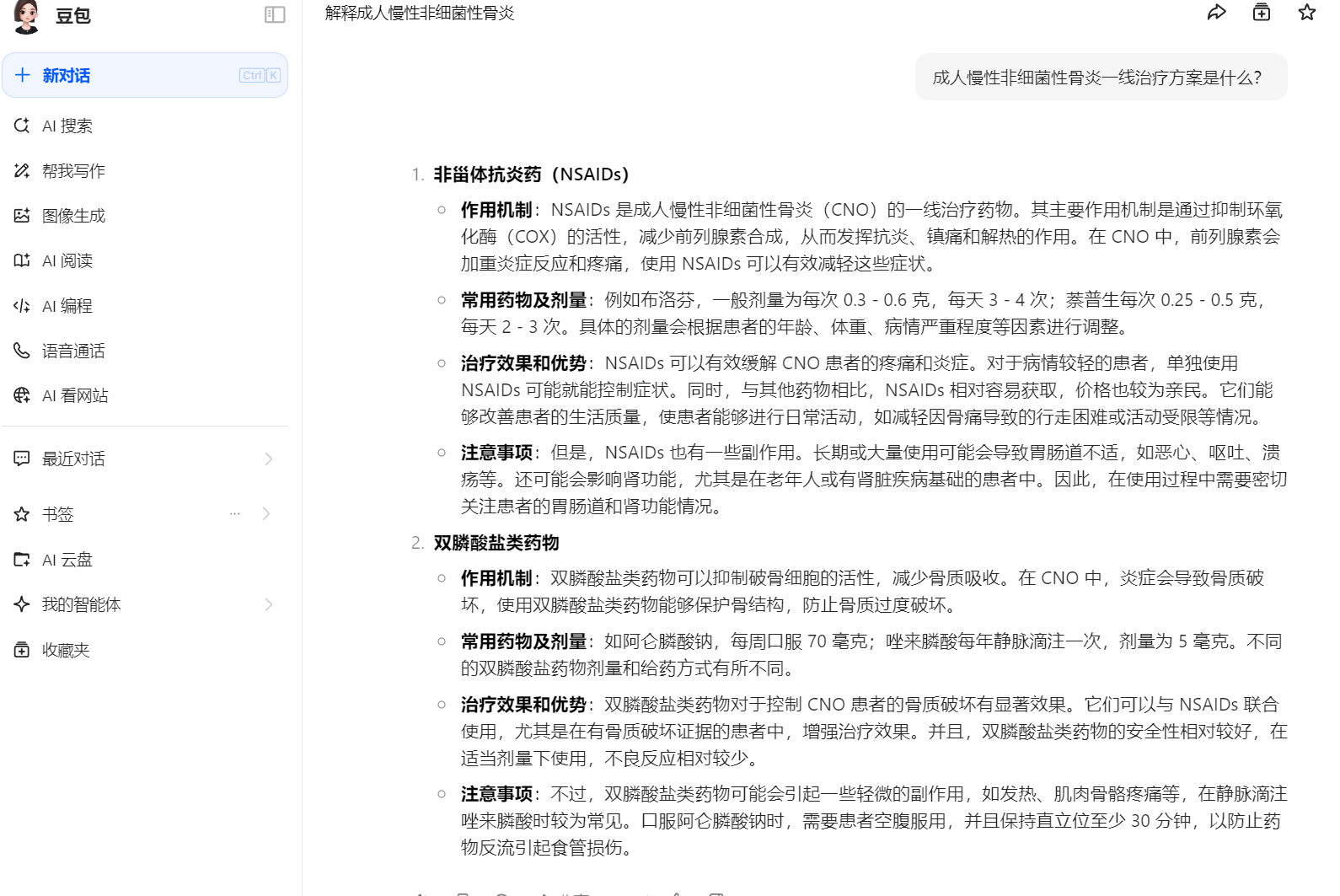
1. **非甾体抗炎药（NSAIDs）**

- **作用机制**：NSAIDs是成人慢性非细菌性骨炎（CNO）的一线治疗药物。其主要作用机制是通过抑制环氧化酶（COX）的活性，减少前列腺素合成，从而发挥抗炎、镇痛和解热的作用。在CNO中，前列腺素会加重炎症反应和疼痛，使用NSAIDs可以有效减轻这些症状。

- **常用药物及剂量**：例如布洛芬，一般剂量为每次0.3 - 0.6克，每天3 - 4次；萘普生每次0.25 - 0.5克，每天2 - 3次。具体的剂量会根据患者的年龄、体重、病情严重程度等因素进行调整。

- **治疗效果和优势**：NSAIDs可以有效缓解CNO患者的疼痛和炎症。对于病情较轻的患者，单独使用NSAIDs可能就能控制症状。同时，与其他药物相比，NSAIDs相对容易获取，价格也较为亲民。它们能够改善患者的生活质量，使患者能够进行日常活动，如减轻因骨痛导致的行走困难或活动受限等情况。

- **注意事项**：但是，NSAIDs也有一些副作用。长期或大量使用可能会导致胃肠道不适，如恶心、呕吐、溃疡等。还可能会影响肾功能，尤其是在老年人或有肾脏疾病基础的患者中。因此，在使用过程中需要密切关注患者的胃肠道和肾功能情况。

2. **双膦酸盐类药物**

- **作用机制**：双膦酸盐类药物可以抑制破骨细胞的活性，减少骨质吸收。在CNO中，炎症会导致骨质破坏，使用双膦酸盐类药物能够保护骨结构，防止骨质过度破坏。

- **常用药物及剂量**：如阿仑膦酸钠，每周口服70毫克；唑来膦酸每年静脉滴注一次，剂量为5毫克。不同的双膦酸盐药物剂量和给药方式有所不同。

- **治疗效果和优势**：双膦酸盐类药物对于控制CNO患者的骨质破坏有显著效果。它们可以与NSAIDs联合使用，尤其是在有骨质破坏证据的患者中，增强治疗效果。并且，双膦酸盐类药物的安全性相对较好，在适当剂量下使用，不良反应相对较少。

- **注意事项**：不过，双膦酸盐类药物可能会引起一些轻微的副作用，如发热、肌肉骨骼疼痛等，在静脉滴注唑来膦酸时较为常见。口服阿仑膦酸钠时，需要患者空腹服用，并且保持直立位至少30分钟，以防止药物反流引起食管损伤。

14无临床症状，也没有放射学疾病活动的慢性非细菌性骨炎的病人需要治疗吗？


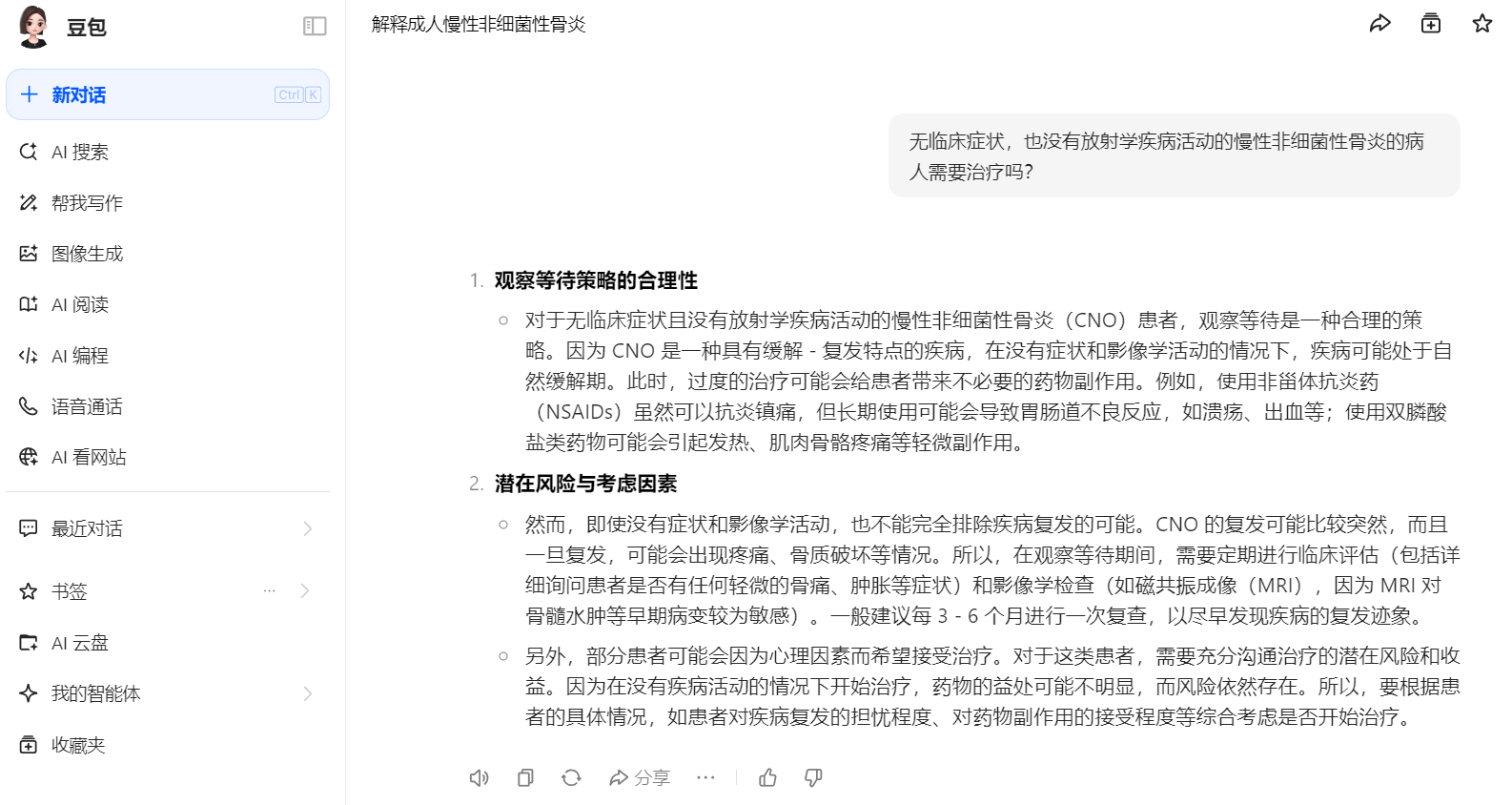
1. **观察等待策略的合理性**

- 对于无临床症状且没有放射学疾病活动的慢性非细菌性骨炎（CNO）患者，观察等待是一种合理的策略。因为CNO是一种具有缓解 - 复发特点的疾病，在没有症状和影像学活动的情况下，疾病可能处于自然缓解期。此时，过度的治疗可能会给患者带来不必要的药物副作用。例如，使用非甾体抗炎药（NSAIDs）虽然可以抗炎镇痛，但长期使用可能会导致胃肠道不良反应，如溃疡、出血等；使用双膦酸盐类药物可能会引起发热、肌肉骨骼疼痛等轻微副作用。

2. **潜在风险与考虑因素**

- 然而，即使没有症状和影像学活动，也不能完全排除疾病复发的可能。CNO的复发可能比较突然，而且一旦复发，可能会出现疼痛、骨质破坏等情况。所以，在观察等待期间，需要定期进行临床评估（包括详细询问患者是否有任何轻微的骨痛、肿胀等症状）和影像学检查（如磁共振成像（MRI），因为MRI对骨髓水肿等早期病变较为敏感）。一般建议每3 - 6个月进行一次复查，以尽早发现疾病的复发迹象。

- 另外，部分患者可能会因为心理因素而希望接受治疗。对于这类患者，需要充分沟通治疗的潜在风险和收益。因为在没有疾病活动的情况下开始治疗，药物的益处可能不明显，而风险依然存在。所以，要根据患者的具体情况，如患者对疾病复发的担忧程度、对药物副作用的接受程度等综合考虑是否开始治疗。

15有临床症状没有放射学疾病活动的慢性非细菌性骨炎的病人如何处理？


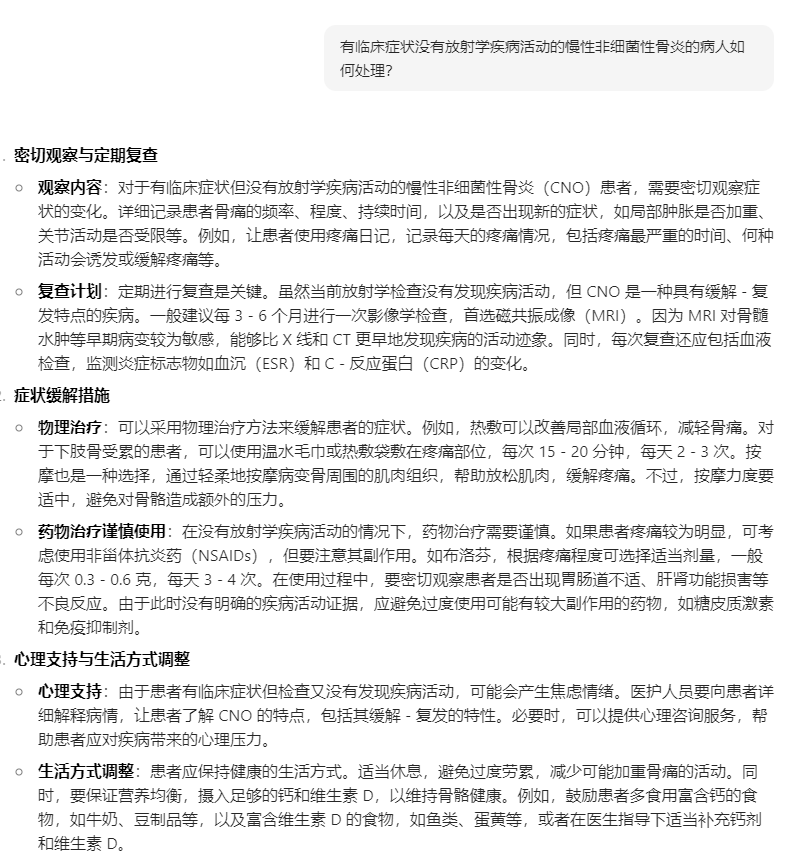
1. **密切观察与定期复查**

- **观察内容**：对于有临床症状但没有放射学疾病活动的慢性非细菌性骨炎（CNO）患者，需要密切观察症状的变化。详细记录患者骨痛的频率、程度、持续时间，以及是否出现新的症状，如局部肿胀是否加重、关节活动是否受限等。例如，让患者使用疼痛日记，记录每天的疼痛情况，包括疼痛最严重的时间、何种活动会诱发或缓解疼痛等。

- **复查计划**：定期进行复查是关键。虽然当前放射学检查没有发现疾病活动，但CNO是一种具有缓解 - 复发特点的疾病。一般建议每3 - 6个月进行一次影像学检查，首选磁共振成像（MRI）。因为MRI对骨髓水肿等早期病变较为敏感，能够比X线和CT更早地发现疾病的活动迹象。同时，每次复查还应包括血液检查，监测炎症标志物如血沉（ESR）和C -反应蛋白（CRP）的变化。

2. **症状缓解措施**

- **物理治疗**：可以采用物理治疗方法来缓解患者的症状。例如，热敷可以改善局部血液循环，减轻骨痛。对于下肢骨受累的患者，可以使用温水毛巾或热敷袋敷在疼痛部位，每次15 - 20分钟，每天2 - 3次。按摩也是一种选择，通过轻柔地按摩病变骨周围的肌肉组织，帮助放松肌肉，缓解疼痛。不过，按摩力度要适中，避免对骨骼造成额外的压力。

- **药物治疗谨慎使用**：在没有放射学疾病活动的情况下，药物治疗需要谨慎。如果患者疼痛较为明显，可考虑使用非甾体抗炎药（NSAIDs），但要注意其副作用。如布洛芬，根据疼痛程度可选择适当剂量，一般每次0.3 - 0.6克，每天3 - 4次。在使用过程中，要密切观察患者是否出现胃肠道不适、肝肾功能损害等不良反应。由于此时没有明确的疾病活动证据，应避免过度使用可能有较大副作用的药物，如糖皮质激素和免疫抑制剂。

3. **心理支持与生活方式调整**

- **心理支持**：由于患者有临床症状但检查又没有发现疾病活动，可能会产生焦虑情绪。医护人员要向患者详细解释病情，让患者了解CNO的特点，包括其缓解 - 复发的特性。必要时，可以提供心理咨询服务，帮助患者应对疾病带来的心理压力。

- **生活方式调整**：患者应保持健康的生活方式。适当休息，避免过度劳累，减少可能加重骨痛的活动。同时，要保证营养均衡，摄入足够的钙和维生素D，以维持骨骼健康。例如，鼓励患者多食用富含钙的食物，如牛奶、豆制品等，以及富含维生素D的食物，如鱼类、蛋黄等，或者在医生指导下适当补充钙剂和维生素D。

16有椎体塌陷风险的脊柱骨病变的慢性非细菌性骨炎患者和出现明显骨骼累积损伤的慢性非细菌性骨炎患者如何治疗？


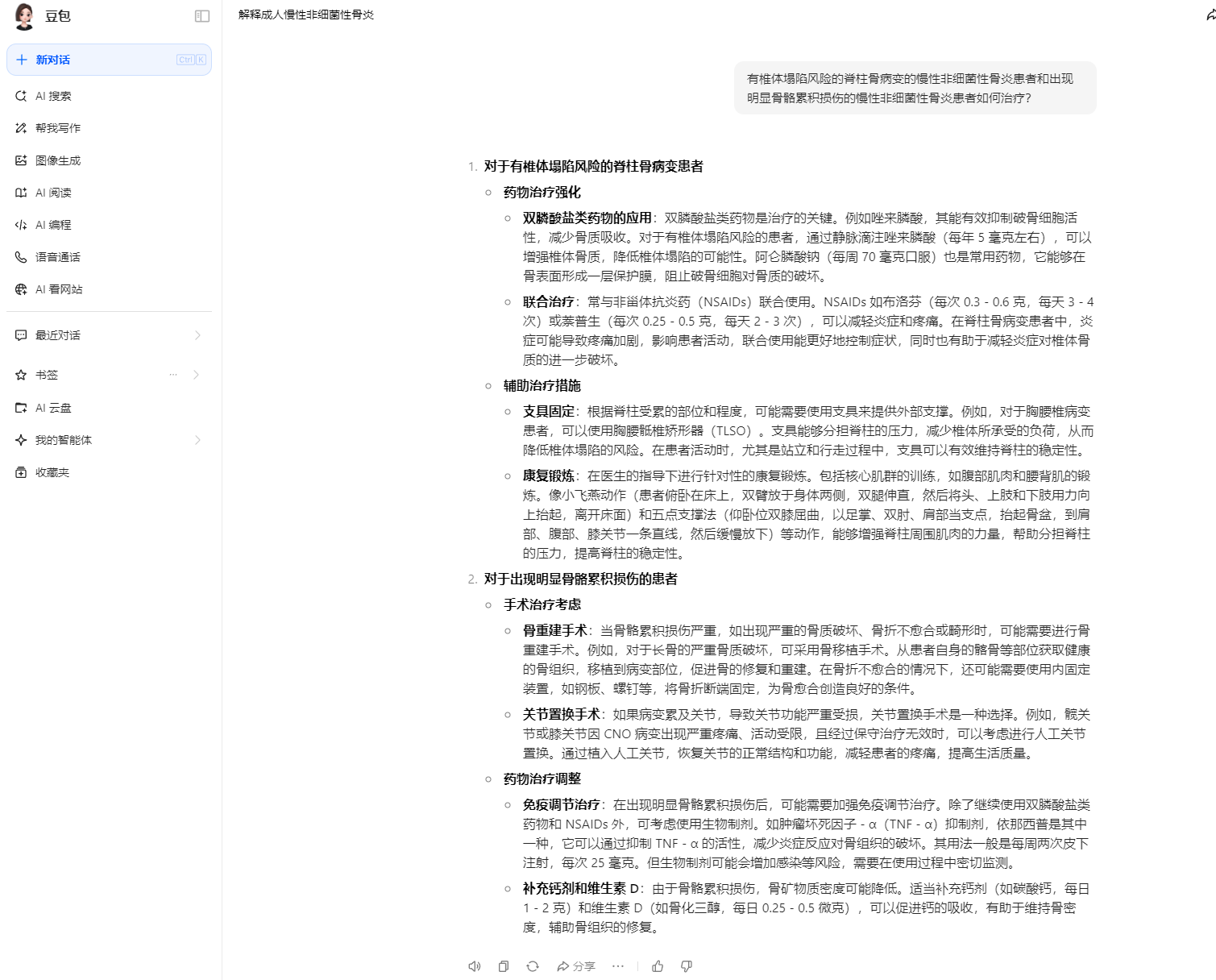
1. **对于有椎体塌陷风险的脊柱骨病变患者**

- **药物治疗强化**

- **双膦酸盐类药物的应用**：双膦酸盐类药物是治疗的关键。例如唑来膦酸，其能有效抑制破骨细胞活性，减少骨质吸收。对于有椎体塌陷风险的患者，通过静脉滴注唑来膦酸（每年5毫克左右），可以增强椎体骨质，降低椎体塌陷的可能性。阿仑膦酸钠（每周70毫克口服）也是常用药物，它能够在骨表面形成一层保护膜，阻止破骨细胞对骨质的破坏。

- **联合治疗**：常与非甾体抗炎药（NSAIDs）联合使用。NSAIDs如布洛芬（每次0.3 - 0.6克，每天3 - 4次）或萘普生（每次0.25 - 0.5克，每天2 - 3次），可以减轻炎症和疼痛。在脊柱骨病变患者中，炎症可能导致疼痛加剧，影响患者活动，联合使用能更好地控制症状，同时也有助于减轻炎症对椎体骨质的进一步破坏。

- **辅助治疗措施**

- **支具固定**：根据脊柱受累的部位和程度，可能需要使用支具来提供外部支撑。例如，对于胸腰椎病变患者，可以使用胸腰骶椎矫形器（TLSO）。支具能够分担脊柱的压力，减少椎体所承受的负荷，从而降低椎体塌陷的风险。在患者活动时，尤其是站立和行走过程中，支具可以有效维持脊柱的稳定性。

- **康复锻炼**：在医生的指导下进行针对性的康复锻炼。包括核心肌群的训练，如腹部肌肉和腰背肌的锻炼。像小飞燕动作（患者俯卧在床上，双臂放于身体两侧，双腿伸直，然后将头、上肢和下肢用力向上抬起，离开床面）和五点支撑法（仰卧位双膝屈曲，以足掌、双肘、肩部当支点，抬起骨盆，到肩部、腹部、膝关节一条直线，然后缓慢放下）等动作，能够增强脊柱周围肌肉的力量，帮助分担脊柱的压力，提高脊柱的稳定性。

2. **对于出现明显骨骼累积损伤的患者**

- **手术治疗考虑**

- **骨重建手术**：当骨骼累积损伤严重，如出现严重的骨质破坏、骨折不愈合或畸形时，可能需要进行骨重建手术。例如，对于长骨的严重骨质破坏，可采用骨移植手术。从患者自身的髂骨等部位获取健康的骨组织，移植到病变部位，促进骨的修复和重建。在骨折不愈合的情况下，还可能需要使用内固定装置，如钢板、螺钉等，将骨折断端固定，为骨愈合创造良好的条件。

- **关节置换手术**：如果病变累及关节，导致关节功能严重受损，关节置换手术是一种选择。例如，髋关节或膝关节因CNO病变出现严重疼痛、活动受限，且经过保守治疗无效时，可以考虑进行人工关节置换。通过植入人工关节，恢复关节的正常结构和功能，减轻患者的疼痛，提高生活质量。

- **药物治疗调整**

- **免疫调节治疗**：在出现明显骨骼累积损伤后，可能需要加强免疫调节治疗。除了继续使用双膦酸盐类药物和NSAIDs外，可考虑使用生物制剂。如肿瘤坏死因子 - α（TNF - α）抑制剂，依那西普是其中一种，它可以通过抑制TNF - α的活性，减少炎症反应对骨组织的破坏。其用法一般是每周两次皮下注射，每次25毫克。但生物制剂可能会增加感染等风险，需要在使用过程中密切监测。

- **补充钙剂和维生素D**：由于骨骼累积损伤，骨矿物质密度可能降低。适当补充钙剂（如碳酸钙，每日1 - 2克）和维生素D（如骨化三醇，每日0.25 - 0.5微克），可以促进钙的吸收，有助于维持骨密度，辅助骨组织的修复。
