# Supplementary material for: Evaluation of the accuracy and repeatability of Deepseek V3, Doubao, and Kimi1.5 in answering knowledge-related queries about chronic non-bacterial osteitis
Source: Front Artif Intell. 2025 Sep 29;8:1629149. doi: 10.3389/frai.2025.1629149 (PMC12515971; doi:10.3389/frai.2025.1629149)
Supplement: Supplementary file 1 [file Data_Sheet_1.zip › day1/day1-kimi.docx]

1成人慢性非细菌性骨炎的定义是什么？


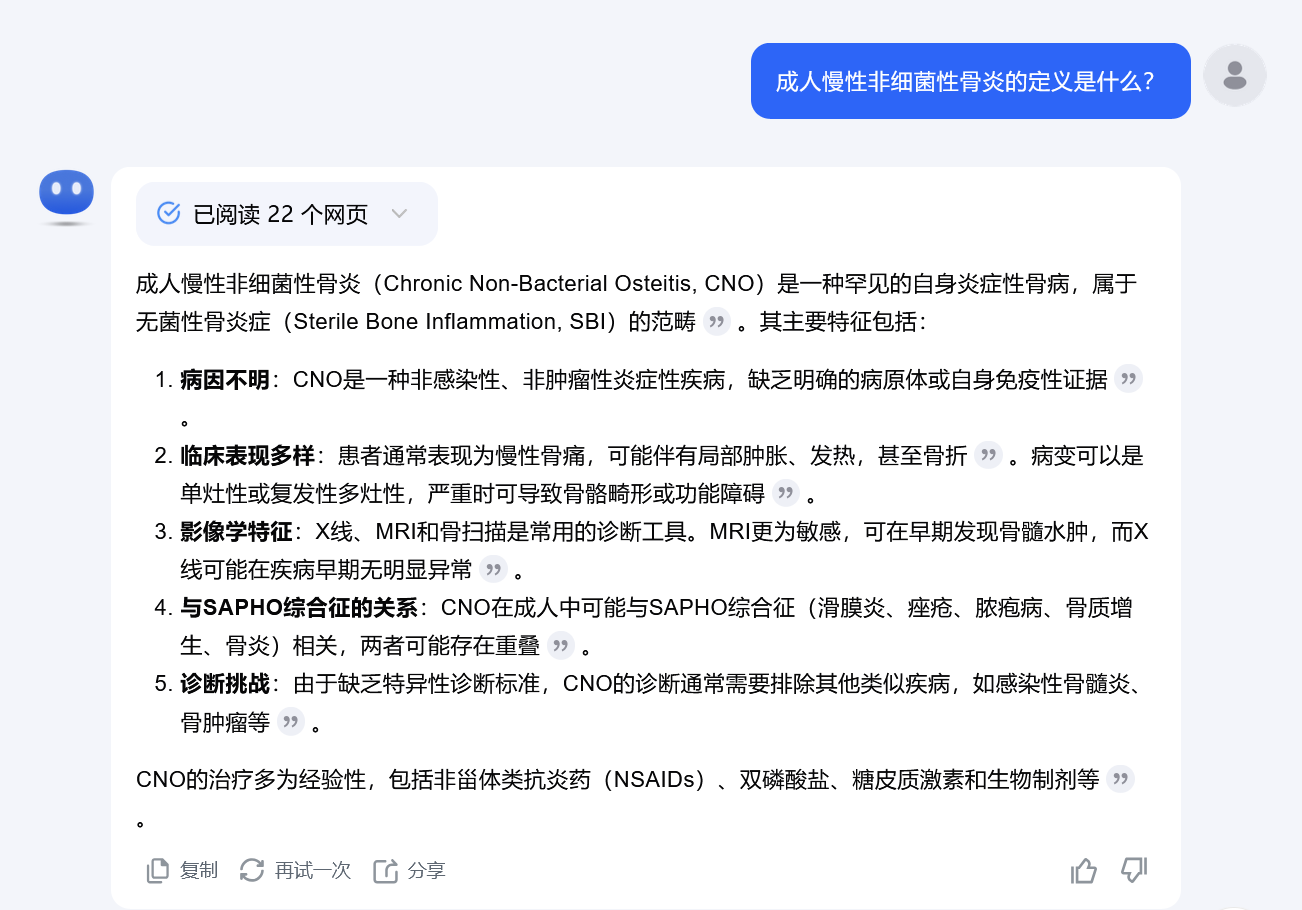


成人慢性非细菌性骨炎（Chronic Non-Bacterial Osteitis, CNO）是一种罕见的自身炎症性骨病，属于无菌性骨炎症（Sterile Bone Inflammation, SBI）的范畴。其主要特征包括：

1. **病因不明**：CNO是一种非感染性、非肿瘤性炎症性疾病，缺乏明确的病原体或自身免疫性证据。

2. **临床表现多样**：患者通常表现为慢性骨痛，可能伴有局部肿胀、发热，甚至骨折。病变可以是单灶性或复发性多灶性，严重时可导致骨骼畸形或功能障碍。

3. **影像学特征**：X线、MRI和骨扫描是常用的诊断工具。MRI更为敏感，可在早期发现骨髓水肿，而X线可能在疾病早期无明显异常。

4. **与SAPHO综合征的关系**：CNO在成人中可能与SAPHO综合征（滑膜炎、痤疮、脓疱病、骨质增生、骨炎）相关，两者可能存在重叠。

5. **诊断挑战**：由于缺乏特异性诊断标准，CNO的诊断通常需要排除其他类似疾病，如感染性骨髓炎、骨肿瘤等。

CNO的治疗多为经验性，包括非甾体类抗炎药（NSAIDs）、双磷酸盐、糖皮质激素和生物制剂等。

**2成人慢性非细菌性骨炎的典型影像学特征是什么？**


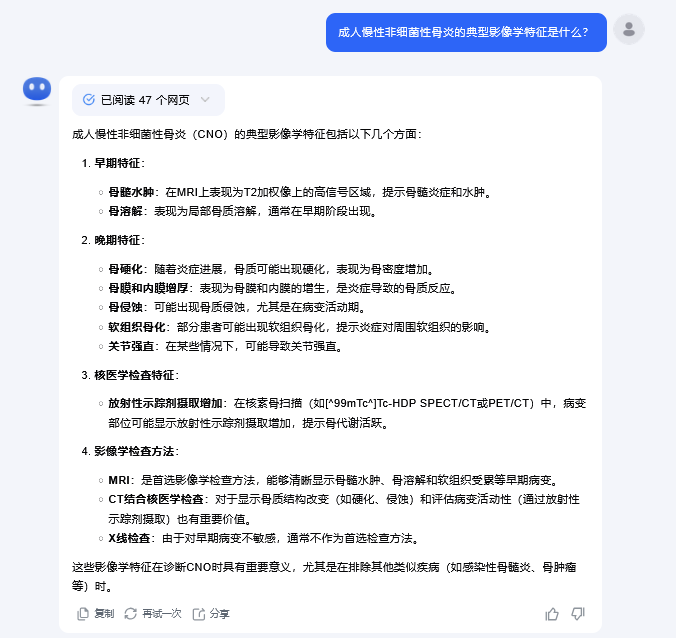


成人慢性非细菌性骨炎（CNO）的典型影像学特征包括以下几个方面：

1. **早期特征**：

- **骨髓水肿**：在MRI上表现为T2加权像上的高信号区域，提示骨髓炎症和水肿。

- **骨溶解**：表现为局部骨质溶解，通常在早期阶段出现。

2. **晚期特征**：

- **骨硬化**：随着炎症进展，骨质可能出现硬化，表现为骨密度增加。

- **骨膜和内膜增厚**：表现为骨膜和内膜的增生，是炎症导致的骨质反应。

- **骨侵蚀**：可能出现骨质侵蚀，尤其是在病变活动期。

- **软组织骨化**：部分患者可能出现软组织骨化，提示炎症对周围软组织的影响。

- **关节强直**：在某些情况下，可能导致关节强直。

3. **核医学检查特征**：

- **放射性示踪剂摄取增加**：在核素骨扫描（如[^99mTc^]Tc-HDP SPECT/CT或PET/CT）中，病变部位可能显示放射性示踪剂摄取增加，提示骨代谢活跃。

4. **影像学检查方法**：

- **MRI**：是首选影像学检查方法，能够清晰显示骨髓水肿、骨溶解和软组织受累等早期病变。

- **CT结合核医学检查**：对于显示骨质结构改变（如硬化、侵蚀）和评估病变活动性（通过放射性示踪剂摄取）也有重要价值。

- **X线检查**：由于对早期病变不敏感，通常不作为首选检查方法。

这些影像学特征在诊断CNO时具有重要意义，尤其是在排除其他类似疾病（如感染性骨髓炎、骨肿瘤等）时。

3成人慢性非细菌性骨炎最常见的表现是什么？


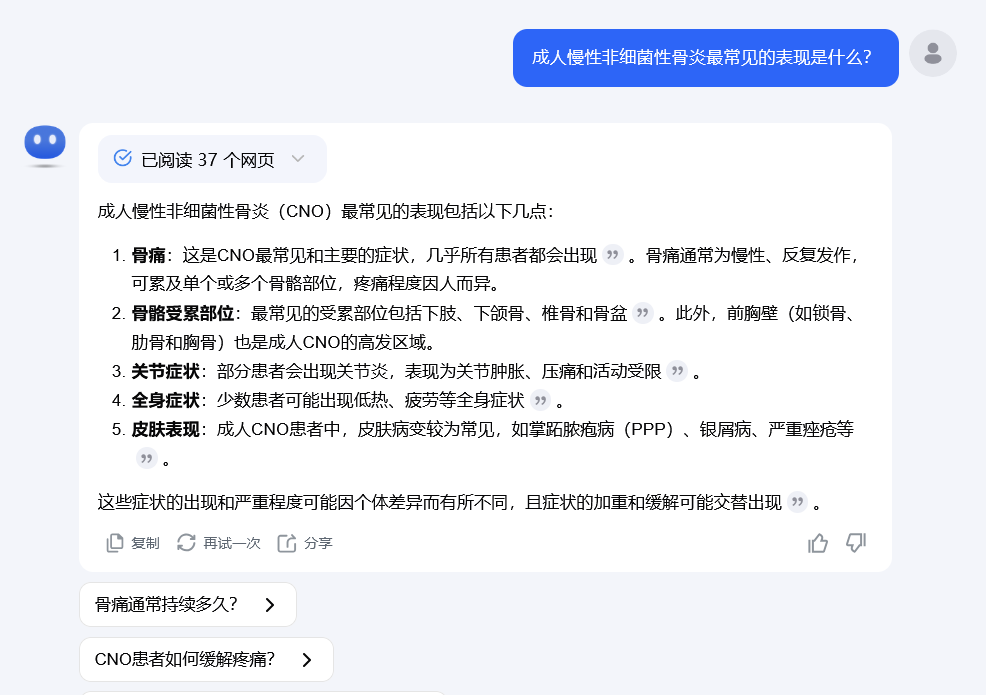


成人慢性非细菌性骨炎（CNO）最常见的表现包括以下几点：

1. **骨痛**：这是CNO最常见和主要的症状，几乎所有患者都会出现。骨痛通常为慢性、反复发作，可累及单个或多个骨骼部位，疼痛程度因人而异。

2. **骨骼受累部位**：最常见的受累部位包括下肢、下颌骨、椎骨和骨盆。此外，前胸壁（如锁骨、肋骨和胸骨）也是成人CNO的高发区域。

3. **关节症状**：部分患者会出现关节炎，表现为关节肿胀、压痛和活动受限。

4. **全身症状**：少数患者可能出现低热、疲劳等全身症状。

5. **皮肤表现**：成人CNO患者中，皮肤病变较为常见，如掌跖脓疱病（PPP）、银屑病、严重痤疮等。

这些症状的出现和严重程度可能因个体差异而有所不同，且症状的加重和缓解可能交替出现。

4成人慢性非细菌性骨炎最常受累的部位是那些？


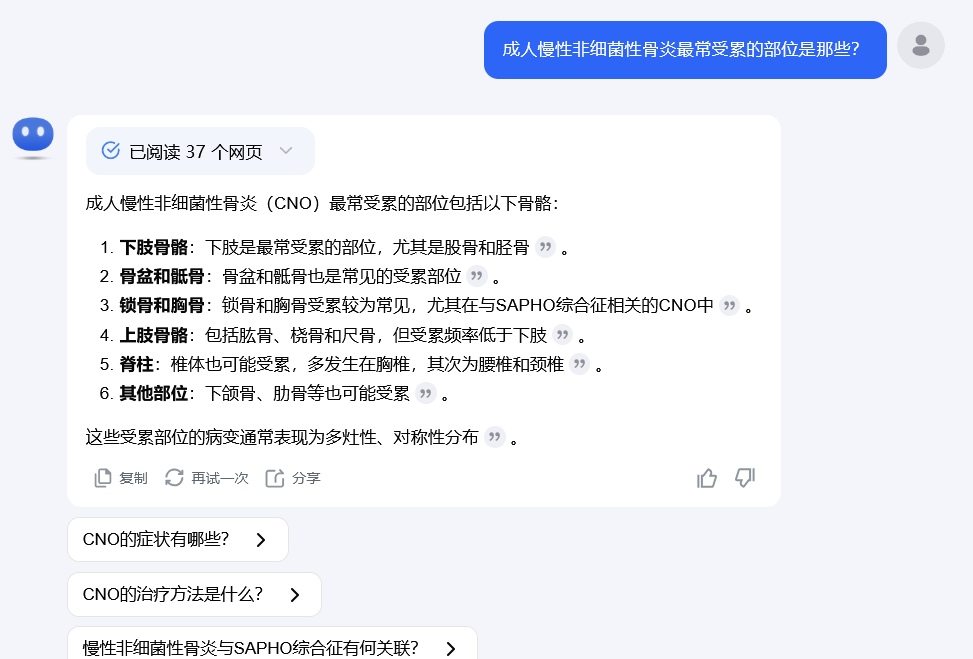


成人慢性非细菌性骨炎（CNO）最常受累的部位包括以下骨骼：

1. **下肢骨骼**：下肢是最常受累的部位，尤其是股骨和胫骨。

2. **骨盆和骶骨**：骨盆和骶骨也是常见的受累部位。

3. **锁骨和胸骨**：锁骨和胸骨受累较为常见，尤其在与SAPHO综合征相关的CNO中。

4. **上肢骨骼**：包括肱骨、桡骨和尺骨，但受累频率低于下肢。

5. **脊柱**：椎体也可能受累，多发生在胸椎，其次为腰椎和颈椎。

6. **其他部位**：下颌骨、肋骨等也可能受累。

这些受累部位的病变通常表现为多灶性、对称性分布。

5成人慢性非细菌性骨炎有没有特异性的炎症标志物？


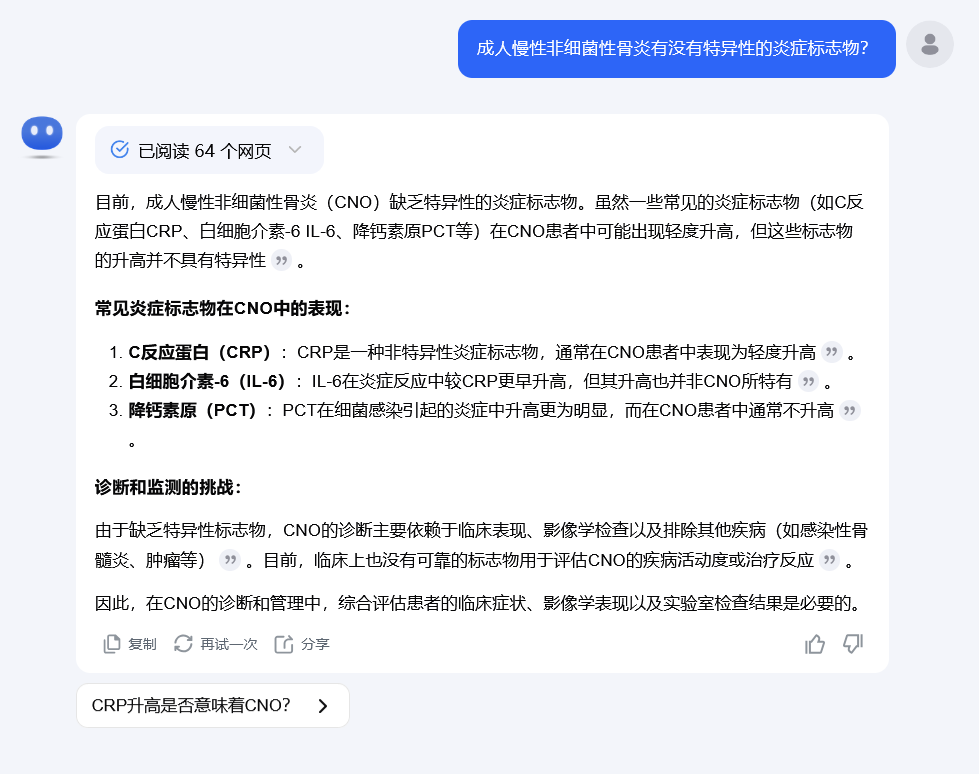


目前，成人慢性非细菌性骨炎（CNO）缺乏特异性的炎症标志物。虽然一些常见的炎症标志物（如C反应蛋白CRP、白细胞介素-6 IL-6、降钙素原PCT等）在CNO患者中可能出现轻度升高，但这些标志物的升高并不具有特异性。

### 常见炎症标志物在CNO中的表现：

1. **C反应蛋白（CRP）**：CRP是一种非特异性炎症标志物，通常在CNO患者中表现为轻度升高。

2. **白细胞介素-6（IL-6）**：IL-6在炎症反应中较CRP更早升高，但其升高也并非CNO所特有。

3. **降钙素原（PCT）**：PCT在细菌感染引起的炎症中升高更为明显，而在CNO患者中通常不升高。

### 诊断和监测的挑战：

由于缺乏特异性标志物，CNO的诊断主要依赖于临床表现、影像学检查以及排除其他疾病（如感染性骨髓炎、肿瘤等）。目前，临床上也没有可靠的标志物用于评估CNO的疾病活动度或治疗反应。

因此，在CNO的诊断和管理中，综合评估患者的临床症状、影像学表现以及实验室检查结果是必要的。

6慢性非细菌性骨炎首选的影像学检查是什么？


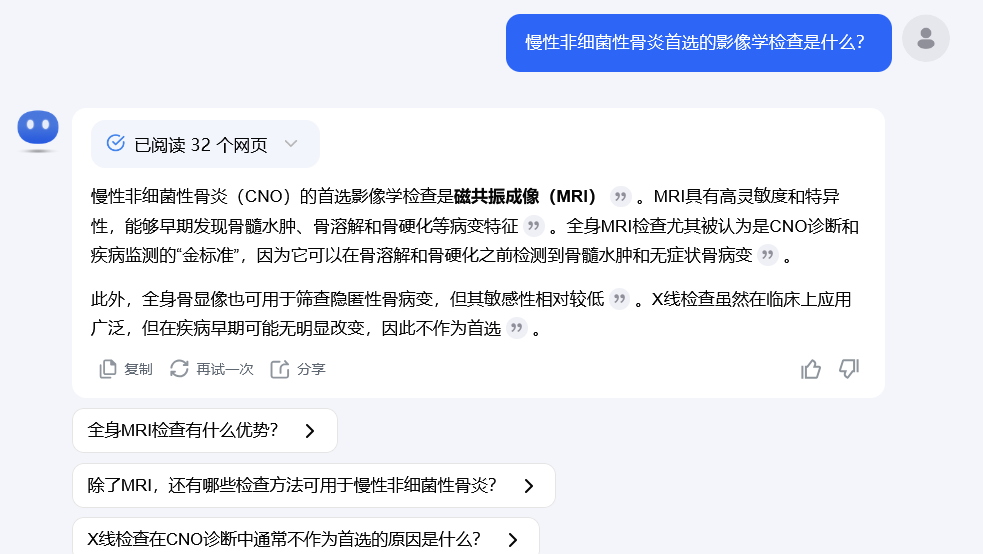


慢性非细菌性骨炎（CNO）的首选影像学检查是**磁共振成像（MRI）**。MRI具有高灵敏度和特异性，能够早期发现骨髓水肿、骨溶解和骨硬化等病变特征。全身MRI检查尤其被认为是CNO诊断和疾病监测的“金标准”，因为它可以在骨溶解和骨硬化之前检测到骨髓水肿和无症状骨病变。

此外，全身骨显像也可用于筛查隐匿性骨病变，但其敏感性相对较低。X线检查虽然在临床上应用广泛，但在疾病早期可能无明显改变，因此不作为首选。

7全身成像检查是不是诊断成人慢性非细菌性骨炎的先决条件？


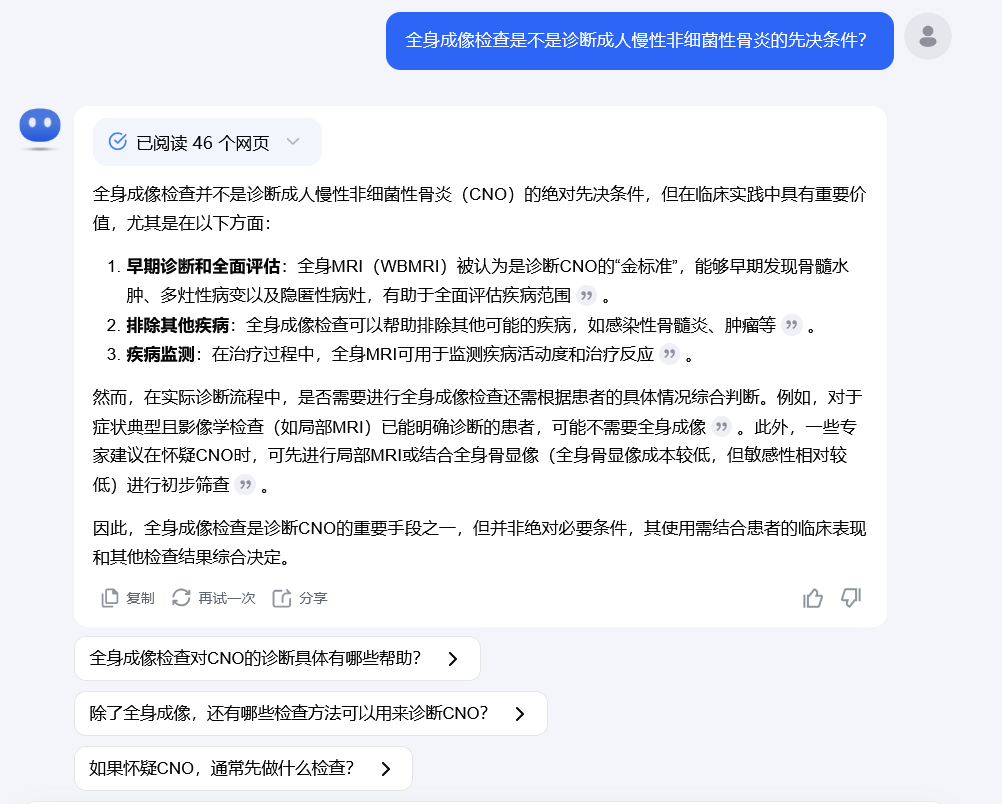


全身成像检查并不是诊断成人慢性非细菌性骨炎（CNO）的绝对先决条件，但在临床实践中具有重要价值，尤其是在以下方面：

1. **早期诊断和全面评估**：全身MRI（WBMRI）被认为是诊断CNO的“金标准”，能够早期发现骨髓水肿、多灶性病变以及隐匿性病灶，有助于全面评估疾病范围。

2. **排除其他疾病**：全身成像检查可以帮助排除其他可能的疾病，如感染性骨髓炎、肿瘤等。

3. **疾病监测**：在治疗过程中，全身MRI可用于监测疾病活动度和治疗反应。

然而，在实际诊断流程中，是否需要进行全身成像检查还需根据患者的具体情况综合判断。例如，对于症状典型且影像学检查（如局部MRI）已能明确诊断的患者，可能不需要全身成像。此外，一些专家建议在怀疑CNO时，可先进行局部MRI或结合全身骨显像（全身骨显像成本较低，但敏感性相对较低）进行初步筛查。

因此，全身成像检查是诊断CNO的重要手段之一，但并非绝对必要条件，其使用需结合患者的临床表现和其他检查结果综合决定。

8诊断成人慢性非细菌性骨炎有必要常规骨活检吗？


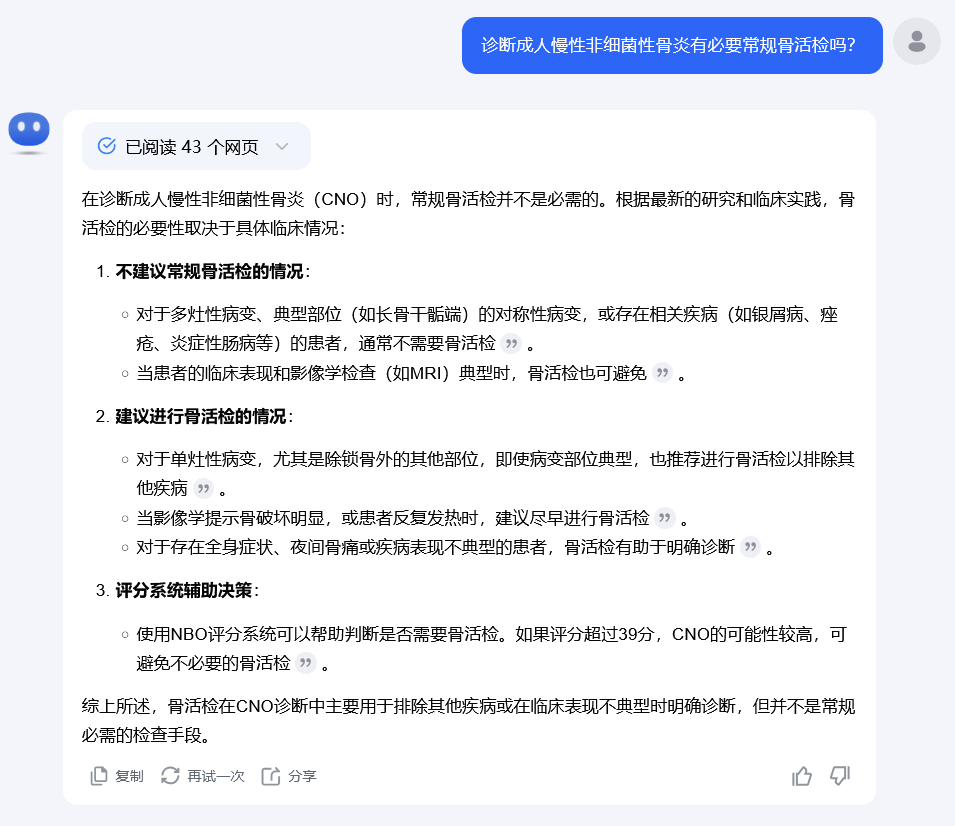


在诊断成人慢性非细菌性骨炎（CNO）时，常规骨活检并不是必需的。根据最新的研究和临床实践，骨活检的必要性取决于具体临床情况：

1. **不建议常规骨活检的情况**：

- 对于多灶性病变、典型部位（如长骨干骺端）的对称性病变，或存在相关疾病（如银屑病、痤疮、炎症性肠病等）的患者，通常不需要骨活检。

- 当患者的临床表现和影像学检查（如MRI）典型时，骨活检也可避免。

2. **建议进行骨活检的情况**：

- 对于单灶性病变，尤其是除锁骨外的其他部位，即使病变部位典型，也推荐进行骨活检以排除其他疾病。

- 当影像学提示骨破坏明显，或患者反复发热时，建议尽早进行骨活检。

- 对于存在全身症状、夜间骨痛或疾病表现不典型的患者，骨活检有助于明确诊断。

3. **评分系统辅助决策**：

- 使用NBO评分系统可以帮助判断是否需要骨活检。如果评分超过39分，CNO的可能性较高，可避免不必要的骨活检。

综上所述，骨活检在CNO诊断中主要用于排除其他疾病或在临床表现不典型时明确诊断，但并不是常规必需的检查手段。

9慢性非细菌性骨炎如何与恶性骨肿瘤鉴别？


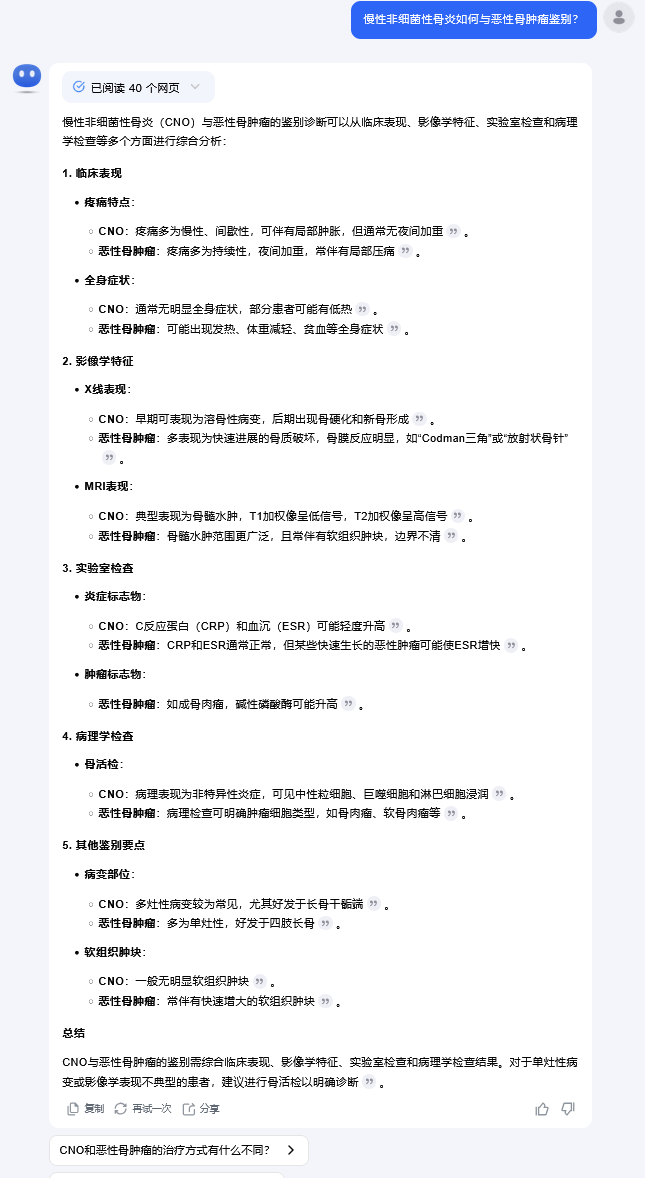


慢性非细菌性骨炎（CNO）与恶性骨肿瘤的鉴别诊断可以从临床表现、影像学特征、实验室检查和病理学检查等多个方面进行综合分析：

### 1. 临床表现

- **疼痛特点**：

- **CNO**：疼痛多为慢性、间歇性，可伴有局部肿胀，但通常无夜间加重。

- **恶性骨肿瘤**：疼痛多为持续性，夜间加重，常伴有局部压痛。

- **全身症状**：

- **CNO**：通常无明显全身症状，部分患者可能有低热。

- **恶性骨肿瘤**：可能出现发热、体重减轻、贫血等全身症状。

### 2. 影像学特征

- **X线表现**：

- **CNO**：早期可表现为溶骨性病变，后期出现骨硬化和新骨形成。

- **恶性骨肿瘤**：多表现为快速进展的骨质破坏，骨膜反应明显，如“Codman三角”或“放射状骨针”。

- **MRI表现**：

- **CNO**：典型表现为骨髓水肿，T1加权像呈低信号，T2加权像呈高信号。

- **恶性骨肿瘤**：骨髓水肿范围更广泛，且常伴有软组织肿块，边界不清。

### 3. 实验室检查

- **炎症标志物**：

- **CNO**：C反应蛋白（CRP）和血沉（ESR）可能轻度升高。

- **恶性骨肿瘤**：CRP和ESR通常正常，但某些快速生长的恶性肿瘤可能使ESR增快。

- **肿瘤标志物**：

- **恶性骨肿瘤**：如成骨肉瘤，碱性磷酸酶可能升高。

### 4. 病理学检查

- **骨活检**：

- **CNO**：病理表现为非特异性炎症，可见中性粒细胞、巨噬细胞和淋巴细胞浸润。

- **恶性骨肿瘤**：病理检查可明确肿瘤细胞类型，如骨肉瘤、软骨肉瘤等。

### 5. 其他鉴别要点

- **病变部位**：

- **CNO**：多灶性病变较为常见，尤其好发于长骨干骺端。

- **恶性骨肿瘤**：多为单灶性，好发于四肢长骨。

- **软组织肿块**：

- **CNO**：一般无明显软组织肿块。

- **恶性骨肿瘤**：常伴有快速增大的软组织肿块。

### 总结

CNO与恶性骨肿瘤的鉴别需综合临床表现、影像学特征、实验室检查和病理学检查结果。对于单灶性病变或影像学表现不典型的患者，建议进行骨活检以明确诊断。

10慢性非细菌性骨炎如何与骨关节炎鉴别？


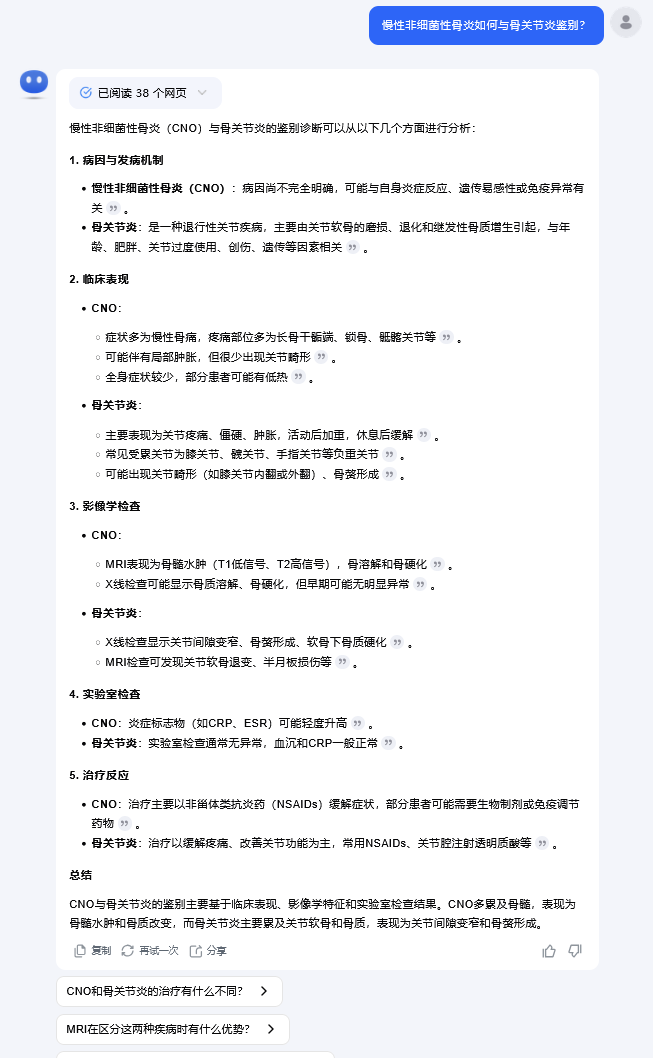
慢性非细菌性骨炎（CNO）与骨关节炎的鉴别诊断可以从以下几个方面进行分析：

### 1. 病因与发病机制

- **慢性非细菌性骨炎（CNO）**：病因尚不完全明确，可能与自身炎症反应、遗传易感性或免疫异常有关。

- **骨关节炎**：是一种退行性关节疾病，主要由关节软骨的磨损、退化和继发性骨质增生引起，与年龄、肥胖、关节过度使用、创伤、遗传等因素相关。

### 2. 临床表现

- **CNO**：

- 症状多为慢性骨痛，疼痛部位多为长骨干骺端、锁骨、骶髂关节等。

- 可能伴有局部肿胀，但很少出现关节畸形。

- 全身症状较少，部分患者可能有低热。

- **骨关节炎**：

- 主要表现为关节疼痛、僵硬、肿胀，活动后加重，休息后缓解。

- 常见受累关节为膝关节、髋关节、手指关节等负重关节。

- 可能出现关节畸形（如膝关节内翻或外翻）、骨赘形成。

### 3. 影像学检查

- **CNO**：

- MRI表现为骨髓水肿（T1低信号、T2高信号），骨溶解和骨硬化。

- X线检查可能显示骨质溶解、骨硬化，但早期可能无明显异常。

- **骨关节炎**：

- X线检查显示关节间隙变窄、骨赘形成、软骨下骨质硬化。

- MRI检查可发现关节软骨退变、半月板损伤等。

### 4. 实验室检查

- **CNO**：炎症标志物（如CRP、ESR）可能轻度升高。

- **骨关节炎**：实验室检查通常无异常，血沉和CRP一般正常。

### 5. 治疗反应

- **CNO**：治疗主要以非甾体类抗炎药（NSAIDs）缓解症状，部分患者可能需要生物制剂或免疫调节药物。

- **骨关节炎**：治疗以缓解疼痛、改善关节功能为主，常用NSAIDs、关节腔注射透明质酸等。

### 总结

CNO与骨关节炎的鉴别主要基于临床表现、影像学特征和实验室检查结果。CNO多累及骨髓，表现为骨髓水肿和骨质改变，而骨关节炎主要累及关节软骨和骨质，表现为关节间隙变窄和骨赘形成。

11慢性非细菌性骨炎如何与纤维发育不良鉴别？


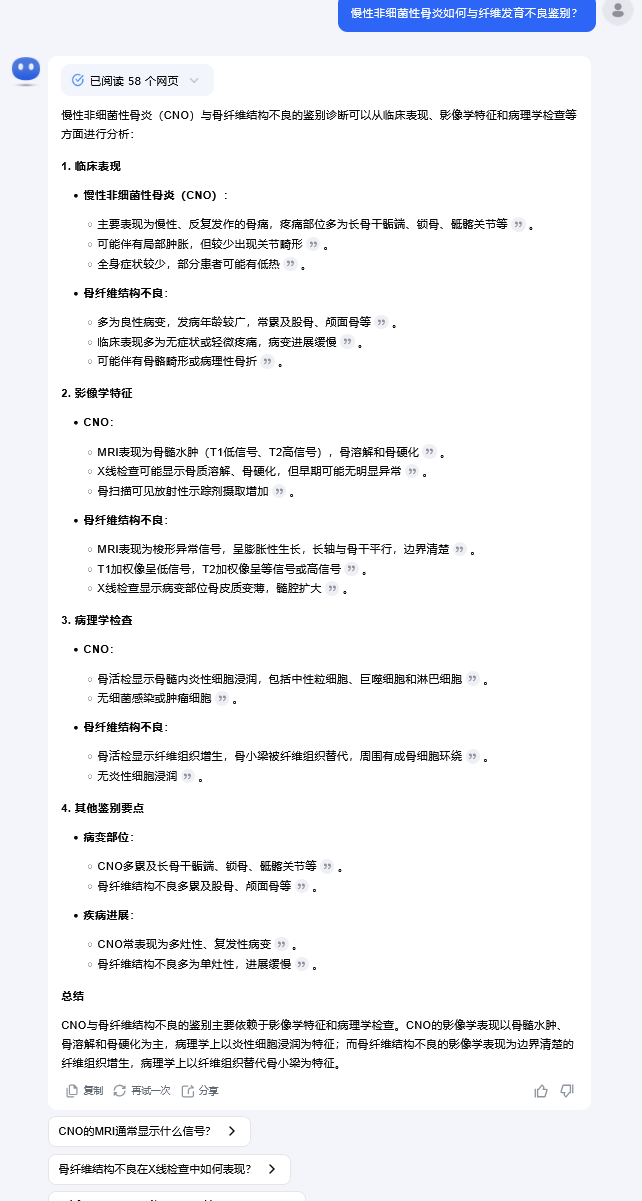


慢性非细菌性骨炎（CNO）与骨纤维结构不良的鉴别诊断可以从临床表现、影像学特征和病理学检查等方面进行分析：

### 1. 临床表现

- **慢性非细菌性骨炎（CNO）**：

- 主要表现为慢性、反复发作的骨痛，疼痛部位多为长骨干骺端、锁骨、骶髂关节等。

- 可能伴有局部肿胀，但较少出现关节畸形。

- 全身症状较少，部分患者可能有低热。

- **骨纤维结构不良**：

- 多为良性病变，发病年龄较广，常累及股骨、颅面骨等。

- 临床表现多为无症状或轻微疼痛，病变进展缓慢。

- 可能伴有骨骼畸形或病理性骨折。

### 2. 影像学特征

- **CNO**：

- MRI表现为骨髓水肿（T1低信号、T2高信号），骨溶解和骨硬化。

- X线检查可能显示骨质溶解、骨硬化，但早期可能无明显异常。

- 骨扫描可见放射性示踪剂摄取增加。

- **骨纤维结构不良**：

- MRI表现为梭形异常信号，呈膨胀性生长，长轴与骨干平行，边界清楚。

- T1加权像呈低信号，T2加权像呈等信号或高信号。

- X线检查显示病变部位骨皮质变薄，髓腔扩大。

### 3. 病理学检查

- **CNO**：

- 骨活检显示骨髓内炎性细胞浸润，包括中性粒细胞、巨噬细胞和淋巴细胞。

- 无细菌感染或肿瘤细胞。

- **骨纤维结构不良**：

- 骨活检显示纤维组织增生，骨小梁被纤维组织替代，周围有成骨细胞环绕。

- 无炎性细胞浸润。

### 4. 其他鉴别要点

- **病变部位**：

- CNO多累及长骨干骺端、锁骨、骶髂关节等。

- 骨纤维结构不良多累及股骨、颅面骨等。

- **疾病进展**：

- CNO常表现为多灶性、复发性病变。

- 骨纤维结构不良多为单灶性，进展缓慢。

### 总结

CNO与骨纤维结构不良的鉴别主要依赖于影像学特征和病理学检查。CNO的影像学表现以骨髓水肿、骨溶解和骨硬化为主，病理学上以炎性细胞浸润为特征；而骨纤维结构不良的影像学表现为边界清楚的纤维组织增生，病理学上以纤维组织替代骨小梁为特征。

12在治慢性非细菌性骨炎过程中应该选择短疗程的糖皮质激素治疗还是长期的糖皮质激素治疗？


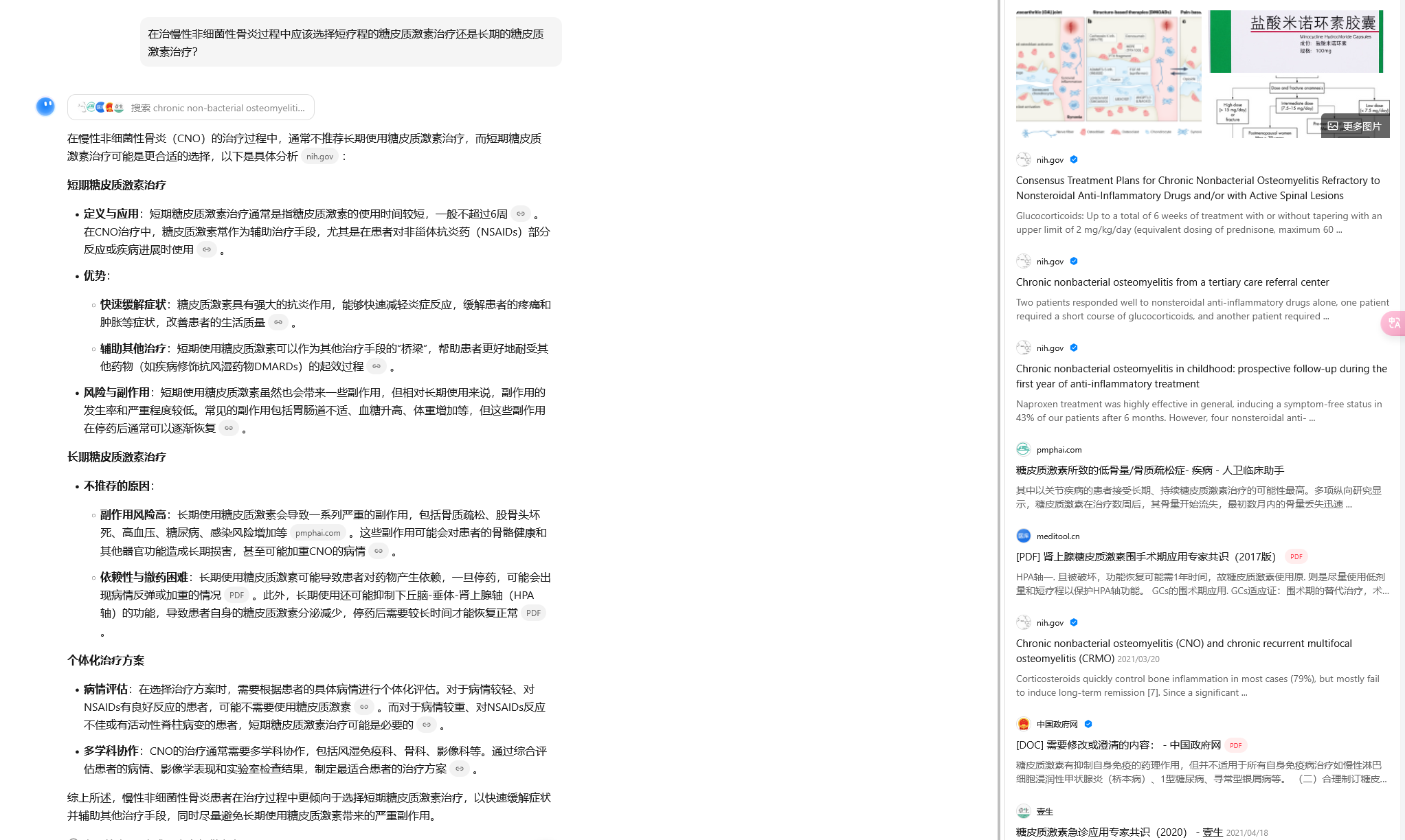


在慢性非细菌性骨炎（CNO）的治疗过程中，通常不推荐长期使用糖皮质激素治疗，而短期糖皮质激素治疗可能是更合适的选择，以下是具体分析：

### 短期糖皮质激素治疗

- **定义与应用**：短期糖皮质激素治疗通常是指糖皮质激素的使用时间较短，一般不超过6周。在CNO治疗中，糖皮质激素常作为辅助治疗手段，尤其是在患者对非甾体抗炎药（NSAIDs）部分反应或疾病进展时使用。

- **优势**：

- **快速缓解症状**：糖皮质激素具有强大的抗炎作用，能够快速减轻炎症反应，缓解患者的疼痛和肿胀等症状，改善患者的生活质量。

- **辅助其他治疗**：短期使用糖皮质激素可以作为其他治疗手段的“桥梁”，帮助患者更好地耐受其他药物（如疾病修饰抗风湿药物DMARDs）的起效过程。

- **风险与副作用**：短期使用糖皮质激素虽然也会带来一些副作用，但相对长期使用来说，副作用的发生率和严重程度较低。常见的副作用包括胃肠道不适、血糖升高、体重增加等，但这些副作用在停药后通常可以逐渐恢复。

### 长期糖皮质激素治疗

- **不推荐的原因**：

- **副作用风险高**：长期使用糖皮质激素会导致一系列严重的副作用，包括骨质疏松、股骨头坏死、高血压、糖尿病、感染风险增加等。这些副作用可能会对患者的骨骼健康和其他器官功能造成长期损害，甚至可能加重CNO的病情。

- **依赖性与撤药困难**：长期使用糖皮质激素可能导致患者对药物产生依赖，一旦停药，可能会出现病情反弹或加重的情况。此外，长期使用还可能抑制下丘脑-垂体-肾上腺轴（HPA轴）的功能，导致患者自身的糖皮质激素分泌减少，停药后需要较长时间才能恢复正常。

### 个体化治疗方案

- **病情评估**：在选择治疗方案时，需要根据患者的具体病情进行个体化评估。对于病情较轻、对NSAIDs有良好反应的患者，可能不需要使用糖皮质激素。而对于病情较重、对NSAIDs反应不佳或有活动性脊柱病变的患者，短期糖皮质激素治疗可能是必要的。

- **多学科协作**：CNO的治疗通常需要多学科协作，包括风湿免疫科、骨科、影像科等。通过综合评估患者的病情、影像学表现和实验室检查结果，制定最适合患者的治疗方案。

综上所述，慢性非细菌性骨炎患者在治疗过程中更倾向于选择短期糖皮质激素治疗，以快速缓解症状并辅助其他治疗手段，同时尽量避免长期使用糖皮质激素带来的严重副作用。

13成人慢性非细菌性骨炎一线治疗方案是什么？


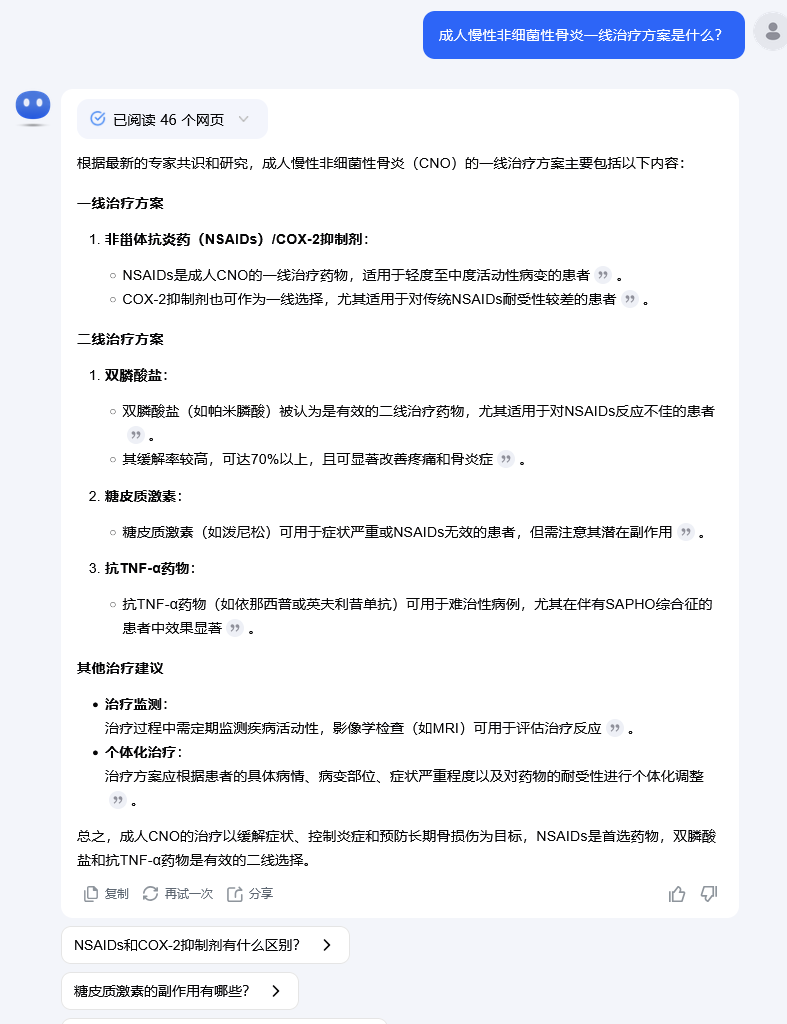
根据最新的专家共识和研究，成人慢性非细菌性骨炎（CNO）的一线治疗方案主要包括以下内容：

### 一线治疗方案

1. **非甾体抗炎药（NSAIDs）/COX-2抑制剂**：

- NSAIDs是成人CNO的一线治疗药物，适用于轻度至中度活动性病变的患者。

- COX-2抑制剂也可作为一线选择，尤其适用于对传统NSAIDs耐受性较差的患者。

### 二线治疗方案

1. **双膦酸盐**：

- 双膦酸盐（如帕米膦酸）被认为是有效的二线治疗药物，尤其适用于对NSAIDs反应不佳的患者。

- 其缓解率较高，可达70%以上，且可显著改善疼痛和骨炎症。

2. **糖皮质激素**：

- 糖皮质激素（如泼尼松）可用于症状严重或NSAIDs无效的患者，但需注意其潜在副作用。

3. **抗TNF-α药物**：

- 抗TNF-α药物（如依那西普或英夫利昔单抗）可用于难治性病例，尤其在伴有SAPHO综合征的患者中效果显著。

### 其他治疗建议

- **治疗监测**：

治疗过程中需定期监测疾病活动性，影像学检查（如MRI）可用于评估治疗反应。

- **个体化治疗**：

治疗方案应根据患者的具体病情、病变部位、症状严重程度以及对药物的耐受性进行个体化调整。

总之，成人CNO的治疗以缓解症状、控制炎症和预防长期骨损伤为目标，NSAIDs是首选药物，双膦酸盐和抗TNF-α药物是有效的二线选择。

14无临床症状，也没有放射学疾病活动的慢性非细菌性骨炎的病人需要治疗吗？
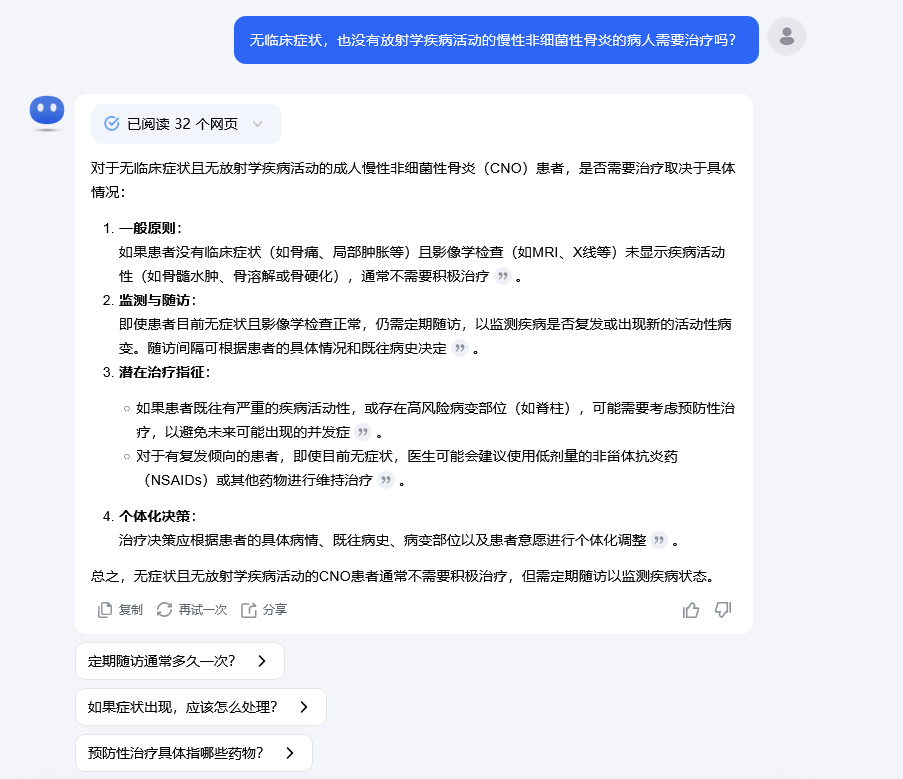
对于无临床症状且无放射学疾病活动的成人慢性非细菌性骨炎（CNO）患者，是否需要治疗取决于具体情况：

1. **一般原则**：

如果患者没有临床症状（如骨痛、局部肿胀等）且影像学检查（如MRI、X线等）未显示疾病活动性（如骨髓水肿、骨溶解或骨硬化），通常不需要积极治疗。

2. **监测与随访**：

即使患者目前无症状且影像学检查正常，仍需定期随访，以监测疾病是否复发或出现新的活动性病变。随访间隔可根据患者的具体情况和既往病史决定。

3. **潜在治疗指征**：

- 如果患者既往有严重的疾病活动性，或存在高风险病变部位（如脊柱），可能需要考虑预防性治疗，以避免未来可能出现的并发症。

- 对于有复发倾向的患者，即使目前无症状，医生可能会建议使用低剂量的非甾体抗炎药（NSAIDs）或其他药物进行维持治疗。

4. **个体化决策**：

治疗决策应根据患者的具体病情、既往病史、病变部位以及患者意愿进行个体化调整。

总之，无症状且无放射学疾病活动的CNO患者通常不需要积极治疗，但需定期随访以监测疾病状态。

15有临床症状没有放射学疾病活动的慢性非细菌性骨炎的病人如何处理？


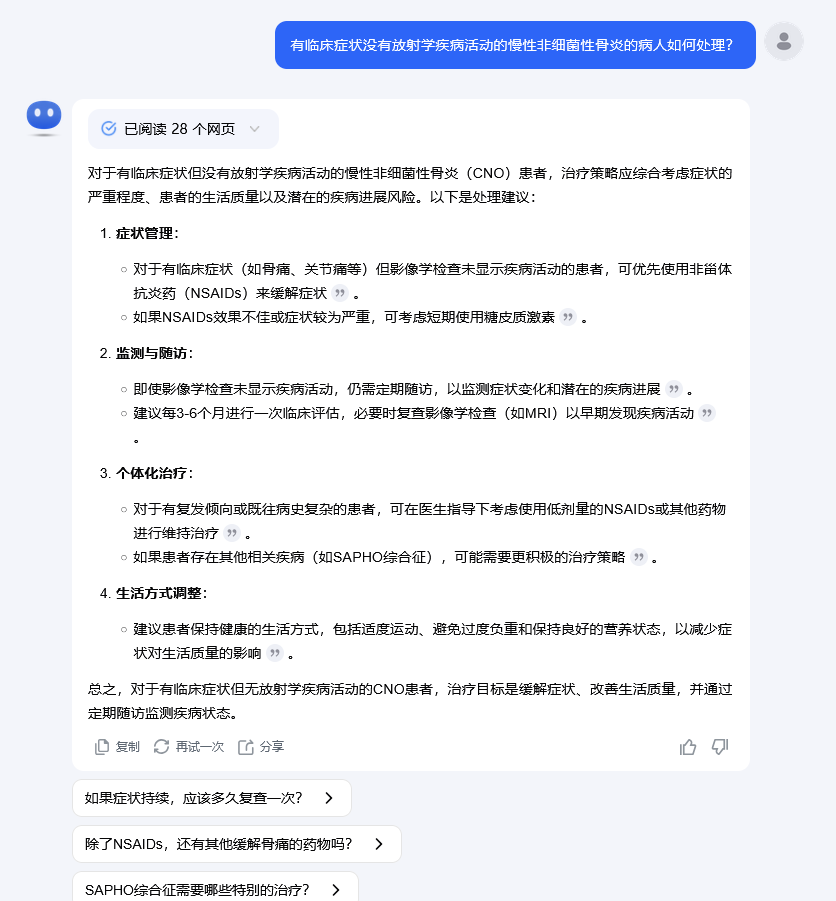


对于有临床症状但没有放射学疾病活动的慢性非细菌性骨炎（CNO）患者，治疗策略应综合考虑症状的严重程度、患者的生活质量以及潜在的疾病进展风险。以下是处理建议：

1. **症状管理**：

- 对于有临床症状（如骨痛、关节痛等）但影像学检查未显示疾病活动的患者，可优先使用非甾体抗炎药（NSAIDs）来缓解症状。

- 如果NSAIDs效果不佳或症状较为严重，可考虑短期使用糖皮质激素。

2. **监测与随访**：

- 即使影像学检查未显示疾病活动，仍需定期随访，以监测症状变化和潜在的疾病进展。

- 建议每3-6个月进行一次临床评估，必要时复查影像学检查（如MRI）以早期发现疾病活动。

3. **个体化治疗**：

- 对于有复发倾向或既往病史复杂的患者，可在医生指导下考虑使用低剂量的NSAIDs或其他药物进行维持治疗。

- 如果患者存在其他相关疾病（如SAPHO综合征），可能需要更积极的治疗策略。

4. **生活方式调整**：

- 建议患者保持健康的生活方式，包括适度运动、避免过度负重和保持良好的营养状态，以减少症状对生活质量的影响。

总之，对于有临床症状但无放射学疾病活动的CNO患者，治疗目标是缓解症状、改善生活质量，并通过定期随访监测疾病状态。

16有椎体塌陷风险的脊柱骨病变的慢性非细菌性骨炎患者和出现明显骨骼累积损伤的慢性非细菌性骨炎患者如何治疗？


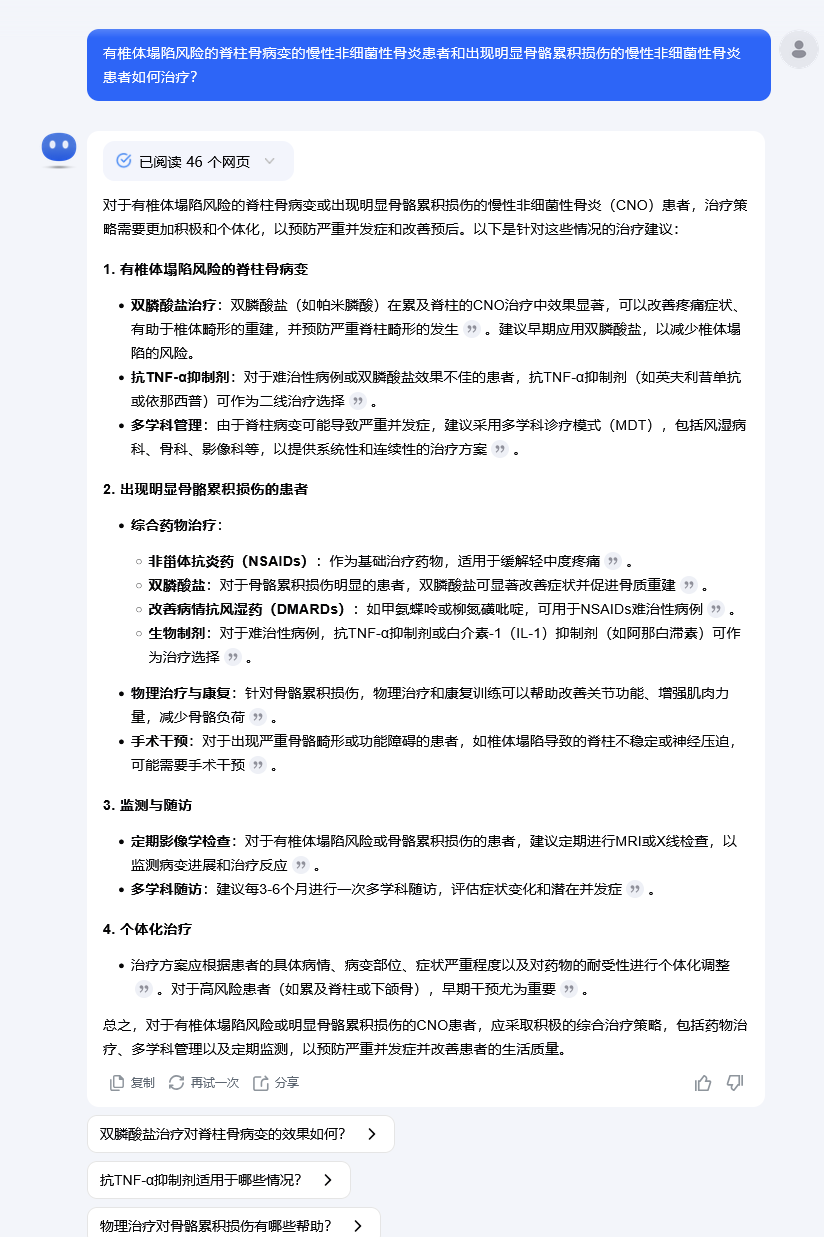


对于有椎体塌陷风险的脊柱骨病变或出现明显骨骼累积损伤的慢性非细菌性骨炎（CNO）患者，治疗策略需要更加积极和个体化，以预防严重并发症和改善预后。以下是针对这些情况的治疗建议：

### 1. **有椎体塌陷风险的脊柱骨病变**

- **双膦酸盐治疗**：双膦酸盐（如帕米膦酸）在累及脊柱的CNO治疗中效果显著，可以改善疼痛症状、有助于椎体畸形的重建，并预防严重脊柱畸形的发生。建议早期应用双膦酸盐，以减少椎体塌陷的风险。

- **抗TNF-α抑制剂**：对于难治性病例或双膦酸盐效果不佳的患者，抗TNF-α抑制剂（如英夫利昔单抗或依那西普）可作为二线治疗选择。

- **多学科管理**：由于脊柱病变可能导致严重并发症，建议采用多学科诊疗模式（MDT），包括风湿病科、骨科、影像科等，以提供系统性和连续性的治疗方案。

### 2. **出现明显骨骼累积损伤的患者**

- **综合药物治疗**：

- **非甾体抗炎药（NSAIDs）**：作为基础治疗药物，适用于缓解轻中度疼痛。

- **双膦酸盐**：对于骨骼累积损伤明显的患者，双膦酸盐可显著改善症状并促进骨质重建。

- **改善病情抗风湿药（DMARDs）**：如甲氨蝶呤或柳氮磺吡啶，可用于NSAIDs难治性病例。

- **生物制剂**：对于难治性病例，抗TNF-α抑制剂或白介素-1（IL-1）抑制剂（如阿那白滞素）可作为治疗选择。

- **物理治疗与康复**：针对骨骼累积损伤，物理治疗和康复训练可以帮助改善关节功能、增强肌肉力量，减少骨骼负荷。

- **手术干预**：对于出现严重骨骼畸形或功能障碍的患者，如椎体塌陷导致的脊柱不稳定或神经压迫，可能需要手术干预。

### 3. **监测与随访**

- **定期影像学检查**：对于有椎体塌陷风险或骨骼累积损伤的患者，建议定期进行MRI或X线检查，以监测病变进展和治疗反应。

- **多学科随访**：建议每3-6个月进行一次多学科随访，评估症状变化和潜在并发症。

### 4. **个体化治疗**

- 治疗方案应根据患者的具体病情、病变部位、症状严重程度以及对药物的耐受性进行个体化调整。对于高风险患者（如累及脊柱或下颌骨），早期干预尤为重要。

总之，对于有椎体塌陷风险或明显骨骼累积损伤的CNO患者，应采取积极的综合治疗策略，包括药物治疗、多学科管理以及定期监测，以预防严重并发症并改善患者的生活质量。
