# Supplementary material for: Evaluation of the accuracy and repeatability of Deepseek V3, Doubao, and Kimi1.5 in answering knowledge-related queries about chronic non-bacterial osteitis
Source: Front Artif Intell. 2025 Sep 29;8:1629149. doi: 10.3389/frai.2025.1629149 (PMC12515971; doi:10.3389/frai.2025.1629149)
Supplement: Supplementary file 1 [file Data_Sheet_1.zip › day14/Day14-deepseek.docx]

1成人慢性非细菌性骨炎的定义是什么？


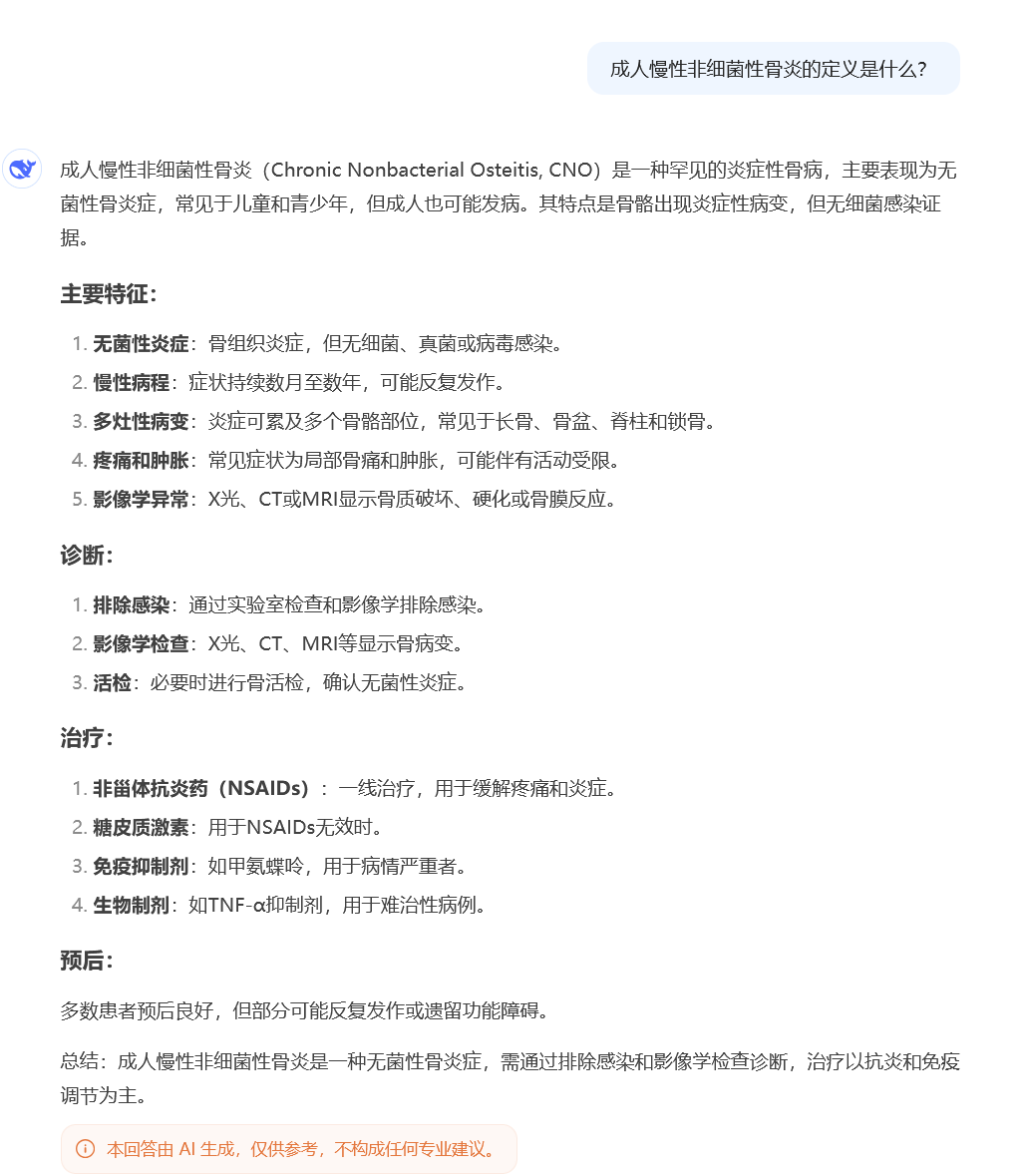
成人慢性非细菌性骨炎（Chronic Nonbacterial Osteitis, CNO）是一种罕见的炎症性骨病，主要表现为无菌性骨炎症，常见于儿童和青少年，但成人也可能发病。其特点是骨骼出现炎症性病变，但无细菌感染证据。

### 主要特征：

1. **无菌性炎症**：骨组织炎症，但无细菌、真菌或病毒感染。

2. **慢性病程**：症状持续数月至数年，可能反复发作。

3. **多灶性病变**：炎症可累及多个骨骼部位，常见于长骨、骨盆、脊柱和锁骨。

4. **疼痛和肿胀**：常见症状为局部骨痛和肿胀，可能伴有活动受限。

5. **影像学异常**：X光、CT或MRI显示骨质破坏、硬化或骨膜反应。

### 诊断：

1. **排除感染**：通过实验室检查和影像学排除感染。

2. **影像学检查**：X光、CT、MRI等显示骨病变。

3. **活检**：必要时进行骨活检，确认无菌性炎症。

### 治疗：

1. **非甾体抗炎药（NSAIDs）**：一线治疗，用于缓解疼痛和炎症。

2. **糖皮质激素**：用于NSAIDs无效时。

3. **免疫抑制剂**：如甲氨蝶呤，用于病情严重者。

4. **生物制剂**：如TNF-α抑制剂，用于难治性病例。

### 预后：

多数患者预后良好，但部分可能反复发作或遗留功能障碍。

总结：成人慢性非细菌性骨炎是一种无菌性骨炎症，需通过排除感染和影像学检查诊断，治疗以抗炎和免疫调节为主。

2成人慢性非细菌性骨炎的典型影像学特征是什么？


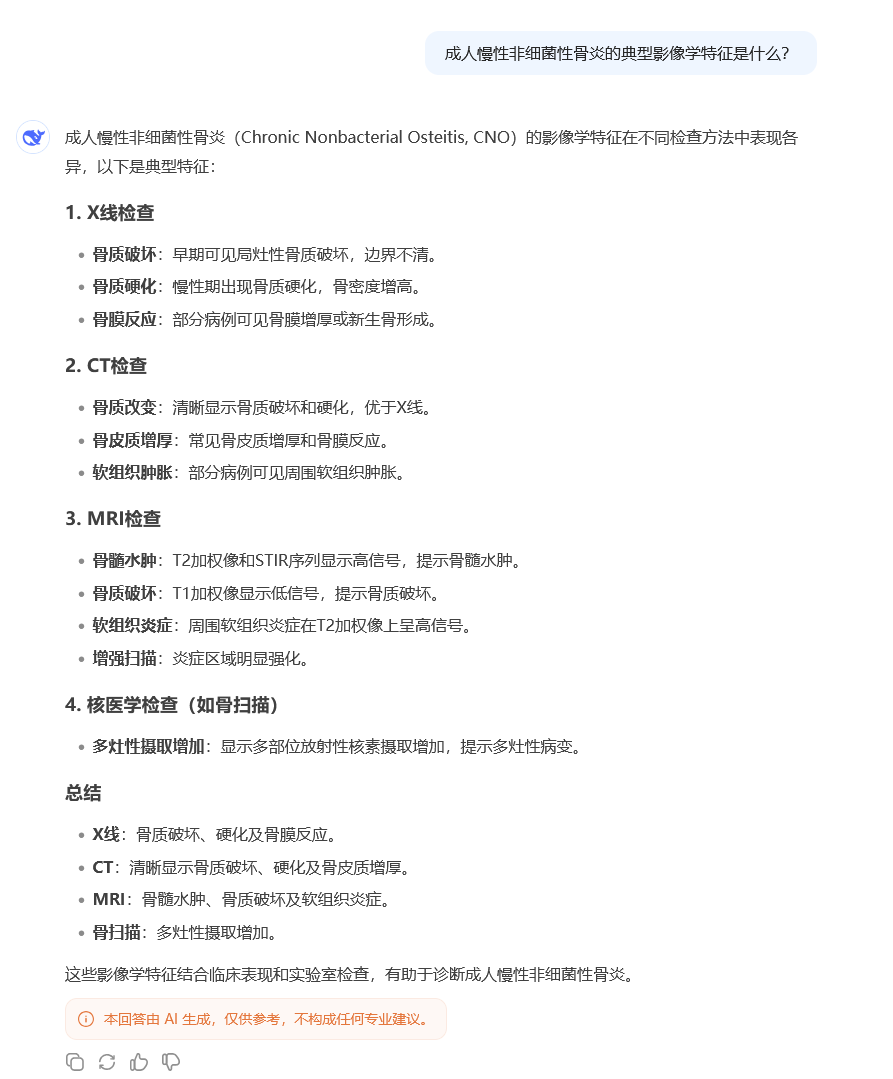
成人慢性非细菌性骨炎（Chronic Nonbacterial Osteitis, CNO）的影像学特征在不同检查方法中表现各异，以下是典型特征：

### 1. **X线检查**

- **骨质破坏**：早期可见局灶性骨质破坏，边界不清。

- **骨质硬化**：慢性期出现骨质硬化，骨密度增高。

- **骨膜反应**：部分病例可见骨膜增厚或新生骨形成。

### 2. **CT检查**

- **骨质改变**：清晰显示骨质破坏和硬化，优于X线。

- **骨皮质增厚**：常见骨皮质增厚和骨膜反应。

- **软组织肿胀**：部分病例可见周围软组织肿胀。

### 3. **MRI检查**

- **骨髓水肿**：T2加权像和STIR序列显示高信号，提示骨髓水肿。

- **骨质破坏**：T1加权像显示低信号，提示骨质破坏。

- **软组织炎症**：周围软组织炎症在T2加权像上呈高信号。

- **增强扫描**：炎症区域明显强化。

### 4. **核医学检查（如骨扫描）**

- **多灶性摄取增加**：显示多部位放射性核素摄取增加，提示多灶性病变。

### 总结

- **X线**：骨质破坏、硬化及骨膜反应。

- **CT**：清晰显示骨质破坏、硬化及骨皮质增厚。

- **MRI**：骨髓水肿、骨质破坏及软组织炎症。

- **骨扫描**：多灶性摄取增加。

这些影像学特征结合临床表现和实验室检查，有助于诊断成人慢性非细菌性骨炎。

3成人慢性非细菌性骨炎最常见的表现是什么？


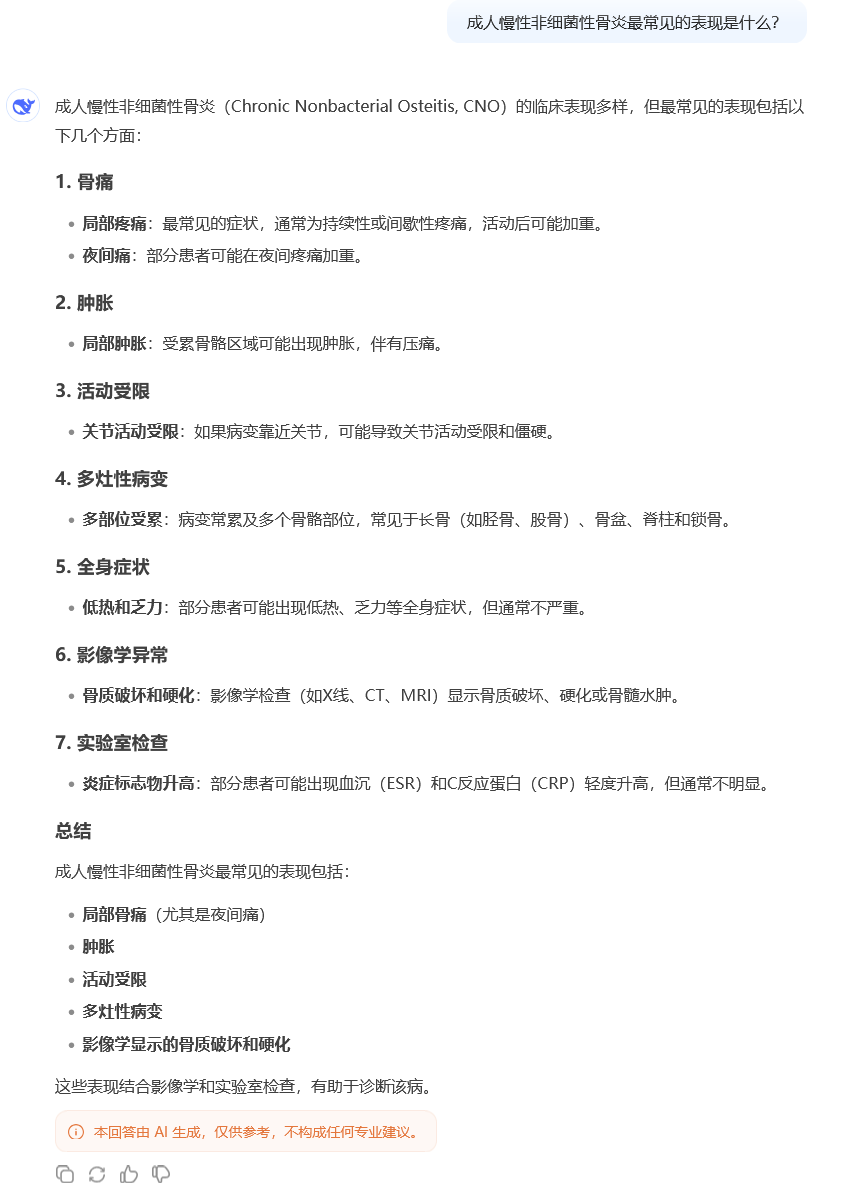
成人慢性非细菌性骨炎（Chronic Nonbacterial Osteitis, CNO）的临床表现多样，但最常见的表现包括以下几个方面：

### 1. **骨痛**

- **局部疼痛**：最常见的症状，通常为持续性或间歇性疼痛，活动后可能加重。

- **夜间痛**：部分患者可能在夜间疼痛加重。

### 2. **肿胀**

- **局部肿胀**：受累骨骼区域可能出现肿胀，伴有压痛。

### 3. **活动受限**

- **关节活动受限**：如果病变靠近关节，可能导致关节活动受限和僵硬。

### 4. **多灶性病变**

- **多部位受累**：病变常累及多个骨骼部位，常见于长骨（如胫骨、股骨）、骨盆、脊柱和锁骨。

### 5. **全身症状**

- **低热和乏力**：部分患者可能出现低热、乏力等全身症状，但通常不严重。

### 6. **影像学异常**

- **骨质破坏和硬化**：影像学检查（如X线、CT、MRI）显示骨质破坏、硬化或骨髓水肿。

### 7. **实验室检查**

- **炎症标志物升高**：部分患者可能出现血沉（ESR）和C反应蛋白（CRP）轻度升高，但通常不明显。

### 总结

成人慢性非细菌性骨炎最常见的表现包括：

- **局部骨痛**（尤其是夜间痛）

- **肿胀**

- **活动受限**

- **多灶性病变**

- **影像学显示的骨质破坏和硬化**

这些表现结合影像学和实验室检查，有助于诊断该病。

4成人慢性非细菌性骨炎最常受累的部位是那些？
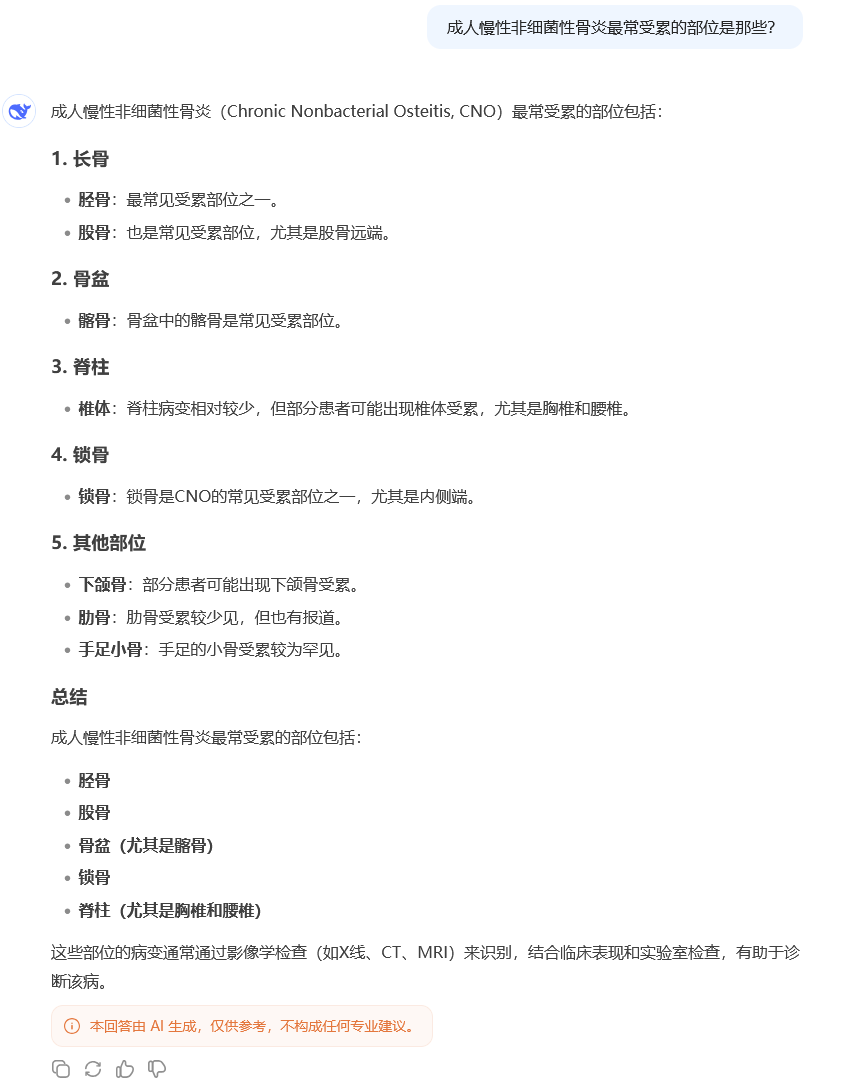
成人慢性非细菌性骨炎（Chronic Nonbacterial Osteitis, CNO）最常受累的部位包括：

### 1. **长骨**

- **胫骨**：最常见受累部位之一。

- **股骨**：也是常见受累部位，尤其是股骨远端。

### 2. **骨盆**

- **髂骨**：骨盆中的髂骨是常见受累部位。

### 3. **脊柱**

- **椎体**：脊柱病变相对较少，但部分患者可能出现椎体受累，尤其是胸椎和腰椎。

### 4. **锁骨**

- **锁骨**：锁骨是CNO的常见受累部位之一，尤其是内侧端。

### 5. **其他部位**

- **下颌骨**：部分患者可能出现下颌骨受累。

- **肋骨**：肋骨受累较少见，但也有报道。

- **手足小骨**：手足的小骨受累较为罕见。

### 总结

成人慢性非细菌性骨炎最常受累的部位包括：

- **胫骨**

- **股骨**

- **骨盆（尤其是髂骨）**

- **锁骨**

- **脊柱（尤其是胸椎和腰椎）**

这些部位的病变通常通过影像学检查（如X线、CT、MRI）来识别，结合临床表现和实验室检查，有助于诊断该病。

5成人慢性非细菌性骨炎有没有特异性的炎症标志物？
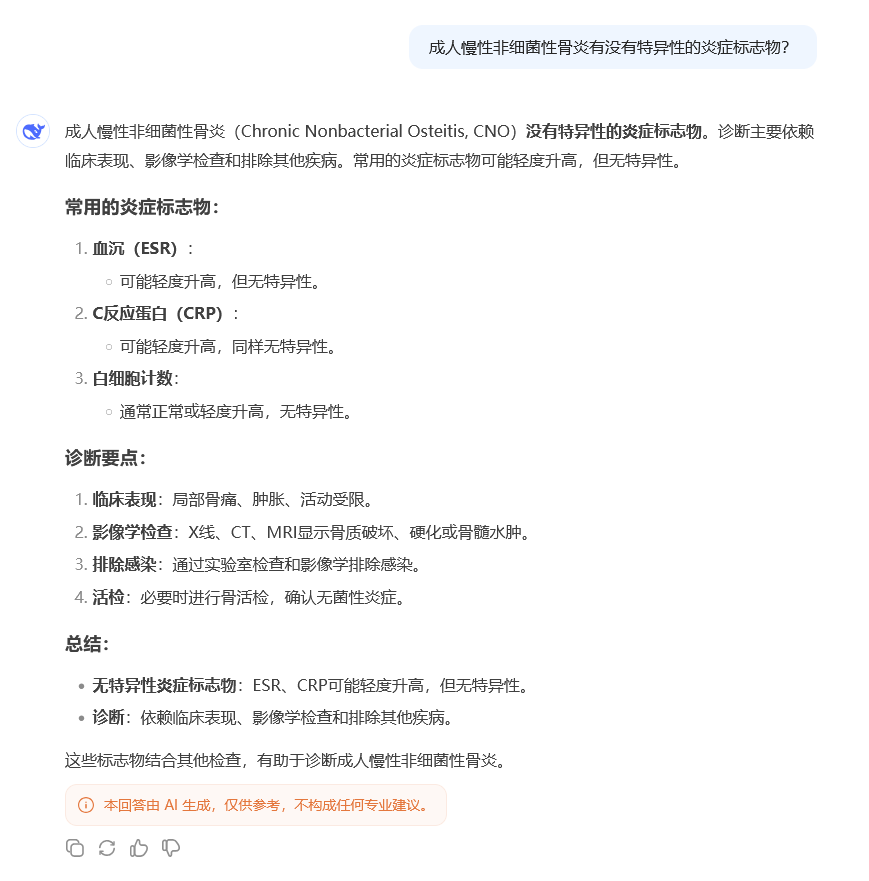
成人慢性非细菌性骨炎（Chronic Nonbacterial Osteitis, CNO）**没有特异性的炎症标志物**。诊断主要依赖临床表现、影像学检查和排除其他疾病。常用的炎症标志物可能轻度升高，但无特异性。

### 常用的炎症标志物：

1. **血沉（ESR）**：

- 可能轻度升高，但无特异性。

2. **C反应蛋白（CRP）**：

- 可能轻度升高，同样无特异性。

3. **白细胞计数**：

- 通常正常或轻度升高，无特异性。

### 诊断要点：

1. **临床表现**：局部骨痛、肿胀、活动受限。

2. **影像学检查**：X线、CT、MRI显示骨质破坏、硬化或骨髓水肿。

3. **排除感染**：通过实验室检查和影像学排除感染。

4. **活检**：必要时进行骨活检，确认无菌性炎症。

### 总结：

- **无特异性炎症标志物**：ESR、CRP可能轻度升高，但无特异性。

- **诊断**：依赖临床表现、影像学检查和排除其他疾病。

这些标志物结合其他检查，有助于诊断成人慢性非细菌性骨炎。

6慢性非细菌性骨炎首选的影像学检查是什么？


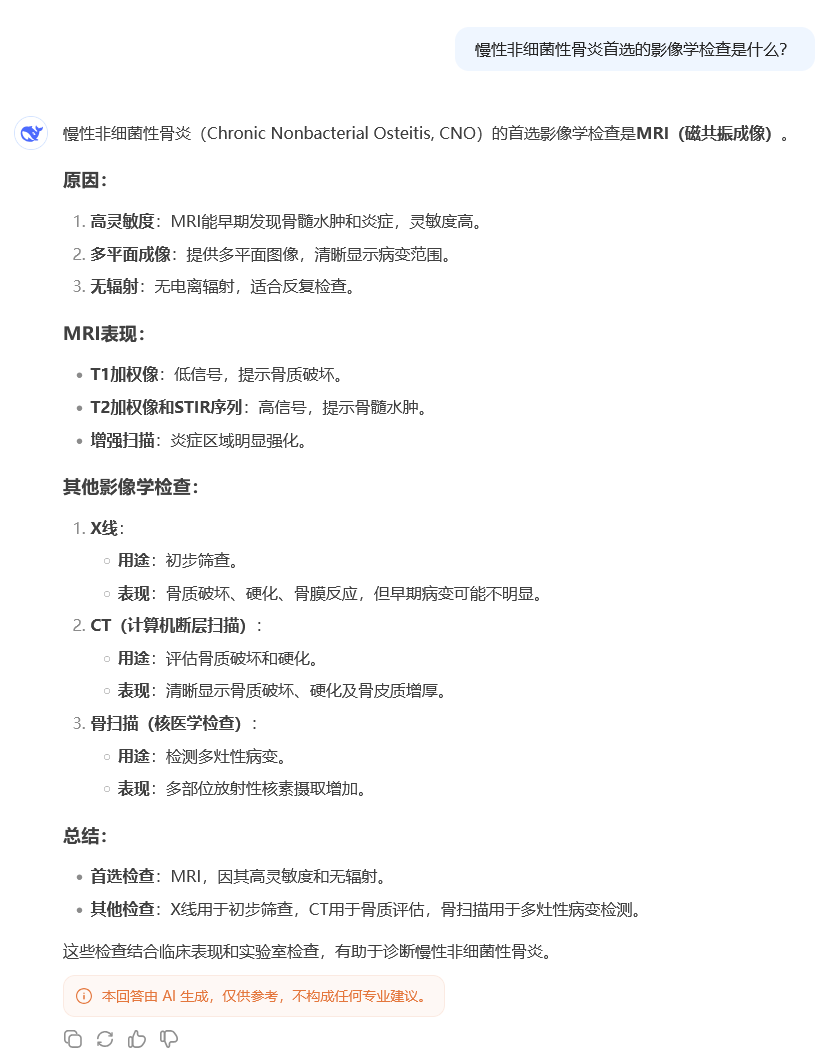
慢性非细菌性骨炎（Chronic Nonbacterial Osteitis, CNO）的首选影像学检查是**MRI（磁共振成像）**。

### 原因：

1. **高灵敏度**：MRI能早期发现骨髓水肿和炎症，灵敏度高。

2. **多平面成像**：提供多平面图像，清晰显示病变范围。

3. **无辐射**：无电离辐射，适合反复检查。

### MRI表现：

- **T1加权像**：低信号，提示骨质破坏。

- **T2加权像和STIR序列**：高信号，提示骨髓水肿。

- **增强扫描**：炎症区域明显强化。

### 其他影像学检查：

1. **X线**：

- **用途**：初步筛查。

- **表现**：骨质破坏、硬化、骨膜反应，但早期病变可能不明显。

2. **CT（计算机断层扫描）**：

- **用途**：评估骨质破坏和硬化。

- **表现**：清晰显示骨质破坏、硬化及骨皮质增厚。

3. **骨扫描（核医学检查）**：

- **用途**：检测多灶性病变。

- **表现**：多部位放射性核素摄取增加。

### 总结：

- **首选检查**：MRI，因其高灵敏度和无辐射。

- **其他检查**：X线用于初步筛查，CT用于骨质评估，骨扫描用于多灶性病变检测。

这些检查结合临床表现和实验室检查，有助于诊断慢性非细菌性骨炎。

7全身成像检查是不是诊断成人慢性非细菌性骨炎的先决条件？
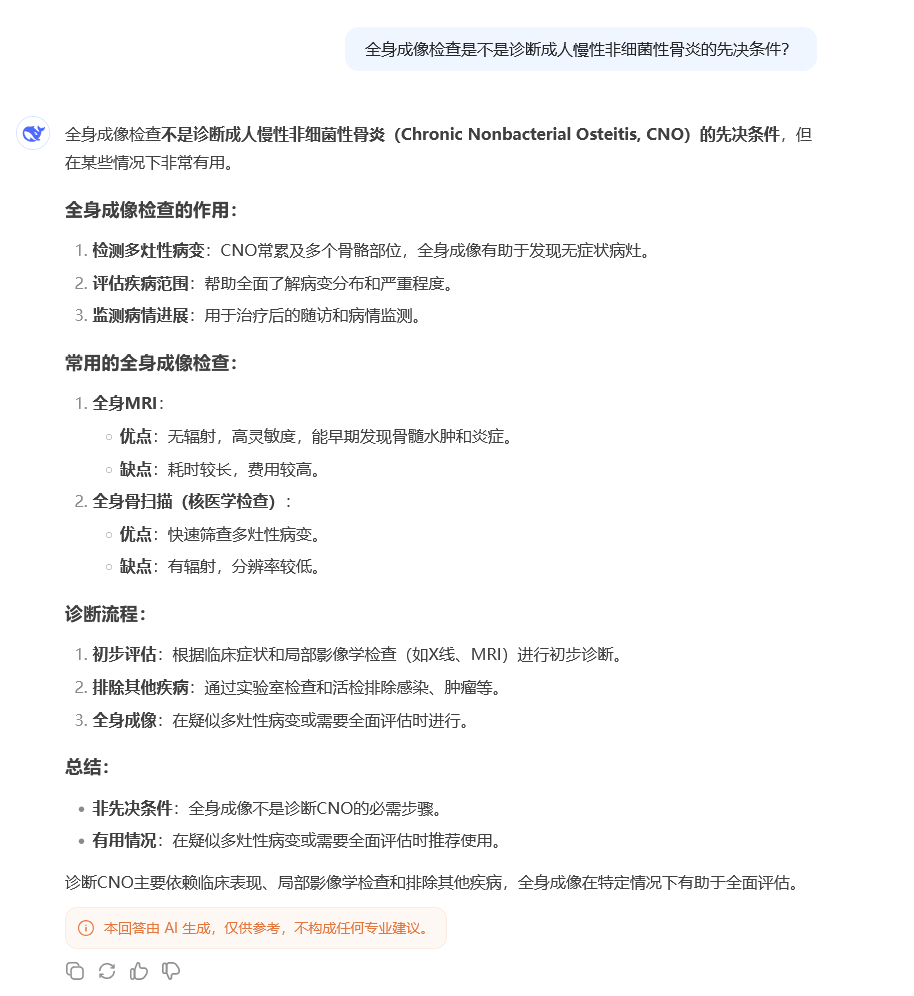
全身成像检查**不是诊断成人慢性非细菌性骨炎（Chronic Nonbacterial Osteitis, CNO）的先决条件**，但在某些情况下非常有用。

### 全身成像检查的作用：

1. **检测多灶性病变**：CNO常累及多个骨骼部位，全身成像有助于发现无症状病灶。

2. **评估疾病范围**：帮助全面了解病变分布和严重程度。

3. **监测病情进展**：用于治疗后的随访和病情监测。

### 常用的全身成像检查：

1. **全身MRI**：

- **优点**：无辐射，高灵敏度，能早期发现骨髓水肿和炎症。

- **缺点**：耗时较长，费用较高。

2. **全身骨扫描（核医学检查）**：

- **优点**：快速筛查多灶性病变。

- **缺点**：有辐射，分辨率较低。

### 诊断流程：

1. **初步评估**：根据临床症状和局部影像学检查（如X线、MRI）进行初步诊断。

2. **排除其他疾病**：通过实验室检查和活检排除感染、肿瘤等。

3. **全身成像**：在疑似多灶性病变或需要全面评估时进行。

### 总结：

- **非先决条件**：全身成像不是诊断CNO的必需步骤。

- **有用情况**：在疑似多灶性病变或需要全面评估时推荐使用。

诊断CNO主要依赖临床表现、局部影像学检查和排除其他疾病，全身成像在特定情况下有助于全面评估。

8诊断成人慢性非细菌性骨炎有必要常规骨活检吗？


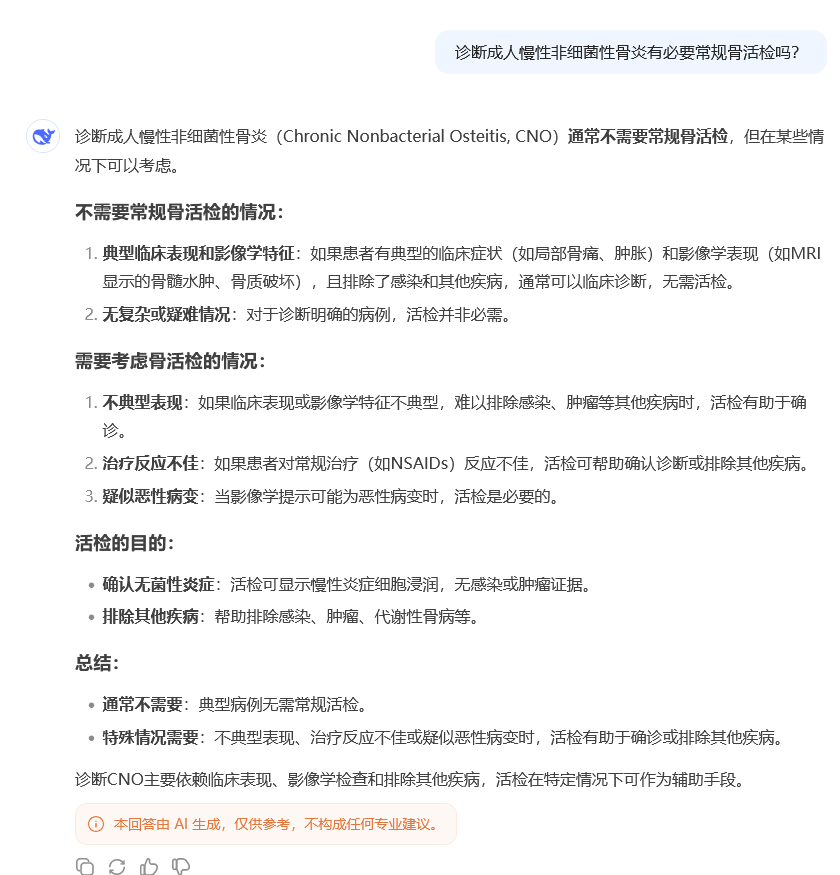
诊断成人慢性非细菌性骨炎（Chronic Nonbacterial Osteitis, CNO）**通常不需要常规骨活检**，但在某些情况下可以考虑。

### 不需要常规骨活检的情况：

1. **典型临床表现和影像学特征**：如果患者有典型的临床症状（如局部骨痛、肿胀）和影像学表现（如MRI显示的骨髓水肿、骨质破坏），且排除了感染和其他疾病，通常可以临床诊断，无需活检。

2. **无复杂或疑难情况**：对于诊断明确的病例，活检并非必需。

### 需要考虑骨活检的情况：

1. **不典型表现**：如果临床表现或影像学特征不典型，难以排除感染、肿瘤等其他疾病时，活检有助于确诊。

2. **治疗反应不佳**：如果患者对常规治疗（如NSAIDs）反应不佳，活检可帮助确认诊断或排除其他疾病。

3. **疑似恶性病变**：当影像学提示可能为恶性病变时，活检是必要的。

### 活检的目的：

- **确认无菌性炎症**：活检可显示慢性炎症细胞浸润，无感染或肿瘤证据。

- **排除其他疾病**：帮助排除感染、肿瘤、代谢性骨病等。

### 总结：

- **通常不需要**：典型病例无需常规活检。

- **特殊情况需要**：不典型表现、治疗反应不佳或疑似恶性病变时，活检有助于确诊或排除其他疾病。

诊断CNO主要依赖临床表现、影像学检查和排除其他疾病，活检在特定情况下可作为辅助手段。

9慢性非细菌性骨炎如何与恶性骨肿瘤鉴别？
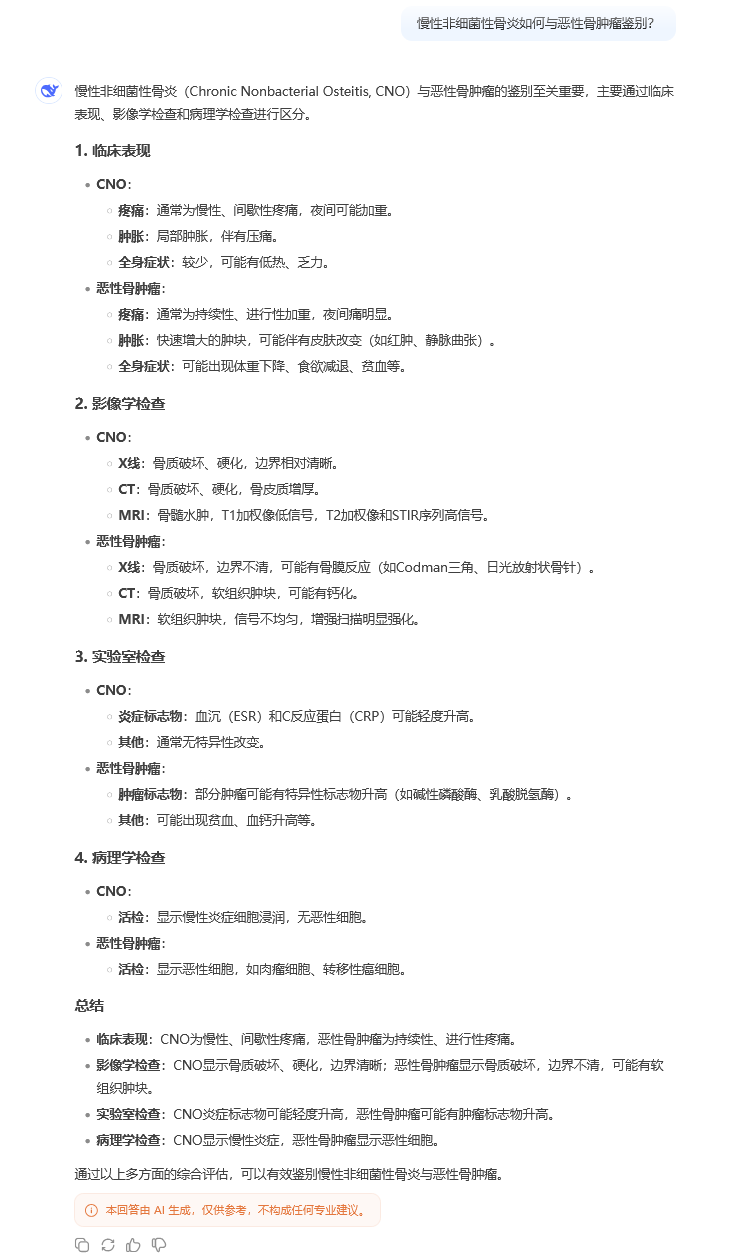
慢性非细菌性骨炎（Chronic Nonbacterial Osteitis, CNO）与恶性骨肿瘤的鉴别至关重要，主要通过临床表现、影像学检查和病理学检查进行区分。

### 1. **临床表现**

- **CNO**：

- **疼痛**：通常为慢性、间歇性疼痛，夜间可能加重。

- **肿胀**：局部肿胀，伴有压痛。

- **全身症状**：较少，可能有低热、乏力。

- **恶性骨肿瘤**：

- **疼痛**：通常为持续性、进行性加重，夜间痛明显。

- **肿胀**：快速增大的肿块，可能伴有皮肤改变（如红肿、静脉曲张）。

- **全身症状**：可能出现体重下降、食欲减退、贫血等。

### 2. **影像学检查**

- **CNO**：

- **X线**：骨质破坏、硬化，边界相对清晰。

- **CT**：骨质破坏、硬化，骨皮质增厚。

- **MRI**：骨髓水肿，T1加权像低信号，T2加权像和STIR序列高信号。

- **恶性骨肿瘤**：

- **X线**：骨质破坏，边界不清，可能有骨膜反应（如Codman三角、日光放射状骨针）。

- **CT**：骨质破坏，软组织肿块，可能有钙化。

- **MRI**：软组织肿块，信号不均匀，增强扫描明显强化。

### 3. **实验室检查**

- **CNO**：

- **炎症标志物**：血沉（ESR）和C反应蛋白（CRP）可能轻度升高。

- **其他**：通常无特异性改变。

- **恶性骨肿瘤**：

- **肿瘤标志物**：部分肿瘤可能有特异性标志物升高（如碱性磷酸酶、乳酸脱氢酶）。

- **其他**：可能出现贫血、血钙升高等。

### 4. **病理学检查**

- **CNO**：

- **活检**：显示慢性炎症细胞浸润，无恶性细胞。

- **恶性骨肿瘤**：

- **活检**：显示恶性细胞，如肉瘤细胞、转移性癌细胞。

### 总结

- **临床表现**：CNO为慢性、间歇性疼痛，恶性骨肿瘤为持续性、进行性疼痛。

- **影像学检查**：CNO显示骨质破坏、硬化，边界清晰；恶性骨肿瘤显示骨质破坏，边界不清，可能有软组织肿块。

- **实验室检查**：CNO炎症标志物可能轻度升高，恶性骨肿瘤可能有肿瘤标志物升高。

- **病理学检查**：CNO显示慢性炎症，恶性骨肿瘤显示恶性细胞。

通过以上多方面的综合评估，可以有效鉴别慢性非细菌性骨炎与恶性骨肿瘤。

10慢性非细菌性骨炎如何与骨关节炎鉴别？
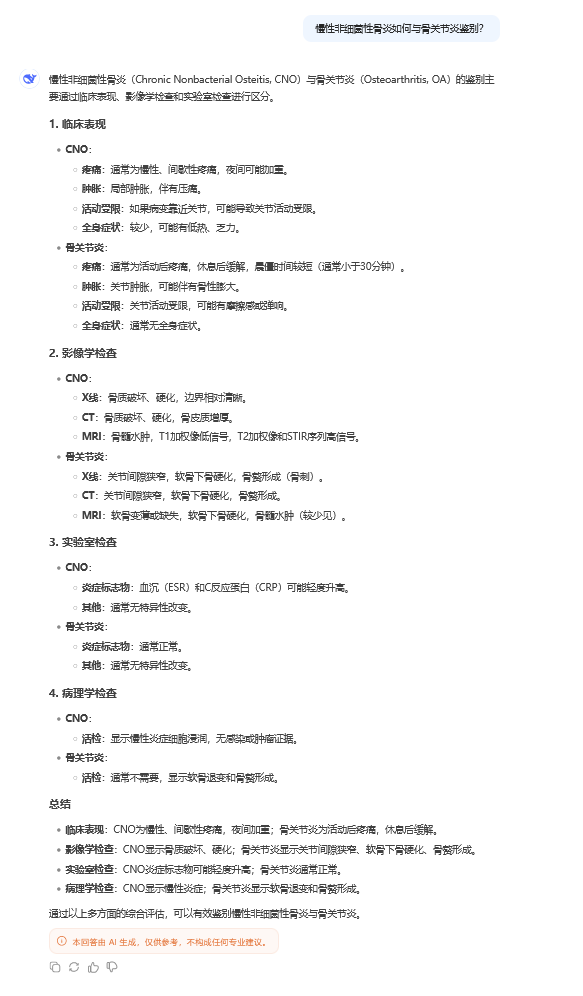
慢性非细菌性骨炎（Chronic Nonbacterial Osteitis, CNO）与骨关节炎（Osteoarthritis, OA）的鉴别主要通过临床表现、影像学检查和实验室检查进行区分。

### 1. **临床表现**

- **CNO**：

- **疼痛**：通常为慢性、间歇性疼痛，夜间可能加重。

- **肿胀**：局部肿胀，伴有压痛。

- **活动受限**：如果病变靠近关节，可能导致关节活动受限。

- **全身症状**：较少，可能有低热、乏力。

- **骨关节炎**：

- **疼痛**：通常为活动后疼痛，休息后缓解，晨僵时间较短（通常小于30分钟）。

- **肿胀**：关节肿胀，可能伴有骨性膨大。

- **活动受限**：关节活动受限，可能有摩擦感或弹响。

- **全身症状**：通常无全身症状。

### 2. **影像学检查**

- **CNO**：

- **X线**：骨质破坏、硬化，边界相对清晰。

- **CT**：骨质破坏、硬化，骨皮质增厚。

- **MRI**：骨髓水肿，T1加权像低信号，T2加权像和STIR序列高信号。

- **骨关节炎**：

- **X线**：关节间隙狭窄，软骨下骨硬化，骨赘形成（骨刺）。

- **CT**：关节间隙狭窄，软骨下骨硬化，骨赘形成。

- **MRI**：软骨变薄或缺失，软骨下骨硬化，骨髓水肿（较少见）。

### 3. **实验室检查**

- **CNO**：

- **炎症标志物**：血沉（ESR）和C反应蛋白（CRP）可能轻度升高。

- **其他**：通常无特异性改变。

- **骨关节炎**：

- **炎症标志物**：通常正常。

- **其他**：通常无特异性改变。

### 4. **病理学检查**

- **CNO**：

- **活检**：显示慢性炎症细胞浸润，无感染或肿瘤证据。

- **骨关节炎**：

- **活检**：通常不需要，显示软骨退变和骨赘形成。

### 总结

- **临床表现**：CNO为慢性、间歇性疼痛，夜间加重；骨关节炎为活动后疼痛，休息后缓解。

- **影像学检查**：CNO显示骨质破坏、硬化；骨关节炎显示关节间隙狭窄、软骨下骨硬化、骨赘形成。

- **实验室检查**：CNO炎症标志物可能轻度升高；骨关节炎通常正常。

- **病理学检查**：CNO显示慢性炎症；骨关节炎显示软骨退变和骨赘形成。

通过以上多方面的综合评估，可以有效鉴别慢性非细菌性骨炎与骨关节炎。

11慢性非细菌性骨炎如何与纤维发育不良鉴别？


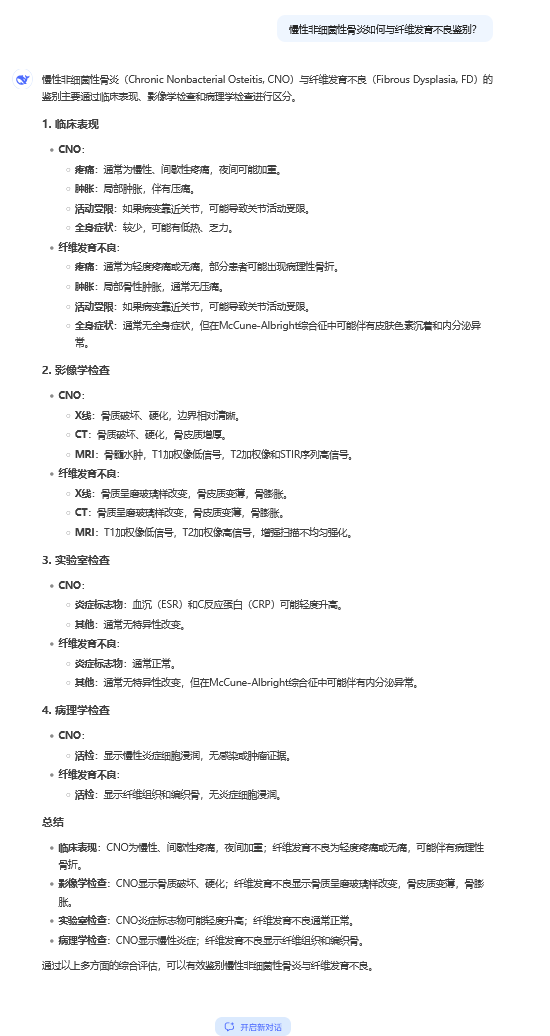
慢性非细菌性骨炎（Chronic Nonbacterial Osteitis, CNO）与纤维发育不良（Fibrous Dysplasia, FD）的鉴别主要通过临床表现、影像学检查和病理学检查进行区分。

### 1. **临床表现**

- **CNO**：

- **疼痛**：通常为慢性、间歇性疼痛，夜间可能加重。

- **肿胀**：局部肿胀，伴有压痛。

- **活动受限**：如果病变靠近关节，可能导致关节活动受限。

- **全身症状**：较少，可能有低热、乏力。

- **纤维发育不良**：

- **疼痛**：通常为轻度疼痛或无痛，部分患者可能出现病理性骨折。

- **肿胀**：局部骨性肿胀，通常无压痛。

- **活动受限**：如果病变靠近关节，可能导致关节活动受限。

- **全身症状**：通常无全身症状，但在McCune-Albright综合征中可能伴有皮肤色素沉着和内分泌异常。

### 2. **影像学检查**

- **CNO**：

- **X线**：骨质破坏、硬化，边界相对清晰。

- **CT**：骨质破坏、硬化，骨皮质增厚。

- **MRI**：骨髓水肿，T1加权像低信号，T2加权像和STIR序列高信号。

- **纤维发育不良**：

- **X线**：骨质呈磨玻璃样改变，骨皮质变薄，骨膨胀。

- **CT**：骨质呈磨玻璃样改变，骨皮质变薄，骨膨胀。

- **MRI**：T1加权像低信号，T2加权像高信号，增强扫描不均匀强化。

### 3. **实验室检查**

- **CNO**：

- **炎症标志物**：血沉（ESR）和C反应蛋白（CRP）可能轻度升高。

- **其他**：通常无特异性改变。

- **纤维发育不良**：

- **炎症标志物**：通常正常。

- **其他**：通常无特异性改变，但在McCune-Albright综合征中可能伴有内分泌异常。

### 4. **病理学检查**

- **CNO**：

- **活检**：显示慢性炎症细胞浸润，无感染或肿瘤证据。

- **纤维发育不良**：

- **活检**：显示纤维组织和编织骨，无炎症细胞浸润。

### 总结

- **临床表现**：CNO为慢性、间歇性疼痛，夜间加重；纤维发育不良为轻度疼痛或无痛，可能伴有病理性骨折。

- **影像学检查**：CNO显示骨质破坏、硬化；纤维发育不良显示骨质呈磨玻璃样改变，骨皮质变薄，骨膨胀。

- **实验室检查**：CNO炎症标志物可能轻度升高；纤维发育不良通常正常。

- **病理学检查**：CNO显示慢性炎症；纤维发育不良显示纤维组织和编织骨。

通过以上多方面的综合评估，可以有效鉴别慢性非细菌性骨炎与纤维发育不良。

12在治慢性非细菌性骨炎过程中应该选择短疗程的糖皮质激素治疗还是长期的糖皮质激素治疗？


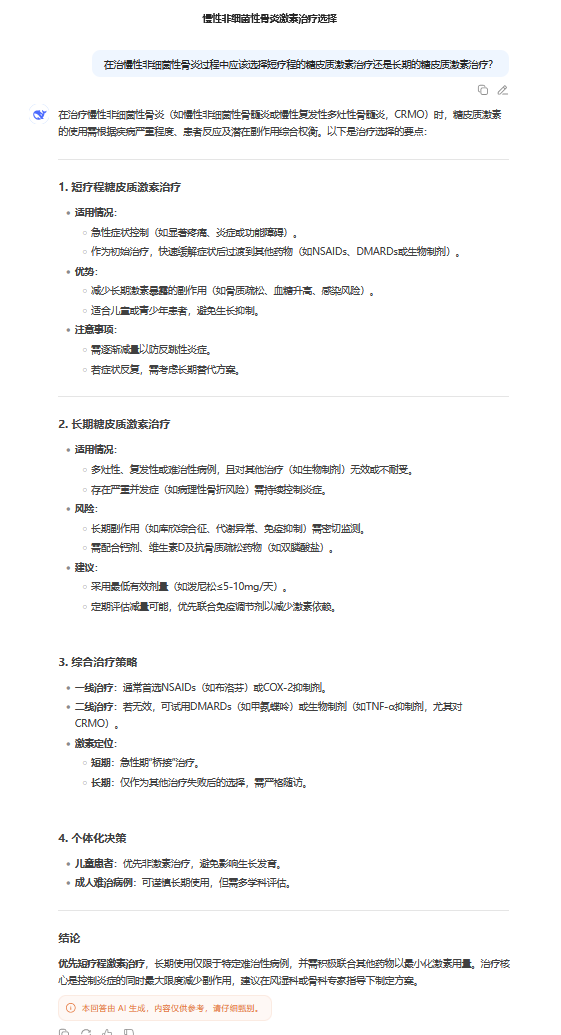
在治疗慢性非细菌性骨炎（如慢性非细菌性骨髓炎或慢性复发性多灶性骨髓炎，CRMO）时，糖皮质激素的使用需根据疾病严重程度、患者反应及潜在副作用综合权衡。以下是治疗选择的要点：

---

### **1. 短疗程糖皮质激素治疗**

- **适用情况**：

- 急性症状控制（如显著疼痛、炎症或功能障碍）。

- 作为初始治疗，快速缓解症状后过渡到其他药物（如NSAIDs、DMARDs或生物制剂）。

- **优势**：

- 减少长期激素暴露的副作用（如骨质疏松、血糖升高、感染风险）。

- 适合儿童或青少年患者，避免生长抑制。

- **注意事项**：

- 需逐渐减量以防反跳性炎症。

- 若症状反复，需考虑长期替代方案。

---

### **2. 长期糖皮质激素治疗**

- **适用情况**：

- 多灶性、复发性或难治性病例，且对其他治疗（如生物制剂）无效或不耐受。

- 存在严重并发症（如病理性骨折风险）需持续控制炎症。

- **风险**：

- 长期副作用（如库欣综合征、代谢异常、免疫抑制）需密切监测。

- 需配合钙剂、维生素D及抗骨质疏松药物（如双膦酸盐）。

- **建议**：

- 采用最低有效剂量（如泼尼松≤5-10mg/天）。

- 定期评估减量可能，优先联合免疫调节剂以减少激素依赖。

---

### **3. 综合治疗策略**

- **一线治疗**：通常首选NSAIDs（如布洛芬）或COX-2抑制剂。

- **二线治疗**：若无效，可试用DMARDs（如甲氨蝶呤）或生物制剂（如TNF-α抑制剂，尤其对CRMO）。

- **激素定位**：

- **短期**：急性期“桥接”治疗。

- **长期**：仅作为其他治疗失败后的选择，需严格随访。

---

### **4. 个体化决策**

- **儿童患者**：优先非激素治疗，避免影响生长发育。

- **成人难治病例**：可谨慎长期使用，但需多学科评估。

---

### **结论**

**优先短疗程激素治疗**，长期使用仅限于特定难治性病例，并需积极联合其他药物以最小化激素用量。治疗核心是控制炎症的同时最大限度减少副作用，建议在风湿科或骨科专家指导下制定方案。

13成人慢性非细菌性骨炎一线治疗方案是什么？


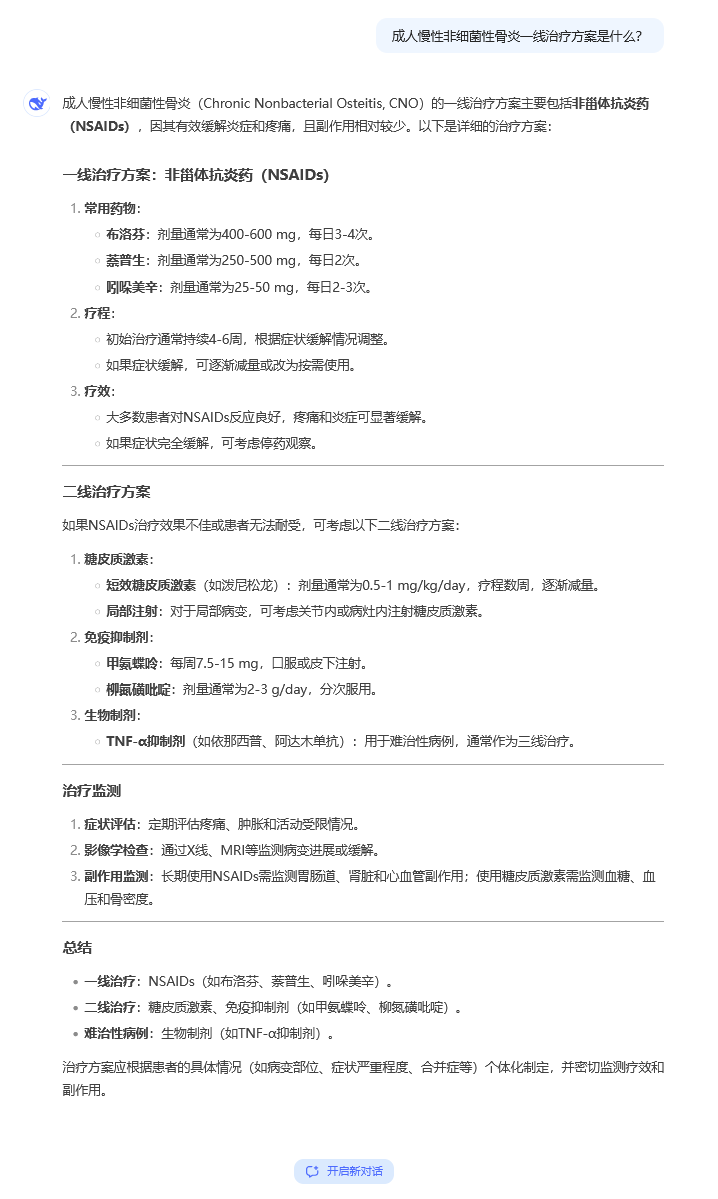


成人慢性非细菌性骨炎（Chronic Nonbacterial Osteitis, CNO）的一线治疗方案主要包括**非甾体抗炎药（NSAIDs）**，因其有效缓解炎症和疼痛，且副作用相对较少。以下是详细的治疗方案：

---

### **一线治疗方案：非甾体抗炎药（NSAIDs）**

1. **常用药物**：

- **布洛芬**：剂量通常为400-600 mg，每日3-4次。

- **萘普生**：剂量通常为250-500 mg，每日2次。

- **吲哚美辛**：剂量通常为25-50 mg，每日2-3次。

2. **疗程**：

- 初始治疗通常持续4-6周，根据症状缓解情况调整。

- 如果症状缓解，可逐渐减量或改为按需使用。

3. **疗效**：

- 大多数患者对NSAIDs反应良好，疼痛和炎症可显著缓解。

- 如果症状完全缓解，可考虑停药观察。

---

### **二线治疗方案**

如果NSAIDs治疗效果不佳或患者无法耐受，可考虑以下二线治疗方案：

1. **糖皮质激素**：

- **短效糖皮质激素**（如泼尼松龙）：剂量通常为0.5-1 mg/kg/day，疗程数周，逐渐减量。

- **局部注射**：对于局部病变，可考虑关节内或病灶内注射糖皮质激素。

2. **免疫抑制剂**：

- **甲氨蝶呤**：每周7.5-15 mg，口服或皮下注射。

- **柳氮磺吡啶**：剂量通常为2-3 g/day，分次服用。

3. **生物制剂**：

- **TNF-α抑制剂**（如依那西普、阿达木单抗）：用于难治性病例，通常作为三线治疗。

---

### **治疗监测**

1. **症状评估**：定期评估疼痛、肿胀和活动受限情况。

2. **影像学检查**：通过X线、MRI等监测病变进展或缓解。

3. **副作用监测**：长期使用NSAIDs需监测胃肠道、肾脏和心血管副作用；使用糖皮质激素需监测血糖、血压和骨密度。

---

### **总结**

- **一线治疗**：NSAIDs（如布洛芬、萘普生、吲哚美辛）。

- **二线治疗**：糖皮质激素、免疫抑制剂（如甲氨蝶呤、柳氮磺吡啶）。

- **难治性病例**：生物制剂（如TNF-α抑制剂）。

治疗方案应根据患者的具体情况（如病变部位、症状严重程度、合并症等）个体化制定，并密切监测疗效和副作用。

14无临床症状，也没有放射学疾病活动的慢性非细菌性骨炎的病人需要治疗吗？


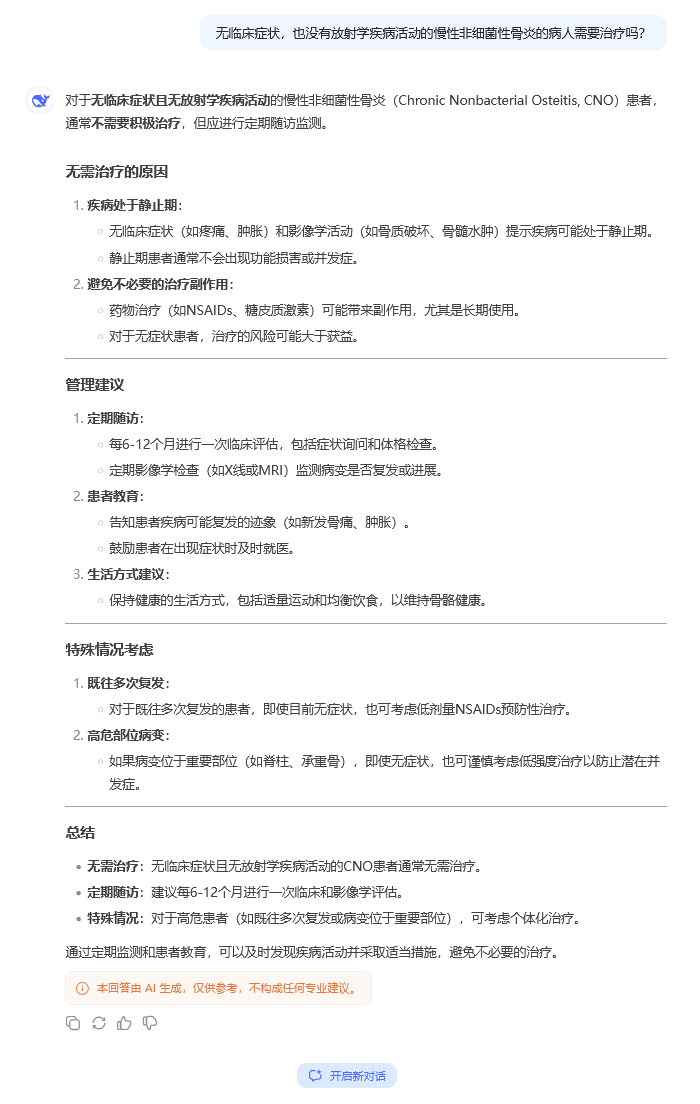
对于**无临床症状且无放射学疾病活动**的慢性非细菌性骨炎（Chronic Nonbacterial Osteitis, CNO）患者，通常**不需要积极治疗**，但应进行定期随访监测。

---

### **无需治疗的原因**

1. **疾病处于静止期**：

- 无临床症状（如疼痛、肿胀）和影像学活动（如骨质破坏、骨髓水肿）提示疾病可能处于静止期。

- 静止期患者通常不会出现功能损害或并发症。

2. **避免不必要的治疗副作用**：

- 药物治疗（如NSAIDs、糖皮质激素）可能带来副作用，尤其是长期使用。

- 对于无症状患者，治疗的风险可能大于获益。

---

### **管理建议**

1. **定期随访**：

- 每6-12个月进行一次临床评估，包括症状询问和体格检查。

- 定期影像学检查（如X线或MRI）监测病变是否复发或进展。

2. **患者教育**：

- 告知患者疾病可能复发的迹象（如新发骨痛、肿胀）。

- 鼓励患者在出现症状时及时就医。

3. **生活方式建议**：

- 保持健康的生活方式，包括适量运动和均衡饮食，以维持骨骼健康。

---

### **特殊情况考虑**

1. **既往多次复发**：

- 对于既往多次复发的患者，即使目前无症状，也可考虑低剂量NSAIDs预防性治疗。

2. **高危部位病变**：

- 如果病变位于重要部位（如脊柱、承重骨），即使无症状，也可谨慎考虑低强度治疗以防止潜在并发症。

---

### **总结**

- **无需治疗**：无临床症状且无放射学疾病活动的CNO患者通常无需治疗。

- **定期随访**：建议每6-12个月进行一次临床和影像学评估。

- **特殊情况**：对于高危患者（如既往多次复发或病变位于重要部位），可考虑个体化治疗。

通过定期监测和患者教育，可以及时发现疾病活动并采取适当措施，避免不必要的治疗。

15有临床症状没有放射学疾病活动的慢性非细菌性骨炎的病人如何处理？


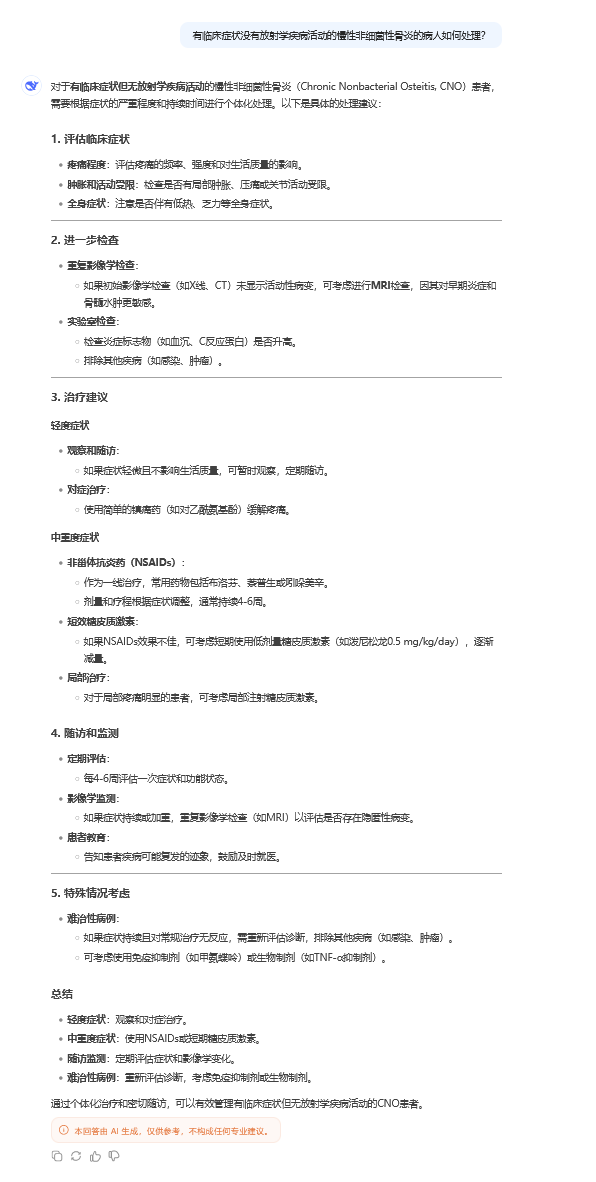
对于**有临床症状但无放射学疾病活动**的慢性非细菌性骨炎（Chronic Nonbacterial Osteitis, CNO）患者，需要根据症状的严重程度和持续时间进行个体化处理。以下是具体的处理建议：

---

### **1. 评估临床症状**

- **疼痛程度**：评估疼痛的频率、强度和对生活质量的影响。

- **肿胀和活动受限**：检查是否有局部肿胀、压痛或关节活动受限。

- **全身症状**：注意是否伴有低热、乏力等全身症状。

---

### **2. 进一步检查**

- **重复影像学检查**：

- 如果初始影像学检查（如X线、CT）未显示活动性病变，可考虑进行**MRI**检查，因其对早期炎症和骨髓水肿更敏感。

- **实验室检查**：

- 检查炎症标志物（如血沉、C反应蛋白）是否升高。

- 排除其他疾病（如感染、肿瘤）。

---

### **3. 治疗建议**

#### **轻度症状**

- **观察和随访**：

- 如果症状轻微且不影响生活质量，可暂时观察，定期随访。

- **对症治疗**：

- 使用简单的镇痛药（如对乙酰氨基酚）缓解疼痛。

#### **中重度症状**

- **非甾体抗炎药（NSAIDs）**：

- 作为一线治疗，常用药物包括布洛芬、萘普生或吲哚美辛。

- 剂量和疗程根据症状调整，通常持续4-6周。

- **短效糖皮质激素**：

- 如果NSAIDs效果不佳，可考虑短期使用低剂量糖皮质激素（如泼尼松龙0.5 mg/kg/day），逐渐减量。

- **局部治疗**：

- 对于局部疼痛明显的患者，可考虑局部注射糖皮质激素。

---

### **4. 随访和监测**

- **定期评估**：

- 每4-6周评估一次症状和功能状态。

- **影像学监测**：

- 如果症状持续或加重，重复影像学检查（如MRI）以评估是否存在隐匿性病变。

- **患者教育**：

- 告知患者疾病可能复发的迹象，鼓励及时就医。

---

### **5. 特殊情况考虑**

- **难治性病例**：

- 如果症状持续且对常规治疗无反应，需重新评估诊断，排除其他疾病（如感染、肿瘤）。

- 可考虑使用免疫抑制剂（如甲氨蝶呤）或生物制剂（如TNF-α抑制剂）。

---

### **总结**

- **轻度症状**：观察和对症治疗。

- **中重度症状**：使用NSAIDs或短期糖皮质激素。

- **随访监测**：定期评估症状和影像学变化。

- **难治性病例**：重新评估诊断，考虑免疫抑制剂或生物制剂。

通过个体化治疗和密切随访，可以有效管理有临床症状但无放射学疾病活动的CNO患者。

16有椎体塌陷风险的脊柱骨病变的慢性非细菌性骨炎患者和出现明显骨骼累积损伤的慢性非细菌性骨炎患者如何治疗？


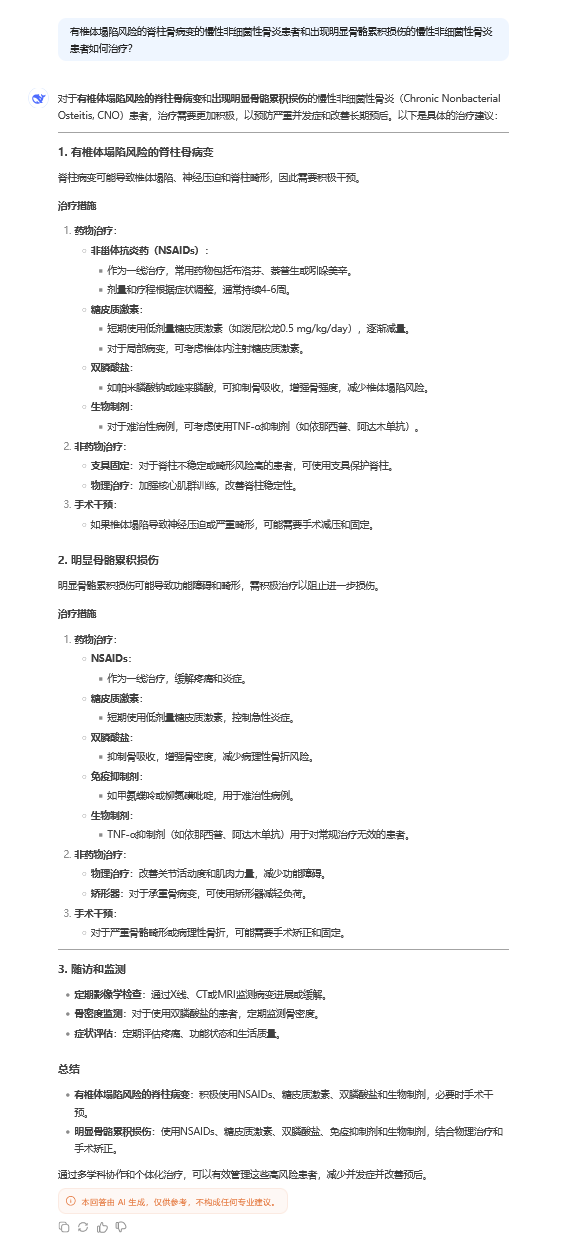
对于**有椎体塌陷风险的脊柱骨病变**和**出现明显骨骼累积损伤**的慢性非细菌性骨炎（Chronic Nonbacterial Osteitis, CNO）患者，治疗需要更加积极，以预防严重并发症和改善长期预后。以下是具体的治疗建议：

---

### **1. 有椎体塌陷风险的脊柱骨病变**

脊柱病变可能导致椎体塌陷、神经压迫和脊柱畸形，因此需要积极干预。

#### **治疗措施**

1. **药物治疗**：

- **非甾体抗炎药（NSAIDs）**：

- 作为一线治疗，常用药物包括布洛芬、萘普生或吲哚美辛。

- 剂量和疗程根据症状调整，通常持续4-6周。

- **糖皮质激素**：

- 短期使用低剂量糖皮质激素（如泼尼松龙0.5 mg/kg/day），逐渐减量。

- 对于局部病变，可考虑椎体内注射糖皮质激素。

- **双膦酸盐**：

- 如帕米膦酸钠或唑来膦酸，可抑制骨吸收，增强骨强度，减少椎体塌陷风险。

- **生物制剂**：

- 对于难治性病例，可考虑使用TNF-α抑制剂（如依那西普、阿达木单抗）。

2. **非药物治疗**：

- **支具固定**：对于脊柱不稳定或畸形风险高的患者，可使用支具保护脊柱。

- **物理治疗**：加强核心肌群训练，改善脊柱稳定性。

3. **手术干预**：

- 如果椎体塌陷导致神经压迫或严重畸形，可能需要手术减压和固定。

---

### **2. 明显骨骼累积损伤**

明显骨骼累积损伤可能导致功能障碍和畸形，需积极治疗以阻止进一步损伤。

#### **治疗措施**

1. **药物治疗**：

- **NSAIDs**：

- 作为一线治疗，缓解疼痛和炎症。

- **糖皮质激素**：

- 短期使用低剂量糖皮质激素，控制急性炎症。

- **双膦酸盐**：

- 抑制骨吸收，增强骨密度，减少病理性骨折风险。

- **免疫抑制剂**：

- 如甲氨蝶呤或柳氮磺吡啶，用于难治性病例。

- **生物制剂**：

- TNF-α抑制剂（如依那西普、阿达木单抗）用于对常规治疗无效的患者。

2. **非药物治疗**：

- **物理治疗**：改善关节活动度和肌肉力量，减少功能障碍。

- **矫形器**：对于承重骨病变，可使用矫形器减轻负荷。

3. **手术干预**：

- 对于严重骨骼畸形或病理性骨折，可能需要手术矫正和固定。

---

### **3. 随访和监测**

- **定期影像学检查**：通过X线、CT或MRI监测病变进展或缓解。

- **骨密度监测**：对于使用双膦酸盐的患者，定期监测骨密度。

- **症状评估**：定期评估疼痛、功能状态和生活质量。

---

### **总结**

- **有椎体塌陷风险的脊柱病变**：积极使用NSAIDs、糖皮质激素、双膦酸盐和生物制剂，必要时手术干预。

- **明显骨骼累积损伤**：使用NSAIDs、糖皮质激素、双膦酸盐、免疫抑制剂和生物制剂，结合物理治疗和手术矫正。

通过多学科协作和个体化治疗，可以有效管理这些高风险患者，减少并发症并改善预后。
