# Supplementary material for: Evaluation of the accuracy and repeatability of Deepseek V3, Doubao, and Kimi1.5 in answering knowledge-related queries about chronic non-bacterial osteitis
Source: Front Artif Intell. 2025 Sep 29;8:1629149. doi: 10.3389/frai.2025.1629149 (PMC12515971; doi:10.3389/frai.2025.1629149)
Supplement: Supplementary file 1 [file Data_Sheet_1.zip › day14/Day14-doubao.docx]

1成人慢性非细菌性骨炎的定义是什么？


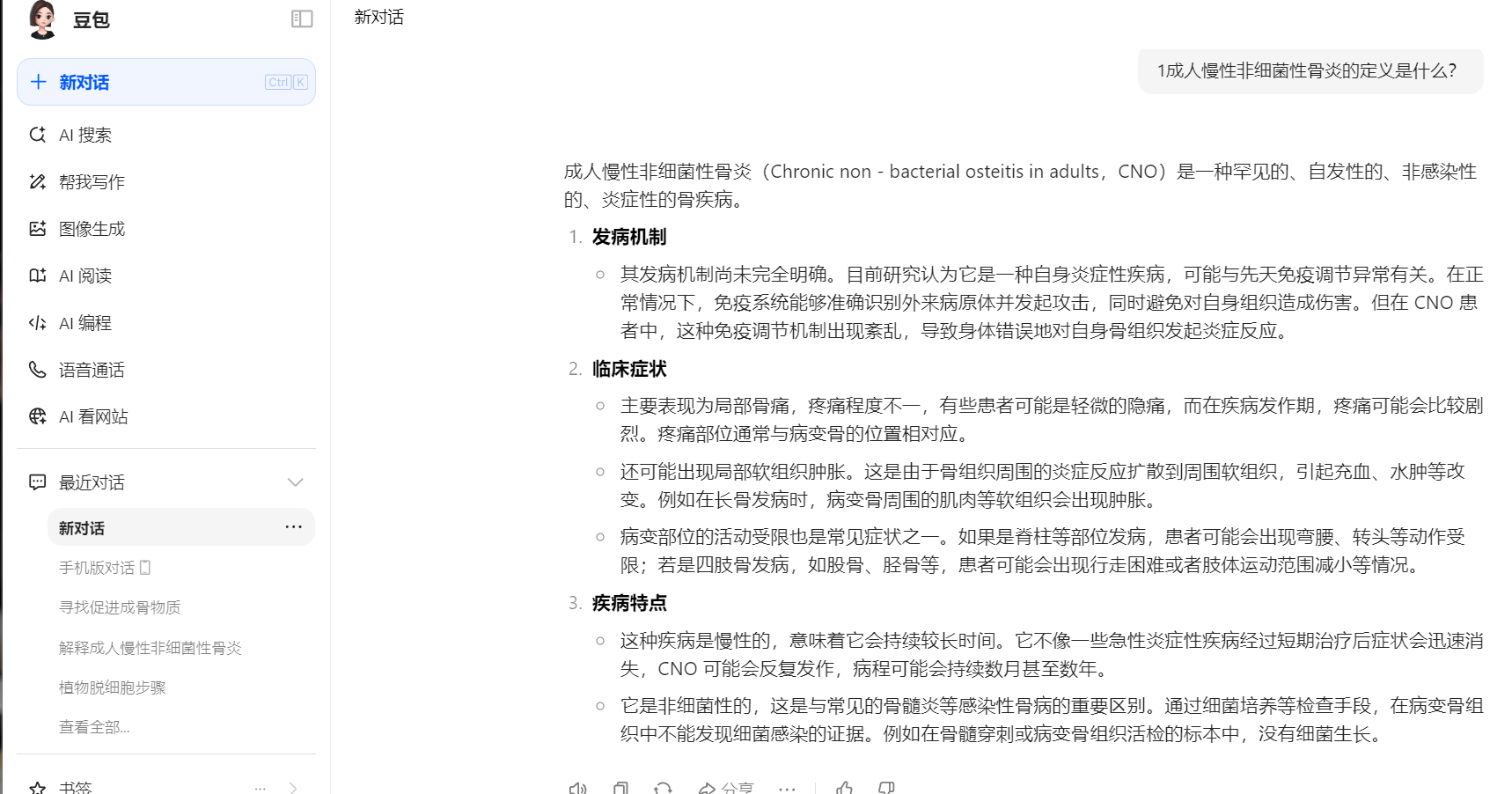


1. **发病机制**

- 其发病机制尚未完全明确。目前研究认为它是一种自身炎症性疾病，可能与先天免疫调节异常有关。在正常情况下，免疫系统能够准确识别外来病原体并发起攻击，同时避免对自身组织造成伤害。但在CNO患者中，这种免疫调节机制出现紊乱，导致身体错误地对自身骨组织发起炎症反应。

2. **临床症状**

- 主要表现为局部骨痛，疼痛程度不一，有些患者可能是轻微的隐痛，而在疾病发作期，疼痛可能会比较剧烈。疼痛部位通常与病变骨的位置相对应。

- 还可能出现局部软组织肿胀。这是由于骨组织周围的炎症反应扩散到周围软组织，引起充血、水肿等改变。例如在长骨发病时，病变骨周围的肌肉等软组织会出现肿胀。

- 病变部位的活动受限也是常见症状之一。如果是脊柱等部位发病，患者可能会出现弯腰、转头等动作受限；若是四肢骨发病，如股骨、胫骨等，患者可能会出现行走困难或者肢体运动范围减小等情况。

3. **疾病特点**

- 这种疾病是慢性的，意味着它会持续较长时间。它不像一些急性炎症性疾病经过短期治疗后症状会迅速消失，CNO可能会反复发作，病程可能会持续数月甚至数年。

- 它是非细菌性的，这是与常见的骨髓炎等感染性骨病的重要区别。通过细菌培养等检查手段，在病变骨组织中不能发现细菌感染的证据。例如在骨髓穿刺或病变骨组织活检的标本中，没有细菌生长。

2成人慢性非细菌性骨炎的典型影像学特征是什么？


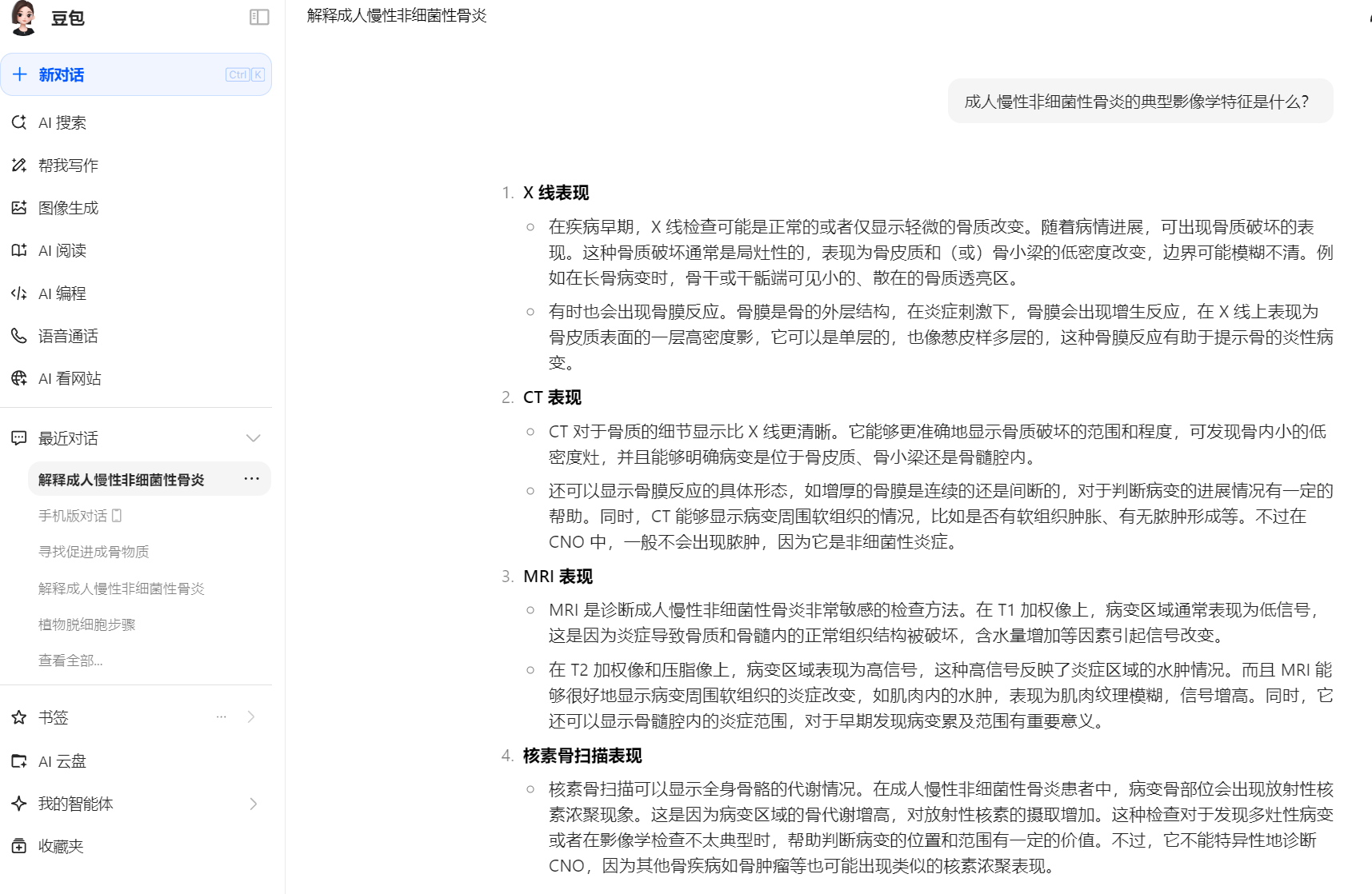
1. **X线表现**

- 在疾病早期，X线检查可能是正常的或者仅显示轻微的骨质改变。随着病情进展，可出现骨质破坏的表现。这种骨质破坏通常是局灶性的，表现为骨皮质和（或）骨小梁的低密度改变，边界可能模糊不清。例如在长骨病变时，骨干或干骺端可见小的、散在的骨质透亮区。

- 有时也会出现骨膜反应。骨膜是骨的外层结构，在炎症刺激下，骨膜会出现增生反应，在X线上表现为骨皮质表面的一层高密度影，它可以是单层的，也像葱皮样多层的，这种骨膜反应有助于提示骨的炎性病变。

2. **CT表现**

- CT对于骨质的细节显示比X线更清晰。它能够更准确地显示骨质破坏的范围和程度，可发现骨内小的低密度灶，并且能够明确病变是位于骨皮质、骨小梁还是骨髓腔内。

- 还可以显示骨膜反应的具体形态，如增厚的骨膜是连续的还是间断的，对于判断病变的进展情况有一定的帮助。同时，CT能够显示病变周围软组织的情况，比如是否有软组织肿胀、有无脓肿形成等。不过在CNO中，一般不会出现脓肿，因为它是非细菌性炎症。

3. **MRI表现**

- MRI是诊断成人慢性非细菌性骨炎非常敏感的检查方法。在T1加权像上，病变区域通常表现为低信号，这是因为炎症导致骨质和骨髓内的正常组织结构被破坏，含水量增加等因素引起信号改变。

- 在T2加权像和压脂像上，病变区域表现为高信号，这种高信号反映了炎症区域的水肿情况。而且MRI能够很好地显示病变周围软组织的炎症改变，如肌肉内的水肿，表现为肌肉纹理模糊，信号增高。同时，它还可以显示骨髓腔内的炎症范围，对于早期发现病变累及范围有重要意义。

4. **核素骨扫描表现**

- 核素骨扫描可以显示全身骨骼的代谢情况。在成人慢性非细菌性骨炎患者中，病变骨部位会出现放射性核素浓聚现象。这是因为病变区域的骨代谢增高，对放射性核素的摄取增加。这种检查对于发现多灶性病变或者在影像学检查不太典型时，帮助判断病变的位置和范围有一定的价值。不过，它不能特异性地诊断CNO，因为其他骨疾病如骨肿瘤等也可能出现类似的核素浓聚表现。

3成人慢性非细菌性骨炎最常见的表现是什么？


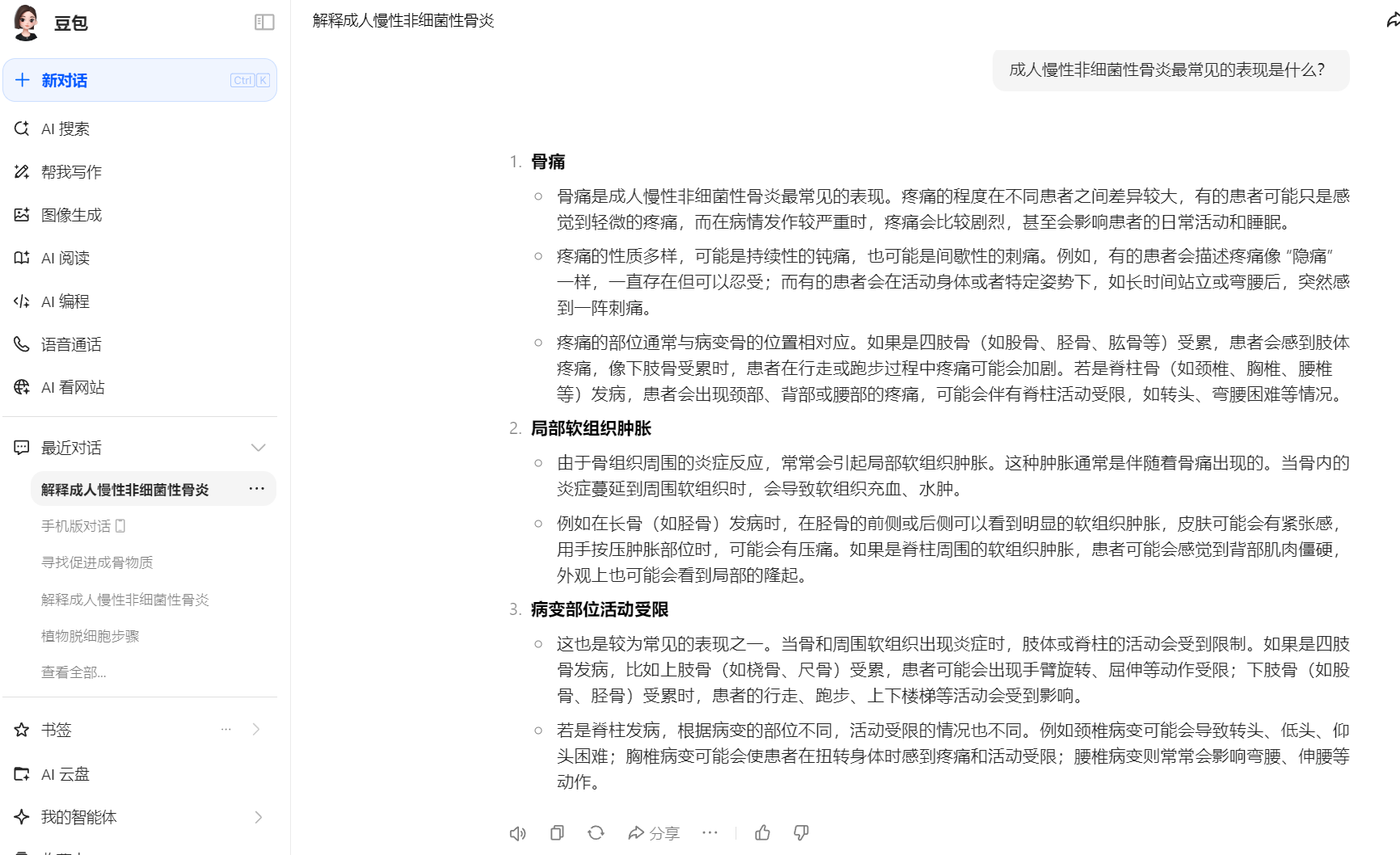
 1. **骨痛**

- 骨痛是成人慢性非细菌性骨炎最常见的表现。疼痛的程度在不同患者之间差异较大，有的患者可能只是感觉到轻微的疼痛，而在病情发作较严重时，疼痛会比较剧烈，甚至会影响患者的日常活动和睡眠。

- 疼痛的性质多样，可能是持续性的钝痛，也可能是间歇性的刺痛。例如，有的患者会描述疼痛像“隐痛”一样，一直存在但可以忍受；而有的患者会在活动身体或者特定姿势下，如长时间站立或弯腰后，突然感到一阵刺痛。

- 疼痛的部位通常与病变骨的位置相对应。如果是四肢骨（如股骨、胫骨、肱骨等）受累，患者会感到肢体疼痛，像下肢骨受累时，患者在行走或跑步过程中疼痛可能会加剧。若是脊柱骨（如颈椎、胸椎、腰椎等）发病，患者会出现颈部、背部或腰部的疼痛，可能会伴有脊柱活动受限，如转头、弯腰困难等情况。

2. **局部软组织肿胀**

- 由于骨组织周围的炎症反应，常常会引起局部软组织肿胀。这种肿胀通常是伴随着骨痛出现的。当骨内的炎症蔓延到周围软组织时，会导致软组织充血、水肿。

- 例如在长骨（如胫骨）发病时，在胫骨的前侧或后侧可以看到明显的软组织肿胀，皮肤可能会有紧张感，用手按压肿胀部位时，可能会有压痛。如果是脊柱周围的软组织肿胀，患者可能会感觉到背部肌肉僵硬，外观上也可能会看到局部的隆起。

3. **病变部位活动受限**

- 这也是较为常见的表现之一。当骨和周围软组织出现炎症时，肢体或脊柱的活动会受到限制。如果是四肢骨发病，比如上肢骨（如桡骨、尺骨）受累，患者可能会出现手臂旋转、屈伸等动作受限；下肢骨（如股骨、胫骨）受累时，患者的行走、跑步、上下楼梯等活动会受到影响。

- 若是脊柱发病，根据病变的部位不同，活动受限的情况也不同。例如颈椎病变可能会导致转头、低头、仰头困难；胸椎病变可能会使患者在扭转身体时感到疼痛和活动受限；腰椎病变则常常会影响弯腰、伸腰等动作。

4成人慢性非细菌性骨炎最常受累的部位是那些？


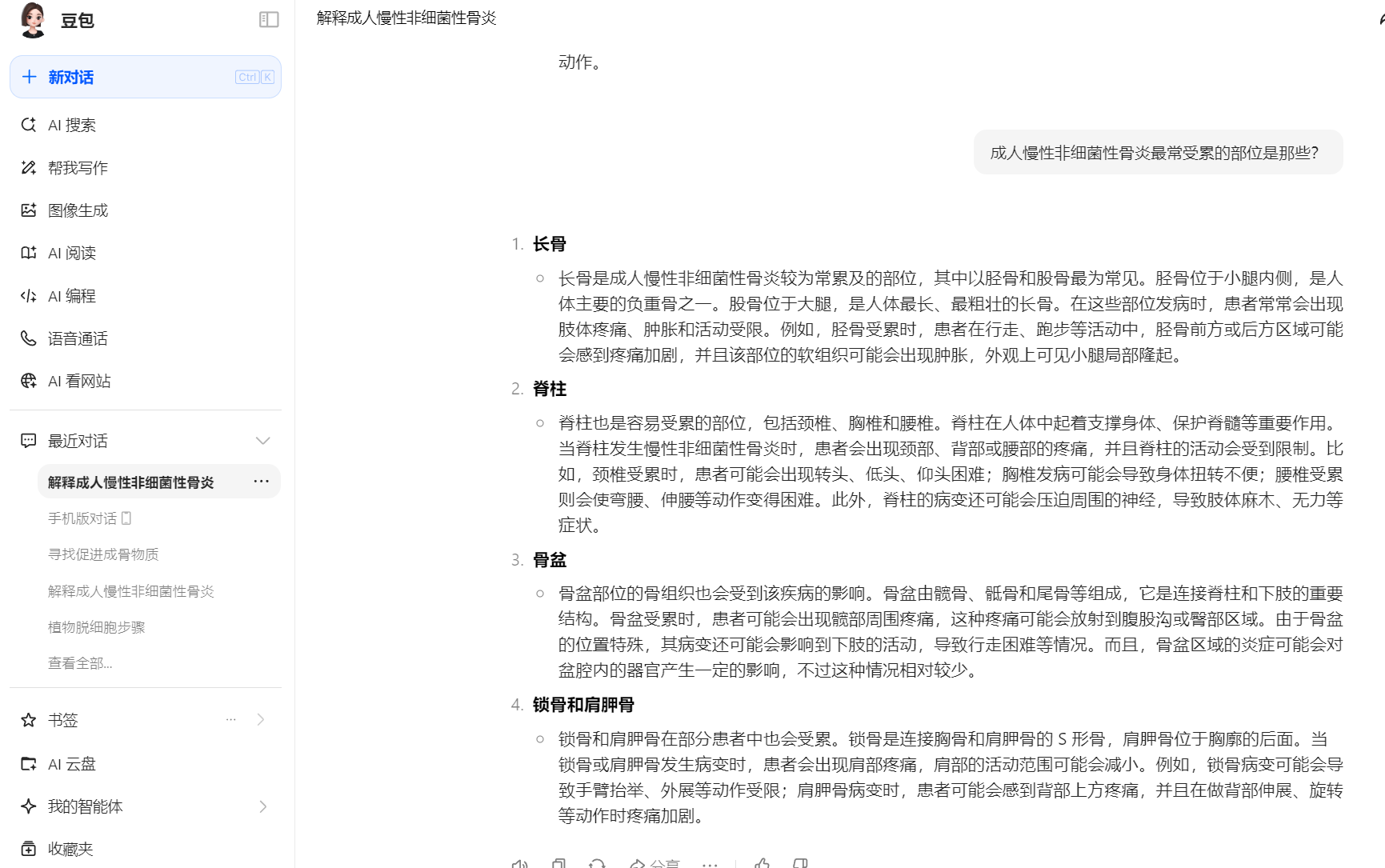
1. **长骨**

- 长骨是成人慢性非细菌性骨炎较为常累及的部位，其中以胫骨和股骨最为常见。胫骨位于小腿内侧，是人体主要的负重骨之一。股骨位于大腿，是人体最长、最粗壮的长骨。在这些部位发病时，患者常常会出现肢体疼痛、肿胀和活动受限。例如，胫骨受累时，患者在行走、跑步等活动中，胫骨前方或后方区域可能会感到疼痛加剧，并且该部位的软组织可能会出现肿胀，外观上可见小腿局部隆起。

2. **脊柱**

- 脊柱也是容易受累的部位，包括颈椎、胸椎和腰椎。脊柱在人体中起着支撑身体、保护脊髓等重要作用。当脊柱发生慢性非细菌性骨炎时，患者会出现颈部、背部或腰部的疼痛，并且脊柱的活动会受到限制。比如，颈椎受累时，患者可能会出现转头、低头、仰头困难；胸椎发病可能会导致身体扭转不便；腰椎受累则会使弯腰、伸腰等动作变得困难。此外，脊柱的病变还可能会压迫周围的神经，导致肢体麻木、无力等症状。

3. **骨盆**

- 骨盆部位的骨组织也会受到该疾病的影响。骨盆由髋骨、骶骨和尾骨等组成，它是连接脊柱和下肢的重要结构。骨盆受累时，患者可能会出现髋部周围疼痛，这种疼痛可能会放射到腹股沟或臀部区域。由于骨盆的位置特殊，其病变还可能会影响到下肢的活动，导致行走困难等情况。而且，骨盆区域的炎症可能会对盆腔内的器官产生一定的影响，不过这种情况相对较少。

4. **锁骨和肩胛骨**

- 锁骨和肩胛骨在部分患者中也会受累。锁骨是连接胸骨和肩胛骨的S形骨，肩胛骨位于胸廓的后面。当锁骨或肩胛骨发生病变时，患者会出现肩部疼痛，肩部的活动范围可能会减小。例如，锁骨病变可能会导致手臂抬举、外展等动作受限；肩胛骨病变时，患者可能会感到背部上方疼痛，并且在做背部伸展、旋转等动作时疼痛加剧。

5成人慢性非细菌性骨炎有没有特异性的炎症标志物？


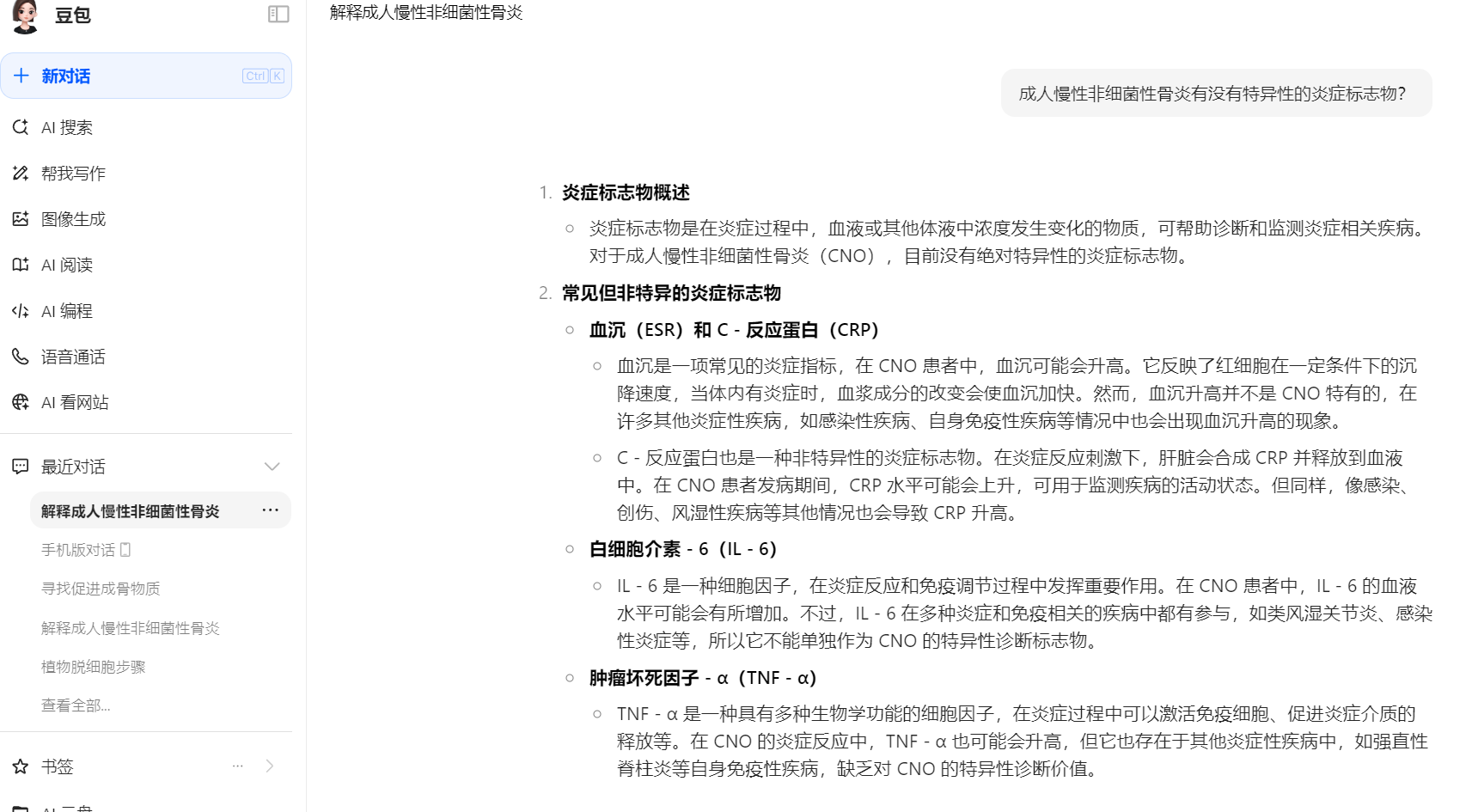
1. **炎症标志物概述**

- 炎症标志物是在炎症过程中，血液或其他体液中浓度发生变化的物质，可帮助诊断和监测炎症相关疾病。对于成人慢性非细菌性骨炎（CNO），目前没有绝对特异性的炎症标志物。

2. **常见但非特异的炎症标志物**

- **血沉（ESR）和C - 反应蛋白（CRP）**

- 血沉是一项常见的炎症指标，在CNO患者中，血沉可能会升高。它反映了红细胞在一定条件下的沉降速度，当体内有炎症时，血浆成分的改变会使血沉加快。然而，血沉升高并不是CNO特有的，在许多其他炎症性疾病，如感染性疾病、自身免疫性疾病等情况中也会出现血沉升高的现象。

- C - 反应蛋白也是一种非特异性的炎症标志物。在炎症反应刺激下，肝脏会合成CRP并释放到血液中。在CNO患者发病期间，CRP水平可能会上升，可用于监测疾病的活动状态。但同样，像感染、创伤、风湿性疾病等其他情况也会导致CRP升高。

- **白细胞介素 - 6（IL - 6）**

- IL - 6是一种细胞因子，在炎症反应和免疫调节过程中发挥重要作用。在CNO患者中，IL - 6的血液水平可能会有所增加。不过，IL - 6在多种炎症和免疫相关的疾病中都有参与，如类风湿关节炎、感染性炎症等，所以它不能单独作为CNO的特异性诊断标志物。

- **肿瘤坏死因子 - α（TNF - α）**

- TNF - α是一种具有多种生物学功能的细胞因子，在炎症过程中可以激活免疫细胞、促进炎症介质的释放等。在CNO的炎症反应中，TNF - α也可能会升高，但它也存在于其他炎症性疾病中，如强直性脊柱炎等自身免疫性疾病，缺乏对CNO的特异性诊断价值。

6慢性非细菌性骨炎首选的影像学检查是什么？


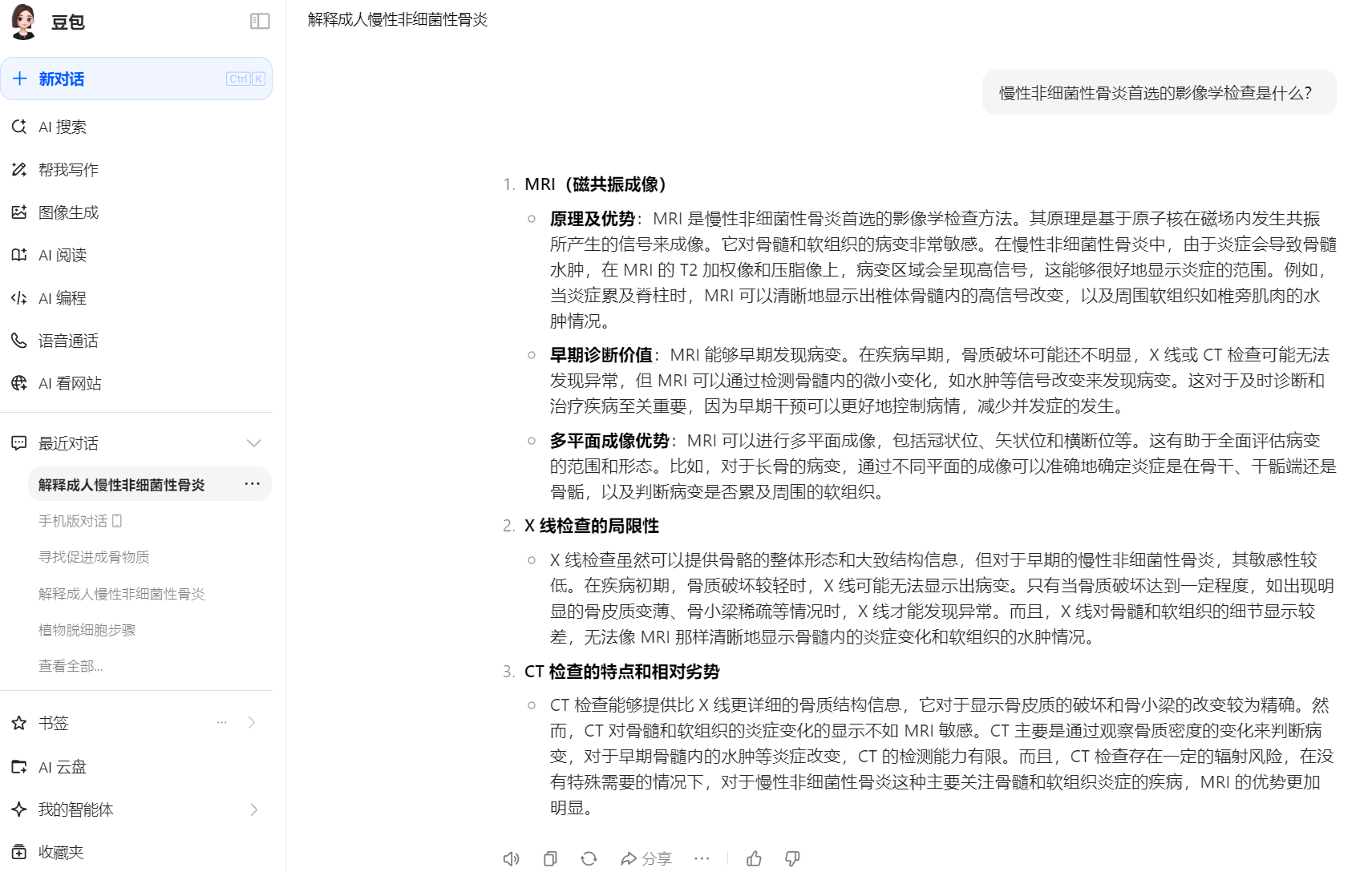
1. **MRI（磁共振成像）**

- **原理及优势**：MRI是慢性非细菌性骨炎首选的影像学检查方法。其原理是基于原子核在磁场内发生共振所产生的信号来成像。它对骨髓和软组织的病变非常敏感。在慢性非细菌性骨炎中，由于炎症会导致骨髓水肿，在MRI的T2加权像和压脂像上，病变区域会呈现高信号，这能够很好地显示炎症的范围。例如，当炎症累及脊柱时，MRI可以清晰地显示出椎体骨髓内的高信号改变，以及周围软组织如椎旁肌肉的水肿情况。

- **早期诊断价值**：MRI能够早期发现病变。在疾病早期，骨质破坏可能还不明显，X线或CT检查可能无法发现异常，但MRI可以通过检测骨髓内的微小变化，如水肿等信号改变来发现病变。这对于及时诊断和治疗疾病至关重要，因为早期干预可以更好地控制病情，减少并发症的发生。

- **多平面成像优势**：MRI可以进行多平面成像，包括冠状位、矢状位和横断位等。这有助于全面评估病变的范围和形态。比如，对于长骨的病变，通过不同平面的成像可以准确地确定炎症是在骨干、干骺端还是骨骺，以及判断病变是否累及周围的软组织。

2. **X线检查的局限性**

- X线检查虽然可以提供骨骼的整体形态和大致结构信息，但对于早期的慢性非细菌性骨炎，其敏感性较低。在疾病初期，骨质破坏较轻时，X线可能无法显示出病变。只有当骨质破坏达到一定程度，如出现明显的骨皮质变薄、骨小梁稀疏等情况时，X线才能发现异常。而且，X线对骨髓和软组织的细节显示较差，无法像MRI那样清晰地显示骨髓内的炎症变化和软组织的水肿情况。

3. **CT检查的特点和相对劣势**

- CT检查能够提供比X线更详细的骨质结构信息，它对于显示骨皮质的破坏和骨小梁的改变较为精确。然而，CT对骨髓和软组织的炎症变化的显示不如MRI敏感。CT主要是通过观察骨质密度的变化来判断病变，对于早期骨髓内的水肿等炎症改变，CT的检测能力有限。而且，CT检查存在一定的辐射风险，在没有特殊需要的情况下，对于慢性非细菌性骨炎这种主要关注骨髓和软组织炎症的疾病，MRI的优势更加明显。

7全身成像检查是不是诊断成人慢性非细菌性骨炎的先决条件？


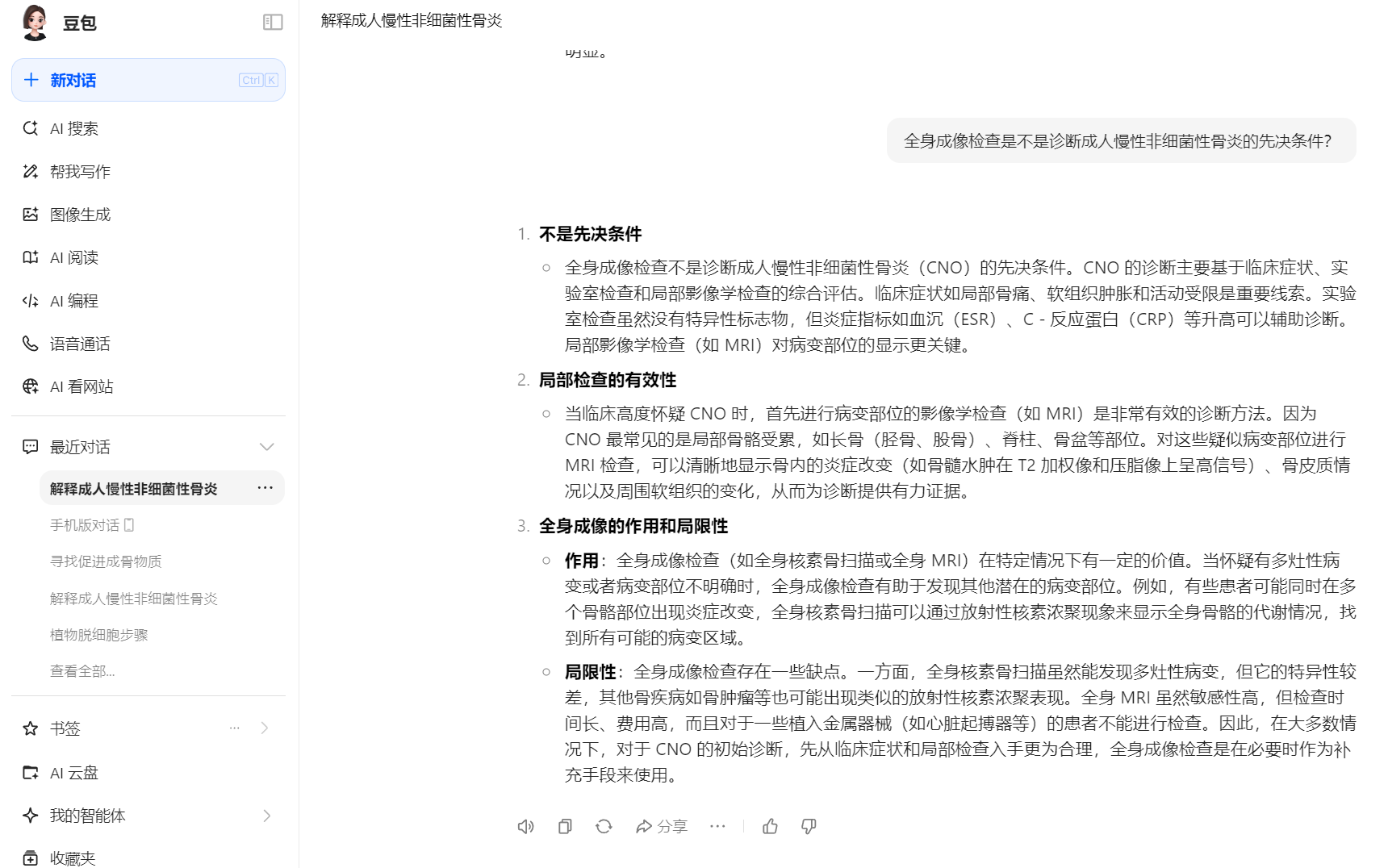
1. **不是先决条件**

- 全身成像检查不是诊断成人慢性非细菌性骨炎（CNO）的先决条件。CNO的诊断主要基于临床症状、实验室检查和局部影像学检查的综合评估。临床症状如局部骨痛、软组织肿胀和活动受限是重要线索。实验室检查虽然没有特异性标志物，但炎症指标如血沉（ESR）、C -反应蛋白（CRP）等升高可以辅助诊断。局部影像学检查（如MRI）对病变部位的显示更关键。

2. **局部检查的有效性**

- 当临床高度怀疑CNO时，首先进行病变部位的影像学检查（如MRI）是非常有效的诊断方法。因为CNO最常见的是局部骨骼受累，如长骨（胫骨、股骨）、脊柱、骨盆等部位。对这些疑似病变部位进行MRI检查，可以清晰地显示骨内的炎症改变（如骨髓水肿在T2加权像和压脂像上呈高信号）、骨皮质情况以及周围软组织的变化，从而为诊断提供有力证据。

3. **全身成像的作用和局限性**

- **作用**：全身成像检查（如全身核素骨扫描或全身MRI）在特定情况下有一定的价值。当怀疑有多灶性病变或者病变部位不明确时，全身成像检查有助于发现其他潜在的病变部位。例如，有些患者可能同时在多个骨骼部位出现炎症改变，全身核素骨扫描可以通过放射性核素浓聚现象来显示全身骨骼的代谢情况，找到所有可能的病变区域。

- **局限性**：全身成像检查存在一些缺点。一方面，全身核素骨扫描虽然能发现多灶性病变，但它的特异性较差，其他骨疾病如骨肿瘤等也可能出现类似的放射性核素浓聚表现。全身MRI虽然敏感性高，但检查时间长、费用高，而且对于一些植入金属器械（如心脏起搏器等）的患者不能进行检查。因此，在大多数情况下，对于CNO的初始诊断，先从临床症状和局部检查入手更为合理，全身成像检查是在必要时作为补充手段来使用。

8诊断成人慢性非细菌性骨炎有必要常规骨活检吗？


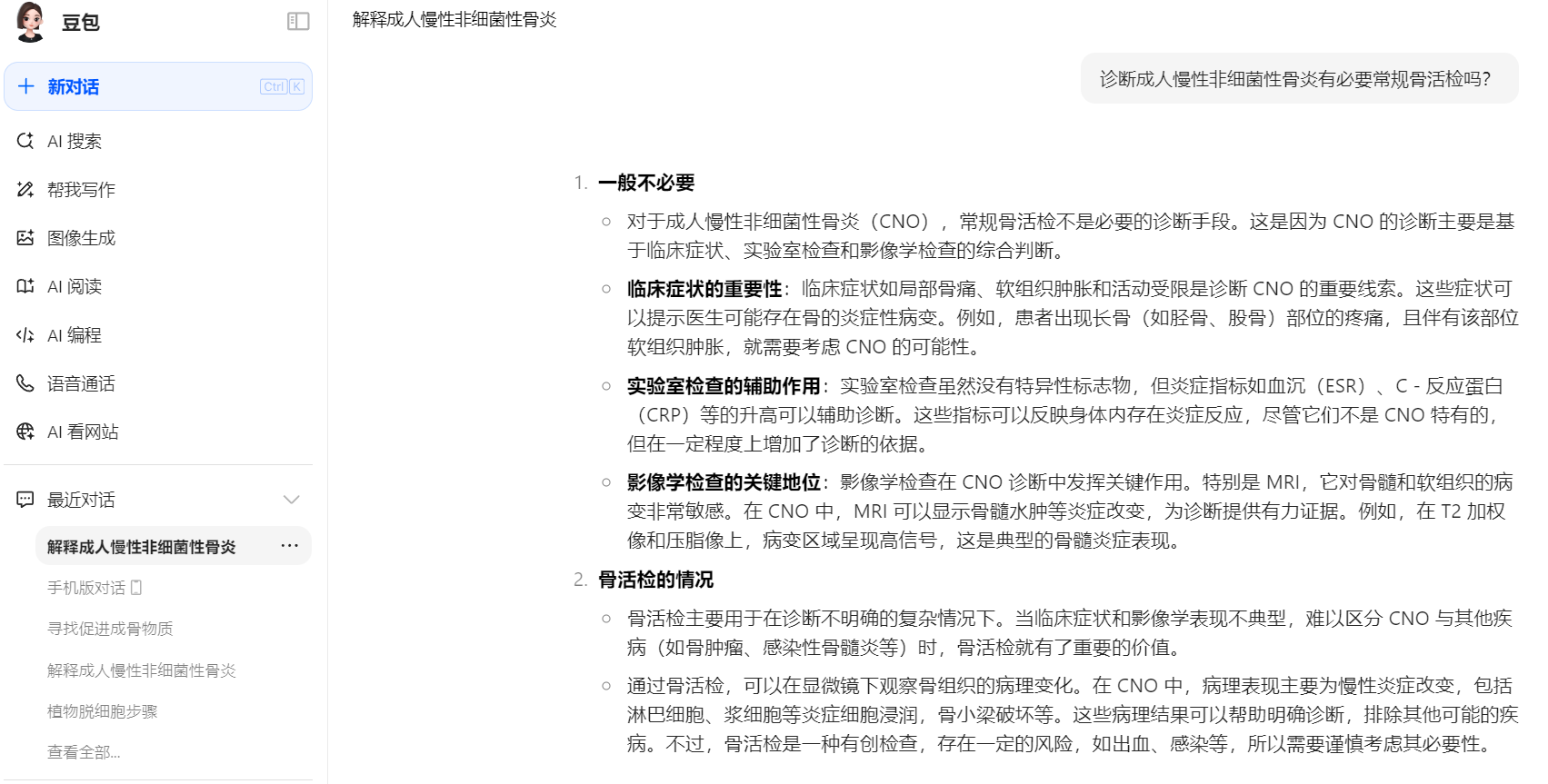
1. **一般不必要**

- 对于成人慢性非细菌性骨炎（CNO），常规骨活检不是必要的诊断手段。这是因为CNO的诊断主要是基于临床症状、实验室检查和影像学检查的综合判断。

- **临床症状的重要性**：临床症状如局部骨痛、软组织肿胀和活动受限是诊断CNO的重要线索。这些症状可以提示医生可能存在骨的炎症性病变。例如，患者出现长骨（如胫骨、股骨）部位的疼痛，且伴有该部位软组织肿胀，就需要考虑CNO的可能性。

- **实验室检查的辅助作用**：实验室检查虽然没有特异性标志物，但炎症指标如血沉（ESR）、C -反应蛋白（CRP）等的升高可以辅助诊断。这些指标可以反映身体内存在炎症反应，尽管它们不是CNO特有的，但在一定程度上增加了诊断的依据。

- **影像学检查的关键地位**：影像学检查在CNO诊断中发挥关键作用。特别是MRI，它对骨髓和软组织的病变非常敏感。在CNO中，MRI可以显示骨髓水肿等炎症改变，为诊断提供有力证据。例如，在T2加权像和压脂像上，病变区域呈现高信号，这是典型的骨髓炎症表现。

2. **骨活检的情况**

- 骨活检主要用于在诊断不明确的复杂情况下。当临床症状和影像学表现不典型，难以区分CNO与其他疾病（如骨肿瘤、感染性骨髓炎等）时，骨活检就有了重要的价值。

- 通过骨活检，可以在显微镜下观察骨组织的病理变化。在CNO中，病理表现主要为慢性炎症改变，包括淋巴细胞、浆细胞等炎症细胞浸润，骨小梁破坏等。这些病理结果可以帮助明确诊断，排除其他可能的疾病。不过，骨活检是一种有创检查，存在一定的风险，如出血、感染等，所以需要谨慎考虑其必要性。

9慢性非细菌性骨炎如何与恶性骨肿瘤鉴别？


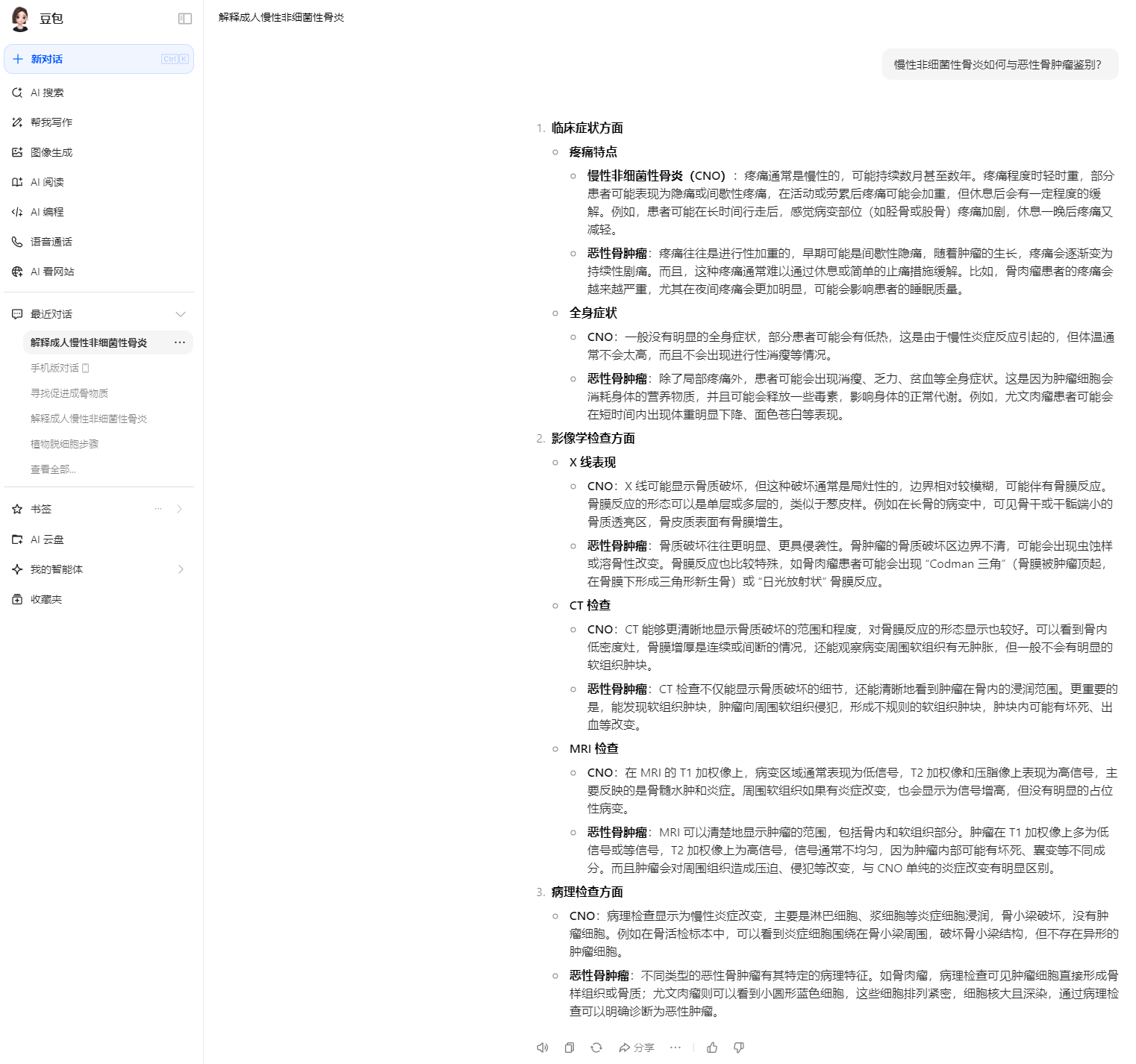
1. **临床症状方面**

- **疼痛特点**

- **慢性非细菌性骨炎（CNO）**：疼痛通常是慢性的，可能持续数月甚至数年。疼痛程度时轻时重，部分患者可能表现为隐痛或间歇性疼痛，在活动或劳累后疼痛可能会加重，但休息后会有一定程度的缓解。例如，患者可能在长时间行走后，感觉病变部位（如胫骨或股骨）疼痛加剧，休息一晚后疼痛又减轻。

- **恶性骨肿瘤**：疼痛往往是进行性加重的，早期可能是间歇性隐痛，随着肿瘤的生长，疼痛会逐渐变为持续性剧痛。而且，这种疼痛通常难以通过休息或简单的止痛措施缓解。比如，骨肉瘤患者的疼痛会越来越严重，尤其在夜间疼痛会更加明显，可能会影响患者的睡眠质量。

- **全身症状**

- **CNO**：一般没有明显的全身症状，部分患者可能会有低热，这是由于慢性炎症反应引起的，但体温通常不会太高，而且不会出现进行性消瘦等情况。

- **恶性骨肿瘤**：除了局部疼痛外，患者可能会出现消瘦、乏力、贫血等全身症状。这是因为肿瘤细胞会消耗身体的营养物质，并且可能会释放一些毒素，影响身体的正常代谢。例如，尤文肉瘤患者可能会在短时间内出现体重明显下降、面色苍白等表现。

2. **影像学检查方面**

- **X线表现**

- **CNO**：X线可能显示骨质破坏，但这种破坏通常是局灶性的，边界相对较模糊，可能伴有骨膜反应。骨膜反应的形态可以是单层或多层的，类似于葱皮样。例如在长骨的病变中，可见骨干或干骺端小的骨质透亮区，骨皮质表面有骨膜增生。

- **恶性骨肿瘤**：骨质破坏往往更明显、更具侵袭性。骨肿瘤的骨质破坏区边界不清，可能会出现虫蚀样或溶骨性改变。骨膜反应也比较特殊，如骨肉瘤患者可能会出现“Codman三角”（骨膜被肿瘤顶起，在骨膜下形成三角形新生骨）或“日光放射状”骨膜反应。

- **CT检查**

- **CNO**：CT能够更清晰地显示骨质破坏的范围和程度，对骨膜反应的形态显示也较好。可以看到骨内低密度灶，骨膜增厚是连续或间断的情况，还能观察病变周围软组织有无肿胀，但一般不会有明显的软组织肿块。

- **恶性骨肿瘤**：CT检查不仅能显示骨质破坏的细节，还能清晰地看到肿瘤在骨内的浸润范围。更重要的是，能发现软组织肿块，肿瘤向周围软组织侵犯，形成不规则的软组织肿块，肿块内可能有坏死、出血等改变。

- **MRI检查**

- **CNO**：在MRI的T1加权像上，病变区域通常表现为低信号，T2加权像和压脂像上表现为高信号，主要反映的是骨髓水肿和炎症。周围软组织如果有炎症改变，也会显示为信号增高，但没有明显的占位性病变。

- **恶性骨肿瘤**：MRI可以清楚地显示肿瘤的范围，包括骨内和软组织部分。肿瘤在T1加权像上多为低信号或等信号，T2加权像上为高信号，信号通常不均匀，因为肿瘤内部可能有坏死、囊变等不同成分。而且肿瘤会对周围组织造成压迫、侵犯等改变，与CNO单纯的炎症改变有明显区别。

3. **病理检查方面**

- **CNO**：病理检查显示为慢性炎症改变，主要是淋巴细胞、浆细胞等炎症细胞浸润，骨小梁破坏，没有肿瘤细胞。例如在骨活检标本中，可以看到炎症细胞围绕在骨小梁周围，破坏骨小梁结构，但不存在异形的肿瘤细胞。

- **恶性骨肿瘤**：不同类型的恶性骨肿瘤有其特定的病理特征。如骨肉瘤，病理检查可见肿瘤细胞直接形成骨样组织或骨质；尤文肉瘤则可以看到小圆形蓝色细胞，这些细胞排列紧密，细胞核大且深染，通过病理检查可以明确诊断为恶性肿瘤。

10慢性非细菌性骨炎如何与骨关节炎鉴别？


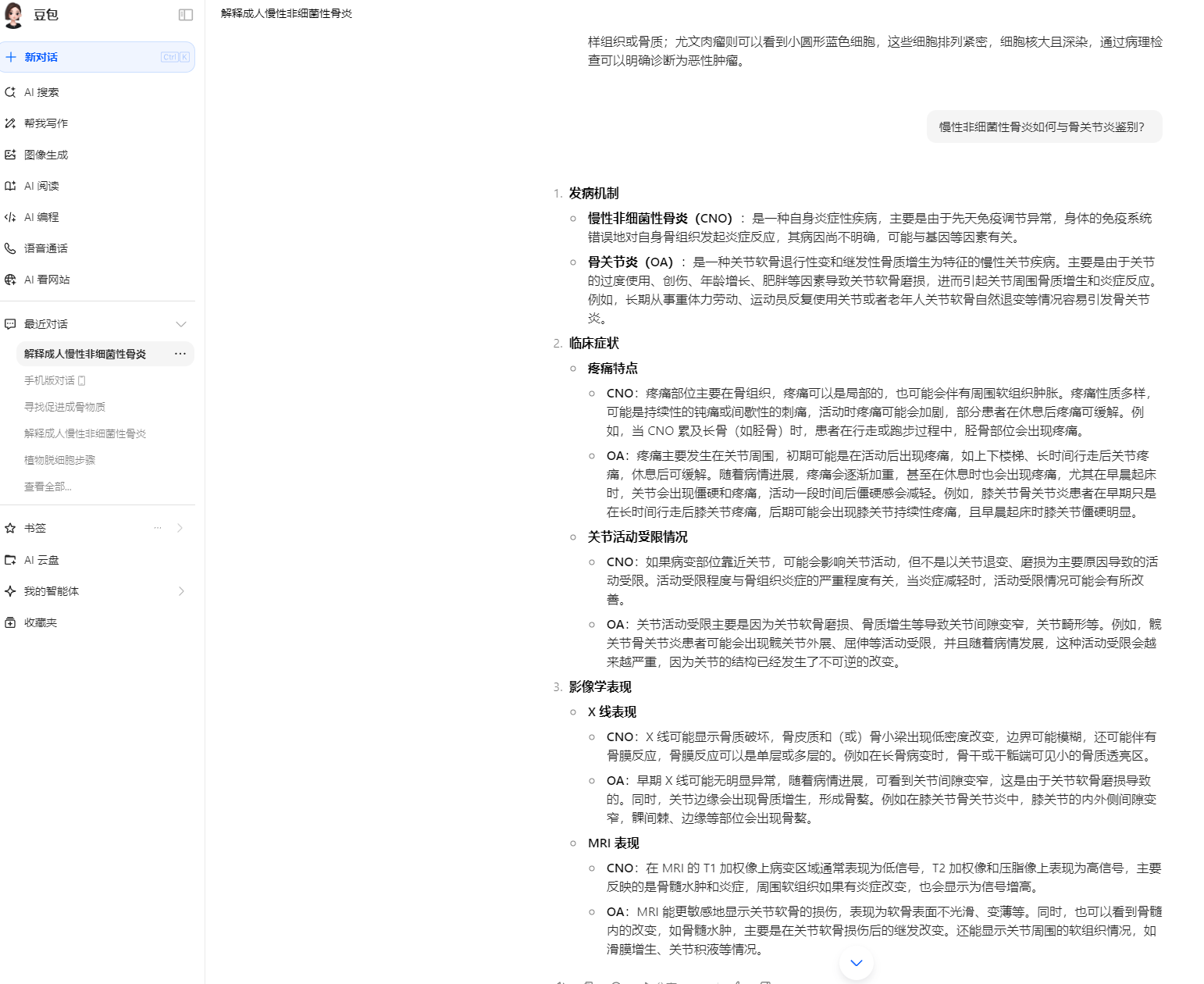
 1. **发病机制**

- **慢性非细菌性骨炎（CNO）**：是一种自身炎症性疾病，主要是由于先天免疫调节异常，身体的免疫系统错误地对自身骨组织发起炎症反应，其病因尚不明确，可能与基因等因素有关。

- **骨关节炎（OA）**：是一种关节软骨退行性变和继发性骨质增生为特征的慢性关节疾病。主要是由于关节的过度使用、创伤、年龄增长、肥胖等因素导致关节软骨磨损，进而引起关节周围骨质增生和炎症反应。例如，长期从事重体力劳动、运动员反复使用关节或者老年人关节软骨自然退变等情况容易引发骨关节炎。

2. **临床症状**

- **疼痛特点**

- **CNO**：疼痛部位主要在骨组织，疼痛可以是局部的，也可能会伴有周围软组织肿胀。疼痛性质多样，可能是持续性的钝痛或间歇性的刺痛，活动时疼痛可能会加剧，部分患者在休息后疼痛可缓解。例如，当CNO累及长骨（如胫骨）时，患者在行走或跑步过程中，胫骨部位会出现疼痛。

- **OA**：疼痛主要发生在关节周围，初期可能是在活动后出现疼痛，如上下楼梯、长时间行走后关节疼痛，休息后可缓解。随着病情进展，疼痛会逐渐加重，甚至在休息时也会出现疼痛，尤其在早晨起床时，关节会出现僵硬和疼痛，活动一段时间后僵硬感会减轻。例如，膝关节骨关节炎患者在早期只是在长时间行走后膝关节疼痛，后期可能会出现膝关节持续性疼痛，且早晨起床时膝关节僵硬明显。

- **关节活动受限情况**

- **CNO**：如果病变部位靠近关节，可能会影响关节活动，但不是以关节退变、磨损为主要原因导致的活动受限。活动受限程度与骨组织炎症的严重程度有关，当炎症减轻时，活动受限情况可能会有所改善。

- **OA**：关节活动受限主要是因为关节软骨磨损、骨质增生等导致关节间隙变窄，关节畸形等。例如，髋关节骨关节炎患者可能会出现髋关节外展、屈伸等活动受限，并且随着病情发展，这种活动受限会越来越严重，因为关节的结构已经发生了不可逆的改变。

3. **影像学表现**

- **X线表现**

- **CNO**：X线可能显示骨质破坏，骨皮质和（或）骨小梁出现低密度改变，边界可能模糊，还可能伴有骨膜反应，骨膜反应可以是单层或多层的。例如在长骨病变时，骨干或干骺端可见小的骨质透亮区。

- **OA**：早期X线可能无明显异常，随着病情进展，可看到关节间隙变窄，这是由于关节软骨磨损导致的。同时，关节边缘会出现骨质增生，形成骨赘。例如在膝关节骨关节炎中，膝关节的内外侧间隙变窄，髁间棘、边缘等部位会出现骨赘。

- **MRI表现**

- **CNO**：在MRI的T1加权像上病变区域通常表现为低信号，T2加权像和压脂像上表现为高信号，主要反映的是骨髓水肿和炎症，周围软组织如果有炎症改变，也会显示为信号增高。

- **OA**：MRI能更敏感地显示关节软骨的损伤，表现为软骨表面不光滑、变薄等。同时，也可以看到骨髓内的改变，如骨髓水肿，主要是在关节软骨损伤后的继发改变。还能显示关节周围的软组织情况，如滑膜增生、关节积液等情况。

11慢性非细菌性骨炎如何与纤维发育不良鉴别？


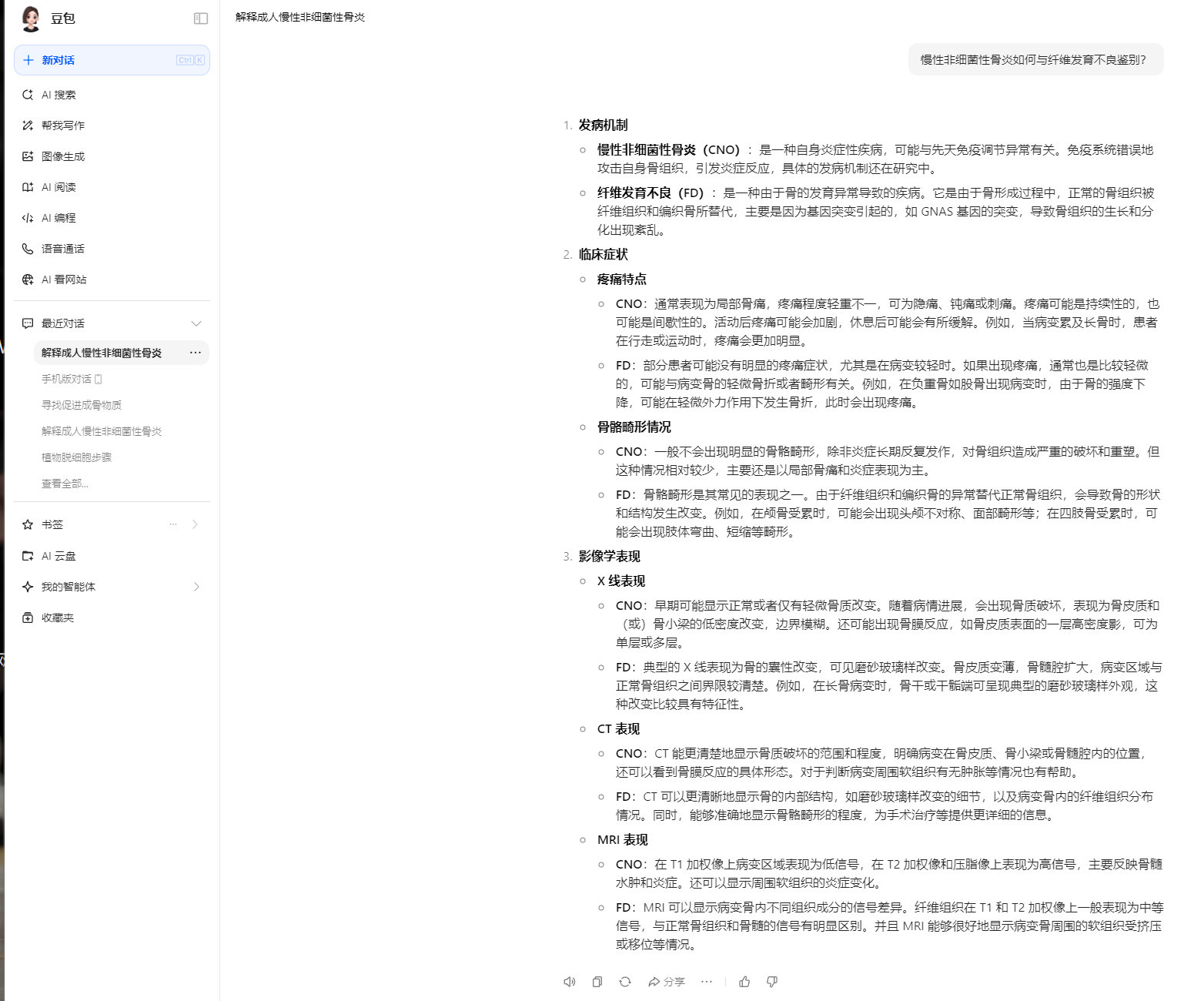
 1. **发病机制**

- **慢性非细菌性骨炎（CNO）**：是一种自身炎症性疾病，可能与先天免疫调节异常有关。免疫系统错误地攻击自身骨组织，引发炎症反应，具体的发病机制还在研究中。

- **纤维发育不良（FD）**：是一种由于骨的发育异常导致的疾病。它是由于骨形成过程中，正常的骨组织被纤维组织和编织骨所替代，主要是因为基因突变引起的，如GNAS基因的突变，导致骨组织的生长和分化出现紊乱。

2. **临床症状**

- **疼痛特点**

- **CNO**：通常表现为局部骨痛，疼痛程度轻重不一，可为隐痛、钝痛或刺痛。疼痛可能是持续性的，也可能是间歇性的。活动后疼痛可能会加剧，休息后可能会有所缓解。例如，当病变累及长骨时，患者在行走或运动时，疼痛会更加明显。

- **FD**：部分患者可能没有明显的疼痛症状，尤其是在病变较轻时。如果出现疼痛，通常也是比较轻微的，可能与病变骨的轻微骨折或者畸形有关。例如，在负重骨如股骨出现病变时，由于骨的强度下降，可能在轻微外力作用下发生骨折，此时会出现疼痛。

- **骨骼畸形情况**

- **CNO**：一般不会出现明显的骨骼畸形，除非炎症长期反复发作，对骨组织造成严重的破坏和重塑。但这种情况相对较少，主要还是以局部骨痛和炎症表现为主。

- **FD**：骨骼畸形是其常见的表现之一。由于纤维组织和编织骨的异常替代正常骨组织，会导致骨的形状和结构发生改变。例如，在颅骨受累时，可能会出现头颅不对称、面部畸形等；在四肢骨受累时，可能会出现肢体弯曲、短缩等畸形。

3. **影像学表现**

- **X线表现**

- **CNO**：早期可能显示正常或者仅有轻微骨质改变。随着病情进展，会出现骨质破坏，表现为骨皮质和（或）骨小梁的低密度改变，边界模糊。还可能出现骨膜反应，如骨皮质表面的一层高密度影，可为单层或多层。

- **FD**：典型的X线表现为骨的囊性改变，可见磨砂玻璃样改变。骨皮质变薄，骨髓腔扩大，病变区域与正常骨组织之间界限较清楚。例如，在长骨病变时，骨干或干骺端可呈现典型的磨砂玻璃样外观，这种改变比较具有特征性。

- **CT表现**

- **CNO**：CT能更清楚地显示骨质破坏的范围和程度，明确病变在骨皮质、骨小梁或骨髓腔内的位置，还可以看到骨膜反应的具体形态。对于判断病变周围软组织有无肿胀等情况也有帮助。

- **FD**：CT可以更清晰地显示骨的内部结构，如磨砂玻璃样改变的细节，以及病变骨内的纤维组织分布情况。同时，能够准确地显示骨骼畸形的程度，为手术治疗等提供更详细的信息。

- **MRI表现**

- **CNO**：在T1加权像上病变区域表现为低信号，在T2加权像和压脂像上表现为高信号，主要反映骨髓水肿和炎症。还可以显示周围软组织的炎症变化。

- **FD**：MRI可以显示病变骨内不同组织成分的信号差异。纤维组织在T1和T2加权像上一般表现为中等信号，与正常骨组织和骨髓的信号有明显区别。并且MRI能够很好地显示病变骨周围的软组织受挤压或移位等情况。

12在治慢性非细菌性骨炎过程中应该选择短疗程的糖皮质激素治疗还是长期的糖皮质激素治疗？


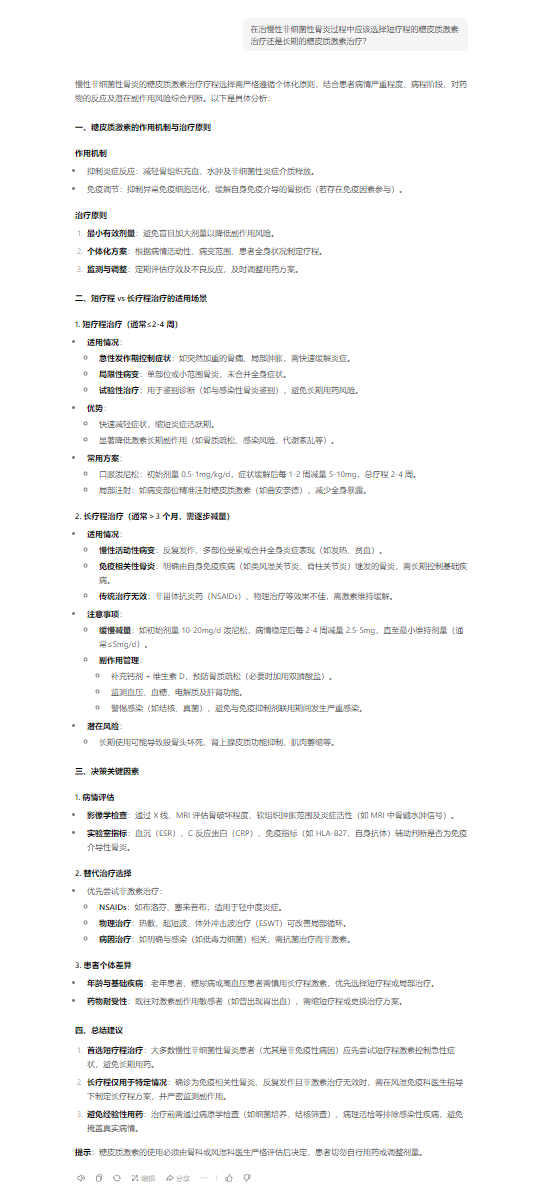


慢性非细菌性骨炎的糖皮质激素治疗疗程选择需严格遵循个体化原则，结合患者病情严重程度、病程阶段、对药物的反应及潜在副作用风险综合判断。以下是具体分析：

### **一、糖皮质激素的作用机制与治疗原则**

#### **作用机制**

- 抑制炎症反应：减轻骨组织充血、水肿及非细菌性炎症介质释放。

- 免疫调节：抑制异常免疫细胞活化，缓解自身免疫介导的骨损伤（若存在免疫因素参与）。

#### **治疗原则**

1. **最小有效剂量**：避免盲目加大剂量以降低副作用风险。

2. **个体化方案**：根据病情活动性、病变范围、患者全身状况制定疗程。

3. **监测与调整**：定期评估疗效及不良反应，及时调整用药方案。

### **二、短疗程 vs 长疗程治疗的适用场景**

#### **1. 短疗程治疗（通常≤2-4周）**

- **适用情况**：

- **急性发作期控制症状**：如突然加重的骨痛、局部肿胀，需快速缓解炎症。

- **局限性病变**：单部位或小范围骨炎，未合并全身症状。

- **试验性治疗**：用于鉴别诊断（如与感染性骨炎鉴别），避免长期用药风险。

- **优势**：

- 快速减轻症状，缩短炎症活跃期。

- 显著降低激素长期副作用（如骨质疏松、感染风险、代谢紊乱等）。

- **常用方案**：

- 口服泼尼松：初始剂量0.5-1mg/kg/d，症状缓解后每1-2周减量5-10mg，总疗程2-4周。

- 局部注射：如病变部位精准注射糖皮质激素（如曲安奈德），减少全身暴露。

#### **2. 长疗程治疗（通常＞3个月，需逐步减量）**

- **适用情况**：

- **慢性活动性病变**：反复发作、多部位受累或合并全身炎症表现（如发热、贫血）。

- **免疫相关性骨炎**：明确由自身免疫疾病（如类风湿关节炎、脊柱关节炎）继发的骨炎，需长期控制基础疾病。

- **传统治疗无效**：非甾体抗炎药（NSAIDs）、物理治疗等效果不佳，需激素维持缓解。

- **注意事项**：

- **缓慢减量**：如初始剂量10-20mg/d泼尼松，病情稳定后每2-4周减量2.5-5mg，直至最小维持剂量（通常≤5mg/d）。

- **副作用管理**：

- 补充钙剂+维生素D，预防骨质疏松（必要时加用双膦酸盐）。

- 监测血压、血糖、电解质及肝肾功能。

- 警惕感染（如结核、真菌），避免与免疫抑制剂联用期间发生严重感染。

- **潜在风险**：

- 长期使用可能导致股骨头坏死、肾上腺皮质功能抑制、肌肉萎缩等。

### **三、决策关键因素**

#### **1. 病情评估**

- **影像学检查**：通过X线、MRI评估骨破坏程度、软组织肿胀范围及炎症活性（如MRI中骨髓水肿信号）。

- **实验室指标**：血沉（ESR）、C反应蛋白（CRP）、免疫指标（如HLA-B27、自身抗体）辅助判断是否为免疫介导性骨炎。

#### **2. 替代治疗选择**

- 优先尝试非激素治疗：

- **NSAIDs**：如布洛芬、塞来昔布，适用于轻中度炎症。

- **物理治疗**：热敷、超短波、体外冲击波治疗（ESWT）可改善局部循环。

- **病因治疗**：如明确与感染（如低毒力细菌）相关，需抗菌治疗而非激素。

#### **3. 患者个体差异**

- **年龄与基础疾病**：老年患者、糖尿病或高血压患者需慎用长疗程激素，优先选择短疗程或局部治疗。

- **药物耐受性**：既往对激素副作用敏感者（如曾出现胃出血），需缩短疗程或更换治疗方案。

### **四、总结建议**

1. **首选短疗程治疗**：大多数慢性非细菌性骨炎患者（尤其是非免疫性病因）应先尝试短疗程激素控制急性症状，避免长期用药。

2. **长疗程仅用于特定情况**：确诊为免疫相关性骨炎、反复发作且非激素治疗无效时，需在风湿免疫科医生指导下制定长疗程方案，并严密监测副作用。

3. **避免经验性用药**：治疗前需通过病原学检查（如细菌培养、结核筛查）、病理活检等排除感染性疾病，避免掩盖真实病情。

**提示**：糖皮质激素的使用必须由骨科或风湿科医生严格评估后决定，患者切勿自行用药或调整剂量。

13成人慢性非细菌性骨炎一线治疗方案是什么？


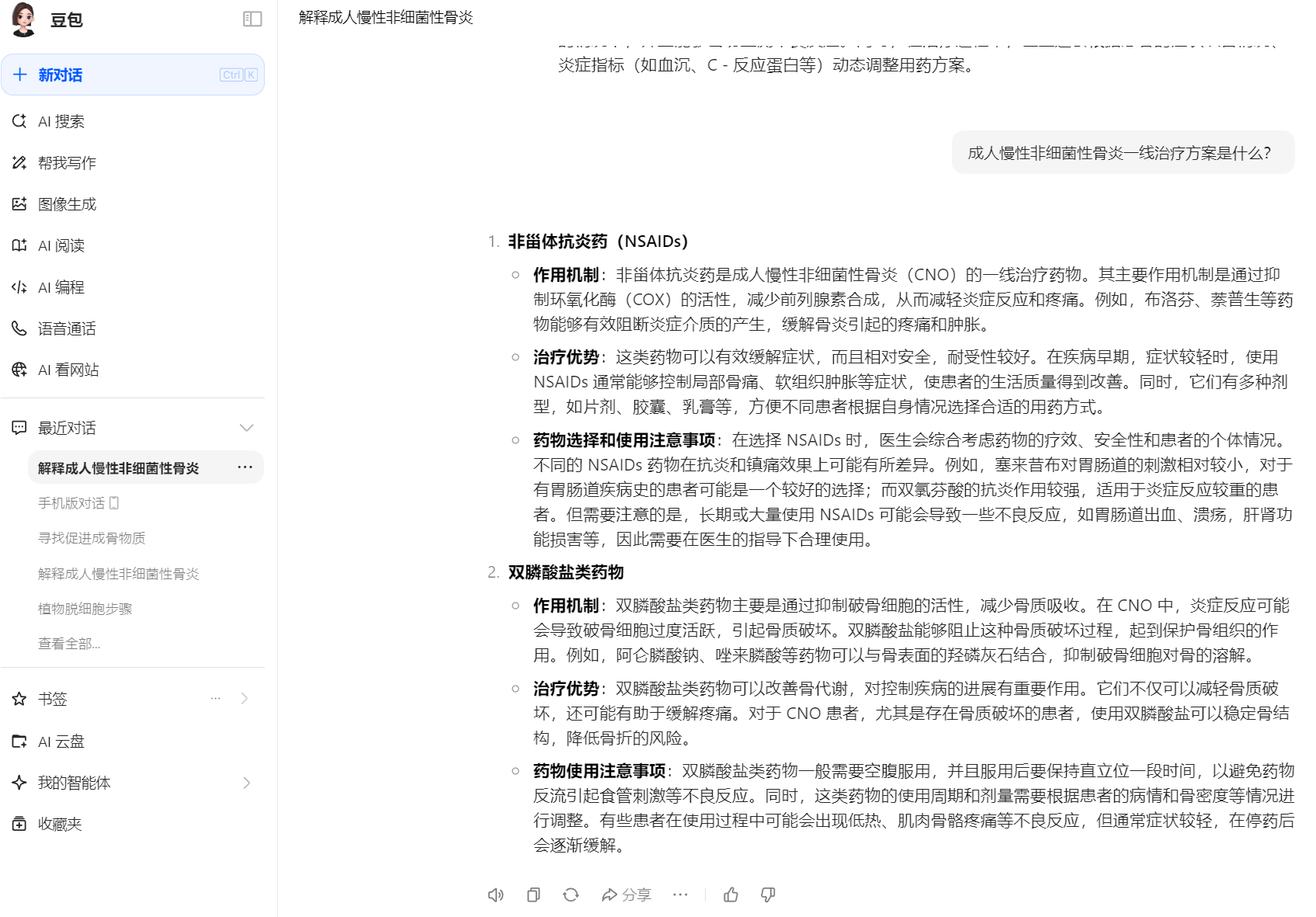
1. **非甾体抗炎药（NSAIDs）**

- **作用机制**：非甾体抗炎药是成人慢性非细菌性骨炎（CNO）的一线治疗药物。其主要作用机制是通过抑制环氧化酶（COX）的活性，减少前列腺素合成，从而减轻炎症反应和疼痛。例如，布洛芬、萘普生等药物能够有效阻断炎症介质的产生，缓解骨炎引起的疼痛和肿胀。

- **治疗优势**：这类药物可以有效缓解症状，而且相对安全，耐受性较好。在疾病早期，症状较轻时，使用NSAIDs通常能够控制局部骨痛、软组织肿胀等症状，使患者的生活质量得到改善。同时，它们有多种剂型，如片剂、胶囊、乳膏等，方便不同患者根据自身情况选择合适的用药方式。

- **药物选择和使用注意事项**：在选择NSAIDs时，医生会综合考虑药物的疗效、安全性和患者的个体情况。不同的NSAIDs药物在抗炎和镇痛效果上可能有所差异。例如，塞来昔布对胃肠道的刺激相对较小，对于有胃肠道疾病史的患者可能是一个较好的选择；而双氯芬酸的抗炎作用较强，适用于炎症反应较重的患者。但需要注意的是，长期或大量使用NSAIDs可能会导致一些不良反应，如胃肠道出血、溃疡，肝肾功能损害等，因此需要在医生的指导下合理使用。

2. **双膦酸盐类药物**

- **作用机制**：双膦酸盐类药物主要是通过抑制破骨细胞的活性，减少骨质吸收。在CNO中，炎症反应可能会导致破骨细胞过度活跃，引起骨质破坏。双膦酸盐能够阻止这种骨质破坏过程，起到保护骨组织的作用。例如，阿仑膦酸钠、唑来膦酸等药物可以与骨表面的羟磷灰石结合，抑制破骨细胞对骨的溶解。

- **治疗优势**：双膦酸盐类药物可以改善骨代谢，对控制疾病的进展有重要作用。它们不仅可以减轻骨质破坏，还可能有助于缓解疼痛。对于CNO患者，尤其是存在骨质破坏的患者，使用双膦酸盐可以稳定骨结构，降低骨折的风险。

- **药物使用注意事项**：双膦酸盐类药物一般需要空腹服用，并且服用后要保持直立位一段时间，以避免药物反流引起食管刺激等不良反应。同时，这类药物的使用周期和剂量需要根据患者的病情和骨密度等情况进行调整。有些患者在使用过程中可能会出现低热、肌肉骨骼疼痛等不良反应，但通常症状较轻，在停药后会逐渐缓解。

14无临床症状，也没有放射学疾病活动的慢性非细菌性骨炎的病人需要治疗吗？


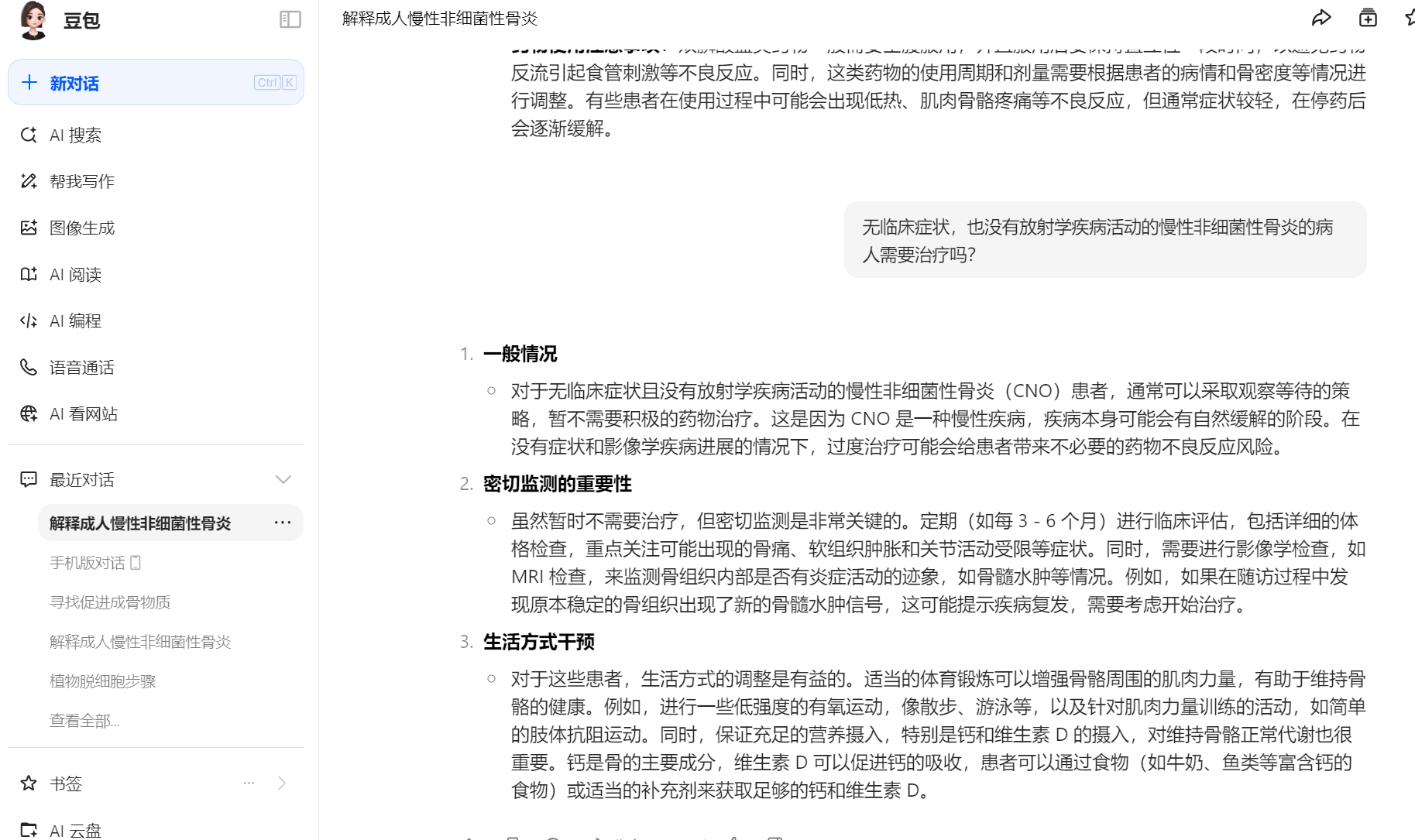
 1. **一般情况**

- 对于无临床症状且没有放射学疾病活动的慢性非细菌性骨炎（CNO）患者，通常可以采取观察等待的策略，暂不需要积极的药物治疗。这是因为CNO是一种慢性疾病，疾病本身可能会有自然缓解的阶段。在没有症状和影像学疾病进展的情况下，过度治疗可能会给患者带来不必要的药物不良反应风险。

2. **密切监测的重要性**

- 虽然暂时不需要治疗，但密切监测是非常关键的。定期（如每3 - 6个月）进行临床评估，包括详细的体格检查，重点关注可能出现的骨痛、软组织肿胀和关节活动受限等症状。同时，需要进行影像学检查，如MRI检查，来监测骨组织内部是否有炎症活动的迹象，如骨髓水肿等情况。例如，如果在随访过程中发现原本稳定的骨组织出现了新的骨髓水肿信号，这可能提示疾病复发，需要考虑开始治疗。

3. **生活方式干预**

- 对于这些患者，生活方式的调整是有益的。适当的体育锻炼可以增强骨骼周围的肌肉力量，有助于维持骨骼的健康。例如，进行一些低强度的有氧运动，像散步、游泳等，以及针对肌肉力量训练的活动，如简单的肢体抗阻运动。同时，保证充足的营养摄入，特别是钙和维生素D的摄入，对维持骨骼正常代谢也很重要。钙是骨的主要成分，维生素D可以促进钙的吸收，患者可以通过食物（如牛奶、鱼类等富含钙的食物）或适当的补充剂来获取足够的钙和维生素D。

15有临床症状没有放射学疾病活动的慢性非细菌性骨炎的病人如何处理？


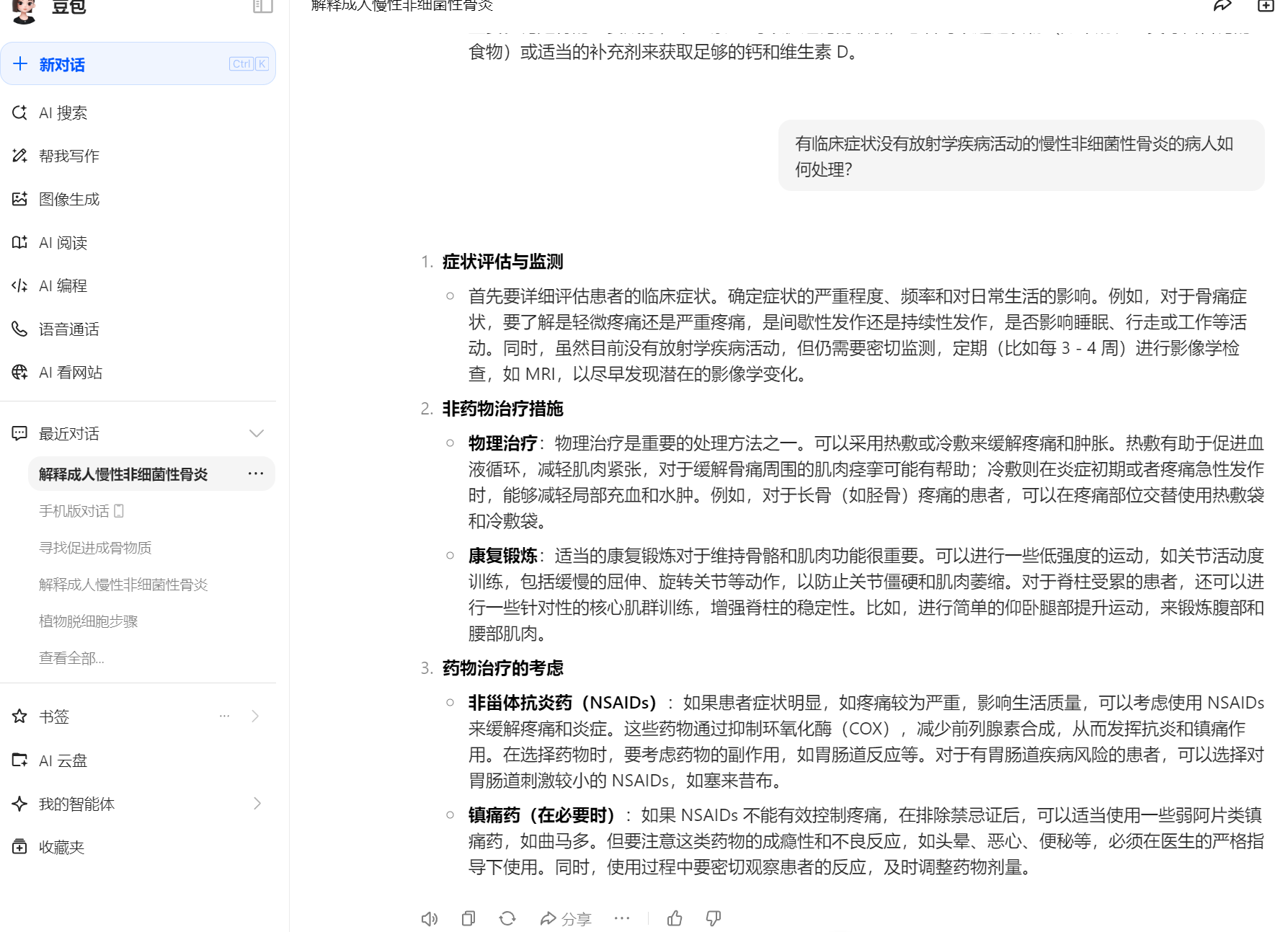
 1. **症状评估与监测**

- 首先要详细评估患者的临床症状。确定症状的严重程度、频率和对日常生活的影响。例如，对于骨痛症状，要了解是轻微疼痛还是严重疼痛，是间歇性发作还是持续性发作，是否影响睡眠、行走或工作等活动。同时，虽然目前没有放射学疾病活动，但仍需要密切监测，定期（比如每3 - 4周）进行影像学检查，如MRI，以尽早发现潜在的影像学变化。

2. **非药物治疗措施**

- **物理治疗**：物理治疗是重要的处理方法之一。可以采用热敷或冷敷来缓解疼痛和肿胀。热敷有助于促进血液循环，减轻肌肉紧张，对于缓解骨痛周围的肌肉痉挛可能有帮助；冷敷则在炎症初期或者疼痛急性发作时，能够减轻局部充血和水肿。例如，对于长骨（如胫骨）疼痛的患者，可以在疼痛部位交替使用热敷袋和冷敷袋。

- **康复锻炼**：适当的康复锻炼对于维持骨骼和肌肉功能很重要。可以进行一些低强度的运动，如关节活动度训练，包括缓慢的屈伸、旋转关节等动作，以防止关节僵硬和肌肉萎缩。对于脊柱受累的患者，还可以进行一些针对性的核心肌群训练，增强脊柱的稳定性。比如，进行简单的仰卧腿部提升运动，来锻炼腹部和腰部肌肉。

3. **药物治疗的考虑**

- **非甾体抗炎药（NSAIDs）**：如果患者症状明显，如疼痛较为严重，影响生活质量，可以考虑使用NSAIDs来缓解疼痛和炎症。这些药物通过抑制环氧化酶（COX），减少前列腺素合成，从而发挥抗炎和镇痛作用。在选择药物时，要考虑药物的副作用，如胃肠道反应等。对于有胃肠道疾病风险的患者，可以选择对胃肠道刺激较小的NSAIDs，如塞来昔布。

- **镇痛药（在必要时）**：如果NSAIDs不能有效控制疼痛，在排除禁忌证后，可以适当使用一些弱阿片类镇痛药，如曲马多。但要注意这类药物的成瘾性和不良反应，如头晕、恶心、便秘等，必须在医生的严格指导下使用。同时，使用过程中要密切观察患者的反应，及时调整药物剂量。

16有椎体塌陷风险的脊柱骨病变的慢性非细菌性骨炎患者和出现明显骨骼累积损伤的慢性非细菌性骨炎患者如何治疗？


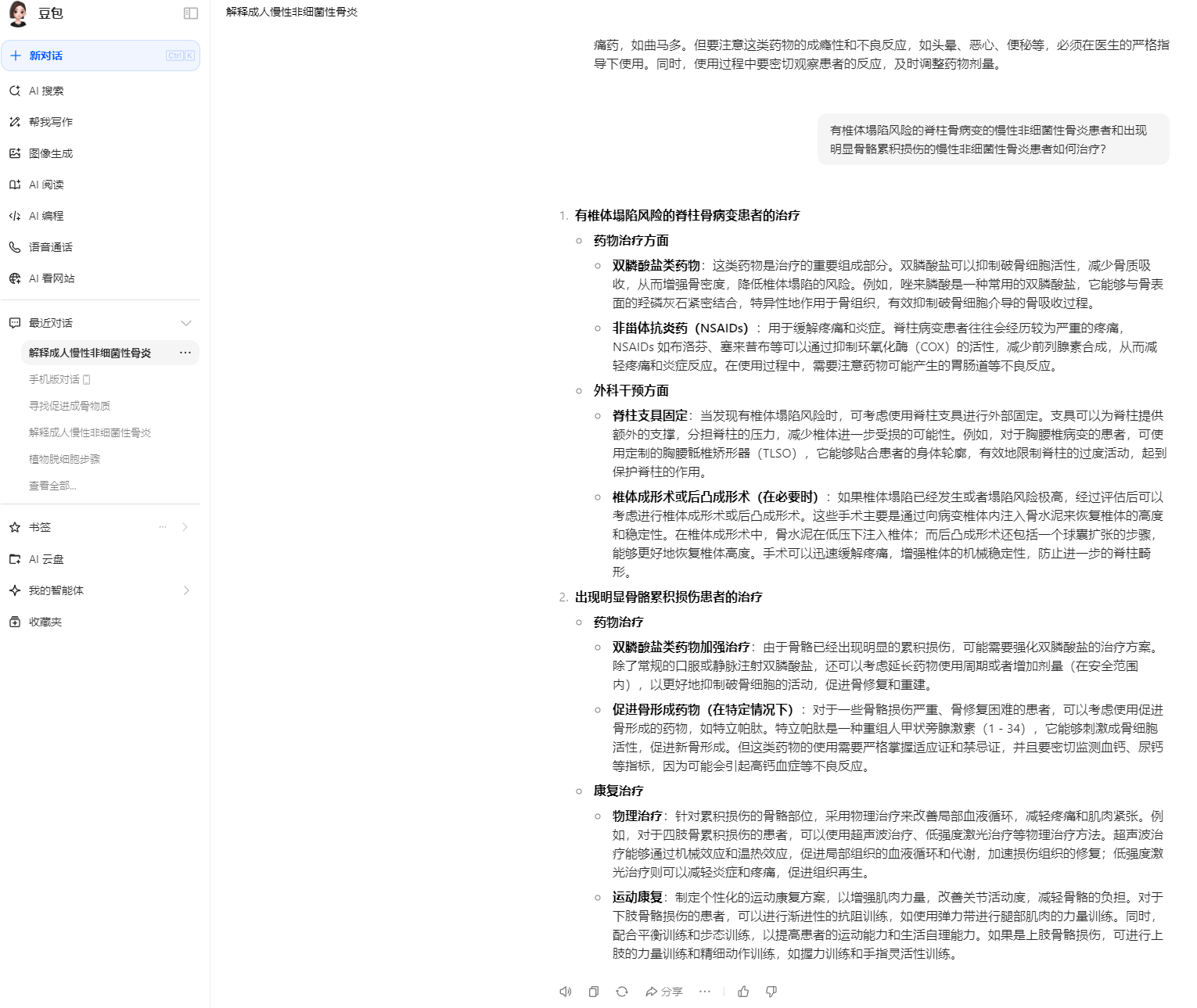
 1. **有椎体塌陷风险的脊柱骨病变患者的治疗**

- **药物治疗方面**

- **双膦酸盐类药物**：这类药物是治疗的重要组成部分。双膦酸盐可以抑制破骨细胞活性，减少骨质吸收，从而增强骨密度，降低椎体塌陷的风险。例如，唑来膦酸是一种常用的双膦酸盐，它能够与骨表面的羟磷灰石紧密结合，特异性地作用于骨组织，有效抑制破骨细胞介导的骨吸收过程。

- **非甾体抗炎药（NSAIDs）**：用于缓解疼痛和炎症。脊柱病变患者往往会经历较为严重的疼痛，NSAIDs如布洛芬、塞来昔布等可以通过抑制环氧化酶（COX）的活性，减少前列腺素合成，从而减轻疼痛和炎症反应。在使用过程中，需要注意药物可能产生的胃肠道等不良反应。

- **外科干预方面**

- **脊柱支具固定**：当发现有椎体塌陷风险时，可考虑使用脊柱支具进行外部固定。支具可以为脊柱提供额外的支撑，分担脊柱的压力，减少椎体进一步受损的可能性。例如，对于胸腰椎病变的患者，可使用定制的胸腰骶椎矫形器（TLSO），它能够贴合患者的身体轮廓，有效地限制脊柱的过度活动，起到保护脊柱的作用。

- **椎体成形术或后凸成形术（在必要时）**：如果椎体塌陷已经发生或者塌陷风险极高，经过评估后可以考虑进行椎体成形术或后凸成形术。这些手术主要是通过向病变椎体内注入骨水泥来恢复椎体的高度和稳定性。在椎体成形术中，骨水泥在低压下注入椎体；而后凸成形术还包括一个球囊扩张的步骤，能够更好地恢复椎体高度。手术可以迅速缓解疼痛，增强椎体的机械稳定性，防止进一步的脊柱畸形。

2. **出现明显骨骼累积损伤患者的治疗**

- **药物治疗**

- **双膦酸盐类药物加强治疗**：由于骨骼已经出现明显的累积损伤，可能需要强化双膦酸盐的治疗方案。除了常规的口服或静脉注射双膦酸盐，还可以考虑延长药物使用周期或者增加剂量（在安全范围内），以更好地抑制破骨细胞的活动，促进骨修复和重建。

- **促进骨形成药物（在特定情况下）**：对于一些骨骼损伤严重、骨修复困难的患者，可以考虑使用促进骨形成的药物，如特立帕肽。特立帕肽是一种重组人甲状旁腺激素（1 - 34），它能够刺激成骨细胞活性，促进新骨形成。但这类药物的使用需要严格掌握适应证和禁忌证，并且要密切监测血钙、尿钙等指标，因为可能会引起高钙血症等不良反应。

- **康复治疗**

- **物理治疗**：针对累积损伤的骨骼部位，采用物理治疗来改善局部血液循环，减轻疼痛和肌肉紧张。例如，对于四肢骨累积损伤的患者，可以使用超声波治疗、低强度激光治疗等物理治疗方法。超声波治疗能够通过机械效应和温热效应，促进局部组织的血液循环和代谢，加速损伤组织的修复；低强度激光治疗则可以减轻炎症和疼痛，促进组织再生。

- **运动康复**：制定个性化的运动康复方案，以增强肌肉力量，改善关节活动度，减轻骨骼的负担。对于下肢骨骼损伤的患者，可以进行渐进性的抗阻训练，如使用弹力带进行腿部肌肉的力量训练。同时，配合平衡训练和步态训练，以提高患者的运动能力和生活自理能力。如果是上肢骨骼损伤，可进行上肢的力量训练和精细动作训练，如握力训练和手指灵活性训练。
