# Supplementary material for: Evaluation of the accuracy and repeatability of Deepseek V3, Doubao, and Kimi1.5 in answering knowledge-related queries about chronic non-bacterial osteitis
Source: Front Artif Intell. 2025 Sep 29;8:1629149. doi: 10.3389/frai.2025.1629149 (PMC12515971; doi:10.3389/frai.2025.1629149)
Supplement: Supplementary file 1 [file Data_Sheet_1.zip › day14/day14-kimi.docx]

1成人慢性非细菌性骨炎的定义是什么？


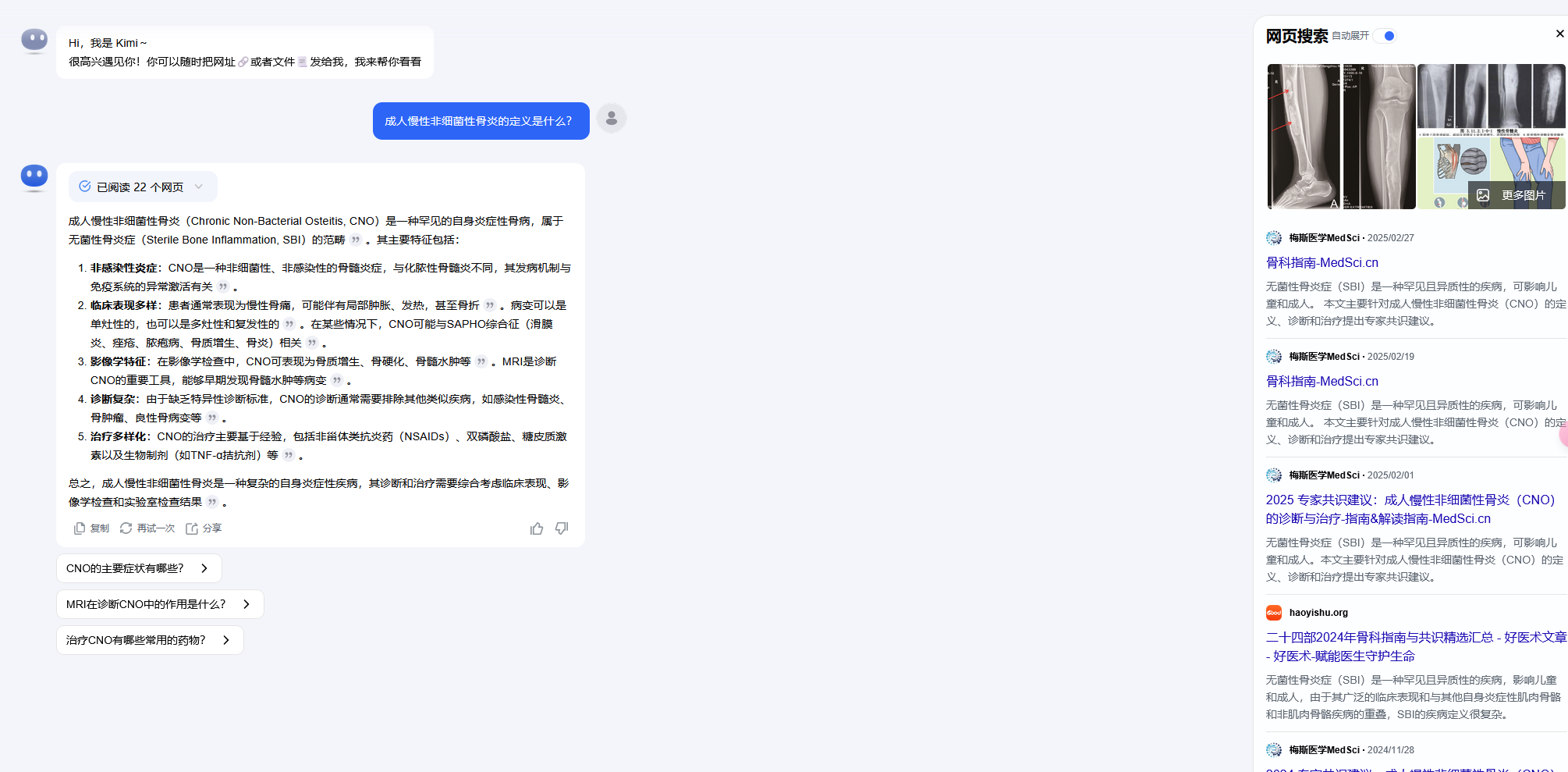


成人慢性非细菌性骨炎（Chronic Non-Bacterial Osteitis, CNO）是一种罕见的自身炎症性骨病，属于无菌性骨炎症（Sterile Bone Inflammation, SBI）的范畴。其主要特征包括：

1. **非感染性炎症**：CNO是一种非细菌性、非感染性的骨髓炎症，与化脓性骨髓炎不同，其发病机制与免疫系统的异常激活有关。

2. **临床表现多样**：患者通常表现为慢性骨痛，可能伴有局部肿胀、发热，甚至骨折。病变可以是单灶性的，也可以是多灶性和复发性的。在某些情况下，CNO可能与SAPHO综合征（滑膜炎、痤疮、脓疱病、骨质增生、骨炎）相关。

3. **影像学特征**：在影像学检查中，CNO可表现为骨质增生、骨硬化、骨髓水肿等。MRI是诊断CNO的重要工具，能够早期发现骨髓水肿等病变。

4. **诊断复杂**：由于缺乏特异性诊断标准，CNO的诊断通常需要排除其他类似疾病，如感染性骨髓炎、骨肿瘤、良性骨病变等。

5. **治疗多样化**：CNO的治疗主要基于经验，包括非甾体类抗炎药（NSAIDs）、双磷酸盐、糖皮质激素以及生物制剂（如TNF-α拮抗剂）等。

总之，成人慢性非细菌性骨炎是一种复杂的自身炎症性疾病，其诊断和治疗需要综合考虑临床表现、影像学检查和实验室检查结果。

2成人慢性非细菌性骨炎的典型影像学特征是什么？


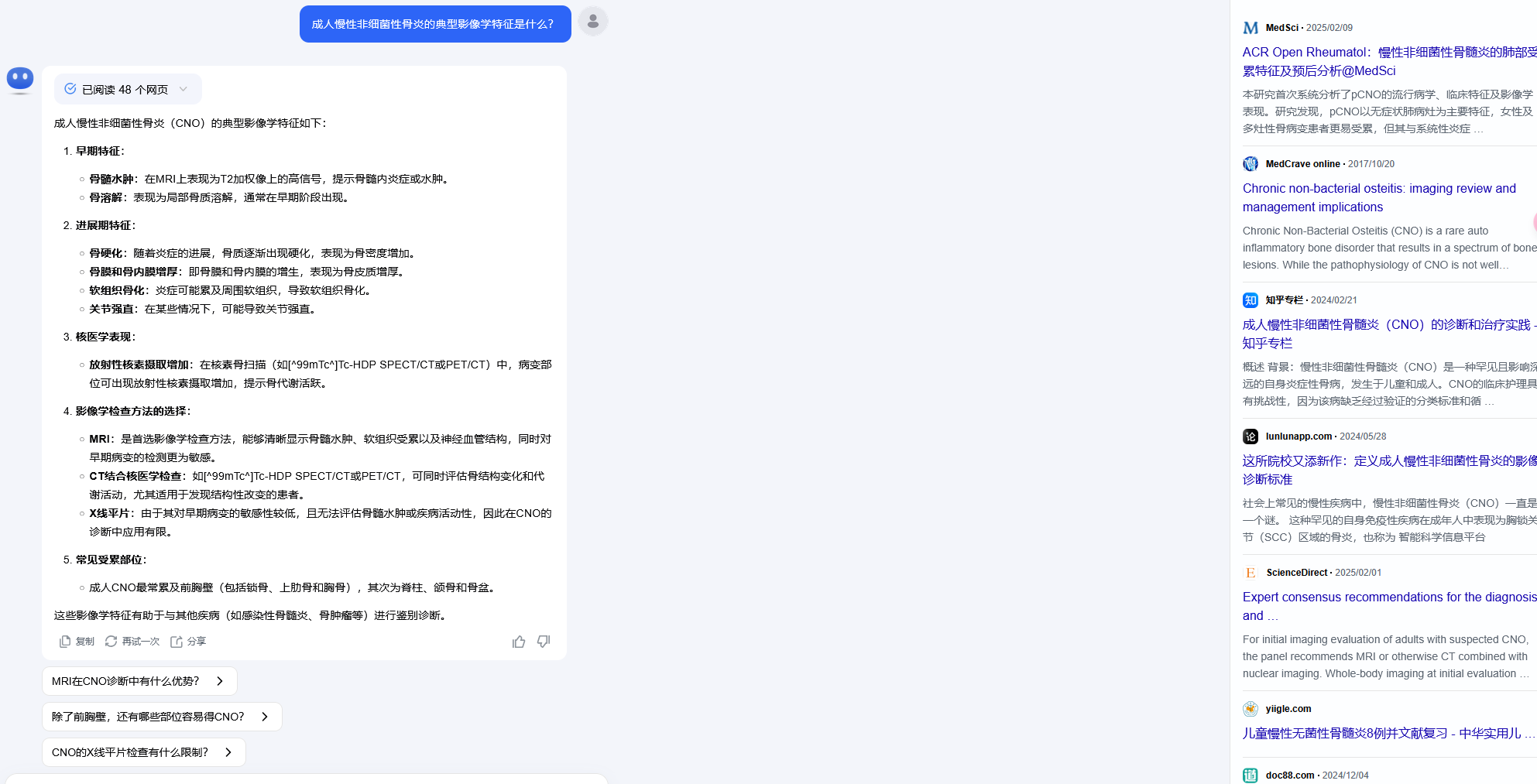


成人慢性非细菌性骨炎（CNO）的典型影像学特征如下：

1. **早期特征**：

- **骨髓水肿**：在MRI上表现为T2加权像上的高信号，提示骨髓内炎症或水肿。

- **骨溶解**：表现为局部骨质溶解，通常在早期阶段出现。

2. **进展期特征**：

- **骨硬化**：随着炎症的进展，骨质逐渐出现硬化，表现为骨密度增加。

- **骨膜和骨内膜增厚**：即骨膜和骨内膜的增生，表现为骨皮质增厚。

- **软组织骨化**：炎症可能累及周围软组织，导致软组织骨化。

- **关节强直**：在某些情况下，可能导致关节强直。

3. **核医学表现**：

- **放射性核素摄取增加**：在核素骨扫描（如[^99mTc^]Tc-HDP SPECT/CT或PET/CT）中，病变部位可出现放射性核素摄取增加，提示骨代谢活跃。

4. **影像学检查方法的选择**：

- **MRI**：是首选影像学检查方法，能够清晰显示骨髓水肿、软组织受累以及神经血管结构，同时对早期病变的检测更为敏感。

- **CT结合核医学检查**：如[^99mTc^]Tc-HDP SPECT/CT或PET/CT，可同时评估骨结构变化和代谢活动，尤其适用于发现结构性改变的患者。

- **X线平片**：由于其对早期病变的敏感性较低，且无法评估骨髓水肿或疾病活动性，因此在CNO的诊断中应用有限。

5. **常见受累部位**：

- 成人CNO最常累及前胸壁（包括锁骨、上肋骨和胸骨），其次为脊柱、颌骨和骨盆。

这些影像学特征有助于与其他疾病（如感染性骨髓炎、骨肿瘤等）进行鉴别诊断。

3成人慢性非细菌性骨炎最常见的表现是什么？


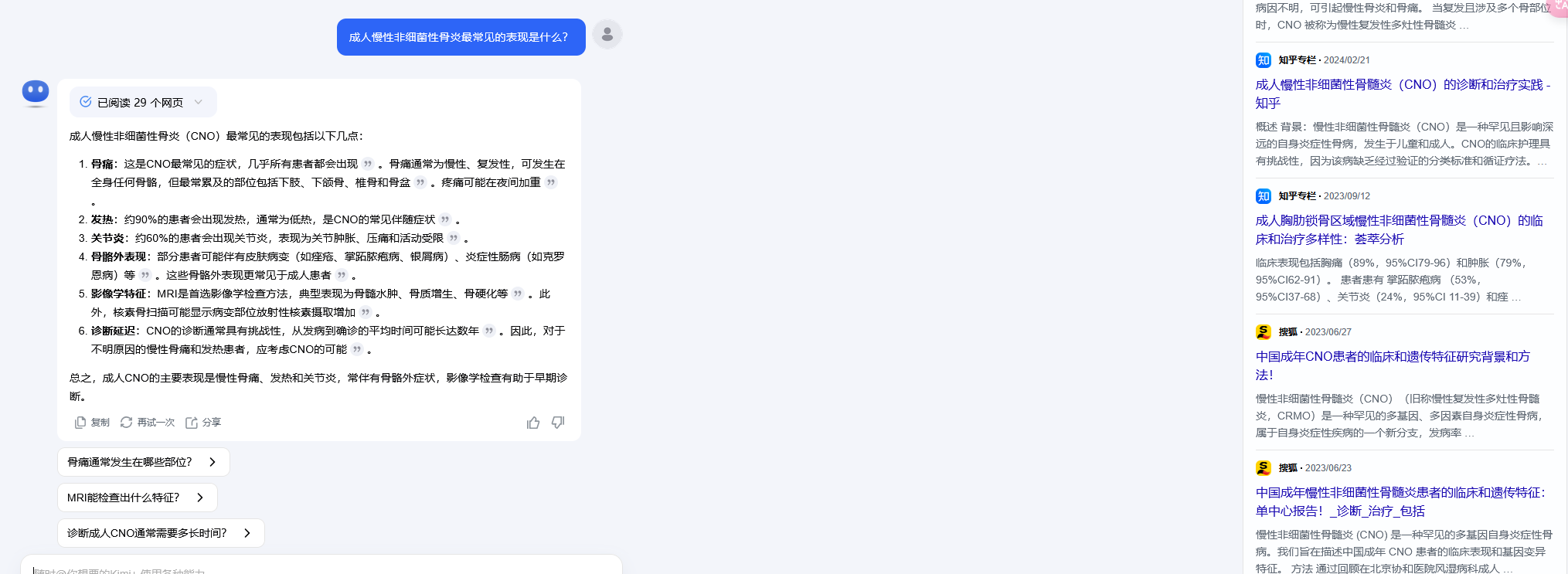


成人慢性非细菌性骨炎（CNO）最常见的表现包括以下几点：

1. **骨痛**：这是CNO最常见的症状，几乎所有患者都会出现。骨痛通常为慢性、复发性，可发生在全身任何骨骼，但最常累及的部位包括下肢、下颌骨、椎骨和骨盆。疼痛可能在夜间加重。

2. **发热**：约90%的患者会出现发热，通常为低热，是CNO的常见伴随症状。

3. **关节炎**：约60%的患者会出现关节炎，表现为关节肿胀、压痛和活动受限。

4. **骨骼外表现**：部分患者可能伴有皮肤病变（如痤疮、掌跖脓疱病、银屑病）、炎症性肠病（如克罗恩病）等。这些骨骼外表现更常见于成人患者。

5. **影像学特征**：MRI是首选影像学检查方法，典型表现为骨髓水肿、骨质增生、骨硬化等。此外，核素骨扫描可能显示病变部位放射性核素摄取增加。

6. **诊断延迟**：CNO的诊断通常具有挑战性，从发病到确诊的平均时间可能长达数年。因此，对于不明原因的慢性骨痛和发热患者，应考虑CNO的可能。

总之，成人CNO的主要表现是慢性骨痛、发热和关节炎，常伴有骨骼外症状，影像学检查有助于早期诊断。

4成人慢性非细菌性骨炎最常受累的部位是那些？


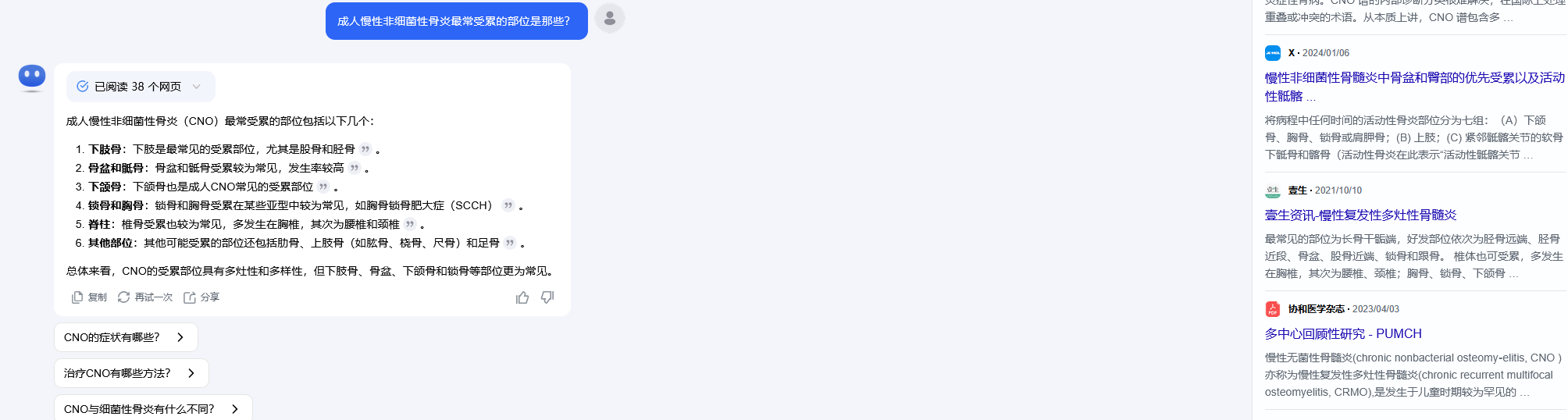


成人慢性非细菌性骨炎（CNO）最常受累的部位包括以下几个：

1. **下肢骨**：下肢是最常见的受累部位，尤其是股骨和胫骨。

2. **骨盆和骶骨**：骨盆和骶骨受累较为常见，发生率较高。

3. **下颌骨**：下颌骨也是成人CNO常见的受累部位。

4. **锁骨和胸骨**：锁骨和胸骨受累在某些亚型中较为常见，如胸骨锁骨肥大症（SCCH）。

5. **脊柱**：椎骨受累也较为常见，多发生在胸椎，其次为腰椎和颈椎。

6. **其他部位**：其他可能受累的部位还包括肋骨、上肢骨（如肱骨、桡骨、尺骨）和足骨。

总体来看，CNO的受累部位具有多灶性和多样性，但下肢骨、骨盆、下颌骨和锁骨等部位更为常见。

5成人慢性非细菌性骨炎有没有特异性的炎症标志物？


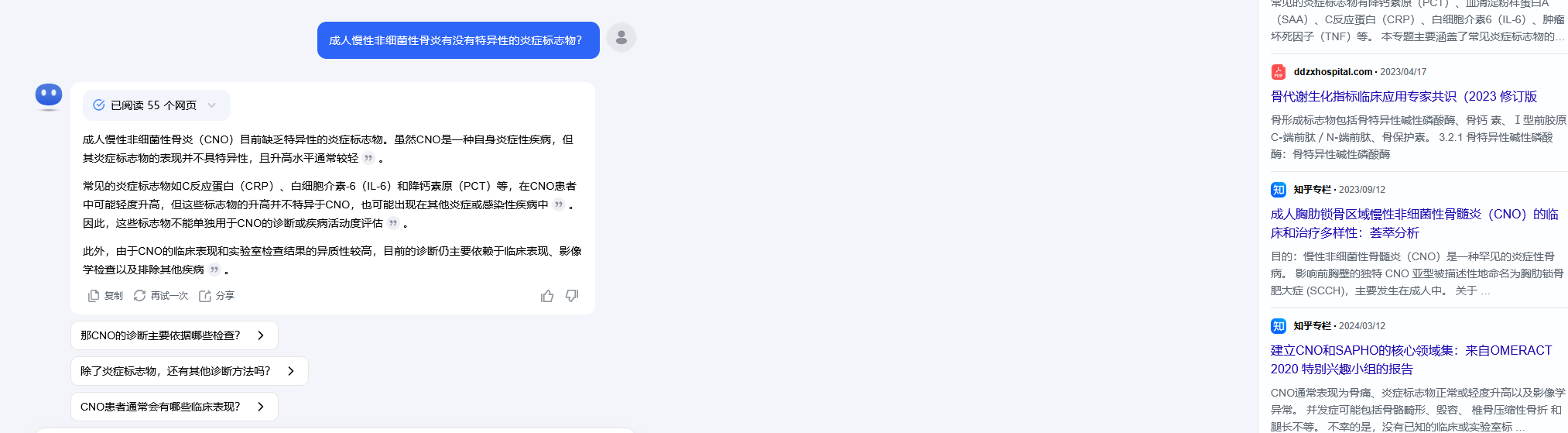


成人慢性非细菌性骨炎（CNO）目前缺乏特异性的炎症标志物。虽然CNO是一种自身炎症性疾病，但其炎症标志物的表现并不具特异性，且升高水平通常较轻。

常见的炎症标志物如C反应蛋白（CRP）、白细胞介素-6（IL-6）和降钙素原（PCT）等，在CNO患者中可能轻度升高，但这些标志物的升高并不特异于CNO，也可能出现在其他炎症或感染性疾病中。因此，这些标志物不能单独用于CNO的诊断或疾病活动度评估。

此外，由于CNO的临床表现和实验室检查结果的异质性较高，目前的诊断仍主要依赖于临床表现、影像学检查以及排除其他疾病。

6慢性非细菌性骨炎首选的影像学检查是什么？


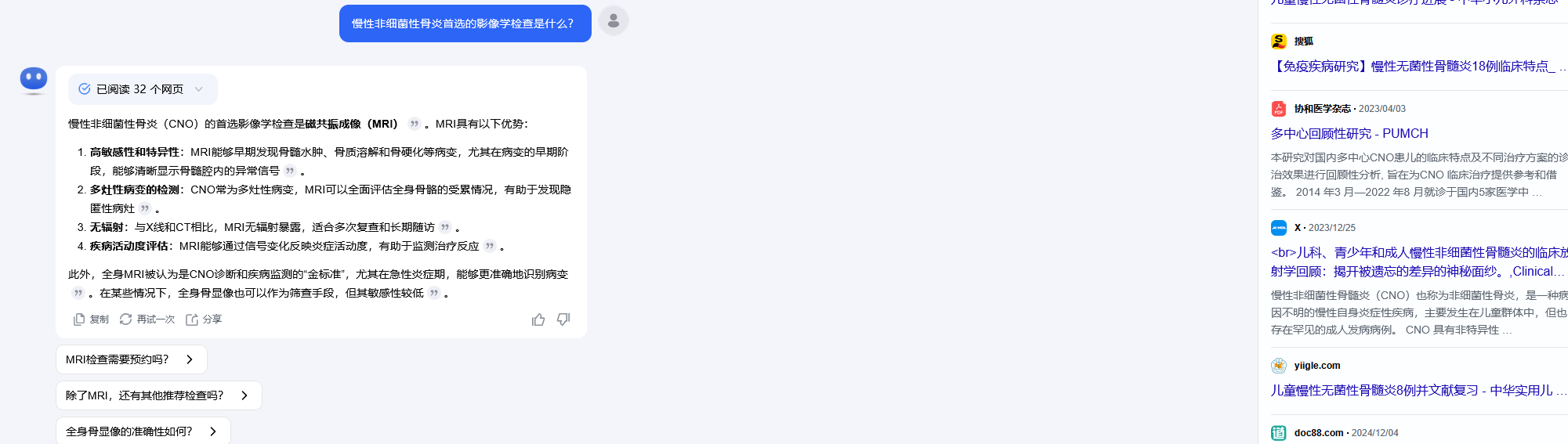


慢性非细菌性骨炎（CNO）的首选影像学检查是**磁共振成像（MRI）**。MRI具有以下优势：

1. **高敏感性和特异性**：MRI能够早期发现骨髓水肿、骨质溶解和骨硬化等病变，尤其在病变的早期阶段，能够清晰显示骨髓腔内的异常信号。

2. **多灶性病变的检测**：CNO常为多灶性病变，MRI可以全面评估全身骨骼的受累情况，有助于发现隐匿性病灶。

3. **无辐射**：与X线和CT相比，MRI无辐射暴露，适合多次复查和长期随访。

4. **疾病活动度评估**：MRI能够通过信号变化反映炎症活动度，有助于监测治疗反应。

此外，全身MRI被认为是CNO诊断和疾病监测的“金标准”，尤其在急性炎症期，能够更准确地识别病变。在某些情况下，全身骨显像也可以作为筛查手段，但其敏感性较低。

7全身成像检查是不是诊断成人慢性非细菌性骨炎的先决条件？


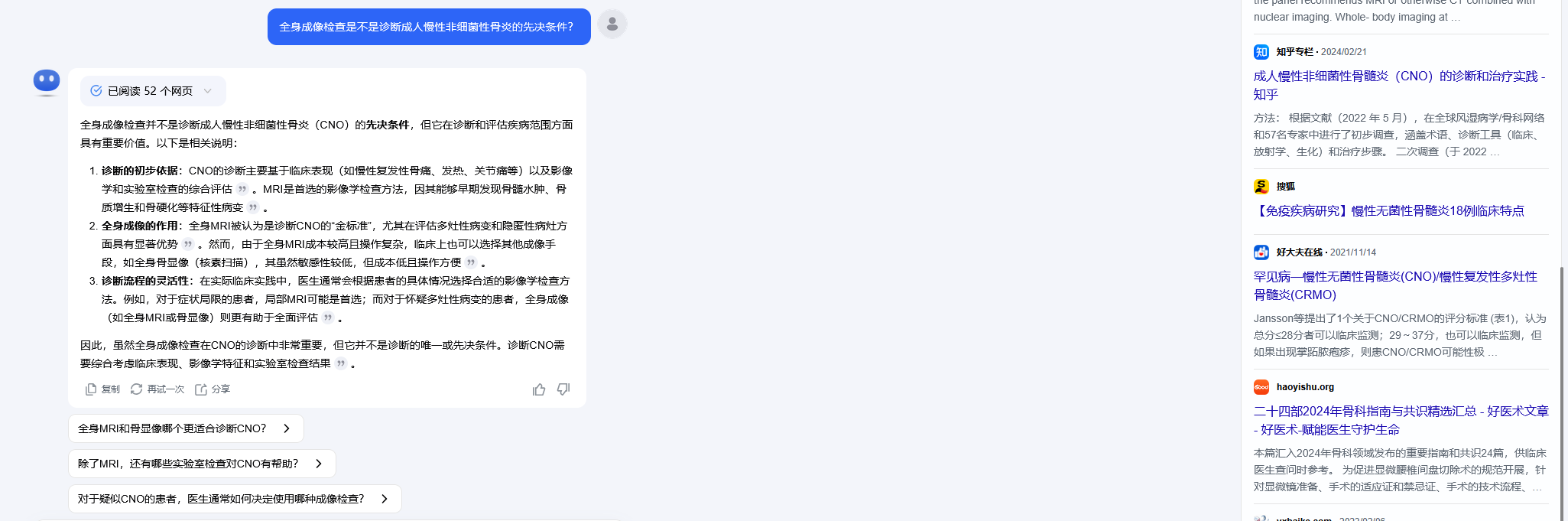


全身成像检查并不是诊断成人慢性非细菌性骨炎（CNO）的**先决条件**，但它在诊断和评估疾病范围方面具有重要价值。以下是相关说明：

1. **诊断的初步依据**：CNO的诊断主要基于临床表现（如慢性复发性骨痛、发热、关节痛等）以及影像学和实验室检查的综合评估。MRI是首选的影像学检查方法，因其能够早期发现骨髓水肿、骨质增生和骨硬化等特征性病变。

2. **全身成像的作用**：全身MRI被认为是诊断CNO的“金标准”，尤其在评估多灶性病变和隐匿性病灶方面具有显著优势。然而，由于全身MRI成本较高且操作复杂，临床上也可以选择其他成像手段，如全身骨显像（核素扫描），其虽然敏感性较低，但成本低且操作方便。

3. **诊断流程的灵活性**：在实际临床实践中，医生通常会根据患者的具体情况选择合适的影像学检查方法。例如，对于症状局限的患者，局部MRI可能是首选；而对于怀疑多灶性病变的患者，全身成像（如全身MRI或骨显像）则更有助于全面评估。

因此，虽然全身成像检查在CNO的诊断中非常重要，但它并不是诊断的唯一或先决条件。诊断CNO需要综合考虑临床表现、影像学特征和实验室检查结果。

8诊断成人慢性非细菌性骨炎有必要常规骨活检吗？


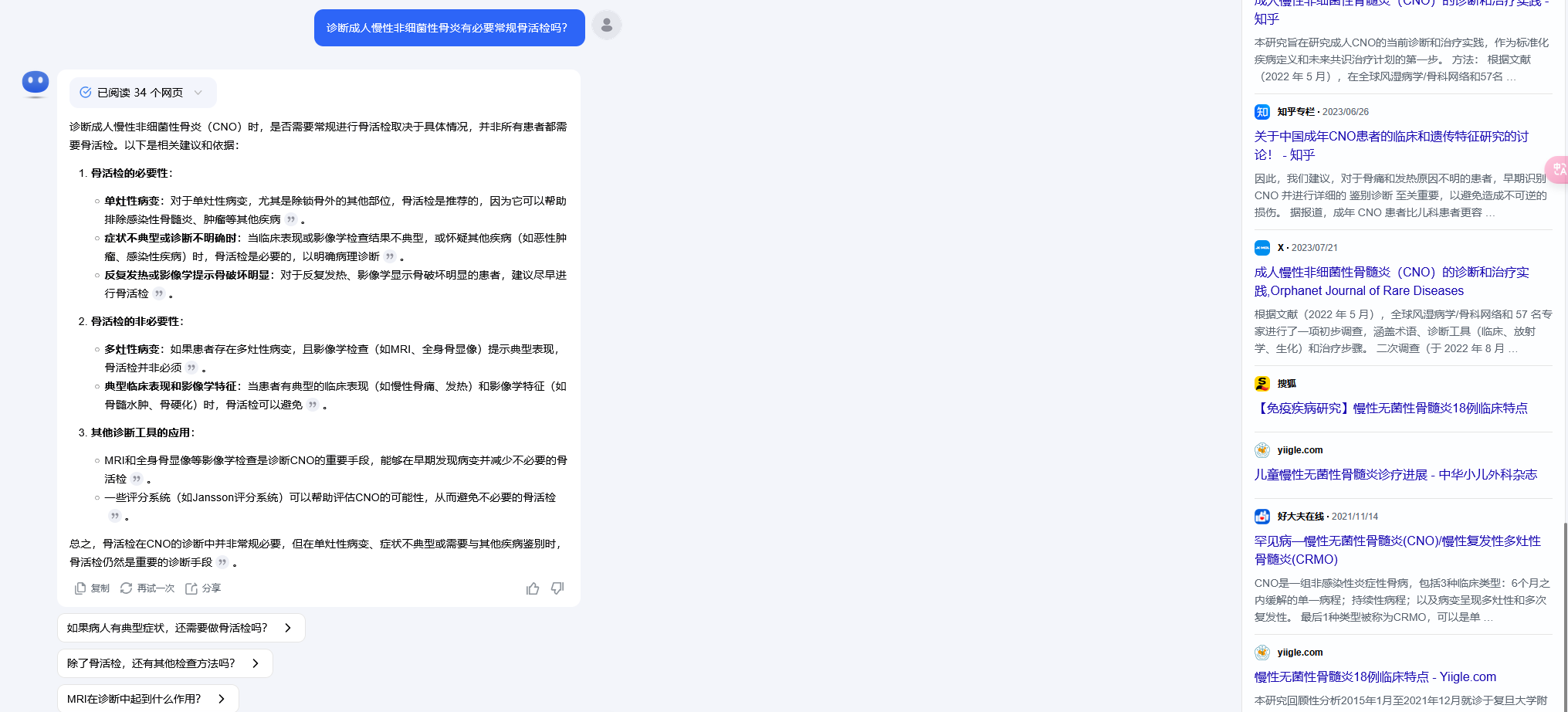


诊断成人慢性非细菌性骨炎（CNO）时，是否需要常规进行骨活检取决于具体情况，并非所有患者都需要骨活检。以下是相关建议和依据：

1. **骨活检的必要性**：

- **单灶性病变**：对于单灶性病变，尤其是除锁骨外的其他部位，骨活检是推荐的，因为它可以帮助排除感染性骨髓炎、肿瘤等其他疾病。

- **症状不典型或诊断不明确时**：当临床表现或影像学检查结果不典型，或怀疑其他疾病（如恶性肿瘤、感染性疾病）时，骨活检是必要的，以明确病理诊断。

- **反复发热或影像学提示骨破坏明显**：对于反复发热、影像学显示骨破坏明显的患者，建议尽早进行骨活检。

2. **骨活检的非必要性**：

- **多灶性病变**：如果患者存在多灶性病变，且影像学检查（如MRI、全身骨显像）提示典型表现，骨活检并非必须。

- **典型临床表现和影像学特征**：当患者有典型的临床表现（如慢性骨痛、发热）和影像学特征（如骨髓水肿、骨硬化）时，骨活检可以避免。

3. **其他诊断工具的应用**：

- MRI和全身骨显像等影像学检查是诊断CNO的重要手段，能够在早期发现病变并减少不必要的骨活检。

- 一些评分系统（如Jansson评分系统）可以帮助评估CNO的可能性，从而避免不必要的骨活检。

总之，骨活检在CNO的诊断中并非常规必要，但在单灶性病变、症状不典型或需要与其他疾病鉴别时，骨活检仍然是重要的诊断手段。

9慢性非细菌性骨炎如何与恶性骨肿瘤鉴别？


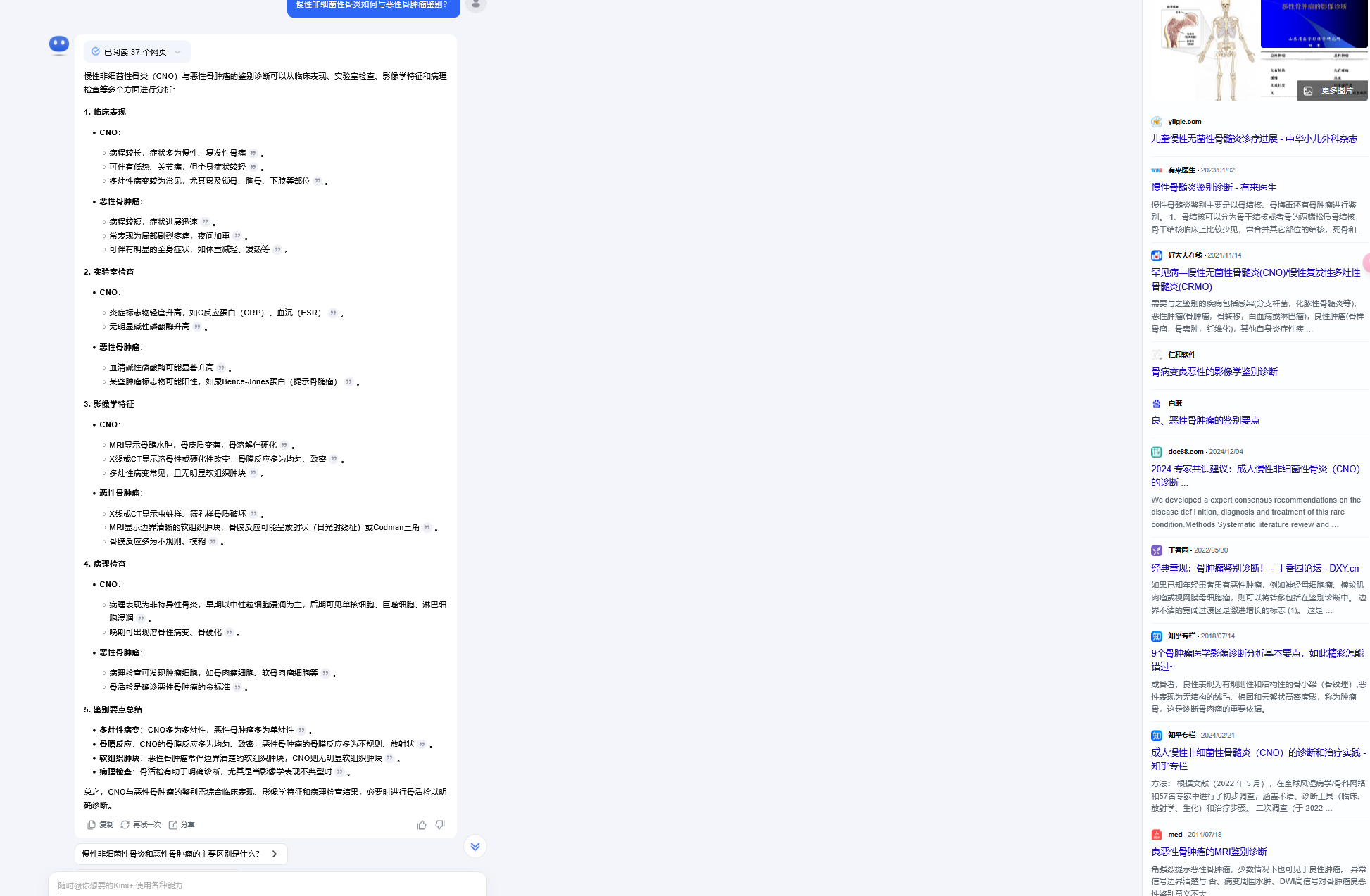


慢性非细菌性骨炎（CNO）与恶性骨肿瘤的鉴别诊断可以从临床表现、实验室检查、影像学特征和病理检查等多个方面进行分析：

### 1. 临床表现

- **CNO**：

- 病程较长，症状多为慢性、复发性骨痛。

- 可伴有低热、关节痛，但全身症状较轻。

- 多灶性病变较为常见，尤其累及锁骨、胸骨、下肢等部位。

- **恶性骨肿瘤**：

- 病程较短，症状进展迅速。

- 常表现为局部剧烈疼痛，夜间加重。

- 可伴有明显的全身症状，如体重减轻、发热等。

### 2. 实验室检查

- **CNO**：

- 炎症标志物轻度升高，如C反应蛋白（CRP）、血沉（ESR）。

- 无明显碱性磷酸酶升高。

- **恶性骨肿瘤**：

- 血清碱性磷酸酶可能显著升高。

- 某些肿瘤标志物可能阳性，如尿Bence-Jones蛋白（提示骨髓瘤）。

### 3. 影像学特征

- **CNO**：

- MRI显示骨髓水肿，骨皮质变薄，骨溶解伴硬化。

- X线或CT显示溶骨性或硬化性改变，骨膜反应多为均匀、致密。

- 多灶性病变常见，且无明显软组织肿块。

- **恶性骨肿瘤**：

- X线或CT显示虫蛀样、筛孔样骨质破坏。

- MRI显示边界清晰的软组织肿块，骨膜反应可能呈放射状（日光射线征）或Codman三角。

- 骨膜反应多为不规则、模糊。

### 4. 病理检查

- **CNO**：

- 病理表现为非特异性骨炎，早期以中性粒细胞浸润为主，后期可见单核细胞、巨噬细胞、淋巴细胞浸润。

- 晚期可出现溶骨性病变、骨硬化。

- **恶性骨肿瘤**：

- 病理检查可发现肿瘤细胞，如骨肉瘤细胞、软骨肉瘤细胞等。

- 骨活检是确诊恶性骨肿瘤的金标准。

### 5. 鉴别要点总结

- **多灶性病变**：CNO多为多灶性，恶性骨肿瘤多为单灶性。

- **骨膜反应**：CNO的骨膜反应多为均匀、致密；恶性骨肿瘤的骨膜反应多为不规则、放射状。

- **软组织肿块**：恶性骨肿瘤常伴边界清楚的软组织肿块，CNO则无明显软组织肿块。

- **病理检查**：骨活检有助于明确诊断，尤其是当影像学表现不典型时。

总之，CNO与恶性骨肿瘤的鉴别需综合临床表现、影像学特征和病理检查结果，必要时进行骨活检以明确诊断。

10慢性非细菌性骨炎如何与骨关节炎鉴别？


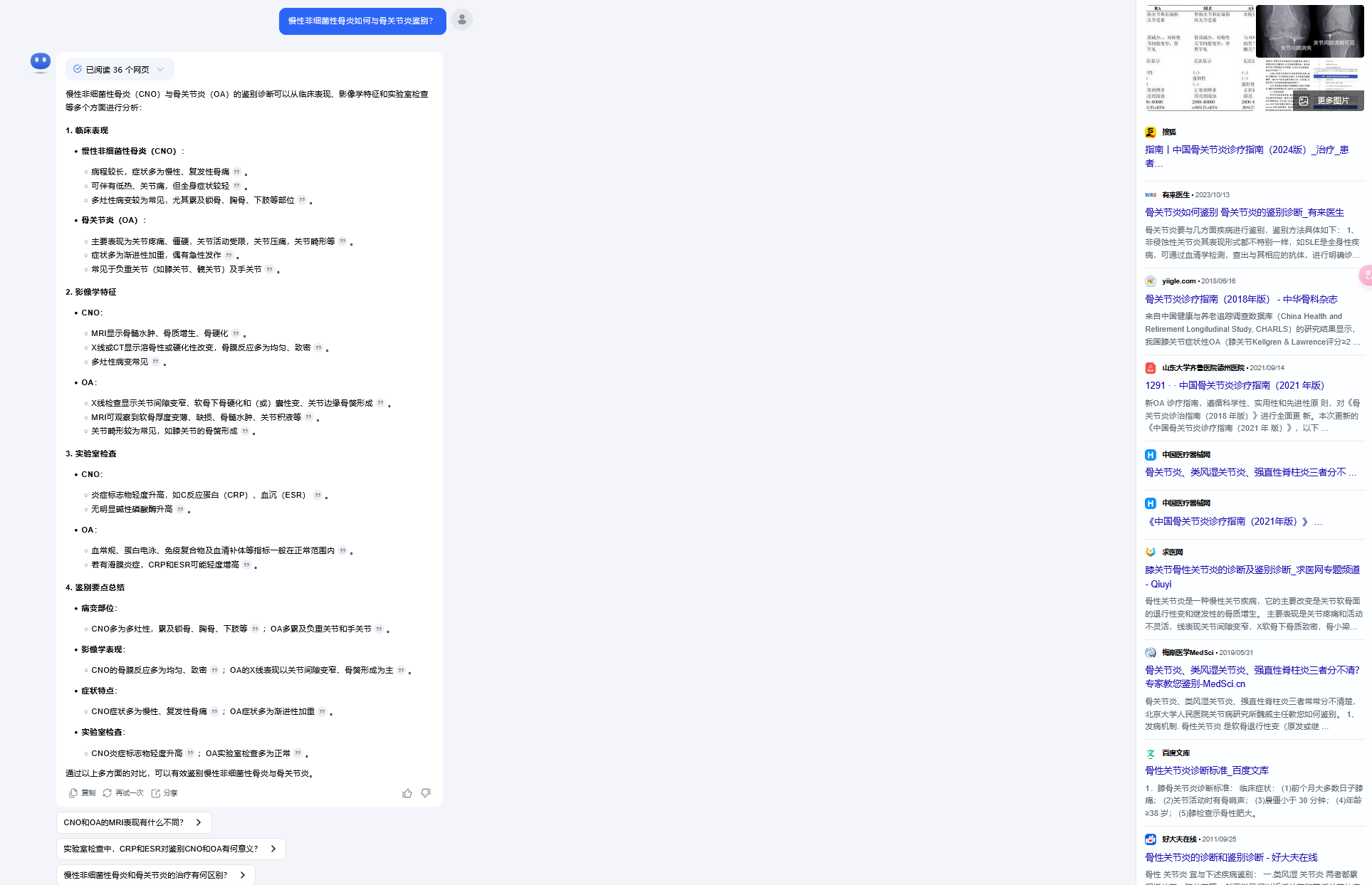


慢性非细菌性骨炎（CNO）与骨关节炎（OA）的鉴别诊断可以从临床表现、影像学特征和实验室检查等多个方面进行分析：

### 1. 临床表现

- **慢性非细菌性骨炎（CNO）**：

- 病程较长，症状多为慢性、复发性骨痛。

- 可伴有低热、关节痛，但全身症状较轻。

- 多灶性病变较为常见，尤其累及锁骨、胸骨、下肢等部位。

- **骨关节炎（OA）**：

- 主要表现为关节疼痛、僵硬，关节活动受限，关节压痛，关节畸形等。

- 症状多为渐进性加重，偶有急性发作。

- 常见于负重关节（如膝关节、髋关节）及手关节。

### 2. 影像学特征

- **CNO**：

- MRI显示骨髓水肿、骨质增生、骨硬化。

- X线或CT显示溶骨性或硬化性改变，骨膜反应多为均匀、致密。

- 多灶性病变常见。

- **OA**：

- X线检查显示关节间隙变窄、软骨下骨硬化和（或）囊性变、关节边缘骨赘形成。

- MRI可观察到软骨厚度变薄、缺损、骨髓水肿、关节积液等。

- 关节畸形较为常见，如膝关节的骨赘形成。

### 3. 实验室检查

- **CNO**：

- 炎症标志物轻度升高，如C反应蛋白（CRP）、血沉（ESR）。

- 无明显碱性磷酸酶升高。

- **OA**：

- 血常规、蛋白电泳、免疫复合物及血清补体等指标一般在正常范围内。

- 若有滑膜炎症，CRP和ESR可能轻度增高。

### 4. 鉴别要点总结

- **病变部位**：

- CNO多为多灶性，累及锁骨、胸骨、下肢等；OA多累及负重关节和手关节。

- **影像学表现**：

- CNO的骨膜反应多为均匀、致密；OA的X线表现以关节间隙变窄、骨赘形成为主。

- **症状特点**：

- CNO症状多为慢性、复发性骨痛；OA症状多为渐进性加重。

- **实验室检查**：

- CNO炎症标志物轻度升高；OA实验室检查多为正常。

通过以上多方面的对比，可以有效鉴别慢性非细菌性骨炎与骨关节炎。

11慢性非细菌性骨炎如何与纤维发育不良鉴别？


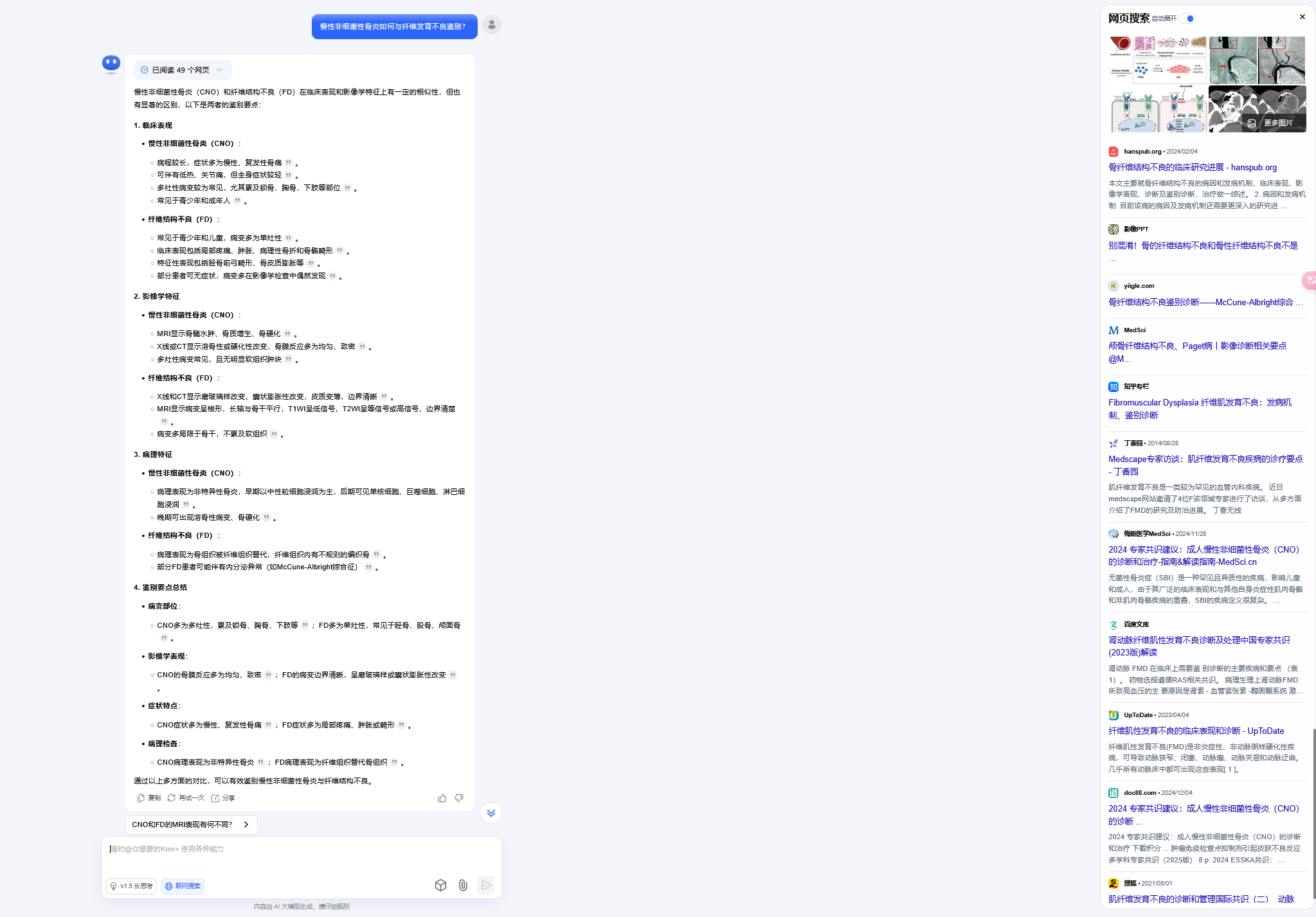


慢性非细菌性骨炎（CNO）和纤维结构不良（FD）在临床表现和影像学特征上有一定的相似性，但也有显著的区别，以下是两者的鉴别要点：

### 1. **临床表现**

- **慢性非细菌性骨炎（CNO）**：

- 病程较长，症状多为慢性、复发性骨痛。

- 可伴有低热、关节痛，但全身症状较轻。

- 多灶性病变较为常见，尤其累及锁骨、胸骨、下肢等部位。

- 常见于青少年和成年人。

- **纤维结构不良（FD）**：

- 常见于青少年和儿童，病变多为单灶性。

- 临床表现包括局部疼痛、肿胀、病理性骨折和骨骼畸形。

- 特征性表现包括胫骨前弓畸形、骨皮质膨胀等。

- 部分患者可无症状，病变多在影像学检查中偶然发现。

### 2. **影像学特征**

- **慢性非细菌性骨炎（CNO）**：

- MRI显示骨髓水肿、骨质增生、骨硬化。

- X线或CT显示溶骨性或硬化性改变，骨膜反应多为均匀、致密。

- 多灶性病变常见，且无明显软组织肿块。

- **纤维结构不良（FD）**：

- X线和CT显示磨玻璃样改变、囊状膨胀性改变，皮质变薄，边界清晰。

- MRI显示病变呈梭形，长轴与骨干平行，T1WI呈低信号，T2WI呈等信号或高信号，边界清楚。

- 病变多局限于骨干，不累及软组织。

### 3. **病理特征**

- **慢性非细菌性骨炎（CNO）**：

- 病理表现为非特异性骨炎，早期以中性粒细胞浸润为主，后期可见单核细胞、巨噬细胞、淋巴细胞浸润。

- 晚期可出现溶骨性病变、骨硬化。

- **纤维结构不良（FD）**：

- 病理表现为骨组织被纤维组织替代，纤维组织内有不规则的编织骨。

- 部分FD患者可能伴有内分泌异常（如McCune-Albright综合征）。

### 4. **鉴别要点总结**

- **病变部位**：

- CNO多为多灶性，累及锁骨、胸骨、下肢等；FD多为单灶性，常见于胫骨、股骨、颅面骨。

- **影像学表现**：

- CNO的骨膜反应多为均匀、致密；FD的病变边界清晰，呈磨玻璃样或囊状膨胀性改变。

- **症状特点**：

- CNO症状多为慢性、复发性骨痛；FD症状多为局部疼痛、肿胀或畸形。

- **病理检查**：

- CNO病理表现为非特异性骨炎；FD病理表现为纤维组织替代骨组织。

通过以上多方面的对比，可以有效鉴别慢性非细菌性骨炎与纤维结构不良。

12在治慢性非细菌性骨炎过程中应该选择短疗程的糖皮质激素治疗还是长期的糖皮质激素治疗？
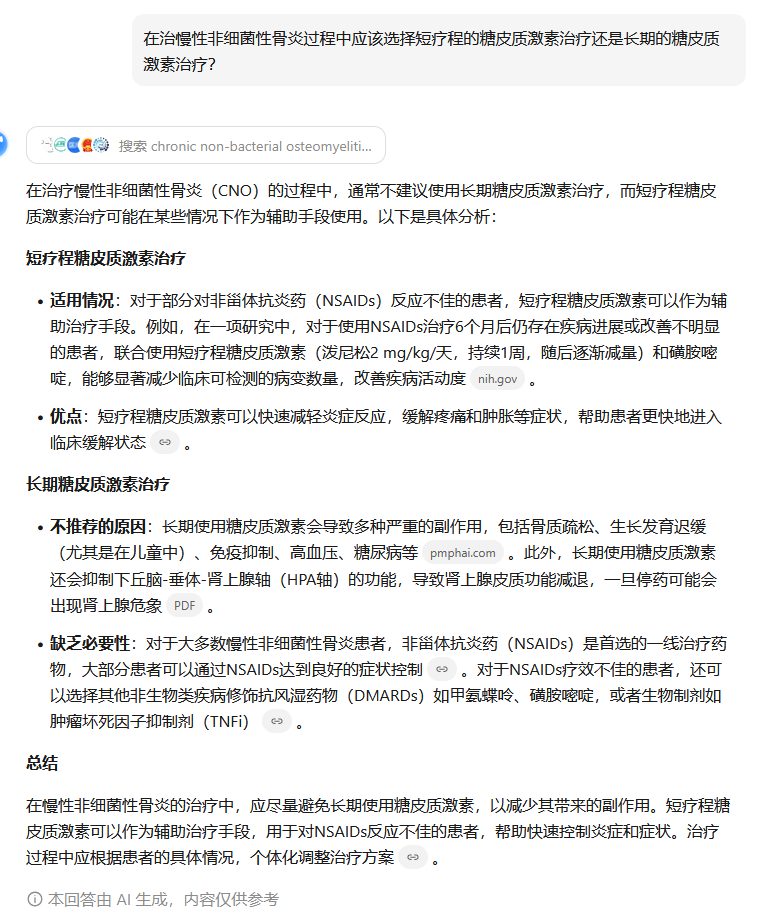


在治疗慢性非细菌性骨炎（CNO）的过程中，通常不建议使用长期糖皮质激素治疗，而短疗程糖皮质激素治疗可能在某些情况下作为辅助手段使用。以下是具体分析：

### 短疗程糖皮质激素治疗

- **适用情况**：对于部分对非甾体抗炎药（NSAIDs）反应不佳的患者，短疗程糖皮质激素可以作为辅助治疗手段。例如，在一项研究中，对于使用NSAIDs治疗6个月后仍存在疾病进展或改善不明显的患者，联合使用短疗程糖皮质激素（泼尼松2 mg/kg/天，持续1周，随后逐渐减量）和磺胺嘧啶，能够显著减少临床可检测的病变数量，改善疾病活动度。

- **优点**：短疗程糖皮质激素可以快速减轻炎症反应，缓解疼痛和肿胀等症状，帮助患者更快地进入临床缓解状态。

### 长期糖皮质激素治疗

- **不推荐的原因**：长期使用糖皮质激素会导致多种严重的副作用，包括骨质疏松、生长发育迟缓（尤其是在儿童中）、免疫抑制、高血压、糖尿病等。此外，长期使用糖皮质激素还会抑制下丘脑-垂体-肾上腺轴（HPA轴）的功能，导致肾上腺皮质功能减退，一旦停药可能会出现肾上腺危象。

- **缺乏必要性**：对于大多数慢性非细菌性骨炎患者，非甾体抗炎药（NSAIDs）是首选的一线治疗药物，大部分患者可以通过NSAIDs达到良好的症状控制。对于NSAIDs疗效不佳的患者，还可以选择其他非生物类疾病修饰抗风湿药物（DMARDs）如甲氨蝶呤、磺胺嘧啶，或者生物制剂如肿瘤坏死因子抑制剂（TNFi）。

### 总结

在慢性非细菌性骨炎的治疗中，应尽量避免长期使用糖皮质激素，以减少其带来的副作用。短疗程糖皮质激素可以作为辅助治疗手段，用于对NSAIDs反应不佳的患者，帮助快速控制炎症和症状。治疗过程中应根据患者的具体情况，个体化调整治疗方案。

13成人慢性非细菌性骨炎一线治疗方案是什么？


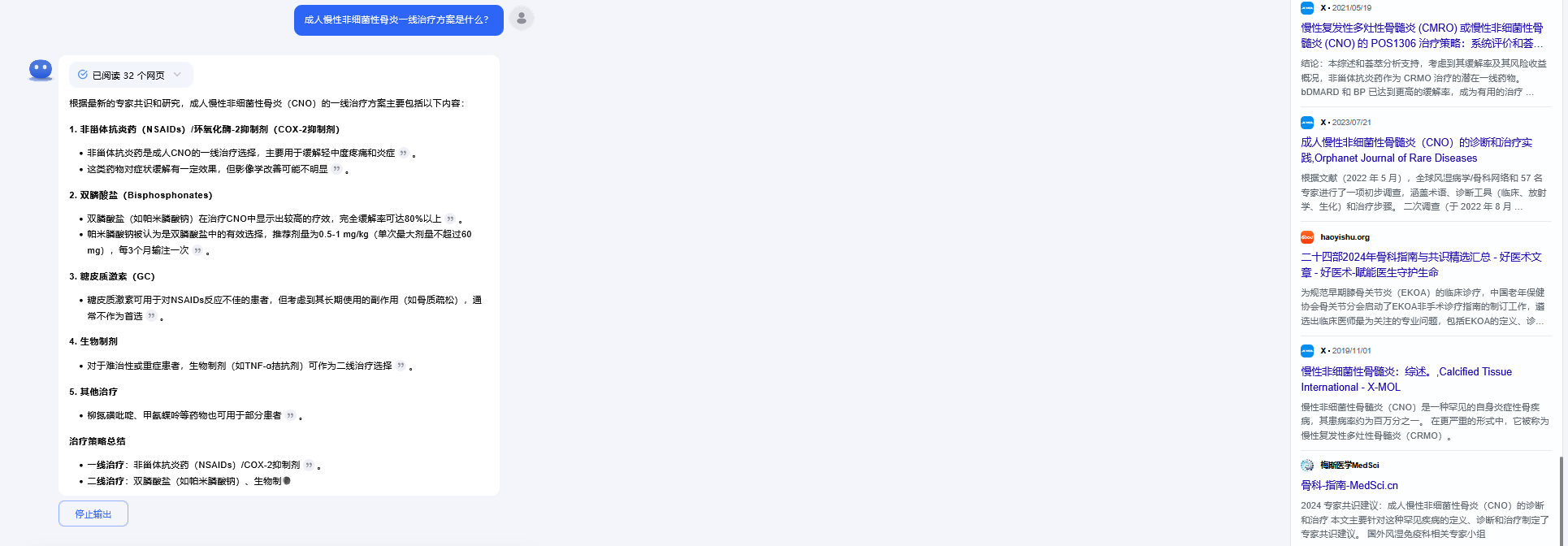


根据最新的专家共识和研究，成人慢性非细菌性骨炎（CNO）的一线治疗方案主要包括以下内容：

### 1. **非甾体抗炎药（NSAIDs）/环氧化酶-2抑制剂（COX-2抑制剂）**

- 非甾体抗炎药是成人CNO的一线治疗选择，主要用于缓解轻中度疼痛和炎症。

- 这类药物对症状缓解有一定效果，但影像学改善可能不明显。

### 2. **双膦酸盐（Bisphosphonates）**

- 双膦酸盐（如帕米膦酸钠）在治疗CNO中显示出较高的疗效，完全缓解率可达80%以上。

- 帕米膦酸钠被认为是双膦酸盐中的有效选择，推荐剂量为0.5-1 mg/kg（单次最大剂量不超过60 mg），每3个月输注一次。

### 3. **糖皮质激素（GC）**

- 糖皮质激素可用于对NSAIDs反应不佳的患者，但考虑到其长期使用的副作用（如骨质疏松），通常不作为首选。

### 4. **生物制剂**

- 对于难治性或重症患者，生物制剂（如TNF-α拮抗剂）可作为二线治疗选择。

### 5. **其他治疗**

- 柳氮磺吡啶、甲氨蝶呤等药物也可用于部分患者。

### 治疗策略总结

- **一线治疗**：非甾体抗炎药（NSAIDs）/COX-2抑制剂。

- **二线治疗**：双膦酸盐（如帕米膦酸钠）、生物制剂（如TNF-α拮抗剂）。

- **其他**：糖皮质激素可作为短期或难治性病例的补充治疗。

治疗方案应根据患者的具体病情、症状严重程度以及对药物的耐受性进行个体化调整。建议在专业医生的指导下进行治疗。

14无临床症状，也没有放射学疾病活动的慢性非细菌性骨炎的病人需要治疗吗？


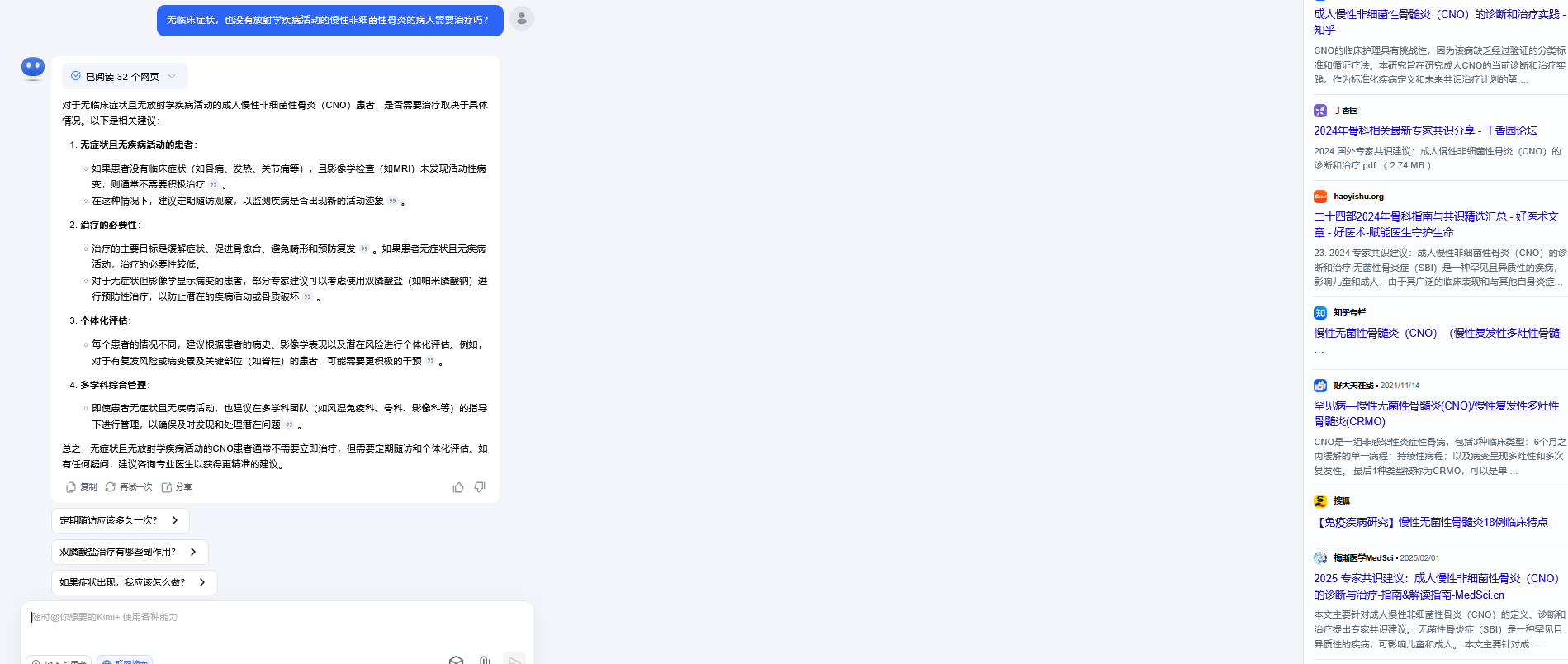


对于无临床症状且无放射学疾病活动的成人慢性非细菌性骨炎（CNO）患者，是否需要治疗取决于具体情况。以下是相关建议：

1. **无症状且无疾病活动的患者**：

- 如果患者没有临床症状（如骨痛、发热、关节痛等），且影像学检查（如MRI）未发现活动性病变，则通常不需要积极治疗。

- 在这种情况下，建议定期随访观察，以监测疾病是否出现新的活动迹象。

2. **治疗的必要性**：

- 治疗的主要目标是缓解症状、促进骨愈合、避免畸形和预防复发。如果患者无症状且无疾病活动，治疗的必要性较低。

- 对于无症状但影像学显示病变的患者，部分专家建议可以考虑使用双膦酸盐（如帕米膦酸钠）进行预防性治疗，以防止潜在的疾病活动或骨质破坏。

3. **个体化评估**：

- 每个患者的情况不同，建议根据患者的病史、影像学表现以及潜在风险进行个体化评估。例如，对于有复发风险或病变累及关键部位（如脊柱）的患者，可能需要更积极的干预。

4. **多学科综合管理**：

- 即使患者无症状且无疾病活动，也建议在多学科团队（如风湿免疫科、骨科、影像科等）的指导下进行管理，以确保及时发现和处理潜在问题。

总之，无症状且无放射学疾病活动的CNO患者通常不需要立即治疗，但需要定期随访和个体化评估。如有任何疑问，建议咨询专业医生以获得更精准的建议。

15有临床症状没有放射学疾病活动的慢性非细菌性骨炎的病人如何处理？


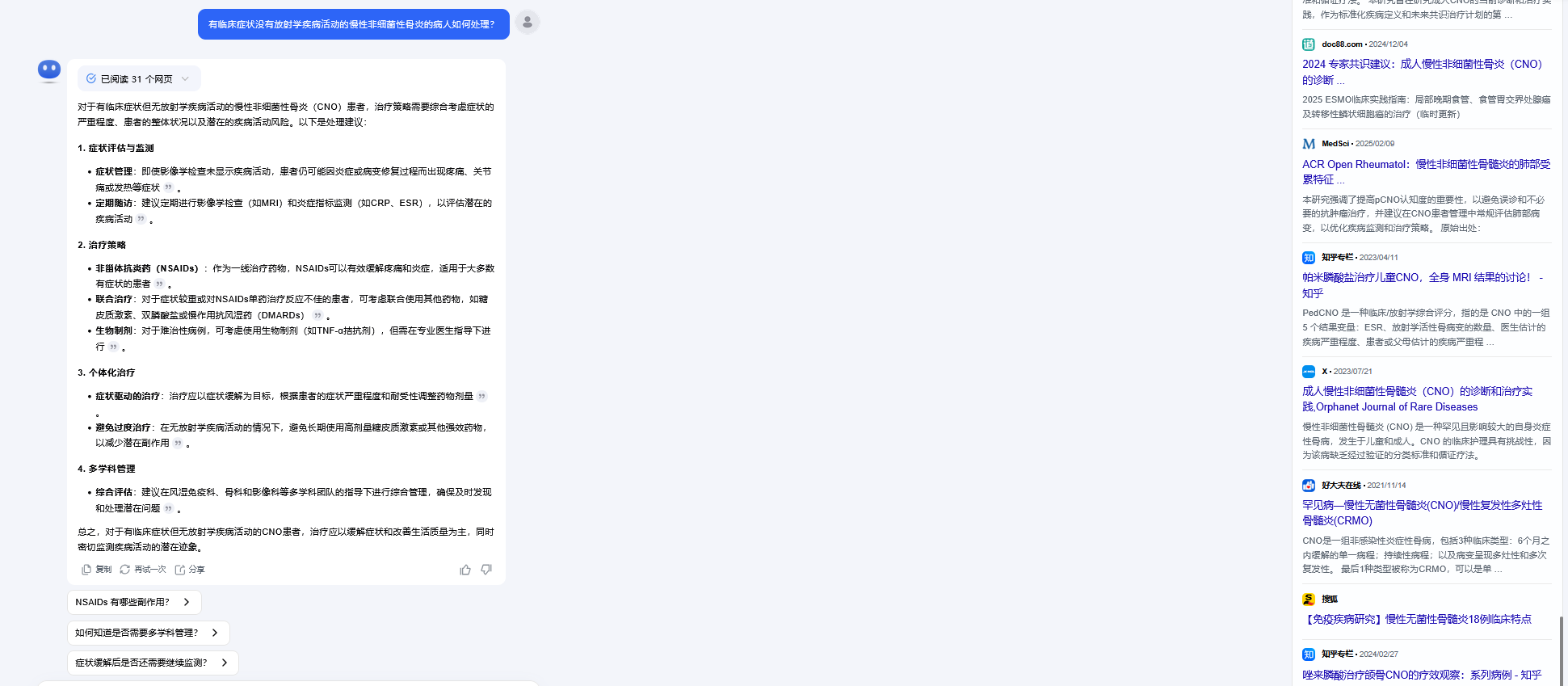


对于有临床症状但无放射学疾病活动的慢性非细菌性骨炎（CNO）患者，治疗策略需要综合考虑症状的严重程度、患者的整体状况以及潜在的疾病活动风险。以下是处理建议：

### 1. **症状评估与监测**

- **症状管理**：即使影像学检查未显示疾病活动，患者仍可能因炎症或病变修复过程而出现疼痛、关节痛或发热等症状。

- **定期随访**：建议定期进行影像学检查（如MRI）和炎症指标监测（如CRP、ESR），以评估潜在的疾病活动。

### 2. **治疗策略**

- **非甾体抗炎药（NSAIDs）**：作为一线治疗药物，NSAIDs可以有效缓解疼痛和炎症，适用于大多数有症状的患者。

- **联合治疗**：对于症状较重或对NSAIDs单药治疗反应不佳的患者，可考虑联合使用其他药物，如糖皮质激素、双膦酸盐或慢作用抗风湿药（DMARDs）。

- **生物制剂**：对于难治性病例，可考虑使用生物制剂（如TNF-α拮抗剂），但需在专业医生指导下进行。

### 3. **个体化治疗**

- **症状驱动的治疗**：治疗应以症状缓解为目标，根据患者的症状严重程度和耐受性调整药物剂量。

- **避免过度治疗**：在无放射学疾病活动的情况下，避免长期使用高剂量糖皮质激素或其他强效药物，以减少潜在副作用。

### 4. **多学科管理**

- **综合评估**：建议在风湿免疫科、骨科和影像科等多学科团队的指导下进行综合管理，确保及时发现和处理潜在问题。

总之，对于有临床症状但无放射学疾病活动的CNO患者，治疗应以缓解症状和改善生活质量为主，同时密切监测疾病活动的潜在迹象。

16有椎体塌陷风险的脊柱骨病变的慢性非细菌性骨炎患者和出现明显骨骼累积损伤的慢性非细菌性骨炎患者如何治疗？


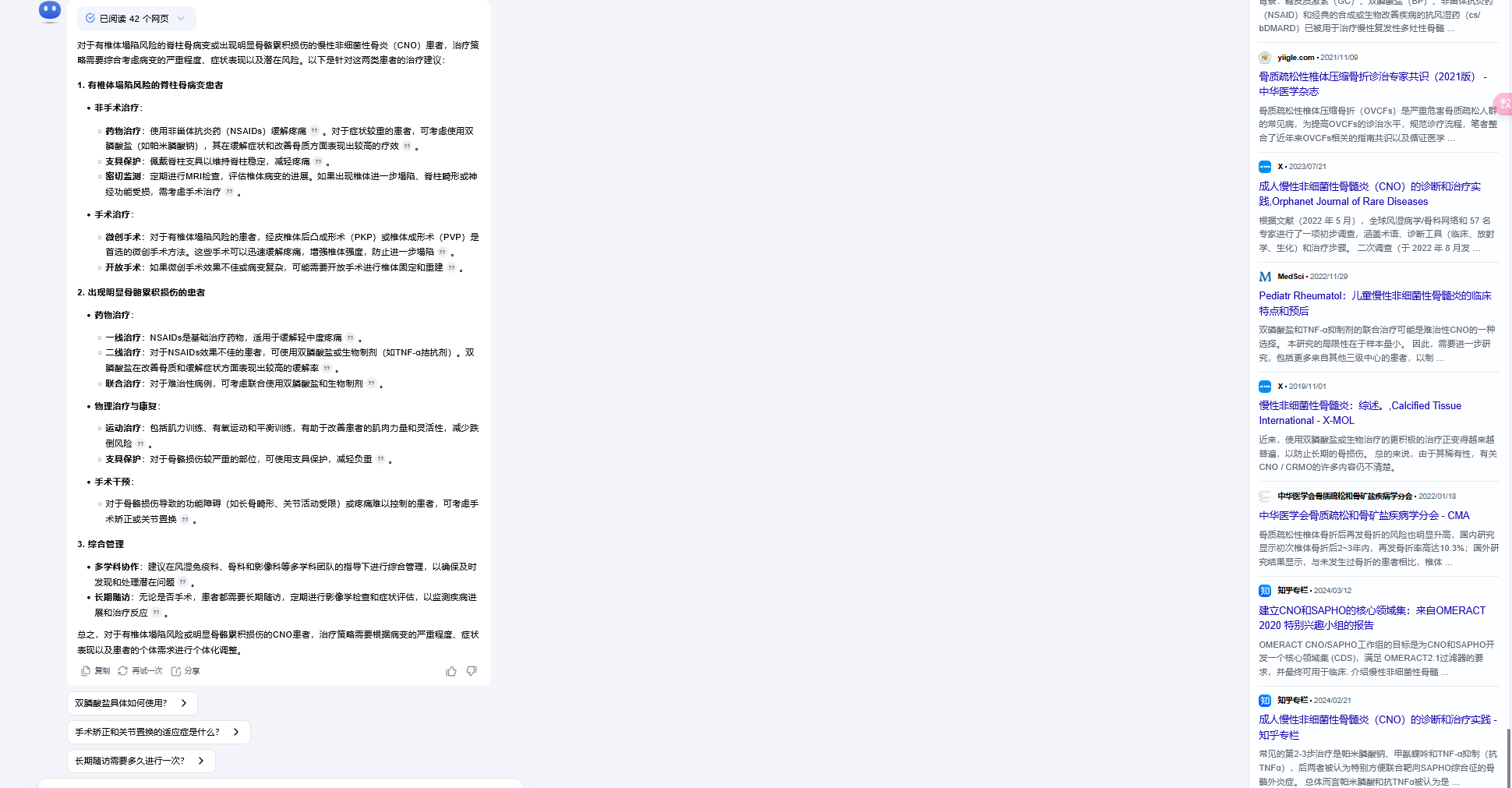


对于有椎体塌陷风险的脊柱骨病变或出现明显骨骼累积损伤的慢性非细菌性骨炎（CNO）患者，治疗策略需要综合考虑病变的严重程度、症状表现以及潜在风险。以下是针对这两类患者的治疗建议：

### 1. **有椎体塌陷风险的脊柱骨病变患者**

- **非手术治疗**：

- **药物治疗**：使用非甾体抗炎药（NSAIDs）缓解疼痛。对于症状较重的患者，可考虑使用双膦酸盐（如帕米膦酸钠），其在缓解症状和改善骨质方面表现出较高的疗效。

- **支具保护**：佩戴脊柱支具以维持脊柱稳定，减轻疼痛。

- **密切监测**：定期进行MRI检查，评估椎体病变的进展。如果出现椎体进一步塌陷、脊柱畸形或神经功能受损，需考虑手术治疗。

- **手术治疗**：

- **微创手术**：对于有椎体塌陷风险的患者，经皮椎体后凸成形术（PKP）或椎体成形术（PVP）是首选的微创手术方法。这些手术可以迅速缓解疼痛，增强椎体强度，防止进一步塌陷。

- **开放手术**：如果微创手术效果不佳或病变复杂，可能需要开放手术进行椎体固定和重建。

### 2. **出现明显骨骼累积损伤的患者**

- **药物治疗**：

- **一线治疗**：NSAIDs是基础治疗药物，适用于缓解轻中度疼痛。

- **二线治疗**：对于NSAIDs效果不佳的患者，可使用双膦酸盐或生物制剂（如TNF-α拮抗剂）。双膦酸盐在改善骨质和缓解症状方面表现出较高的缓解率。

- **联合治疗**：对于难治性病例，可考虑联合使用双膦酸盐和生物制剂。

- **物理治疗与康复**：

- **运动治疗**：包括肌力训练、有氧运动和平衡训练，有助于改善患者的肌肉力量和灵活性，减少跌倒风险。

- **支具保护**：对于骨骼损伤较严重的部位，可使用支具保护，减轻负重。

- **手术干预**：

- 对于骨骼损伤导致的功能障碍（如长骨畸形、关节活动受限）或疼痛难以控制的患者，可考虑手术矫正或关节置换。

### 3. **综合管理**

- **多学科协作**：建议在风湿免疫科、骨科和影像科等多学科团队的指导下进行综合管理，以确保及时发现和处理潜在问题。

- **长期随访**：无论是否手术，患者都需要长期随访，定期进行影像学检查和症状评估，以监测疾病进展和治疗反应。

总之，对于有椎体塌陷风险或明显骨骼累积损伤的CNO患者，治疗策略需要根据病变的严重程度、症状表现以及患者的个体需求进行个体化调整。
