# Supplementary material for: Evaluation of the accuracy and repeatability of Deepseek V3, Doubao, and Kimi1.5 in answering knowledge-related queries about chronic non-bacterial osteitis
Source: Front Artif Intell. 2025 Sep 29;8:1629149. doi: 10.3389/frai.2025.1629149 (PMC12515971; doi:10.3389/frai.2025.1629149)
Supplement: Supplementary file 1 [file Data_Sheet_1.zip › day28/day28-Deepseek.docx]

1成人慢性非细菌性骨炎的定义是什么？


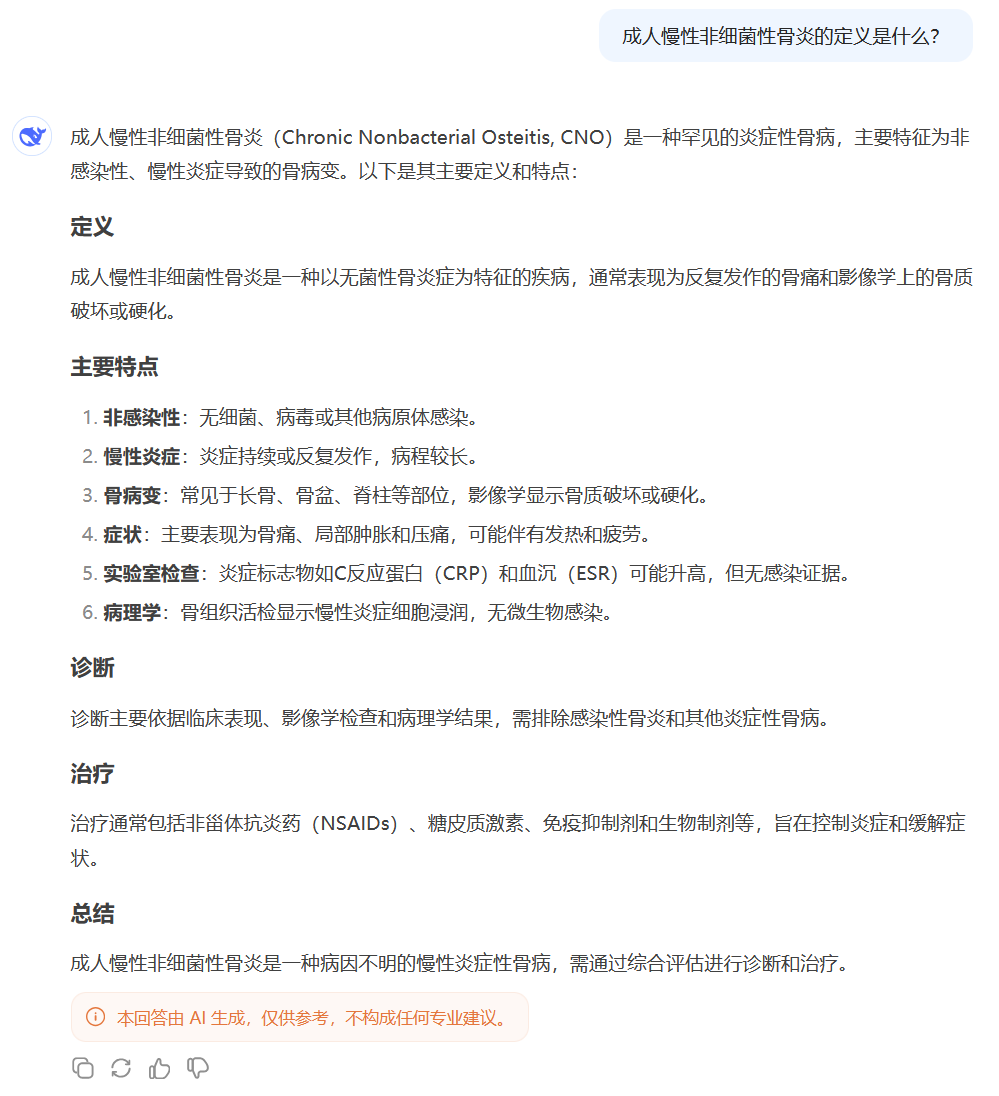
成人慢性非细菌性骨炎（Chronic Nonbacterial Osteitis, CNO）是一种罕见的炎症性骨病，主要特征为非感染性、慢性炎症导致的骨病变。以下是其主要定义和特点：

### 定义

成人慢性非细菌性骨炎是一种以无菌性骨炎症为特征的疾病，通常表现为反复发作的骨痛和影像学上的骨质破坏或硬化。

### 主要特点

1. **非感染性**：无细菌、病毒或其他病原体感染。

2. **慢性炎症**：炎症持续或反复发作，病程较长。

3. **骨病变**：常见于长骨、骨盆、脊柱等部位，影像学显示骨质破坏或硬化。

4. **症状**：主要表现为骨痛、局部肿胀和压痛，可能伴有发热和疲劳。

5. **实验室检查**：炎症标志物如C反应蛋白（CRP）和血沉（ESR）可能升高，但无感染证据。

6. **病理学**：骨组织活检显示慢性炎症细胞浸润，无微生物感染。

### 诊断

诊断主要依据临床表现、影像学检查和病理学结果，需排除感染性骨炎和其他炎症性骨病。

### 治疗

治疗通常包括非甾体抗炎药（NSAIDs）、糖皮质激素、免疫抑制剂和生物制剂等，旨在控制炎症和缓解症状。

### 总结

成人慢性非细菌性骨炎是一种病因不明的慢性炎症性骨病，需通过综合评估进行诊断和治疗。

2成人慢性非细菌性骨炎的典型影像学特征是什么？


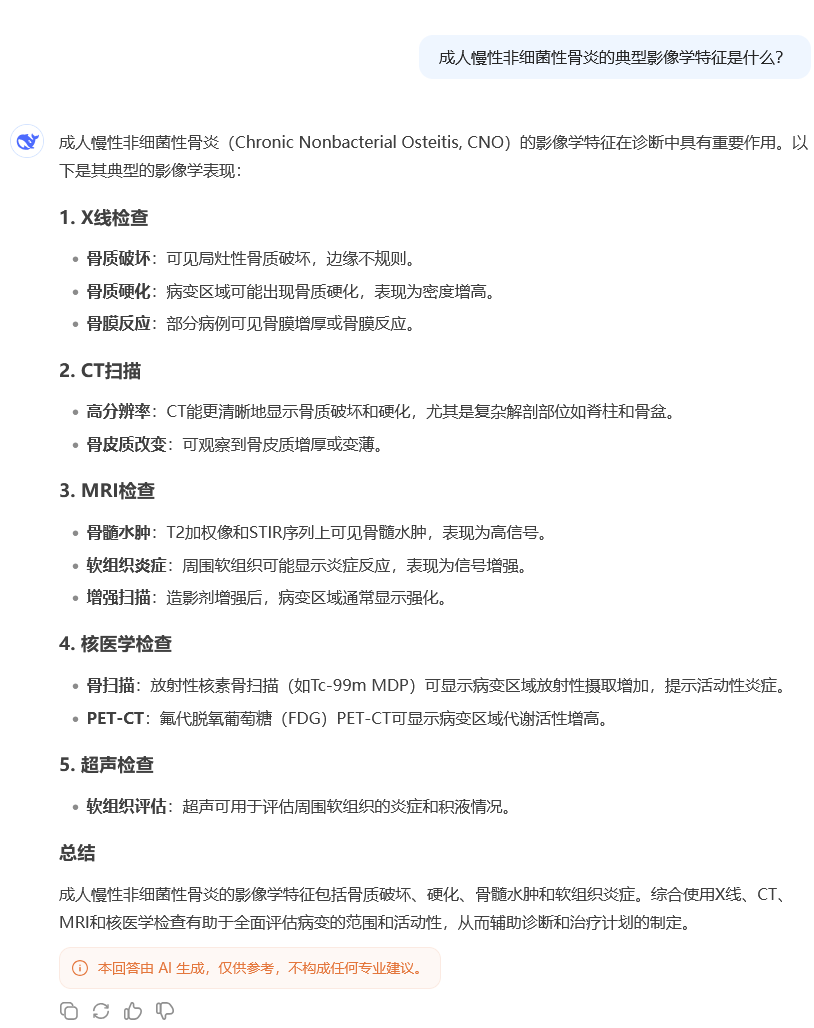
成人慢性非细菌性骨炎（Chronic Nonbacterial Osteitis, CNO）的影像学特征在诊断中具有重要作用。以下是其典型的影像学表现：

### 1. **X线检查**

- **骨质破坏**：可见局灶性骨质破坏，边缘不规则。

- **骨质硬化**：病变区域可能出现骨质硬化，表现为密度增高。

- **骨膜反应**：部分病例可见骨膜增厚或骨膜反应。

### 2. **CT扫描**

- **高分辨率**：CT能更清晰地显示骨质破坏和硬化，尤其是复杂解剖部位如脊柱和骨盆。

- **骨皮质改变**：可观察到骨皮质增厚或变薄。

### 3. **MRI检查**

- **骨髓水肿**：T2加权像和STIR序列上可见骨髓水肿，表现为高信号。

- **软组织炎症**：周围软组织可能显示炎症反应，表现为信号增强。

- **增强扫描**：造影剂增强后，病变区域通常显示强化。

### 4. **核医学检查**

- **骨扫描**：放射性核素骨扫描（如Tc-99m MDP）可显示病变区域放射性摄取增加，提示活动性炎症。

- **PET-CT**：氟代脱氧葡萄糖（FDG）PET-CT可显示病变区域代谢活性增高。

### 5. **超声检查**

- **软组织评估**：超声可用于评估周围软组织的炎症和积液情况。

### 总结

成人慢性非细菌性骨炎的影像学特征包括骨质破坏、硬化、骨髓水肿和软组织炎症。综合使用X线、CT、MRI和核医学检查有助于全面评估病变的范围和活动性，从而辅助诊断和治疗计划的制定。

3成人慢性非细菌性骨炎最常见的表现是什么？


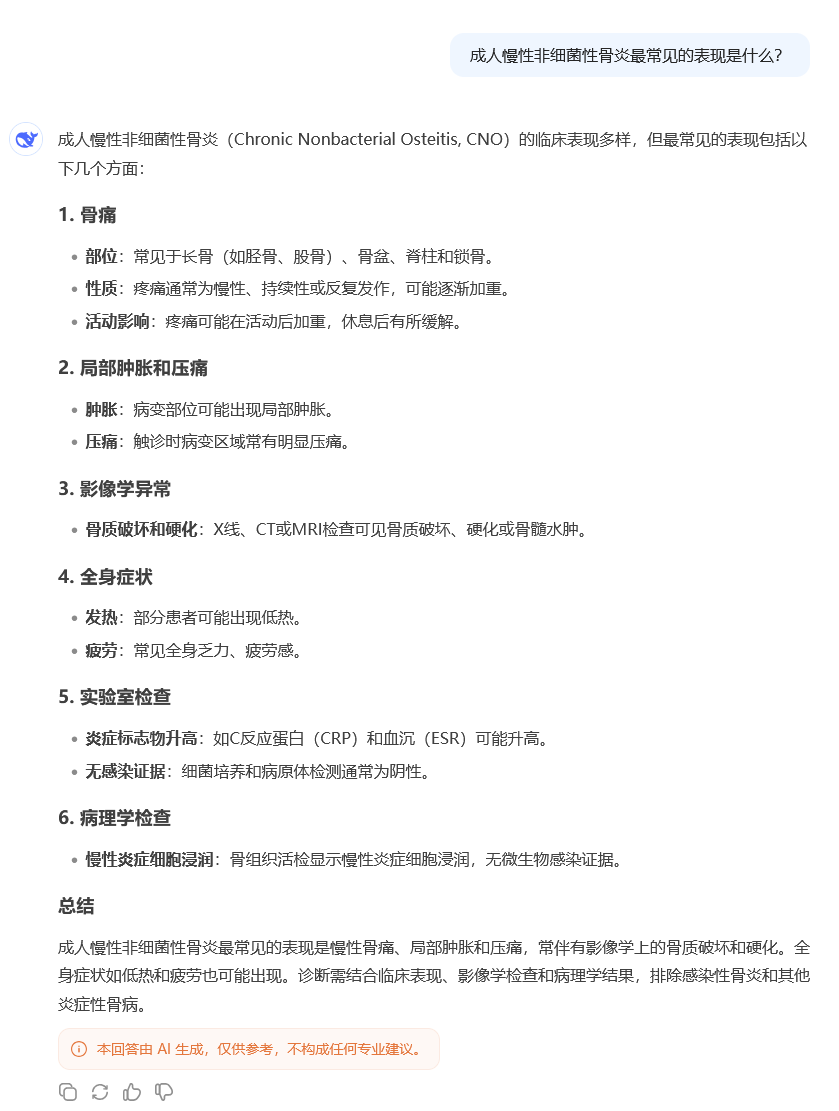
成人慢性非细菌性骨炎（Chronic Nonbacterial Osteitis, CNO）的临床表现多样，但最常见的表现包括以下几个方面：

### 1. **骨痛**

- **部位**：常见于长骨（如胫骨、股骨）、骨盆、脊柱和锁骨。

- **性质**：疼痛通常为慢性、持续性或反复发作，可能逐渐加重。

- **活动影响**：疼痛可能在活动后加重，休息后有所缓解。

### 2. **局部肿胀和压痛**

- **肿胀**：病变部位可能出现局部肿胀。

- **压痛**：触诊时病变区域常有明显压痛。

### 3. **影像学异常**

- **骨质破坏和硬化**：X线、CT或MRI检查可见骨质破坏、硬化或骨髓水肿。

### 4. **全身症状**

- **发热**：部分患者可能出现低热。

- **疲劳**：常见全身乏力、疲劳感。

### 5. **实验室检查**

- **炎症标志物升高**：如C反应蛋白（CRP）和血沉（ESR）可能升高。

- **无感染证据**：细菌培养和病原体检测通常为阴性。

### 6. **病理学检查**

- **慢性炎症细胞浸润**：骨组织活检显示慢性炎症细胞浸润，无微生物感染证据。

### 总结

成人慢性非细菌性骨炎最常见的表现是慢性骨痛、局部肿胀和压痛，常伴有影像学上的骨质破坏和硬化。全身症状如低热和疲劳也可能出现。诊断需结合临床表现、影像学检查和病理学结果，排除感染性骨炎和其他炎症性骨病。

4成人慢性非细菌性骨炎最常受累的部位是那些？


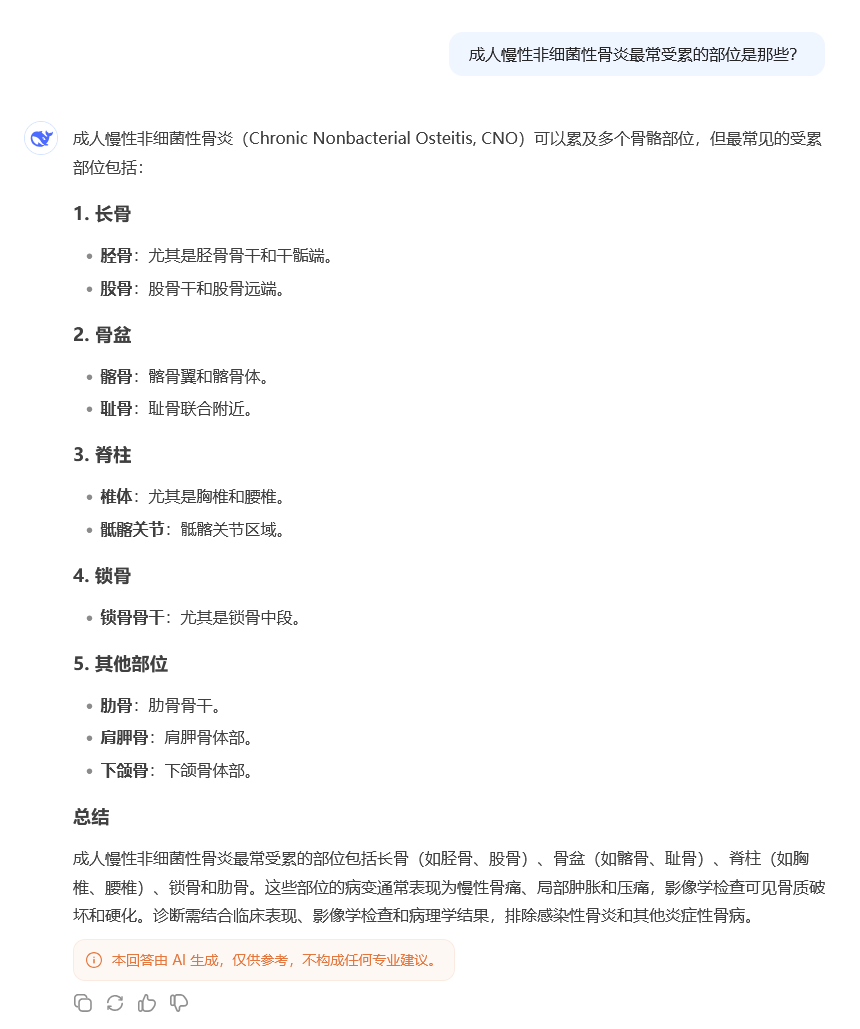
成人慢性非细菌性骨炎（Chronic Nonbacterial Osteitis, CNO）可以累及多个骨骼部位，但最常见的受累部位包括：

### 1. **长骨**

- **胫骨**：尤其是胫骨骨干和干骺端。

- **股骨**：股骨干和股骨远端。

### 2. **骨盆**

- **髂骨**：髂骨翼和髂骨体。

- **耻骨**：耻骨联合附近。

### 3. **脊柱**

- **椎体**：尤其是胸椎和腰椎。

- **骶髂关节**：骶髂关节区域。

### 4. **锁骨**

- **锁骨骨干**：尤其是锁骨中段。

### 5. **其他部位**

- **肋骨**：肋骨骨干。

- **肩胛骨**：肩胛骨体部。

- **下颌骨**：下颌骨体部。

### 总结

成人慢性非细菌性骨炎最常受累的部位包括长骨（如胫骨、股骨）、骨盆（如髂骨、耻骨）、脊柱（如胸椎、腰椎）、锁骨和肋骨。这些部位的病变通常表现为慢性骨痛、局部肿胀和压痛，影像学检查可见骨质破坏和硬化。诊断需结合临床表现、影像学检查和病理学结果，排除感染性骨炎和其他炎症性骨病。

5成人慢性非细菌性骨炎有没有特异性的炎症标志物？


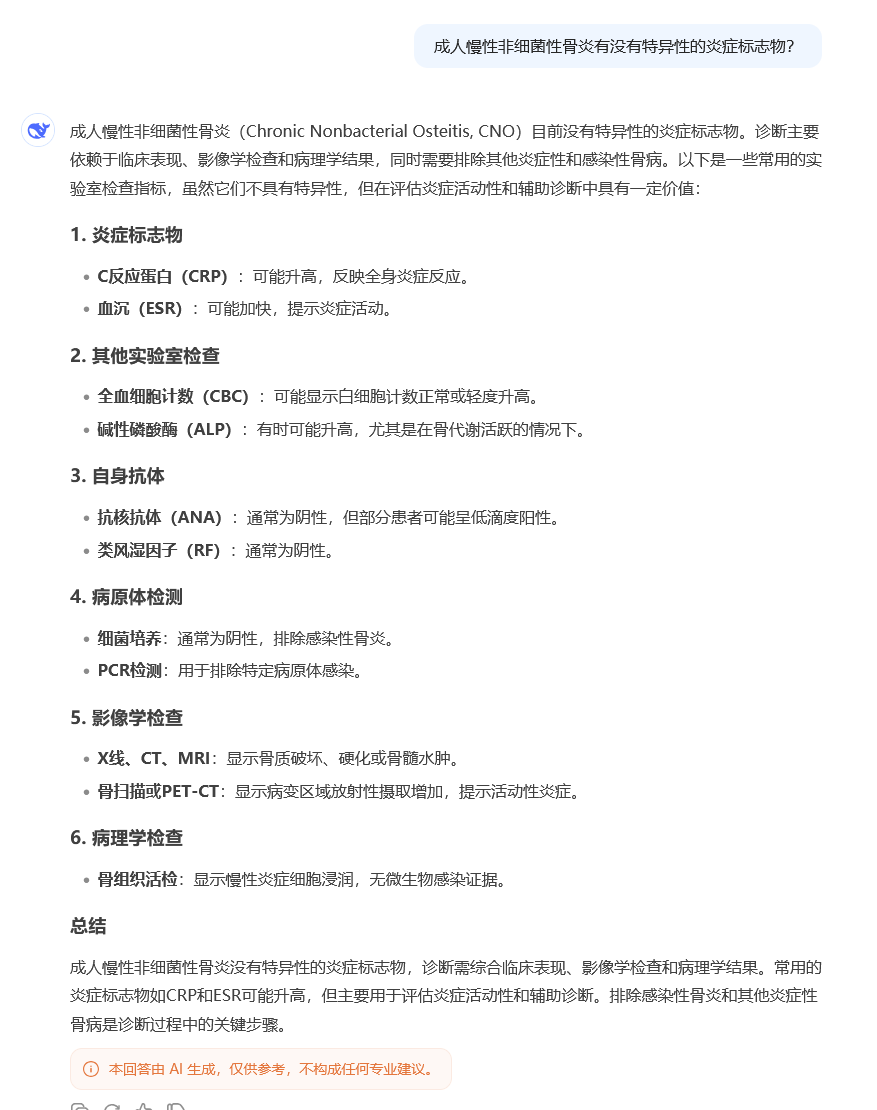
成人慢性非细菌性骨炎（Chronic Nonbacterial Osteitis, CNO）目前没有特异性的炎症标志物。诊断主要依赖于临床表现、影像学检查和病理学结果，同时需要排除其他炎症性和感染性骨病。以下是一些常用的实验室检查指标，虽然它们不具有特异性，但在评估炎症活动性和辅助诊断中具有一定价值：

### 1. **炎症标志物**

- **C反应蛋白（CRP）**：可能升高，反映全身炎症反应。

- **血沉（ESR）**：可能加快，提示炎症活动。

### 2. **其他实验室检查**

- **全血细胞计数（CBC）**：可能显示白细胞计数正常或轻度升高。

- **碱性磷酸酶（ALP）**：有时可能升高，尤其是在骨代谢活跃的情况下。

### 3. **自身抗体**

- **抗核抗体（ANA）**：通常为阴性，但部分患者可能呈低滴度阳性。

- **类风湿因子（RF）**：通常为阴性。

### 4. **病原体检测**

- **细菌培养**：通常为阴性，排除感染性骨炎。

- **PCR检测**：用于排除特定病原体感染。

### 5. **影像学检查**

- **X线、CT、MRI**：显示骨质破坏、硬化或骨髓水肿。

- **骨扫描或PET-CT**：显示病变区域放射性摄取增加，提示活动性炎症。

### 6. **病理学检查**

- **骨组织活检**：显示慢性炎症细胞浸润，无微生物感染证据。

### 总结

成人慢性非细菌性骨炎没有特异性的炎症标志物，诊断需综合临床表现、影像学检查和病理学结果。常用的炎症标志物如CRP和ESR可能升高，但主要用于评估炎症活动性和辅助诊断。排除感染性骨炎和其他炎症性骨病是诊断过程中的关键步骤。

6慢性非细菌性骨炎首选的影像学检查是什么？


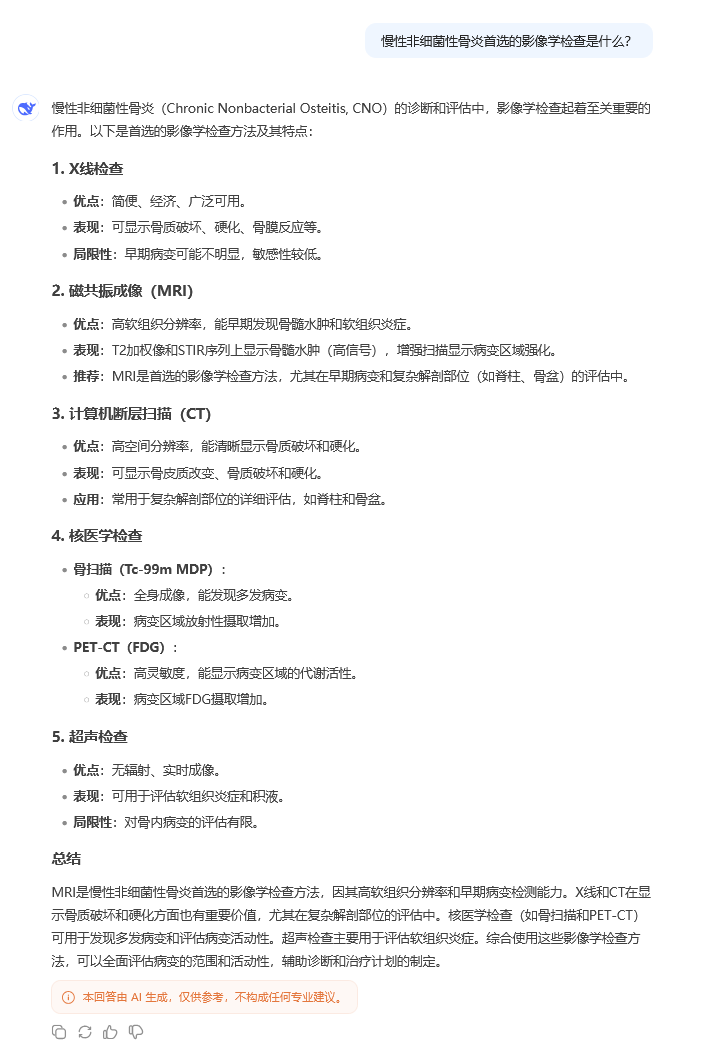
慢性非细菌性骨炎（Chronic Nonbacterial Osteitis, CNO）的诊断和评估中，影像学检查起着至关重要的作用。以下是首选的影像学检查方法及其特点：

### 1. **X线检查**

- **优点**：简便、经济、广泛可用。

- **表现**：可显示骨质破坏、硬化、骨膜反应等。

- **局限性**：早期病变可能不明显，敏感性较低。

### 2. **磁共振成像（MRI）**

- **优点**：高软组织分辨率，能早期发现骨髓水肿和软组织炎症。

- **表现**：T2加权像和STIR序列上显示骨髓水肿（高信号），增强扫描显示病变区域强化。

- **推荐**：MRI是首选的影像学检查方法，尤其在早期病变和复杂解剖部位（如脊柱、骨盆）的评估中。

### 3. **计算机断层扫描（CT）**

- **优点**：高空间分辨率，能清晰显示骨质破坏和硬化。

- **表现**：可显示骨皮质改变、骨质破坏和硬化。

- **应用**：常用于复杂解剖部位的详细评估，如脊柱和骨盆。

### 4. **核医学检查**

- **骨扫描（Tc-99m MDP）**：

- **优点**：全身成像，能发现多发病变。

- **表现**：病变区域放射性摄取增加。

- **PET-CT（FDG）**：

- **优点**：高灵敏度，能显示病变区域的代谢活性。

- **表现**：病变区域FDG摄取增加。

### 5. **超声检查**

- **优点**：无辐射、实时成像。

- **表现**：可用于评估软组织炎症和积液。

- **局限性**：对骨内病变的评估有限。

### 总结

MRI是慢性非细菌性骨炎首选的影像学检查方法，因其高软组织分辨率和早期病变检测能力。X线和CT在显示骨质破坏和硬化方面也有重要价值，尤其在复杂解剖部位的评估中。核医学检查（如骨扫描和PET-CT）可用于发现多发病变和评估病变活动性。超声检查主要用于评估软组织炎症。综合使用这些影像学检查方法，可以全面评估病变的范围和活动性，辅助诊断和治疗计划的制定。

7全身成像检查是不是诊断成人慢性非细菌性骨炎的先决条件？


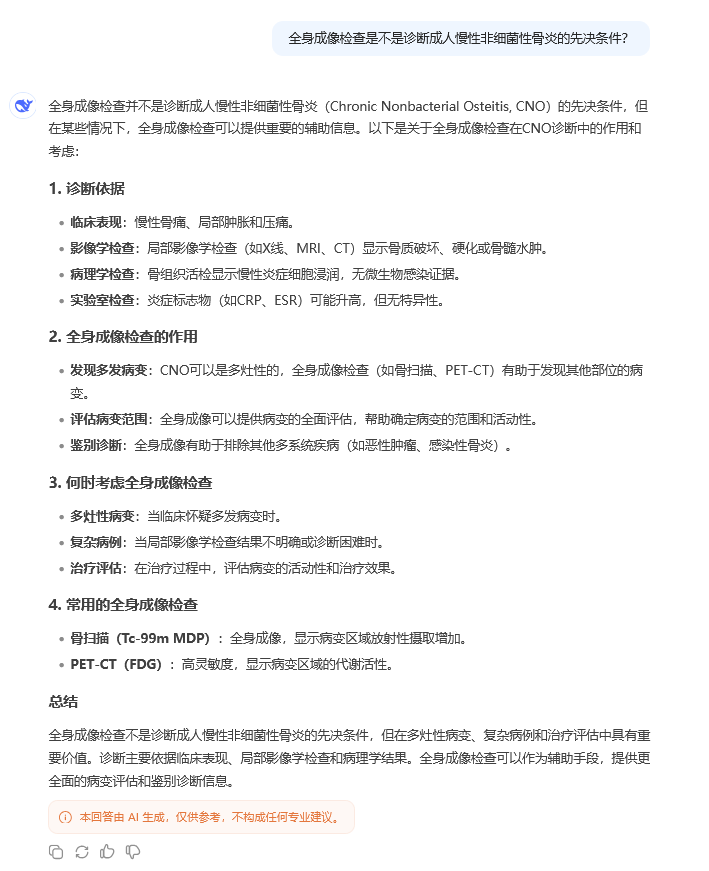
全身成像检查并不是诊断成人慢性非细菌性骨炎（Chronic Nonbacterial Osteitis, CNO）的先决条件，但在某些情况下，全身成像检查可以提供重要的辅助信息。以下是关于全身成像检查在CNO诊断中的作用和考虑：

### 1. **诊断依据**

- **临床表现**：慢性骨痛、局部肿胀和压痛。

- **影像学检查**：局部影像学检查（如X线、MRI、CT）显示骨质破坏、硬化或骨髓水肿。

- **病理学检查**：骨组织活检显示慢性炎症细胞浸润，无微生物感染证据。

- **实验室检查**：炎症标志物（如CRP、ESR）可能升高，但无特异性。

### 2. **全身成像检查的作用**

- **发现多发病变**：CNO可以是多灶性的，全身成像检查（如骨扫描、PET-CT）有助于发现其他部位的病变。

- **评估病变范围**：全身成像可以提供病变的全面评估，帮助确定病变的范围和活动性。

- **鉴别诊断**：全身成像有助于排除其他多系统疾病（如恶性肿瘤、感染性骨炎）。

### 3. **何时考虑全身成像检查**

- **多灶性病变**：当临床怀疑多发病变时。

- **复杂病例**：当局部影像学检查结果不明确或诊断困难时。

- **治疗评估**：在治疗过程中，评估病变的活动性和治疗效果。

### 4. **常用的全身成像检查**

- **骨扫描（Tc-99m MDP）**：全身成像，显示病变区域放射性摄取增加。

- **PET-CT（FDG）**：高灵敏度，显示病变区域的代谢活性。

### 总结

全身成像检查不是诊断成人慢性非细菌性骨炎的先决条件，但在多灶性病变、复杂病例和治疗评估中具有重要价值。诊断主要依据临床表现、局部影像学检查和病理学结果。全身成像检查可以作为辅助手段，提供更全面的病变评估和鉴别诊断信息。

8诊断成人慢性非细菌性骨炎有必要常规骨活检吗？


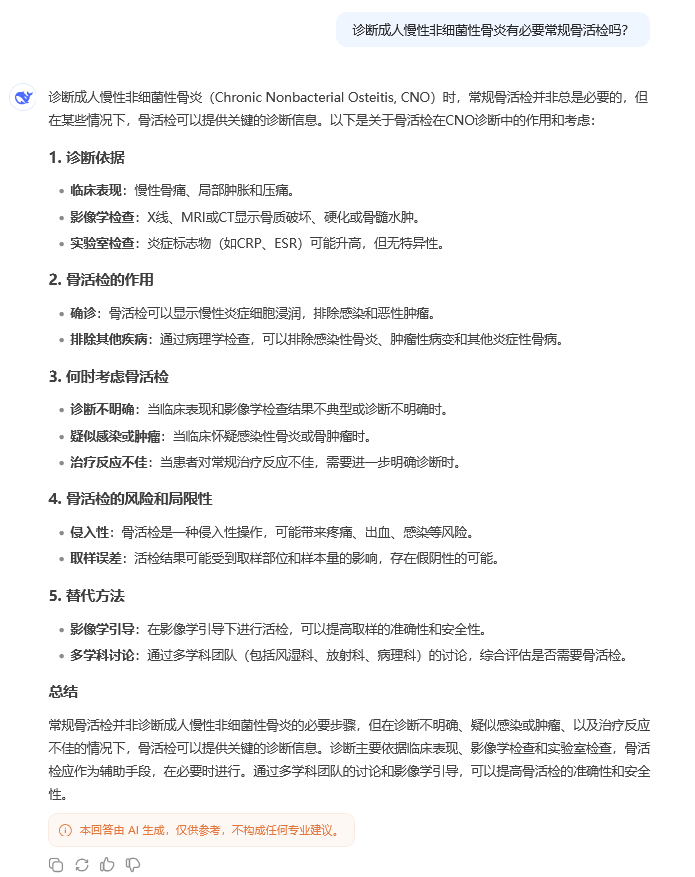
诊断成人慢性非细菌性骨炎（Chronic Nonbacterial Osteitis, CNO）时，常规骨活检并非总是必要的，但在某些情况下，骨活检可以提供关键的诊断信息。以下是关于骨活检在CNO诊断中的作用和考虑：

### 1. **诊断依据**

- **临床表现**：慢性骨痛、局部肿胀和压痛。

- **影像学检查**：X线、MRI或CT显示骨质破坏、硬化或骨髓水肿。

- **实验室检查**：炎症标志物（如CRP、ESR）可能升高，但无特异性。

### 2. **骨活检的作用**

- **确诊**：骨活检可以显示慢性炎症细胞浸润，排除感染和恶性肿瘤。

- **排除其他疾病**：通过病理学检查，可以排除感染性骨炎、肿瘤性病变和其他炎症性骨病。

### 3. **何时考虑骨活检**

- **诊断不明确**：当临床表现和影像学检查结果不典型或诊断不明确时。

- **疑似感染或肿瘤**：当临床怀疑感染性骨炎或骨肿瘤时。

- **治疗反应不佳**：当患者对常规治疗反应不佳，需要进一步明确诊断时。

### 4. **骨活检的风险和局限性**

- **侵入性**：骨活检是一种侵入性操作，可能带来疼痛、出血、感染等风险。

- **取样误差**：活检结果可能受到取样部位和样本量的影响，存在假阴性的可能。

### 5. **替代方法**

- **影像学引导**：在影像学引导下进行活检，可以提高取样的准确性和安全性。

- **多学科讨论**：通过多学科团队（包括风湿科、放射科、病理科）的讨论，综合评估是否需要骨活检。

### 总结

常规骨活检并非诊断成人慢性非细菌性骨炎的必要步骤，但在诊断不明确、疑似感染或肿瘤、以及治疗反应不佳的情况下，骨活检可以提供关键的诊断信息。诊断主要依据临床表现、影像学检查和实验室检查，骨活检应作为辅助手段，在必要时进行。通过多学科团队的讨论和影像学引导，可以提高骨活检的准确性和安全性。

9慢性非细菌性骨炎如何与恶性骨肿瘤鉴别？


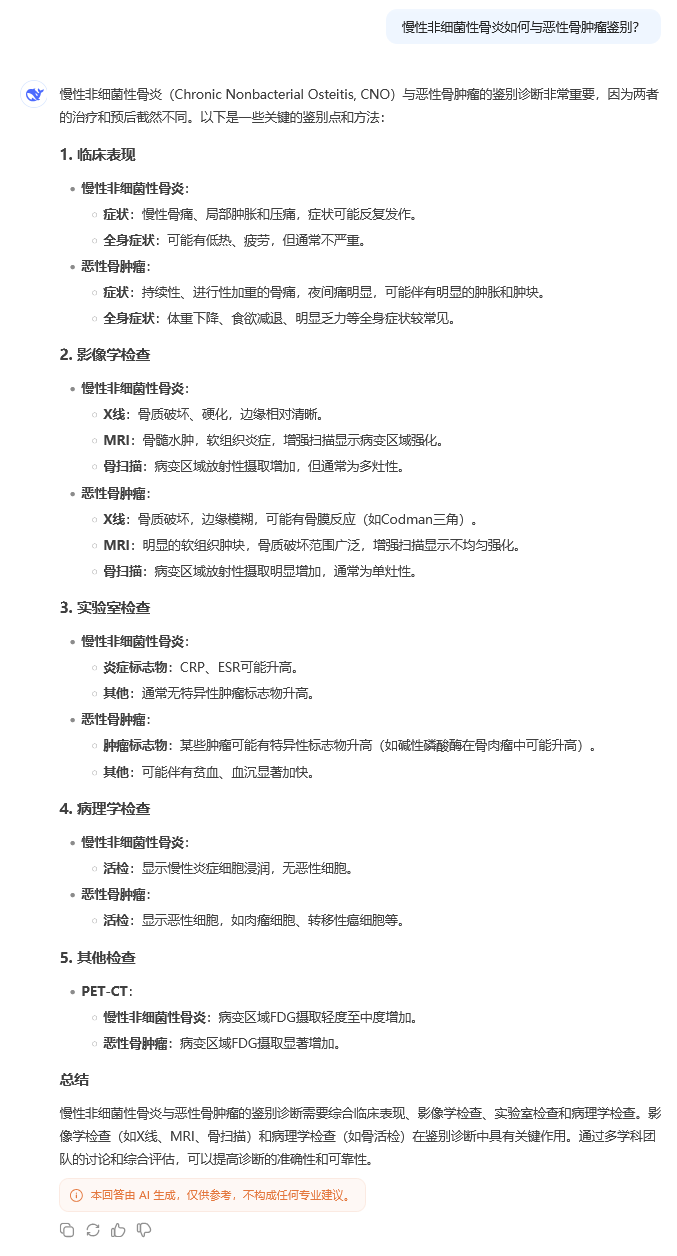
慢性非细菌性骨炎（Chronic Nonbacterial Osteitis, CNO）与恶性骨肿瘤的鉴别诊断非常重要，因为两者的治疗和预后截然不同。以下是一些关键的鉴别点和方法：

### 1. **临床表现**

- **慢性非细菌性骨炎**：

- **症状**：慢性骨痛、局部肿胀和压痛，症状可能反复发作。

- **全身症状**：可能有低热、疲劳，但通常不严重。

- **恶性骨肿瘤**：

- **症状**：持续性、进行性加重的骨痛，夜间痛明显，可能伴有明显的肿胀和肿块。

- **全身症状**：体重下降、食欲减退、明显乏力等全身症状较常见。

### 2. **影像学检查**

- **慢性非细菌性骨炎**：

- **X线**：骨质破坏、硬化，边缘相对清晰。

- **MRI**：骨髓水肿，软组织炎症，增强扫描显示病变区域强化。

- **骨扫描**：病变区域放射性摄取增加，但通常为多灶性。

- **恶性骨肿瘤**：

- **X线**：骨质破坏，边缘模糊，可能有骨膜反应（如Codman三角）。

- **MRI**：明显的软组织肿块，骨质破坏范围广泛，增强扫描显示不均匀强化。

- **骨扫描**：病变区域放射性摄取明显增加，通常为单灶性。

### 3. **实验室检查**

- **慢性非细菌性骨炎**：

- **炎症标志物**：CRP、ESR可能升高。

- **其他**：通常无特异性肿瘤标志物升高。

- **恶性骨肿瘤**：

- **肿瘤标志物**：某些肿瘤可能有特异性标志物升高（如碱性磷酸酶在骨肉瘤中可能升高）。

- **其他**：可能伴有贫血、血沉显著加快。

### 4. **病理学检查**

- **慢性非细菌性骨炎**：

- **活检**：显示慢性炎症细胞浸润，无恶性细胞。

- **恶性骨肿瘤**：

- **活检**：显示恶性细胞，如肉瘤细胞、转移性癌细胞等。

### 5. **其他检查**

- **PET-CT**：

- **慢性非细菌性骨炎**：病变区域FDG摄取轻度至中度增加。

- **恶性骨肿瘤**：病变区域FDG摄取显著增加。

### 总结

慢性非细菌性骨炎与恶性骨肿瘤的鉴别诊断需要综合临床表现、影像学检查、实验室检查和病理学检查。影像学检查（如X线、MRI、骨扫描）和病理学检查（如骨活检）在鉴别诊断中具有关键作用。通过多学科团队的讨论和综合评估，可以提高诊断的准确性和可靠性。

10慢性非细菌性骨炎如何与骨关节炎鉴别？


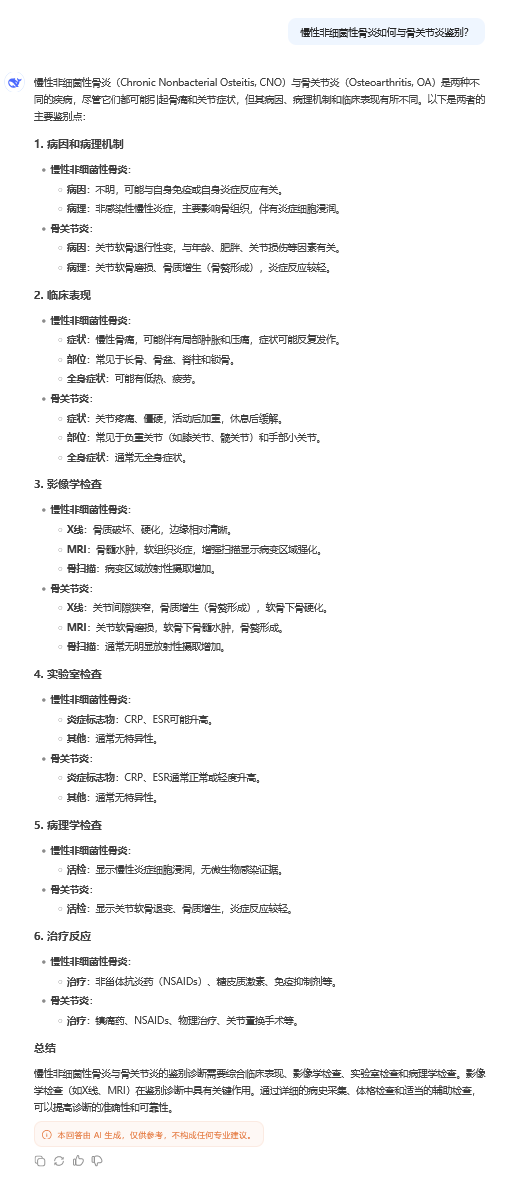
慢性非细菌性骨炎（Chronic Nonbacterial Osteitis, CNO）与骨关节炎（Osteoarthritis, OA）是两种不同的疾病，尽管它们都可能引起骨痛和关节症状，但其病因、病理机制和临床表现有所不同。以下是两者的主要鉴别点：

### 1. **病因和病理机制**

- **慢性非细菌性骨炎**：

- **病因**：不明，可能与自身免疫或自身炎症反应有关。

- **病理**：非感染性慢性炎症，主要影响骨组织，伴有炎症细胞浸润。

- **骨关节炎**：

- **病因**：关节软骨退行性变，与年龄、肥胖、关节损伤等因素有关。

- **病理**：关节软骨磨损、骨质增生（骨赘形成），炎症反应较轻。

### 2. **临床表现**

- **慢性非细菌性骨炎**：

- **症状**：慢性骨痛，可能伴有局部肿胀和压痛，症状可能反复发作。

- **部位**：常见于长骨、骨盆、脊柱和锁骨。

- **全身症状**：可能有低热、疲劳。

- **骨关节炎**：

- **症状**：关节疼痛、僵硬，活动后加重，休息后缓解。

- **部位**：常见于负重关节（如膝关节、髋关节）和手部小关节。

- **全身症状**：通常无全身症状。

### 3. **影像学检查**

- **慢性非细菌性骨炎**：

- **X线**：骨质破坏、硬化，边缘相对清晰。

- **MRI**：骨髓水肿，软组织炎症，增强扫描显示病变区域强化。

- **骨扫描**：病变区域放射性摄取增加。

- **骨关节炎**：

- **X线**：关节间隙狭窄，骨质增生（骨赘形成），软骨下骨硬化。

- **MRI**：关节软骨磨损，软骨下骨髓水肿，骨赘形成。

- **骨扫描**：通常无明显放射性摄取增加。

### 4. **实验室检查**

- **慢性非细菌性骨炎**：

- **炎症标志物**：CRP、ESR可能升高。

- **其他**：通常无特异性。

- **骨关节炎**：

- **炎症标志物**：CRP、ESR通常正常或轻度升高。

- **其他**：通常无特异性。

### 5. **病理学检查**

- **慢性非细菌性骨炎**：

- **活检**：显示慢性炎症细胞浸润，无微生物感染证据。

- **骨关节炎**：

- **活检**：显示关节软骨退变、骨质增生，炎症反应较轻。

### 6. **治疗反应**

- **慢性非细菌性骨炎**：

- **治疗**：非甾体抗炎药（NSAIDs）、糖皮质激素、免疫抑制剂等。

- **骨关节炎**：

- **治疗**：镇痛药、NSAIDs、物理治疗、关节置换手术等。

### 总结

慢性非细菌性骨炎与骨关节炎的鉴别诊断需要综合临床表现、影像学检查、实验室检查和病理学检查。影像学检查（如X线、MRI）在鉴别诊断中具有关键作用。通过详细的病史采集、体格检查和适当的辅助检查，可以提高诊断的准确性和可靠性。

11慢性非细菌性骨炎如何与纤维发育不良鉴别？


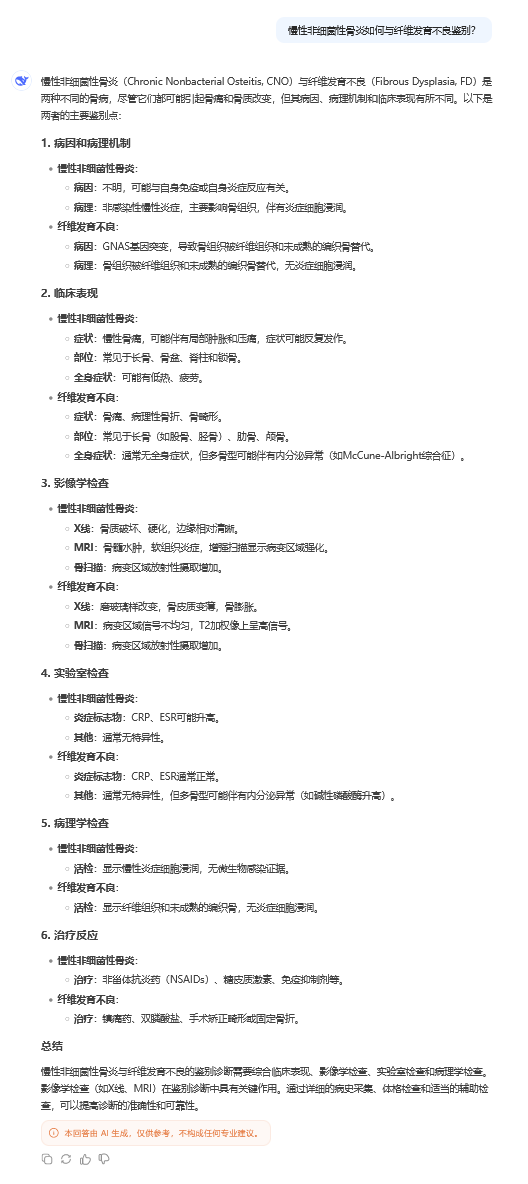
慢性非细菌性骨炎（Chronic Nonbacterial Osteitis, CNO）与纤维发育不良（Fibrous Dysplasia, FD）是两种不同的骨病，尽管它们都可能引起骨痛和骨质改变，但其病因、病理机制和临床表现有所不同。以下是两者的主要鉴别点：

### 1. **病因和病理机制**

- **慢性非细菌性骨炎**：

- **病因**：不明，可能与自身免疫或自身炎症反应有关。

- **病理**：非感染性慢性炎症，主要影响骨组织，伴有炎症细胞浸润。

- **纤维发育不良**：

- **病因**：GNAS基因突变，导致骨组织被纤维组织和未成熟的编织骨替代。

- **病理**：骨组织被纤维组织和未成熟的编织骨替代，无炎症细胞浸润。

### 2. **临床表现**

- **慢性非细菌性骨炎**：

- **症状**：慢性骨痛，可能伴有局部肿胀和压痛，症状可能反复发作。

- **部位**：常见于长骨、骨盆、脊柱和锁骨。

- **全身症状**：可能有低热、疲劳。

- **纤维发育不良**：

- **症状**：骨痛、病理性骨折、骨畸形。

- **部位**：常见于长骨（如股骨、胫骨）、肋骨、颅骨。

- **全身症状**：通常无全身症状，但多骨型可能伴有内分泌异常（如McCune-Albright综合征）。

### 3. **影像学检查**

- **慢性非细菌性骨炎**：

- **X线**：骨质破坏、硬化，边缘相对清晰。

- **MRI**：骨髓水肿，软组织炎症，增强扫描显示病变区域强化。

- **骨扫描**：病变区域放射性摄取增加。

- **纤维发育不良**：

- **X线**：磨玻璃样改变，骨皮质变薄，骨膨胀。

- **MRI**：病变区域信号不均匀，T2加权像上呈高信号。

- **骨扫描**：病变区域放射性摄取增加。

### 4. **实验室检查**

- **慢性非细菌性骨炎**：

- **炎症标志物**：CRP、ESR可能升高。

- **其他**：通常无特异性。

- **纤维发育不良**：

- **炎症标志物**：CRP、ESR通常正常。

- **其他**：通常无特异性，但多骨型可能伴有内分泌异常（如碱性磷酸酶升高）。

### 5. **病理学检查**

- **慢性非细菌性骨炎**：

- **活检**：显示慢性炎症细胞浸润，无微生物感染证据。

- **纤维发育不良**：

- **活检**：显示纤维组织和未成熟的编织骨，无炎症细胞浸润。

### 6. **治疗反应**

- **慢性非细菌性骨炎**：

- **治疗**：非甾体抗炎药（NSAIDs）、糖皮质激素、免疫抑制剂等。

- **纤维发育不良**：

- **治疗**：镇痛药、双膦酸盐、手术矫正畸形或固定骨折。

### 总结

慢性非细菌性骨炎与纤维发育不良的鉴别诊断需要综合临床表现、影像学检查、实验室检查和病理学检查。影像学检查（如X线、MRI）在鉴别诊断中具有关键作用。通过详细的病史采集、体格检查和适当的辅助检查，可以提高诊断的准确性和可靠性。

12在治慢性非细菌性骨炎过程中应该选择短疗程的糖皮质激素治疗还是长期的糖皮质激素治疗？


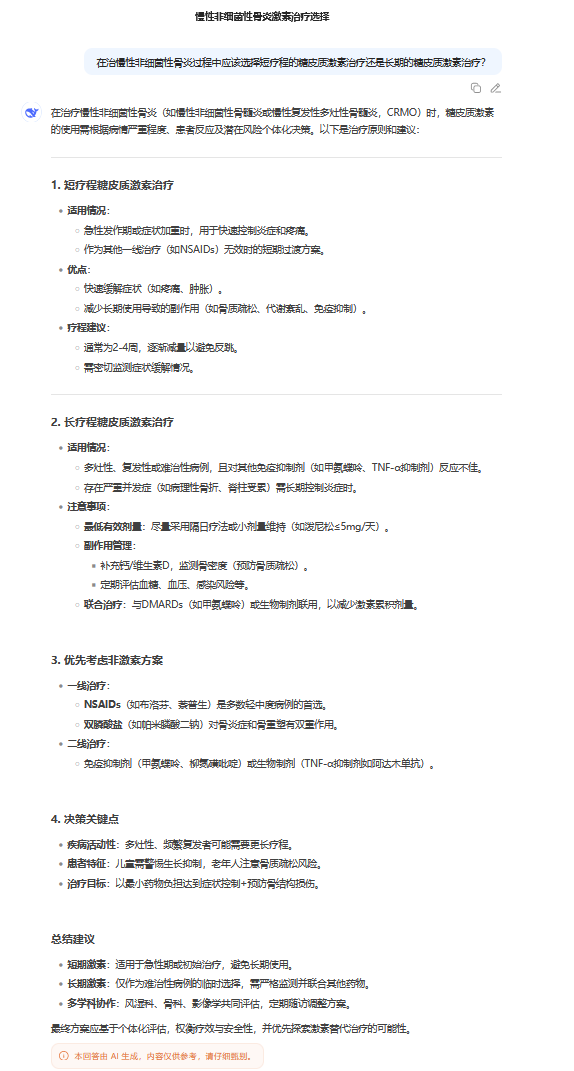
在治疗慢性非细菌性骨炎（如慢性非细菌性骨髓炎或慢性复发性多灶性骨髓炎，CRMO）时，糖皮质激素的使用需根据病情严重程度、患者反应及潜在风险个体化决策。以下是治疗原则和建议：

---

### **1. 短疗程糖皮质激素治疗**

- **适用情况**：

- 急性发作期或症状加重时，用于快速控制炎症和疼痛。

- 作为其他一线治疗（如NSAIDs）无效时的短期过渡方案。

- **优点**：

- 快速缓解症状（如疼痛、肿胀）。

- 减少长期使用导致的副作用（如骨质疏松、代谢紊乱、免疫抑制）。

- **疗程建议**：

- 通常为2-4周，逐渐减量以避免反跳。

- 需密切监测症状缓解情况。

---

### **2. 长疗程糖皮质激素治疗**

- **适用情况**：

- 多灶性、复发性或难治性病例，且对其他免疫抑制剂（如甲氨蝶呤、TNF-α抑制剂）反应不佳。

- 存在严重并发症（如病理性骨折、脊柱受累）需长期控制炎症时。

- **注意事项**：

- **最低有效剂量**：尽量采用隔日疗法或小剂量维持（如泼尼松≤5mg/天）。

- **副作用管理**：

- 补充钙/维生素D，监测骨密度（预防骨质疏松）。

- 定期评估血糖、血压、感染风险等。

- **联合治疗**：与DMARDs（如甲氨蝶呤）或生物制剂联用，以减少激素累积剂量。

---

### **3. 优先考虑非激素方案**

- **一线治疗**：

- **NSAIDs**（如布洛芬、萘普生）是多数轻中度病例的首选。

- **双膦酸盐**（如帕米膦酸二钠）对骨炎症和骨重塑有双重作用。

- **二线治疗**：

- 免疫抑制剂（甲氨蝶呤、柳氮磺吡啶）或生物制剂（TNF-α抑制剂如阿达木单抗）。

---

### **4. 决策关键点**

- **疾病活动性**：多灶性、频繁复发者可能需要更长疗程。

- **患者特征**：儿童需警惕生长抑制，老年人注意骨质疏松风险。

- **治疗目标**：以最小药物负担达到症状控制+预防骨结构损伤。

---

### **总结建议**

- **短期激素**：适用于急性期或初始治疗，避免长期使用。

- **长期激素**：仅作为难治性病例的临时选择，需严格监测并联合其他药物。

- **多学科协作**：风湿科、骨科、影像学共同评估，定期随访调整方案。

最终方案应基于个体化评估，权衡疗效与安全性，并优先探索激素替代治疗的可能性。

13成人慢性非细菌性骨炎一线治疗方案是什么？


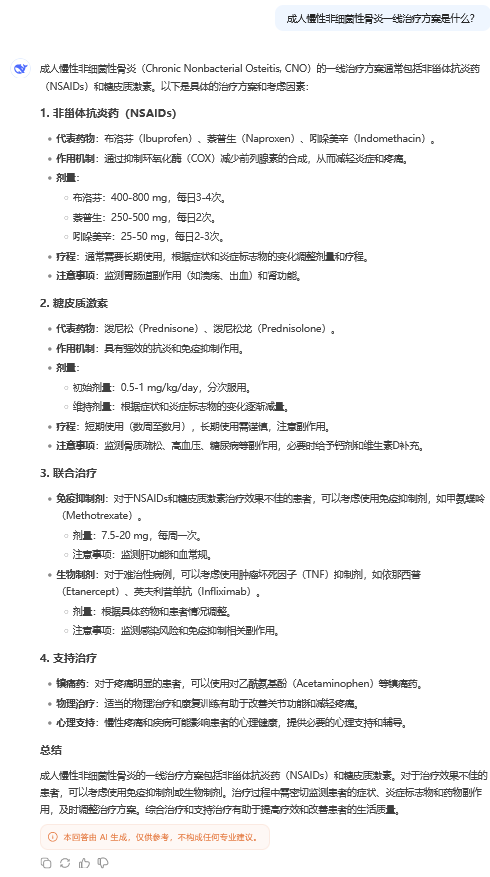


成人慢性非细菌性骨炎（Chronic Nonbacterial Osteitis, CNO）的一线治疗方案通常包括非甾体抗炎药（NSAIDs）和糖皮质激素。以下是具体的治疗方案和考虑因素：

### 1. **非甾体抗炎药（NSAIDs）**

- **代表药物**：布洛芬（Ibuprofen）、萘普生（Naproxen）、吲哚美辛（Indomethacin）。

- **作用机制**：通过抑制环氧化酶（COX）减少前列腺素的合成，从而减轻炎症和疼痛。

- **剂量**：

- 布洛芬：400-800 mg，每日3-4次。

- 萘普生：250-500 mg，每日2次。

- 吲哚美辛：25-50 mg，每日2-3次。

- **疗程**：通常需要长期使用，根据症状和炎症标志物的变化调整剂量和疗程。

- **注意事项**：监测胃肠道副作用（如溃疡、出血）和肾功能。

### 2. **糖皮质激素**

- **代表药物**：泼尼松（Prednisone）、泼尼松龙（Prednisolone）。

- **作用机制**：具有强效的抗炎和免疫抑制作用。

- **剂量**：

- 初始剂量：0.5-1 mg/kg/day，分次服用。

- 维持剂量：根据症状和炎症标志物的变化逐渐减量。

- **疗程**：短期使用（数周至数月），长期使用需谨慎，注意副作用。

- **注意事项**：监测骨质疏松、高血压、糖尿病等副作用，必要时给予钙剂和维生素D补充。

### 3. **联合治疗**

- **免疫抑制剂**：对于NSAIDs和糖皮质激素治疗效果不佳的患者，可以考虑使用免疫抑制剂，如甲氨蝶呤（Methotrexate）。

- 剂量：7.5-20 mg，每周一次。

- 注意事项：监测肝功能和血常规。

- **生物制剂**：对于难治性病例，可以考虑使用肿瘤坏死因子（TNF）抑制剂，如依那西普（Etanercept）、英夫利昔单抗（Infliximab）。

- 剂量：根据具体药物和患者情况调整。

- 注意事项：监测感染风险和免疫抑制相关副作用。

### 4. **支持治疗**

- **镇痛药**：对于疼痛明显的患者，可以使用对乙酰氨基酚（Acetaminophen）等镇痛药。

- **物理治疗**：适当的物理治疗和康复训练有助于改善关节功能和减轻疼痛。

- **心理支持**：慢性疼痛和疾病可能影响患者的心理健康，提供必要的心理支持和辅导。

### 总结

成人慢性非细菌性骨炎的一线治疗方案包括非甾体抗炎药（NSAIDs）和糖皮质激素。对于治疗效果不佳的患者，可以考虑使用免疫抑制剂或生物制剂。治疗过程中需密切监测患者的症状、炎症标志物和药物副作用，及时调整治疗方案。综合治疗和支持治疗有助于提高疗效和改善患者的生活质量。

14无临床症状，也没有放射学疾病活动的慢性非细菌性骨炎的病人需要治疗吗？


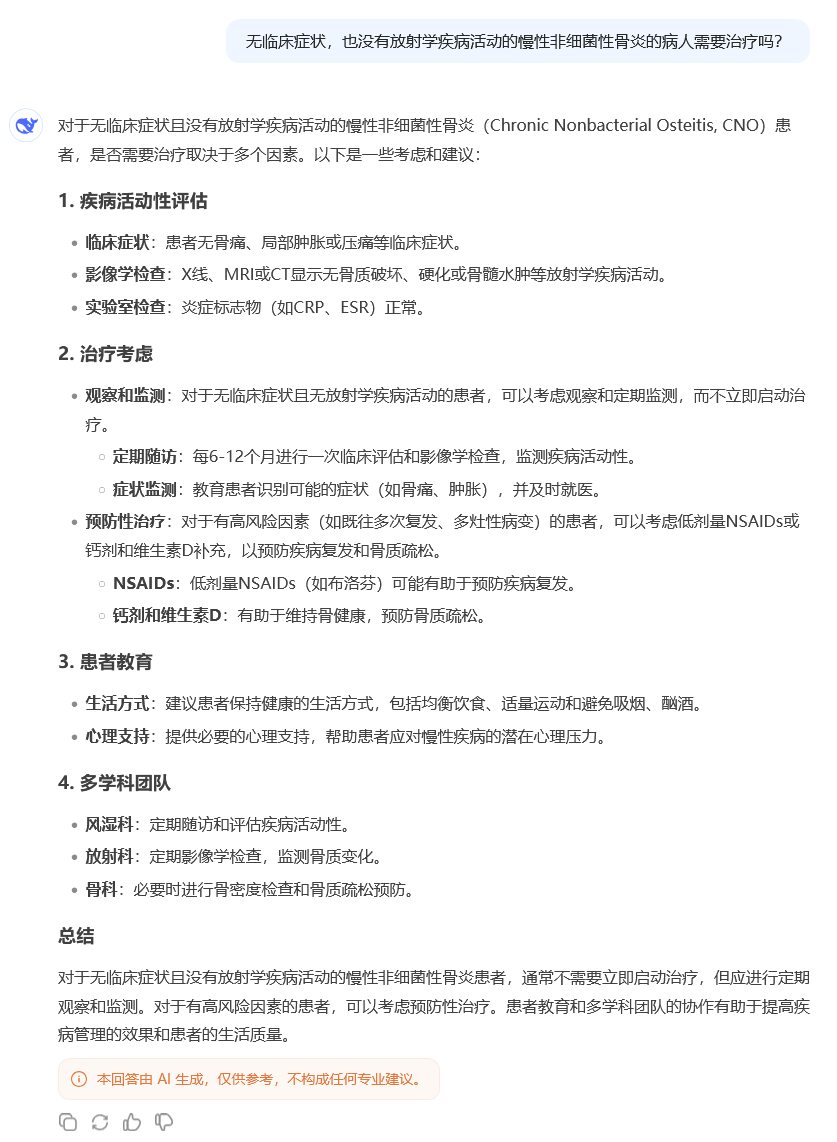
对于无临床症状且没有放射学疾病活动的慢性非细菌性骨炎（Chronic Nonbacterial Osteitis, CNO）患者，是否需要治疗取决于多个因素。以下是一些考虑和建议：

### 1. **疾病活动性评估**

- **临床症状**：患者无骨痛、局部肿胀或压痛等临床症状。

- **影像学检查**：X线、MRI或CT显示无骨质破坏、硬化或骨髓水肿等放射学疾病活动。

- **实验室检查**：炎症标志物（如CRP、ESR）正常。

### 2. **治疗考虑**

- **观察和监测**：对于无临床症状且无放射学疾病活动的患者，可以考虑观察和定期监测，而不立即启动治疗。

- **定期随访**：每6-12个月进行一次临床评估和影像学检查，监测疾病活动性。

- **症状监测**：教育患者识别可能的症状（如骨痛、肿胀），并及时就医。

- **预防性治疗**：对于有高风险因素（如既往多次复发、多灶性病变）的患者，可以考虑低剂量NSAIDs或钙剂和维生素D补充，以预防疾病复发和骨质疏松。

- **NSAIDs**：低剂量NSAIDs（如布洛芬）可能有助于预防疾病复发。

- **钙剂和维生素D**：有助于维持骨健康，预防骨质疏松。

### 3. **患者教育**

- **生活方式**：建议患者保持健康的生活方式，包括均衡饮食、适量运动和避免吸烟、酗酒。

- **心理支持**：提供必要的心理支持，帮助患者应对慢性疾病的潜在心理压力。

### 4. **多学科团队**

- **风湿科**：定期随访和评估疾病活动性。

- **放射科**：定期影像学检查，监测骨质变化。

- **骨科**：必要时进行骨密度检查和骨质疏松预防。

### 总结

对于无临床症状且没有放射学疾病活动的慢性非细菌性骨炎患者，通常不需要立即启动治疗，但应进行定期观察和监测。对于有高风险因素的患者，可以考虑预防性治疗。患者教育和多学科团队的协作有助于提高疾病管理的效果和患者的生活质量。

15有临床症状没有放射学疾病活动的慢性非细菌性骨炎的病人如何处理？


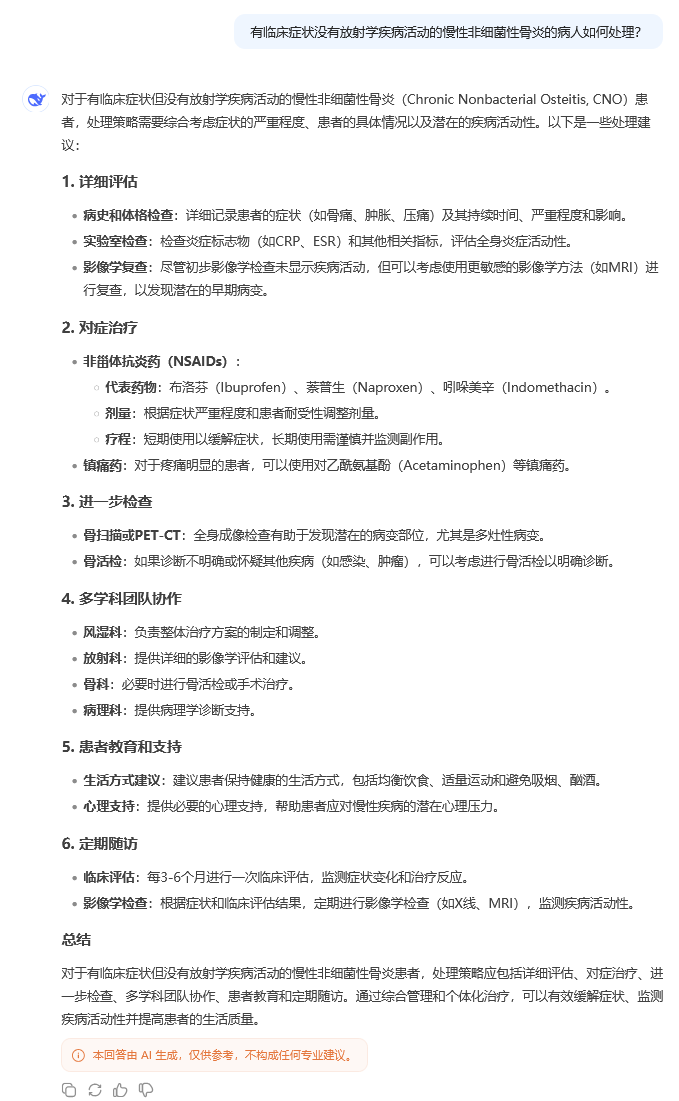
对于有临床症状但没有放射学疾病活动的慢性非细菌性骨炎（Chronic Nonbacterial Osteitis, CNO）患者，处理策略需要综合考虑症状的严重程度、患者的具体情况以及潜在的疾病活动性。以下是一些处理建议：

### 1. **详细评估**

- **病史和体格检查**：详细记录患者的症状（如骨痛、肿胀、压痛）及其持续时间、严重程度和影响。

- **实验室检查**：检查炎症标志物（如CRP、ESR）和其他相关指标，评估全身炎症活动性。

- **影像学复查**：尽管初步影像学检查未显示疾病活动，但可以考虑使用更敏感的影像学方法（如MRI）进行复查，以发现潜在的早期病变。

### 2. **对症治疗**

- **非甾体抗炎药（NSAIDs）**：

- **代表药物**：布洛芬（Ibuprofen）、萘普生（Naproxen）、吲哚美辛（Indomethacin）。

- **剂量**：根据症状严重程度和患者耐受性调整剂量。

- **疗程**：短期使用以缓解症状，长期使用需谨慎并监测副作用。

- **镇痛药**：对于疼痛明显的患者，可以使用对乙酰氨基酚（Acetaminophen）等镇痛药。

### 3. **进一步检查**

- **骨扫描或PET-CT**：全身成像检查有助于发现潜在的病变部位，尤其是多灶性病变。

- **骨活检**：如果诊断不明确或怀疑其他疾病（如感染、肿瘤），可以考虑进行骨活检以明确诊断。

### 4. **多学科团队协作**

- **风湿科**：负责整体治疗方案的制定和调整。

- **放射科**：提供详细的影像学评估和建议。

- **骨科**：必要时进行骨活检或手术治疗。

- **病理科**：提供病理学诊断支持。

### 5. **患者教育和支持**

- **生活方式建议**：建议患者保持健康的生活方式，包括均衡饮食、适量运动和避免吸烟、酗酒。

- **心理支持**：提供必要的心理支持，帮助患者应对慢性疾病的潜在心理压力。

### 6. **定期随访**

- **临床评估**：每3-6个月进行一次临床评估，监测症状变化和治疗反应。

- **影像学检查**：根据症状和临床评估结果，定期进行影像学检查（如X线、MRI），监测疾病活动性。

### 总结

对于有临床症状但没有放射学疾病活动的慢性非细菌性骨炎患者，处理策略应包括详细评估、对症治疗、进一步检查、多学科团队协作、患者教育和定期随访。通过综合管理和个体化治疗，可以有效缓解症状、监测疾病活动性并提高患者的生活质量。

16有椎体塌陷风险的脊柱骨病变的慢性非细菌性骨炎患者和出现明显骨骼累积损伤的慢性非细菌性骨炎患者如何治疗？


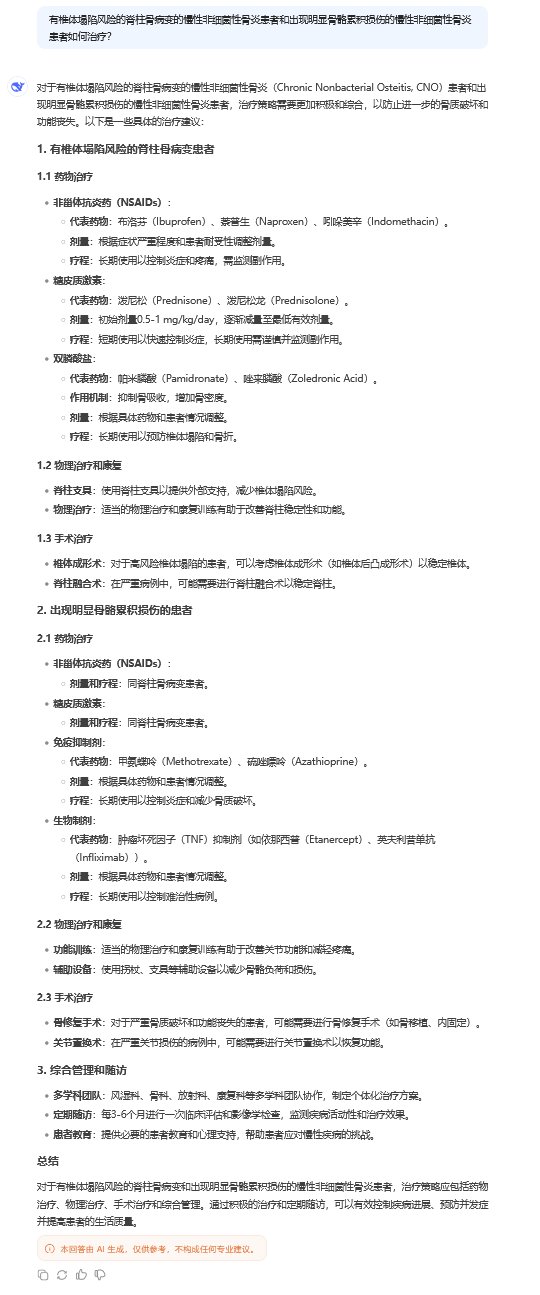


对于有椎体塌陷风险的脊柱骨病变的慢性非细菌性骨炎（Chronic Nonbacterial Osteitis, CNO）患者和出现明显骨骼累积损伤的慢性非细菌性骨炎患者，治疗策略需要更加积极和综合，以防止进一步的骨质破坏和功能丧失。以下是一些具体的治疗建议：

### 1. **有椎体塌陷风险的脊柱骨病变患者**

#### 1.1 **药物治疗**

- **非甾体抗炎药（NSAIDs）**：

- **代表药物**：布洛芬（Ibuprofen）、萘普生（Naproxen）、吲哚美辛（Indomethacin）。

- **剂量**：根据症状严重程度和患者耐受性调整剂量。

- **疗程**：长期使用以控制炎症和疼痛，需监测副作用。

- **糖皮质激素**：

- **代表药物**：泼尼松（Prednisone）、泼尼松龙（Prednisolone）。

- **剂量**：初始剂量0.5-1 mg/kg/day，逐渐减量至最低有效剂量。

- **疗程**：短期使用以快速控制炎症，长期使用需谨慎并监测副作用。

- **双膦酸盐**：

- **代表药物**：帕米膦酸（Pamidronate）、唑来膦酸（Zoledronic Acid）。

- **作用机制**：抑制骨吸收，增加骨密度。

- **剂量**：根据具体药物和患者情况调整。

- **疗程**：长期使用以预防椎体塌陷和骨折。

#### 1.2 **物理治疗和康复**

- **脊柱支具**：使用脊柱支具以提供外部支持，减少椎体塌陷风险。

- **物理治疗**：适当的物理治疗和康复训练有助于改善脊柱稳定性和功能。

#### 1.3 **手术治疗**

- **椎体成形术**：对于高风险椎体塌陷的患者，可以考虑椎体成形术（如椎体后凸成形术）以稳定椎体。

- **脊柱融合术**：在严重病例中，可能需要进行脊柱融合术以稳定脊柱。

### 2. **出现明显骨骼累积损伤的患者**

#### 2.1 **药物治疗**

- **非甾体抗炎药（NSAIDs）**：

- **剂量和疗程**：同脊柱骨病变患者。

- **糖皮质激素**：

- **剂量和疗程**：同脊柱骨病变患者。

- **免疫抑制剂**：

- **代表药物**：甲氨蝶呤（Methotrexate）、硫唑嘌呤（Azathioprine）。

- **剂量**：根据具体药物和患者情况调整。

- **疗程**：长期使用以控制炎症和减少骨质破坏。

- **生物制剂**：

- **代表药物**：肿瘤坏死因子（TNF）抑制剂（如依那西普（Etanercept）、英夫利昔单抗（Infliximab））。

- **剂量**：根据具体药物和患者情况调整。

- **疗程**：长期使用以控制难治性病例。

#### 2.2 **物理治疗和康复**

- **功能训练**：适当的物理治疗和康复训练有助于改善关节功能和减轻疼痛。

- **辅助设备**：使用拐杖、支具等辅助设备以减少骨骼负荷和损伤。

#### 2.3 **手术治疗**

- **骨修复手术**：对于严重骨质破坏和功能丧失的患者，可能需要进行骨修复手术（如骨移植、内固定）。

- **关节置换术**：在严重关节损伤的病例中，可能需要进行关节置换术以恢复功能。

### 3. **综合管理和随访**

- **多学科团队**：风湿科、骨科、放射科、康复科等多学科团队协作，制定个体化治疗方案。

- **定期随访**：每3-6个月进行一次临床评估和影像学检查，监测疾病活动性和治疗效果。

- **患者教育**：提供必要的患者教育和心理支持，帮助患者应对慢性疾病的挑战。

### 总结

对于有椎体塌陷风险的脊柱骨病变和出现明显骨骼累积损伤的慢性非细菌性骨炎患者，治疗策略应包括药物治疗、物理治疗、手术治疗和综合管理。通过积极的治疗和定期随访，可以有效控制疾病进展、预防并发症并提高患者的生活质量。
