# Supplementary material for: Evaluation of the accuracy and repeatability of Deepseek V3, Doubao, and Kimi1.5 in answering knowledge-related queries about chronic non-bacterial osteitis
Source: Front Artif Intell. 2025 Sep 29;8:1629149. doi: 10.3389/frai.2025.1629149 (PMC12515971; doi:10.3389/frai.2025.1629149)
Supplement: Supplementary file 1 [file Data_Sheet_1.zip › day28/day28-Doubao.docx]

1成人慢性非细菌性骨炎的定义是什么？


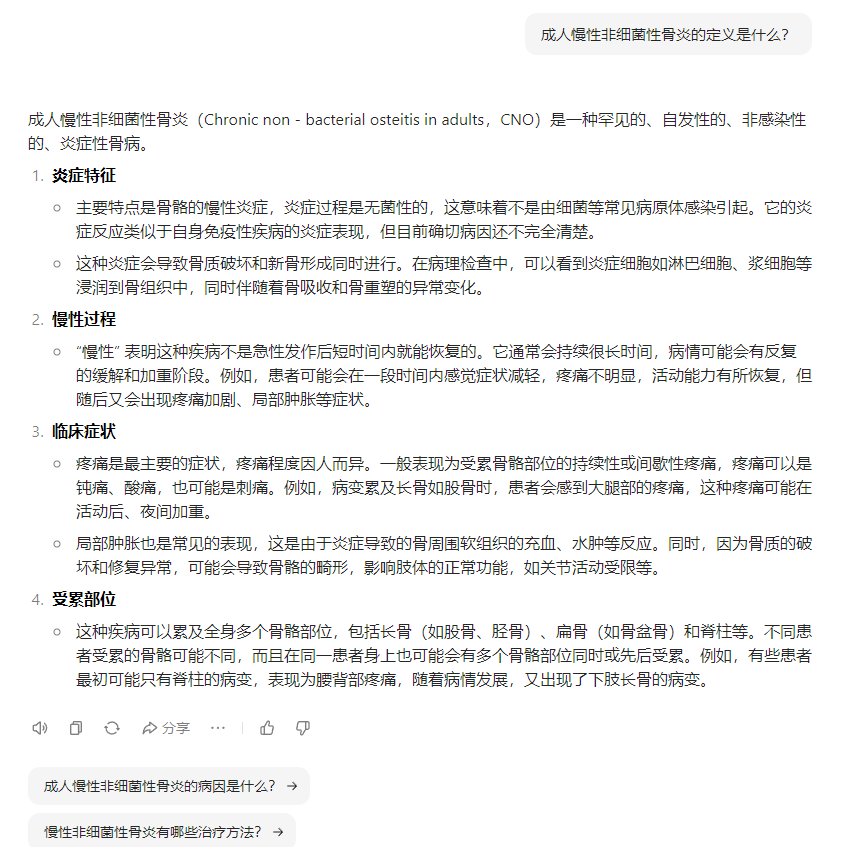


成人慢性非细菌性骨炎（Chronic non - bacterial osteitis in adults，CNO）是一种罕见的、自发性的、非感染性的、炎症性骨病。

1. **炎症特征**

- 主要特点是骨骼的慢性炎症，炎症过程是无菌性的，这意味着不是由细菌等常见病原体感染引起。它的炎症反应类似于自身免疫性疾病的炎症表现，但目前确切病因还不完全清楚。

- 这种炎症会导致骨质破坏和新骨形成同时进行。在病理检查中，可以看到炎症细胞如淋巴细胞、浆细胞等浸润到骨组织中，同时伴随着骨吸收和骨重塑的异常变化。

2. **慢性过程**

- “慢性”表明这种疾病不是急性发作后短时间内就能恢复的。它通常会持续很长时间，病情可能会有反复的缓解和加重阶段。例如，患者可能会在一段时间内感觉症状减轻，疼痛不明显，活动能力有所恢复，但随后又会出现疼痛加剧、局部肿胀等症状。

3. **临床症状**

- 疼痛是最主要的症状，疼痛程度因人而异。一般表现为受累骨骼部位的持续性或间歇性疼痛，疼痛可以是钝痛、酸痛，也可能是刺痛。例如，病变累及长骨如股骨时，患者会感到大腿部的疼痛，这种疼痛可能在活动后、夜间加重。

- 局部肿胀也是常见的表现，这是由于炎症导致的骨周围软组织的充血、水肿等反应。同时，因为骨质的破坏和修复异常，可能会导致骨骼的畸形，影响肢体的正常功能，如关节活动受限等。

4. **受累部位**

- 这种疾病可以累及全身多个骨骼部位，包括长骨（如股骨、胫骨）、扁骨（如骨盆骨）和脊柱等。不同患者受累的骨骼可能不同，而且在同一患者身上也可能会有多个骨骼部位同时或先后受累。例如，有些患者最初可能只有脊柱的病变，表现为腰背部疼痛，随着病情发展，又出现了下肢长骨的病变。

2成人慢性非细菌性骨炎的典型影像学特征是什么？


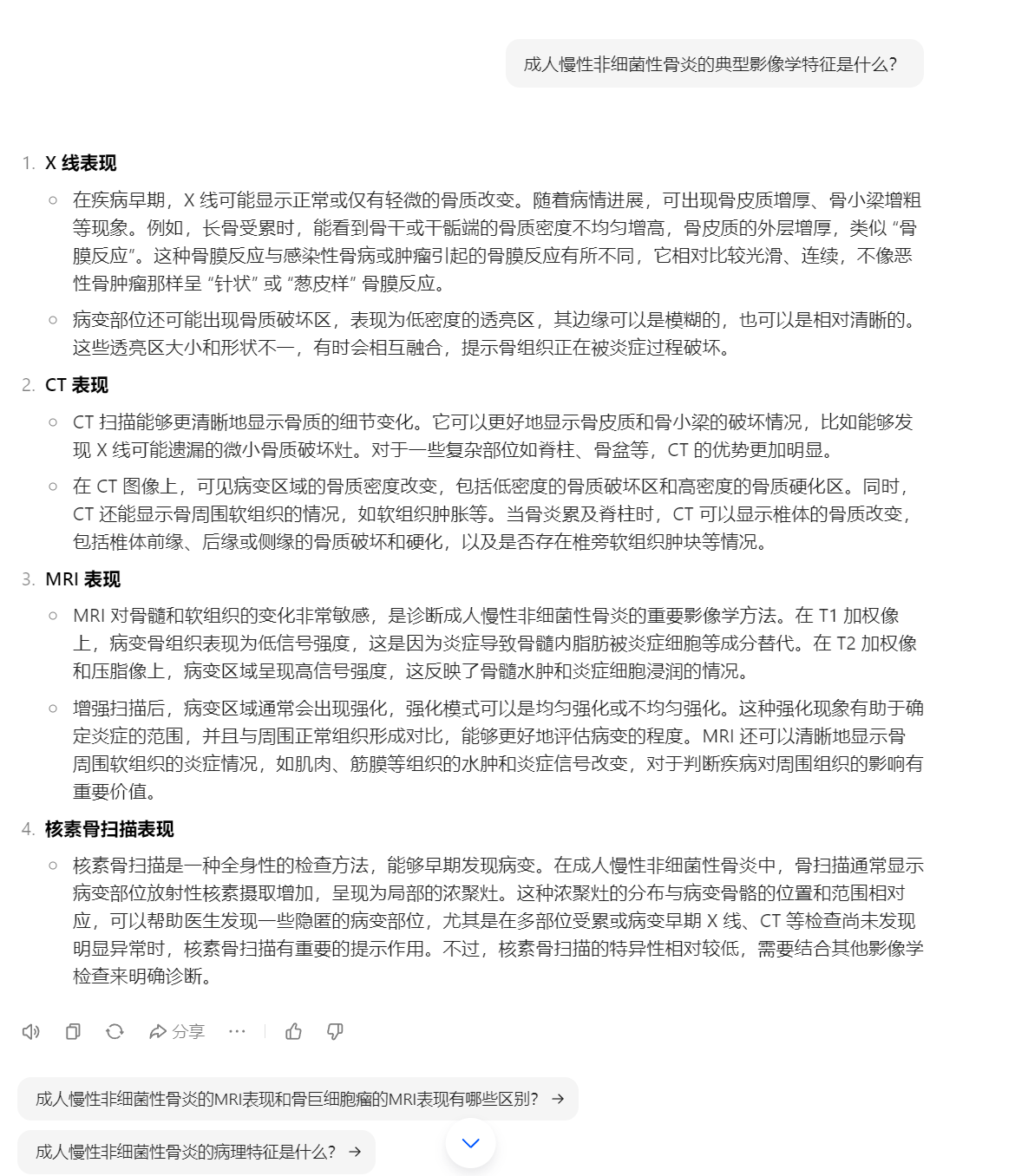
 1. **X线表现**

- 在疾病早期，X线可能显示正常或仅有轻微的骨质改变。随着病情进展，可出现骨皮质增厚、骨小梁增粗等现象。例如，长骨受累时，能看到骨干或干骺端的骨质密度不均匀增高，骨皮质的外层增厚，类似“骨膜反应”。这种骨膜反应与感染性骨病或肿瘤引起的骨膜反应有所不同，它相对比较光滑、连续，不像恶性骨肿瘤那样呈“针状”或“葱皮样”骨膜反应。

- 病变部位还可能出现骨质破坏区，表现为低密度的透亮区，其边缘可以是模糊的，也可以是相对清晰的。这些透亮区大小和形状不一，有时会相互融合，提示骨组织正在被炎症过程破坏。

2. **CT表现**

- CT扫描能够更清晰地显示骨质的细节变化。它可以更好地显示骨皮质和骨小梁的破坏情况，比如能够发现X线可能遗漏的微小骨质破坏灶。对于一些复杂部位如脊柱、骨盆等，CT的优势更加明显。

- 在CT图像上，可见病变区域的骨质密度改变，包括低密度的骨质破坏区和高密度的骨质硬化区。同时，CT还能显示骨周围软组织的情况，如软组织肿胀等。当骨炎累及脊柱时，CT可以显示椎体的骨质改变，包括椎体前缘、后缘或侧缘的骨质破坏和硬化，以及是否存在椎旁软组织肿块等情况。

3. **MRI表现**

- MRI对骨髓和软组织的变化非常敏感，是诊断成人慢性非细菌性骨炎的重要影像学方法。在T1加权像上，病变骨组织表现为低信号强度，这是因为炎症导致骨髓内脂肪被炎症细胞等成分替代。在T2加权像和压脂像上，病变区域呈现高信号强度，这反映了骨髓水肿和炎症细胞浸润的情况。

- 增强扫描后，病变区域通常会出现强化，强化模式可以是均匀强化或不均匀强化。这种强化现象有助于确定炎症的范围，并且与周围正常组织形成对比，能够更好地评估病变的程度。MRI还可以清晰地显示骨周围软组织的炎症情况，如肌肉、筋膜等组织的水肿和炎症信号改变，对于判断疾病对周围组织的影响有重要价值。

4. **核素骨扫描表现**

- 核素骨扫描是一种全身性的检查方法，能够早期发现病变。在成人慢性非细菌性骨炎中，骨扫描通常显示病变部位放射性核素摄取增加，呈现为局部的浓聚灶。这种浓聚灶的分布与病变骨骼的位置和范围相对应，可以帮助医生发现一些隐匿的病变部位，尤其是在多部位受累或病变早期X线、CT等检查尚未发现明显异常时，核素骨扫描有重要的提示作用。不过，核素骨扫描的特异性相对较低，需要结合其他影像学检查来明确诊断。

3成人慢性非细菌性骨炎最常见的表现是什么？


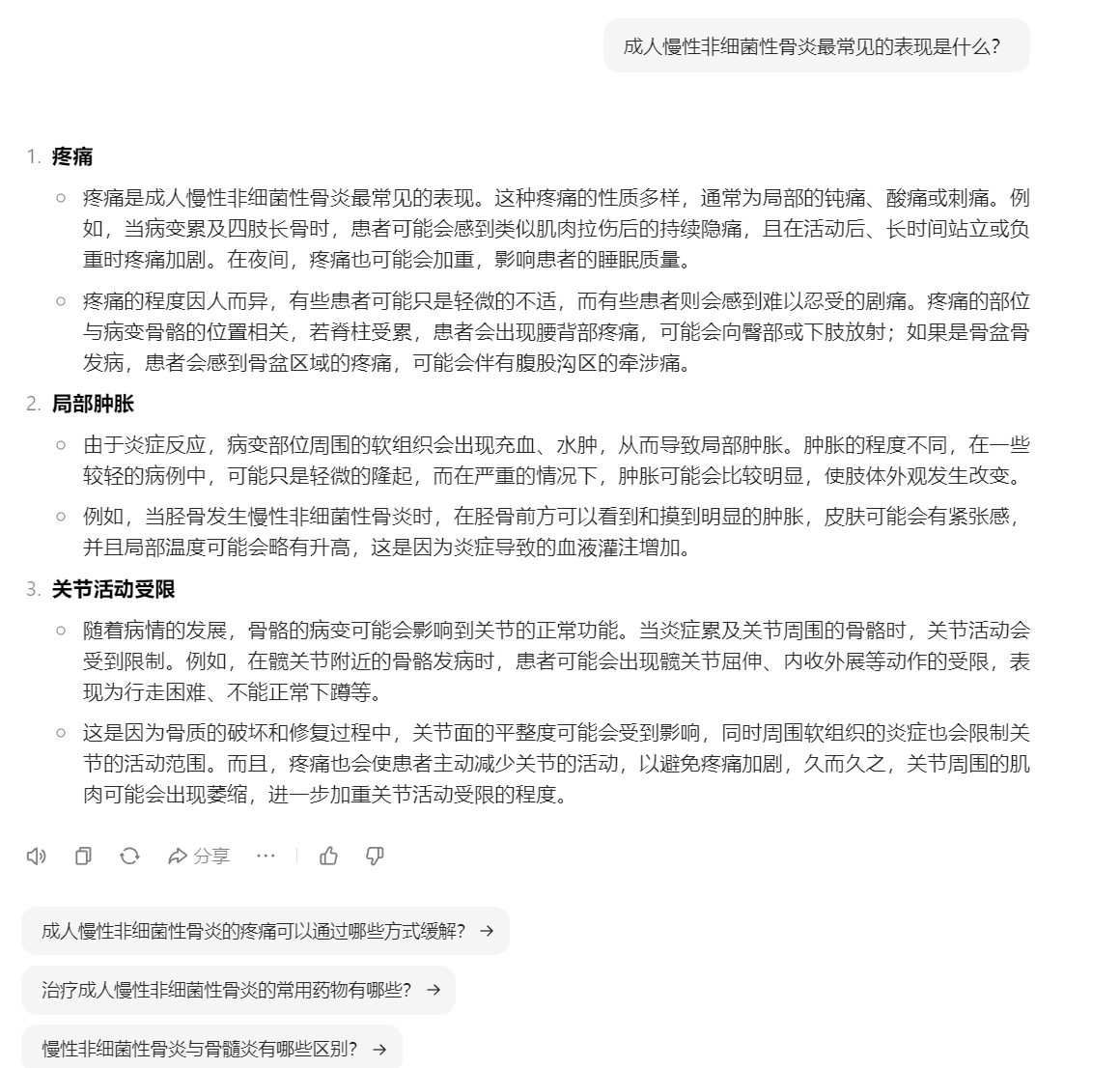
 1. **疼痛**

- 疼痛是成人慢性非细菌性骨炎最常见的表现。这种疼痛的性质多样，通常为局部的钝痛、酸痛或刺痛。例如，当病变累及四肢长骨时，患者可能会感到类似肌肉拉伤后的持续隐痛，且在活动后、长时间站立或负重时疼痛加剧。在夜间，疼痛也可能会加重，影响患者的睡眠质量。

- 疼痛的程度因人而异，有些患者可能只是轻微的不适，而有些患者则会感到难以忍受的剧痛。疼痛的部位与病变骨骼的位置相关，若脊柱受累，患者会出现腰背部疼痛，可能会向臀部或下肢放射；如果是骨盆骨发病，患者会感到骨盆区域的疼痛，可能会伴有腹股沟区的牵涉痛。

2. **局部肿胀**

- 由于炎症反应，病变部位周围的软组织会出现充血、水肿，从而导致局部肿胀。肿胀的程度不同，在一些较轻的病例中，可能只是轻微的隆起，而在严重的情况下，肿胀可能会比较明显，使肢体外观发生改变。

- 例如，当胫骨发生慢性非细菌性骨炎时，在胫骨前方可以看到和摸到明显的肿胀，皮肤可能会有紧张感，并且局部温度可能会略有升高，这是因为炎症导致的血液灌注增加。

3. **关节活动受限**

- 随着病情的发展，骨骼的病变可能会影响到关节的正常功能。当炎症累及关节周围的骨骼时，关节活动会受到限制。例如，在髋关节附近的骨骼发病时，患者可能会出现髋关节屈伸、内收外展等动作的受限，表现为行走困难、不能正常下蹲等。

- 这是因为骨质的破坏和修复过程中，关节面的平整度可能会受到影响，同时周围软组织的炎症也会限制关节的活动范围。而且，疼痛也会使患者主动减少关节的活动，以避免疼痛加剧，久而久之，关节周围的肌肉可能会出现萎缩，进一步加重关节活动受限的程度。

4成人慢性非细菌性骨炎最常受累的部位是那些？


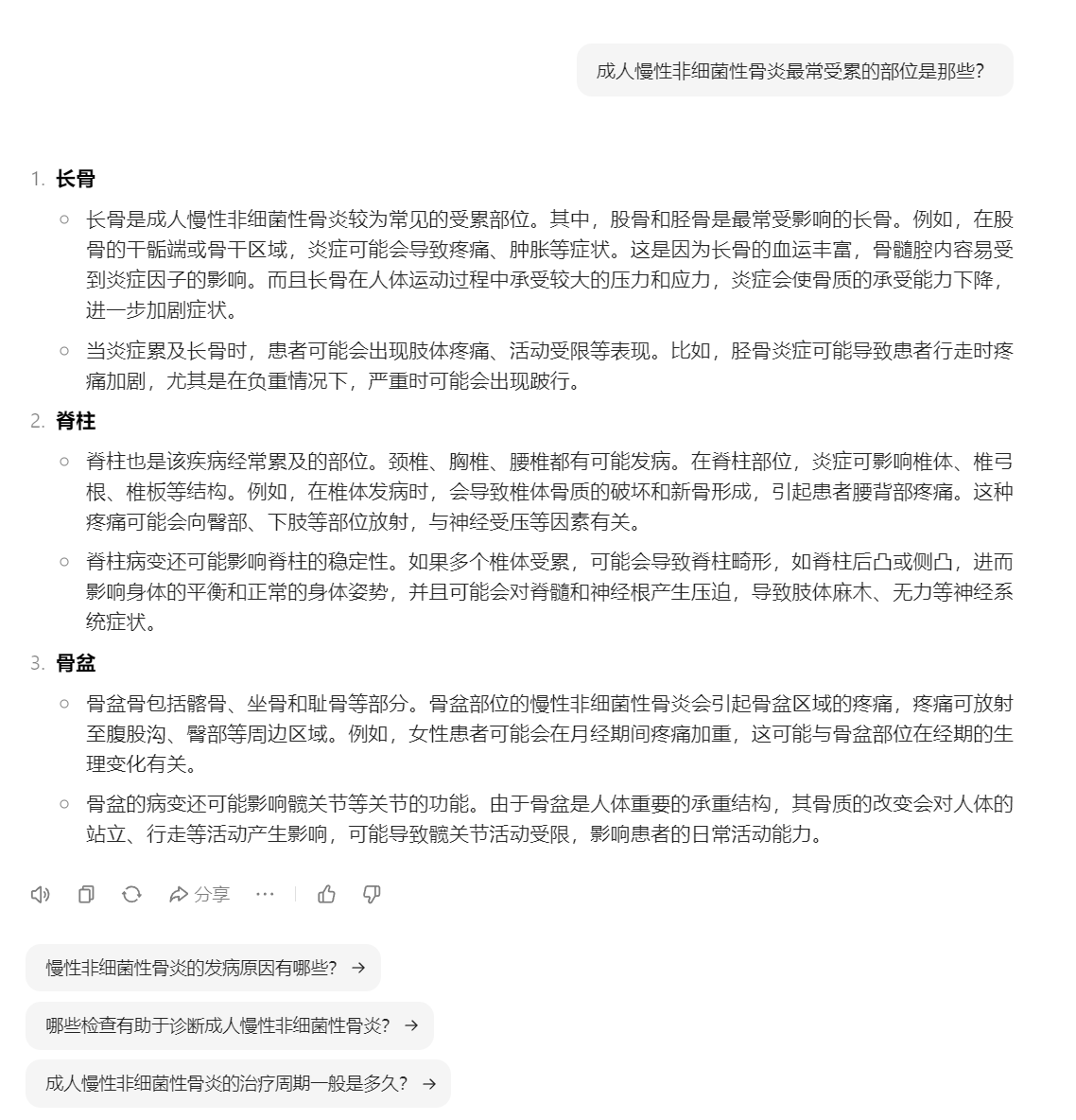
 1. **长骨**

- 长骨是成人慢性非细菌性骨炎较为常见的受累部位。其中，股骨和胫骨是最常受影响的长骨。例如，在股骨的干骺端或骨干区域，炎症可能会导致疼痛、肿胀等症状。这是因为长骨的血运丰富，骨髓腔内容易受到炎症因子的影响。而且长骨在人体运动过程中承受较大的压力和应力，炎症会使骨质的承受能力下降，进一步加剧症状。

- 当炎症累及长骨时，患者可能会出现肢体疼痛、活动受限等表现。比如，胫骨炎症可能导致患者行走时疼痛加剧，尤其是在负重情况下，严重时可能会出现跛行。

2. **脊柱**

- 脊柱也是该疾病经常累及的部位。颈椎、胸椎、腰椎都有可能发病。在脊柱部位，炎症可影响椎体、椎弓根、椎板等结构。例如，在椎体发病时，会导致椎体骨质的破坏和新骨形成，引起患者腰背部疼痛。这种疼痛可能会向臀部、下肢等部位放射，与神经受压等因素有关。

- 脊柱病变还可能影响脊柱的稳定性。如果多个椎体受累，可能会导致脊柱畸形，如脊柱后凸或侧凸，进而影响身体的平衡和正常的身体姿势，并且可能会对脊髓和神经根产生压迫，导致肢体麻木、无力等神经系统症状。

3. **骨盆**

- 骨盆骨包括髂骨、坐骨和耻骨等部分。骨盆部位的慢性非细菌性骨炎会引起骨盆区域的疼痛，疼痛可放射至腹股沟、臀部等周边区域。例如，女性患者可能会在月经期间疼痛加重，这可能与骨盆部位在经期的生理变化有关。

- 骨盆的病变还可能影响髋关节等关节的功能。由于骨盆是人体重要的承重结构，其骨质的改变会对人体的站立、行走等活动产生影响，可能导致髋关节活动受限，影响患者的日常活动能力。

5成人慢性非细菌性骨炎有没有特异性的炎症标志物？


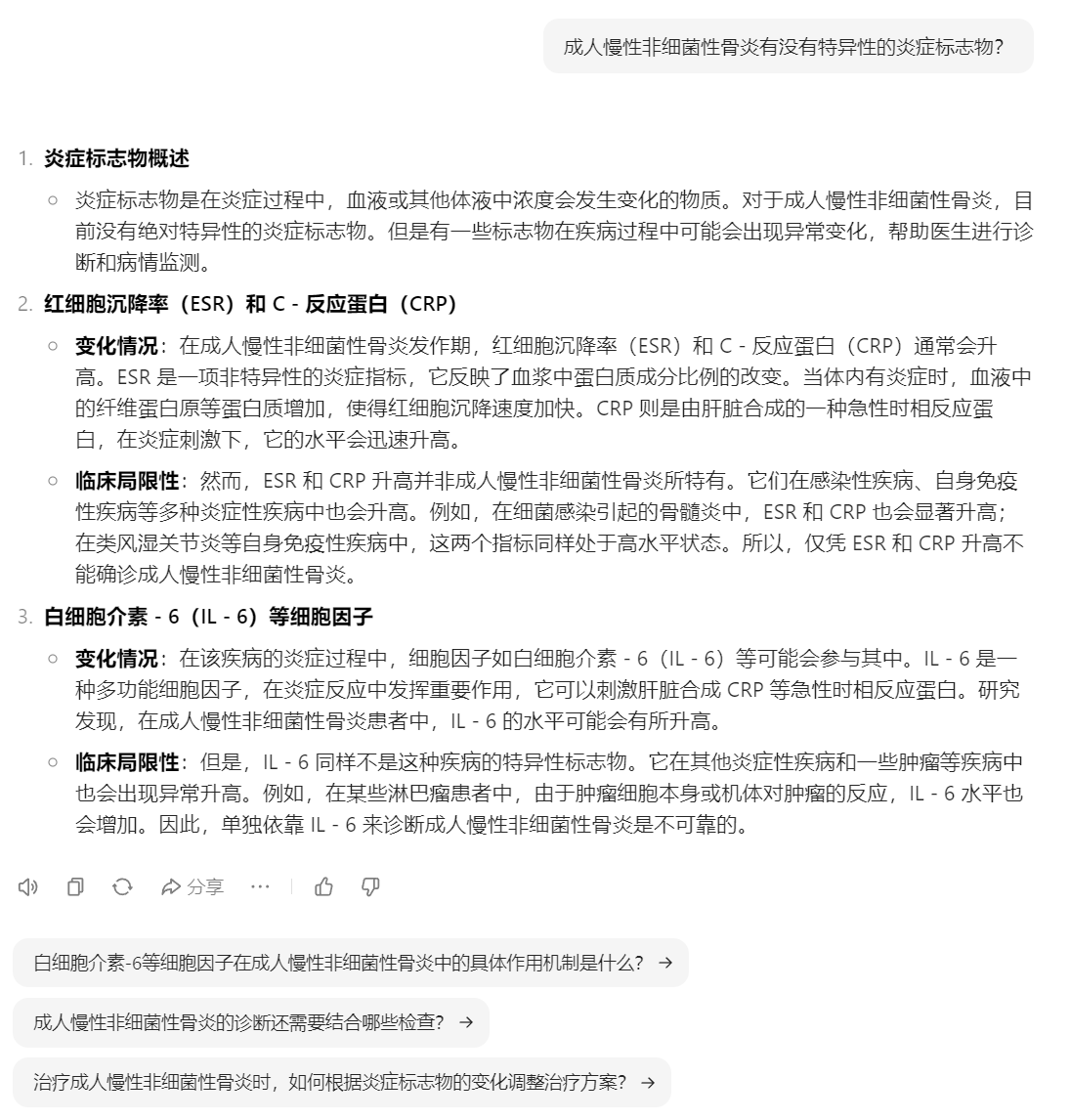
 1. **炎症标志物概述**

- 炎症标志物是在炎症过程中，血液或其他体液中浓度会发生变化的物质。对于成人慢性非细菌性骨炎，目前没有绝对特异性的炎症标志物。但是有一些标志物在疾病过程中可能会出现异常变化，帮助医生进行诊断和病情监测。

2. **红细胞沉降率（ESR）和C - 反应蛋白（CRP）**

- **变化情况**：在成人慢性非细菌性骨炎发作期，红细胞沉降率（ESR）和C - 反应蛋白（CRP）通常会升高。ESR是一项非特异性的炎症指标，它反映了血浆中蛋白质成分比例的改变。当体内有炎症时，血液中的纤维蛋白原等蛋白质增加，使得红细胞沉降速度加快。CRP则是由肝脏合成的一种急性时相反应蛋白，在炎症刺激下，它的水平会迅速升高。

- **临床局限性**：然而，ESR和CRP升高并非成人慢性非细菌性骨炎所特有。它们在感染性疾病、自身免疫性疾病等多种炎症性疾病中也会升高。例如，在细菌感染引起的骨髓炎中，ESR和CRP也会显著升高；在类风湿关节炎等自身免疫性疾病中，这两个指标同样处于高水平状态。所以，仅凭ESR和CRP升高不能确诊成人慢性非细菌性骨炎。

3. **白细胞介素 - 6（IL - 6）等细胞因子**

- **变化情况**：在该疾病的炎症过程中，细胞因子如白细胞介素 - 6（IL - 6）等可能会参与其中。IL - 6是一种多功能细胞因子，在炎症反应中发挥重要作用，它可以刺激肝脏合成CRP等急性时相反应蛋白。研究发现，在成人慢性非细菌性骨炎患者中，IL - 6的水平可能会有所升高。

- **临床局限性**：但是，IL - 6同样不是这种疾病的特异性标志物。它在其他炎症性疾病和一些肿瘤等疾病中也会出现异常升高。例如，在某些淋巴瘤患者中，由于肿瘤细胞本身或机体对肿瘤的反应，IL - 6水平也会增加。因此，单独依靠IL - 6来诊断成人慢性非细菌性骨炎是不可靠的。

6慢性非细菌性骨炎首选的影像学检查是什么？


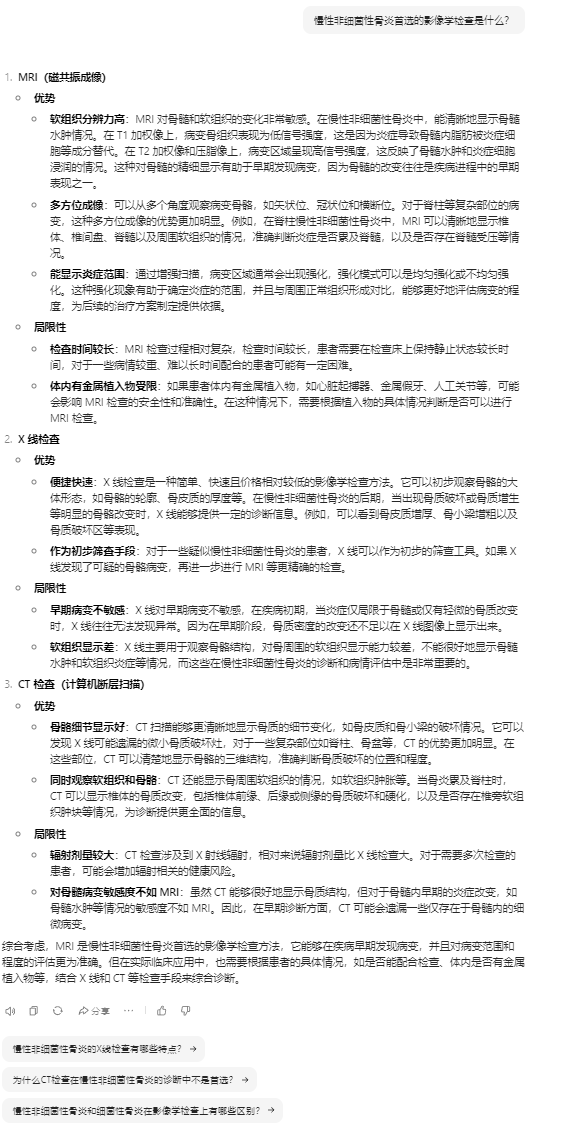
 1. **MRI（磁共振成像）**

- **优势**

- **软组织分辨力高**：MRI对骨髓和软组织的变化非常敏感。在慢性非细菌性骨炎中，能清晰地显示骨髓水肿情况。在T1加权像上，病变骨组织表现为低信号强度，这是因为炎症导致骨髓内脂肪被炎症细胞等成分替代。在T2加权像和压脂像上，病变区域呈现高信号强度，这反映了骨髓水肿和炎症细胞浸润的情况。这种对骨髓的精细显示有助于早期发现病变，因为骨髓的改变往往是疾病进程中的早期表现之一。

- **多方位成像**：可以从多个角度观察病变骨骼，如矢状位、冠状位和横断位。对于脊柱等复杂部位的病变，这种多方位成像的优势更加明显。例如，在脊柱慢性非细菌性骨炎中，MRI可以清晰地显示椎体、椎间盘、脊髓以及周围软组织的情况，准确判断炎症是否累及脊髓，以及是否存在脊髓受压等情况。

- **能显示炎症范围**：通过增强扫描，病变区域通常会出现强化，强化模式可以是均匀强化或不均匀强化。这种强化现象有助于确定炎症的范围，并且与周围正常组织形成对比，能够更好地评估病变的程度，为后续的治疗方案制定提供依据。

- **局限性**

- **检查时间较长**：MRI检查过程相对复杂，检查时间较长，患者需要在检查床上保持静止状态较长时间，对于一些病情较重、难以长时间配合的患者可能有一定困难。

- **体内有金属植入物受限**：如果患者体内有金属植入物，如心脏起搏器、金属假牙、人工关节等，可能会影响MRI检查的安全性和准确性。在这种情况下，需要根据植入物的具体情况判断是否可以进行MRI检查。

2. **X线检查**

- **优势**

- **便捷快速**：X线检查是一种简单、快速且价格相对较低的影像学检查方法。它可以初步观察骨骼的大体形态，如骨骼的轮廓、骨皮质的厚度等。在慢性非细菌性骨炎的后期，当出现骨质破坏或骨质增生等明显的骨骼改变时，X线能够提供一定的诊断信息。例如，可以看到骨皮质增厚、骨小梁增粗以及骨质破坏区等表现。

- **作为初步筛查手段**：对于一些疑似慢性非细菌性骨炎的患者，X线可以作为初步的筛查工具。如果X线发现了可疑的骨骼病变，再进一步进行MRI等更精确的检查。

- **局限性**

- **早期病变不敏感**：X线对早期病变不敏感，在疾病初期，当炎症仅局限于骨髓或仅有轻微的骨质改变时，X线往往无法发现异常。因为在早期阶段，骨质密度的改变还不足以在X线图像上显示出来。

- **软组织显示差**：X线主要用于观察骨骼结构，对骨周围的软组织显示能力较差，不能很好地显示骨髓水肿和软组织炎症等情况，而这些在慢性非细菌性骨炎的诊断和病情评估中是非常重要的。

3. **CT检查（计算机断层扫描）**

- **优势**

- **骨骼细节显示好**：CT扫描能够更清晰地显示骨质的细节变化，如骨皮质和骨小梁的破坏情况。它可以发现X线可能遗漏的微小骨质破坏灶，对于一些复杂部位如脊柱、骨盆等，CT的优势更加明显。在这些部位，CT可以清楚地显示骨骼的三维结构，准确判断骨质破坏的位置和程度。

- **同时观察软组织和骨骼**：CT还能显示骨周围软组织的情况，如软组织肿胀等。当骨炎累及脊柱时，CT可以显示椎体的骨质改变，包括椎体前缘、后缘或侧缘的骨质破坏和硬化，以及是否存在椎旁软组织肿块等情况，为诊断提供更全面的信息。

- **局限性**

- **辐射剂量较大**：CT检查涉及到X射线辐射，相对来说辐射剂量比X线检查大。对于需要多次检查的患者，可能会增加辐射相关的健康风险。

- **对骨髓病变敏感度不如MRI**：虽然CT能够很好地显示骨质结构，但对于骨髓内早期的炎症改变，如骨髓水肿等情况的敏感度不如MRI。因此，在早期诊断方面，CT可能会遗漏一些仅存在于骨髓内的细微病变。

综合考虑，MRI是慢性非细菌性骨炎首选的影像学检查方法，它能够在疾病早期发现病变，并且对病变范围和程度的评估更为准确。但在实际临床应用中，也需要根据患者的具体情况，如是否能配合检查、体内是否有金属植入物等，结合X线和CT等检查手段来综合诊断。

7全身成像检查是不是诊断成人慢性非细菌性骨炎的先决条件？


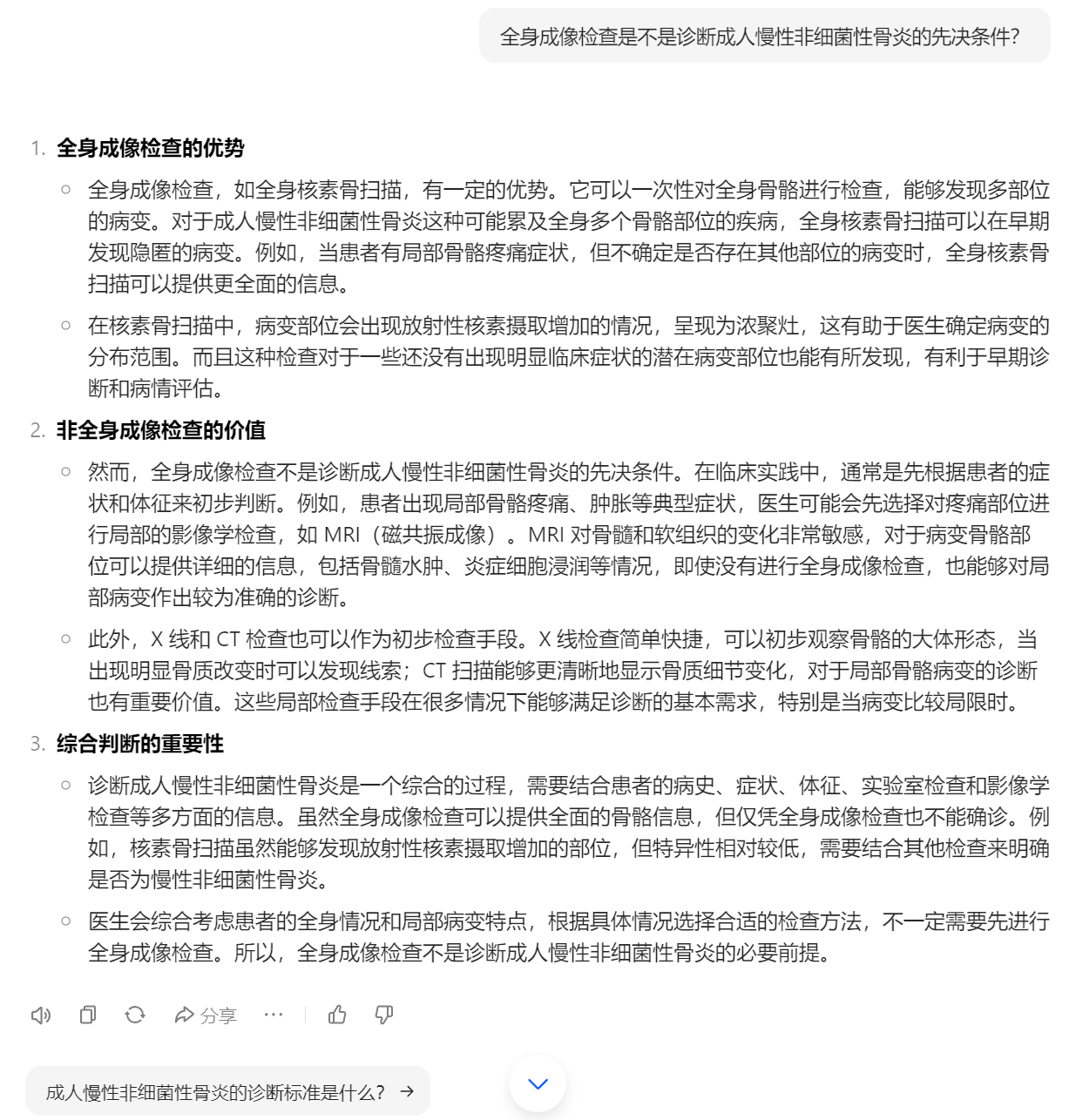
 1. **全身成像检查的优势**

- 全身成像检查，如全身核素骨扫描，有一定的优势。它可以一次性对全身骨骼进行检查，能够发现多部位的病变。对于成人慢性非细菌性骨炎这种可能累及全身多个骨骼部位的疾病，全身核素骨扫描可以在早期发现隐匿的病变。例如，当患者有局部骨骼疼痛症状，但不确定是否存在其他部位的病变时，全身核素骨扫描可以提供更全面的信息。

- 在核素骨扫描中，病变部位会出现放射性核素摄取增加的情况，呈现为浓聚灶，这有助于医生确定病变的分布范围。而且这种检查对于一些还没有出现明显临床症状的潜在病变部位也能有所发现，有利于早期诊断和病情评估。

2. **非全身成像检查的价值**

- 然而，全身成像检查不是诊断成人慢性非细菌性骨炎的先决条件。在临床实践中，通常是先根据患者的症状和体征来初步判断。例如，患者出现局部骨骼疼痛、肿胀等典型症状，医生可能会先选择对疼痛部位进行局部的影像学检查，如MRI（磁共振成像）。MRI对骨髓和软组织的变化非常敏感，对于病变骨骼部位可以提供详细的信息，包括骨髓水肿、炎症细胞浸润等情况，即使没有进行全身成像检查，也能够对局部病变作出较为准确的诊断。

- 此外，X线和CT检查也可以作为初步检查手段。X线检查简单快捷，可以初步观察骨骼的大体形态，当出现明显骨质改变时可以发现线索；CT扫描能够更清晰地显示骨质细节变化，对于局部骨骼病变的诊断也有重要价值。这些局部检查手段在很多情况下能够满足诊断的基本需求，特别是当病变比较局限时。

3. **综合判断的重要性**

- 诊断成人慢性非细菌性骨炎是一个综合的过程，需要结合患者的病史、症状、体征、实验室检查和影像学检查等多方面的信息。虽然全身成像检查可以提供全面的骨骼信息，但仅凭全身成像检查也不能确诊。例如，核素骨扫描虽然能够发现放射性核素摄取增加的部位，但特异性相对较低，需要结合其他检查来明确是否为慢性非细菌性骨炎。

- 医生会综合考虑患者的全身情况和局部病变特点，根据具体情况选择合适的检查方法，不一定需要先进行全身成像检查。所以，全身成像检查不是诊断成人慢性非细菌性骨炎的必要前提。

8诊断成人慢性非细菌性骨炎有必要常规骨活检吗？


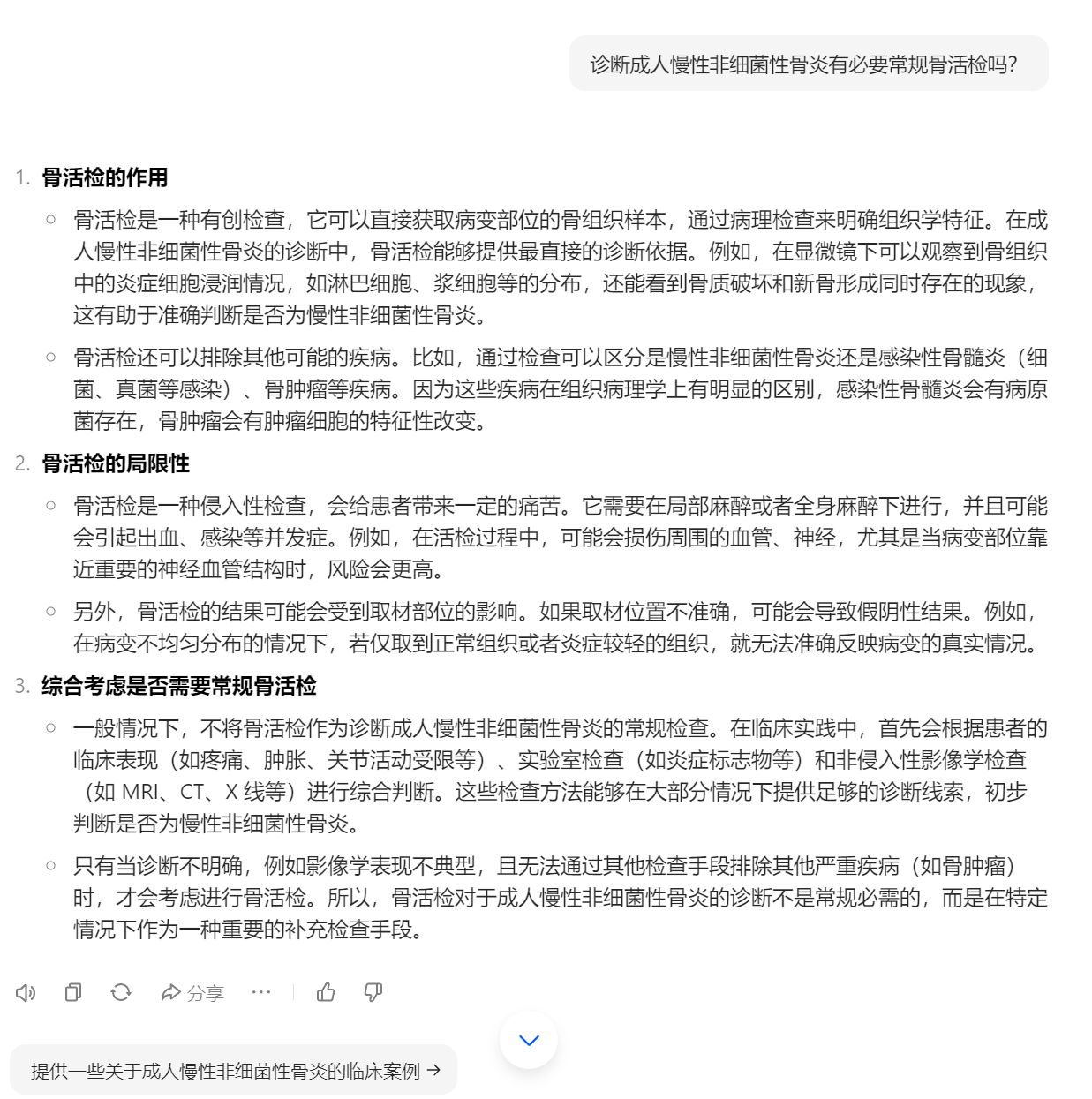
 1. **骨活检的作用**

- 骨活检是一种有创检查，它可以直接获取病变部位的骨组织样本，通过病理检查来明确组织学特征。在成人慢性非细菌性骨炎的诊断中，骨活检能够提供最直接的诊断依据。例如，在显微镜下可以观察到骨组织中的炎症细胞浸润情况，如淋巴细胞、浆细胞等的分布，还能看到骨质破坏和新骨形成同时存在的现象，这有助于准确判断是否为慢性非细菌性骨炎。

- 骨活检还可以排除其他可能的疾病。比如，通过检查可以区分是慢性非细菌性骨炎还是感染性骨髓炎（细菌、真菌等感染）、骨肿瘤等疾病。因为这些疾病在组织病理学上有明显的区别，感染性骨髓炎会有病原菌存在，骨肿瘤会有肿瘤细胞的特征性改变。

2. **骨活检的局限性**

- 骨活检是一种侵入性检查，会给患者带来一定的痛苦。它需要在局部麻醉或者全身麻醉下进行，并且可能会引起出血、感染等并发症。例如，在活检过程中，可能会损伤周围的血管、神经，尤其是当病变部位靠近重要的神经血管结构时，风险会更高。

- 另外，骨活检的结果可能会受到取材部位的影响。如果取材位置不准确，可能会导致假阴性结果。例如，在病变不均匀分布的情况下，若仅取到正常组织或者炎症较轻的组织，就无法准确反映病变的真实情况。

3. **综合考虑是否需要常规骨活检**

- 一般情况下，不将骨活检作为诊断成人慢性非细菌性骨炎的常规检查。在临床实践中，首先会根据患者的临床表现（如疼痛、肿胀、关节活动受限等）、实验室检查（如炎症标志物等）和非侵入性影像学检查（如MRI、CT、X线等）进行综合判断。这些检查方法能够在大部分情况下提供足够的诊断线索，初步判断是否为慢性非细菌性骨炎。

- 只有当诊断不明确，例如影像学表现不典型，且无法通过其他检查手段排除其他严重疾病（如骨肿瘤）时，才会考虑进行骨活检。所以，骨活检对于成人慢性非细菌性骨炎的诊断不是常规必需的，而是在特定情况下作为一种重要的补充检查手段。

9慢性非细菌性骨炎如何与恶性骨肿瘤鉴别？


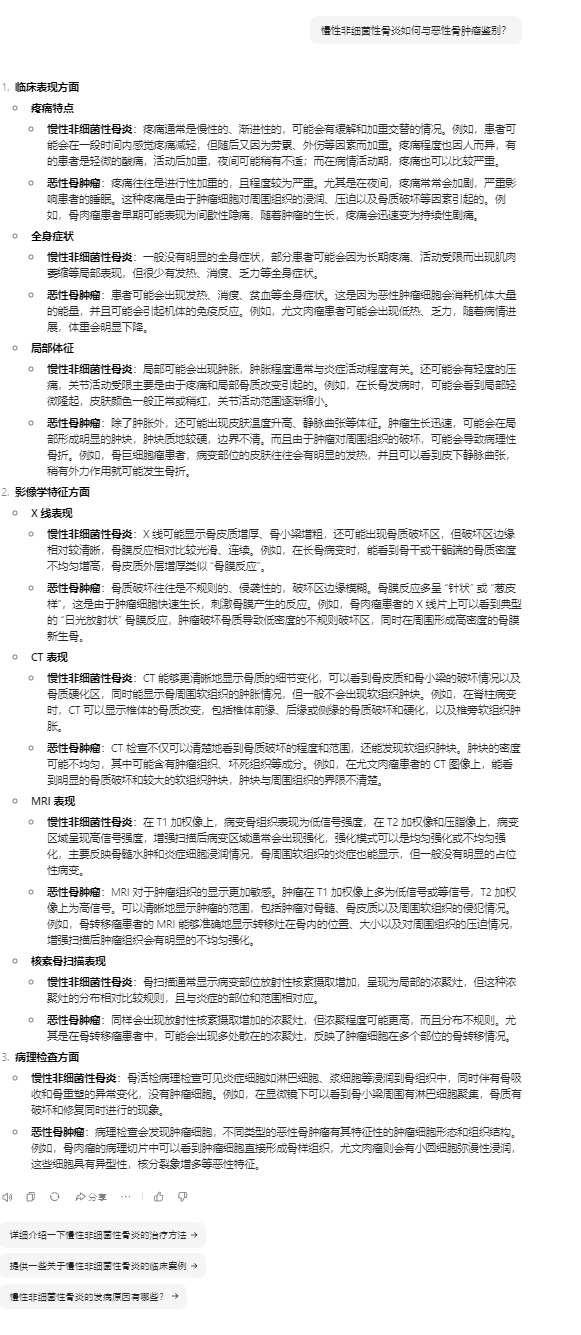
 1. **临床表现方面**

- **疼痛特点**

- **慢性非细菌性骨炎**：疼痛通常是慢性的、渐进性的，可能会有缓解和加重交替的情况。例如，患者可能会在一段时间内感觉疼痛减轻，但随后又因为劳累、外伤等因素而加重。疼痛程度也因人而异，有的患者是轻微的酸痛，活动后加重，夜间可能稍有不适；而在病情活动期，疼痛也可以比较严重。

- **恶性骨肿瘤**：疼痛往往是进行性加重的，且程度较为严重。尤其是在夜间，疼痛常常会加剧，严重影响患者的睡眠。这种疼痛是由于肿瘤细胞对周围组织的浸润、压迫以及骨质破坏等因素引起的。例如，骨肉瘤患者早期可能表现为间歇性隐痛，随着肿瘤的生长，疼痛会迅速变为持续性剧痛。

- **全身症状**

- **慢性非细菌性骨炎**：一般没有明显的全身症状，部分患者可能会因为长期疼痛、活动受限而出现肌肉萎缩等局部表现，但很少有发热、消瘦、乏力等全身症状。

- **恶性骨肿瘤**：患者可能会出现发热、消瘦、贫血等全身症状。这是因为恶性肿瘤细胞会消耗机体大量的能量，并且可能会引起机体的免疫反应。例如，尤文肉瘤患者可能会出现低热、乏力，随着病情进展，体重会明显下降。

- **局部体征**

- **慢性非细菌性骨炎**：局部可能会出现肿胀，肿胀程度通常与炎症活动程度有关。还可能会有轻度的压痛，关节活动受限主要是由于疼痛和局部骨质改变引起的。例如，在长骨发病时，可能会看到局部轻微隆起，皮肤颜色一般正常或稍红，关节活动范围逐渐缩小。

- **恶性骨肿瘤**：除了肿胀外，还可能出现皮肤温度升高、静脉曲张等体征。肿瘤生长迅速，可能会在局部形成明显的肿块，肿块质地较硬，边界不清。而且由于肿瘤对周围组织的破坏，可能会导致病理性骨折。例如，骨巨细胞瘤患者，病变部位的皮肤往往会有明显的发热，并且可以看到皮下静脉曲张，稍有外力作用就可能发生骨折。

2. **影像学特征方面**

- **X线表现**

- **慢性非细菌性骨炎**：X线可能显示骨皮质增厚、骨小梁增粗，还可能出现骨质破坏区，但破坏区边缘相对较清晰，骨膜反应相对比较光滑、连续。例如，在长骨病变时，能看到骨干或干骺端的骨质密度不均匀增高，骨皮质外层增厚类似“骨膜反应”。

- **恶性骨肿瘤**：骨质破坏往往是不规则的、侵袭性的，破坏区边缘模糊。骨膜反应多呈“针状”或“葱皮样”，这是由于肿瘤细胞快速生长，刺激骨膜产生的反应。例如，骨肉瘤患者的X线片上可以看到典型的“日光放射状”骨膜反应，肿瘤破坏骨质导致低密度的不规则破坏区，同时在周围形成高密度的骨膜新生骨。

- **CT表现**

- **慢性非细菌性骨炎**：CT能够更清晰地显示骨质的细节变化，可以看到骨皮质和骨小梁的破坏情况以及骨质硬化区，同时能显示骨周围软组织的肿胀情况，但一般不会出现软组织肿块。例如，在脊柱病变时，CT可以显示椎体的骨质改变，包括椎体前缘、后缘或侧缘的骨质破坏和硬化，以及椎旁软组织肿胀。

- **恶性骨肿瘤**：CT检查不仅可以清楚地看到骨质破坏的程度和范围，还能发现软组织肿块。肿块的密度可能不均匀，其中可能含有肿瘤组织、坏死组织等成分。例如，在尤文肉瘤患者的CT图像上，能看到明显的骨质破坏和较大的软组织肿块，肿块与周围组织的界限不清楚。

- **MRI表现**

- **慢性非细菌性骨炎**：在T1加权像上，病变骨组织表现为低信号强度，在T2加权像和压脂像上，病变区域呈现高信号强度，增强扫描后病变区域通常会出现强化，强化模式可以是均匀强化或不均匀强化，主要反映骨髓水肿和炎症细胞浸润情况，骨周围软组织的炎症也能显示，但一般没有明显的占位性病变。

- **恶性骨肿瘤**：MRI对于肿瘤组织的显示更加敏感。肿瘤在T1加权像上多为低信号或等信号，T2加权像上为高信号。可以清晰地显示肿瘤的范围，包括肿瘤对骨髓、骨皮质以及周围软组织的侵犯情况。例如，骨转移瘤患者的MRI能够准确地显示转移灶在骨内的位置、大小以及对周围组织的压迫情况，增强扫描后肿瘤组织会有明显的不均匀强化。

- **核素骨扫描表现**

- **慢性非细菌性骨炎**：骨扫描通常显示病变部位放射性核素摄取增加，呈现为局部的浓聚灶，但这种浓聚灶的分布相对比较规则，且与炎症的部位和范围相对应。

- **恶性骨肿瘤**：同样会出现放射性核素摄取增加的浓聚灶，但浓聚程度可能更高，而且分布不规则。尤其是在骨转移瘤患者中，可能会出现多处散在的浓聚灶，反映了肿瘤细胞在多个部位的骨转移情况。

3. **病理检查方面**

- **慢性非细菌性骨炎**：骨活检病理检查可见炎症细胞如淋巴细胞、浆细胞等浸润到骨组织中，同时伴有骨吸收和骨重塑的异常变化，没有肿瘤细胞。例如，在显微镜下可以看到骨小梁周围有淋巴细胞聚集，骨质有破坏和修复同时进行的现象。

- **恶性骨肿瘤**：病理检查会发现肿瘤细胞，不同类型的恶性骨肿瘤有其特征性的肿瘤细胞形态和组织结构。例如，骨肉瘤的病理切片中可以看到肿瘤细胞直接形成骨样组织，尤文肉瘤则会有小圆细胞弥漫性浸润，这些细胞具有异型性，核分裂象增多等恶性特征。

10慢性非细菌性骨炎如何与骨关节炎鉴别？


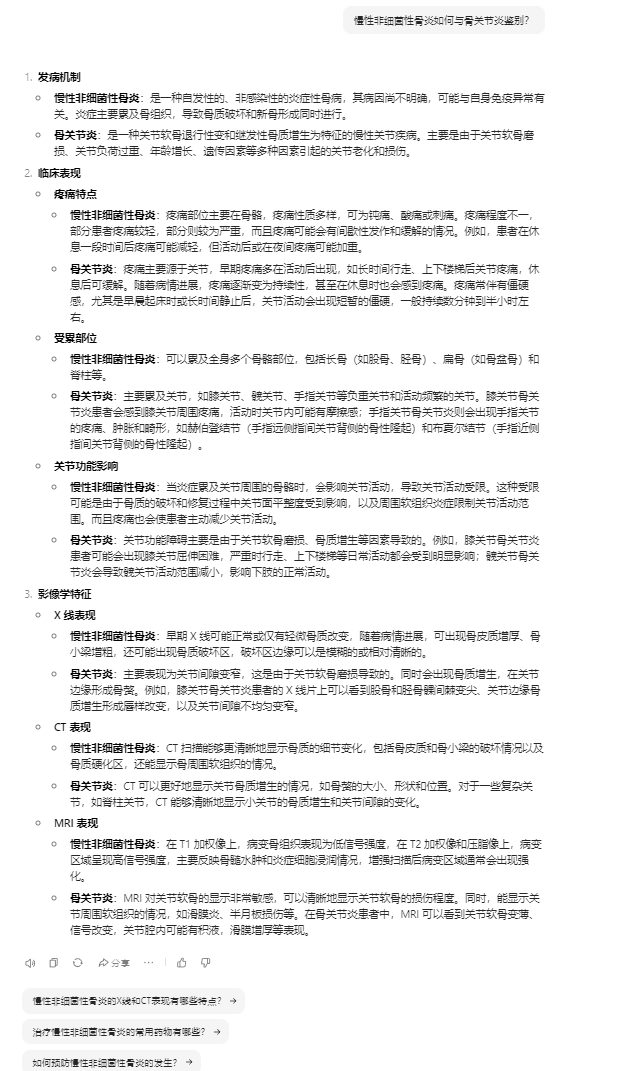
 1. **发病机制**

- **慢性非细菌性骨炎**：是一种自发性的、非感染性的炎症性骨病，其病因尚不明确，可能与自身免疫异常有关。炎症主要累及骨组织，导致骨质破坏和新骨形成同时进行。

- **骨关节炎**：是一种关节软骨退行性变和继发性骨质增生为特征的慢性关节疾病。主要是由于关节软骨磨损、关节负荷过重、年龄增长、遗传因素等多种因素引起的关节老化和损伤。

2. **临床表现**

- **疼痛特点**

- **慢性非细菌性骨炎**：疼痛部位主要在骨骼，疼痛性质多样，可为钝痛、酸痛或刺痛。疼痛程度不一，部分患者疼痛较轻，部分则较为严重，而且疼痛可能会有间歇性发作和缓解的情况。例如，患者在休息一段时间后疼痛可能减轻，但活动后或在夜间疼痛可能加重。

- **骨关节炎**：疼痛主要源于关节，早期疼痛多在活动后出现，如长时间行走、上下楼梯后关节疼痛，休息后可缓解。随着病情进展，疼痛逐渐变为持续性，甚至在休息时也会感到疼痛。疼痛常伴有僵硬感，尤其是早晨起床时或长时间静止后，关节活动会出现短暂的僵硬，一般持续数分钟到半小时左右。

- **受累部位**

- **慢性非细菌性骨炎**：可以累及全身多个骨骼部位，包括长骨（如股骨、胫骨）、扁骨（如骨盆骨）和脊柱等。

- **骨关节炎**：主要累及关节，如膝关节、髋关节、手指关节等负重关节和活动频繁的关节。膝关节骨关节炎患者会感到膝关节周围疼痛，活动时关节内可能有摩擦感；手指关节骨关节炎则会出现手指关节的疼痛、肿胀和畸形，如赫伯登结节（手指远侧指间关节背侧的骨性隆起）和布夏尔结节（手指近侧指间关节背侧的骨性隆起）。

- **关节功能影响**

- **慢性非细菌性骨炎**：当炎症累及关节周围的骨骼时，会影响关节活动，导致关节活动受限。这种受限可能是由于骨质的破坏和修复过程中关节面平整度受到影响，以及周围软组织炎症限制关节活动范围。而且疼痛也会使患者主动减少关节活动。

- **骨关节炎**：关节功能障碍主要是由于关节软骨磨损、骨质增生等因素导致的。例如，膝关节骨关节炎患者可能会出现膝关节屈伸困难，严重时行走、上下楼梯等日常活动都会受到明显影响；髋关节骨关节炎会导致髋关节活动范围减小，影响下肢的正常活动。

3. **影像学特征**

- **X线表现**

- **慢性非细菌性骨炎**：早期X线可能正常或仅有轻微骨质改变，随着病情进展，可出现骨皮质增厚、骨小梁增粗，还可能出现骨质破坏区，破坏区边缘可以是模糊的或相对清晰的。

- **骨关节炎**：主要表现为关节间隙变窄，这是由于关节软骨磨损导致的。同时会出现骨质增生，在关节边缘形成骨赘。例如，膝关节骨关节炎患者的X线片上可以看到股骨和胫骨髁间棘变尖、关节边缘骨质增生形成唇样改变，以及关节间隙不均匀变窄。

- **CT表现**

- **慢性非细菌性骨炎**：CT扫描能够更清晰地显示骨质的细节变化，包括骨皮质和骨小梁的破坏情况以及骨质硬化区，还能显示骨周围软组织的情况。

- **骨关节炎**：CT可以更好地显示关节骨质增生的情况，如骨赘的大小、形状和位置。对于一些复杂关节，如脊柱关节，CT能够清晰地显示小关节的骨质增生和关节间隙的变化。

- **MRI表现**

- **慢性非细菌性骨炎**：在T1加权像上，病变骨组织表现为低信号强度，在T2加权像和压脂像上，病变区域呈现高信号强度，主要反映骨髓水肿和炎症细胞浸润情况，增强扫描后病变区域通常会出现强化。

- **骨关节炎**：MRI对关节软骨的显示非常敏感，可以清晰地显示关节软骨的损伤程度。同时，能显示关节周围软组织的情况，如滑膜炎、半月板损伤等。在骨关节炎患者中，MRI可以看到关节软骨变薄、信号改变，关节腔内可能有积液，滑膜增厚等表现。

11慢性非细菌性骨炎如何与纤维发育不良鉴别？


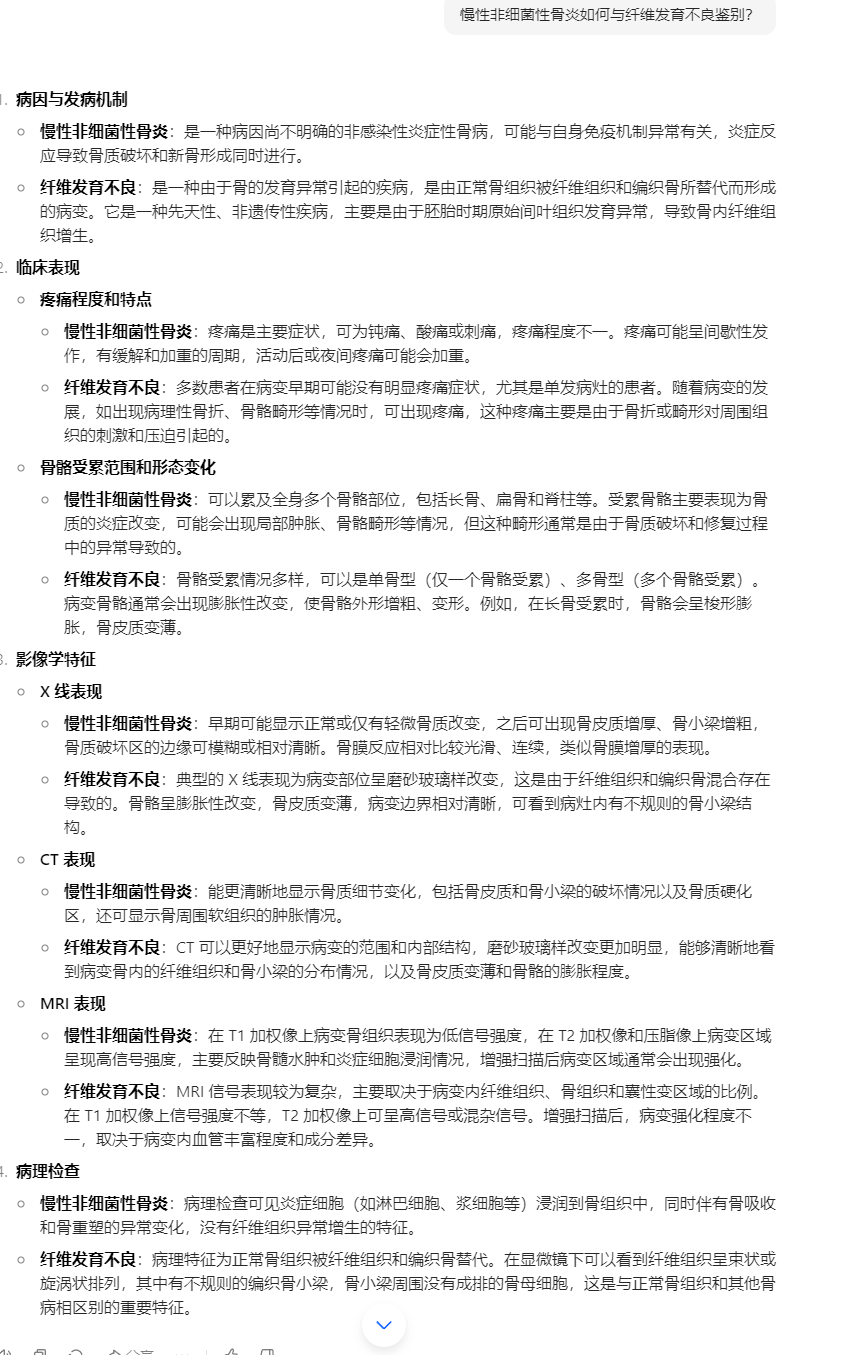
 1. **病因与发病机制**

- **慢性非细菌性骨炎**：是一种病因尚不明确的非感染性炎症性骨病，可能与自身免疫机制异常有关，炎症反应导致骨质破坏和新骨形成同时进行。

- **纤维发育不良**：是一种由于骨的发育异常引起的疾病，是由正常骨组织被纤维组织和编织骨所替代而形成的病变。它是一种先天性、非遗传性疾病，主要是由于胚胎时期原始间叶组织发育异常，导致骨内纤维组织增生。

2. **临床表现**

- **疼痛程度和特点**

- **慢性非细菌性骨炎**：疼痛是主要症状，可为钝痛、酸痛或刺痛，疼痛程度不一。疼痛可能呈间歇性发作，有缓解和加重的周期，活动后或夜间疼痛可能会加重。

- **纤维发育不良**：多数患者在病变早期可能没有明显疼痛症状，尤其是单发病灶的患者。随着病变的发展，如出现病理性骨折、骨骼畸形等情况时，可出现疼痛，这种疼痛主要是由于骨折或畸形对周围组织的刺激和压迫引起的。

- **骨骼受累范围和形态变化**

- **慢性非细菌性骨炎**：可以累及全身多个骨骼部位，包括长骨、扁骨和脊柱等。受累骨骼主要表现为骨质的炎症改变，可能会出现局部肿胀、骨骼畸形等情况，但这种畸形通常是由于骨质破坏和修复过程中的异常导致的。

- **纤维发育不良**：骨骼受累情况多样，可以是单骨型（仅一个骨骼受累）、多骨型（多个骨骼受累）。病变骨骼通常会出现膨胀性改变，使骨骼外形增粗、变形。例如，在长骨受累时，骨骼会呈梭形膨胀，骨皮质变薄。

3. **影像学特征**

- **X线表现**

- **慢性非细菌性骨炎**：早期可能显示正常或仅有轻微骨质改变，之后可出现骨皮质增厚、骨小梁增粗，骨质破坏区的边缘可模糊或相对清晰。骨膜反应相对比较光滑、连续，类似骨膜增厚的表现。

- **纤维发育不良**：典型的X线表现为病变部位呈磨砂玻璃样改变，这是由于纤维组织和编织骨混合存在导致的。骨骼呈膨胀性改变，骨皮质变薄，病变边界相对清晰，可看到病灶内有不规则的骨小梁结构。

- **CT表现**

- **慢性非细菌性骨炎**：能更清晰地显示骨质细节变化，包括骨皮质和骨小梁的破坏情况以及骨质硬化区，还可显示骨周围软组织的肿胀情况。

- **纤维发育不良**：CT可以更好地显示病变的范围和内部结构，磨砂玻璃样改变更加明显，能够清晰地看到病变骨内的纤维组织和骨小梁的分布情况，以及骨皮质变薄和骨骼的膨胀程度。

- **MRI表现**

- **慢性非细菌性骨炎**：在T1加权像上病变骨组织表现为低信号强度，在T2加权像和压脂像上病变区域呈现高信号强度，主要反映骨髓水肿和炎症细胞浸润情况，增强扫描后病变区域通常会出现强化。

- **纤维发育不良**：MRI信号表现较为复杂，主要取决于病变内纤维组织、骨组织和囊性变区域的比例。在T1加权像上信号强度不等，T2加权像上可呈高信号或混杂信号。增强扫描后，病变强化程度不一，取决于病变内血管丰富程度和成分差异。

4. **病理检查**

- **慢性非细菌性骨炎**：病理检查可见炎症细胞（如淋巴细胞、浆细胞等）浸润到骨组织中，同时伴有骨吸收和骨重塑的异常变化，没有纤维组织异常增生的特征。

- **纤维发育不良**：病理特征为正常骨组织被纤维组织和编织骨替代。在显微镜下可以看到纤维组织呈束状或旋涡状排列，其中有不规则的编织骨小梁，骨小梁周围没有成排的骨母细胞，这是与正常骨组织和其他骨病相区别的重要特征。

12在治慢性非细菌性骨炎过程中应该选择短疗程的糖皮质激素治疗还是长期的糖皮质激素治疗？


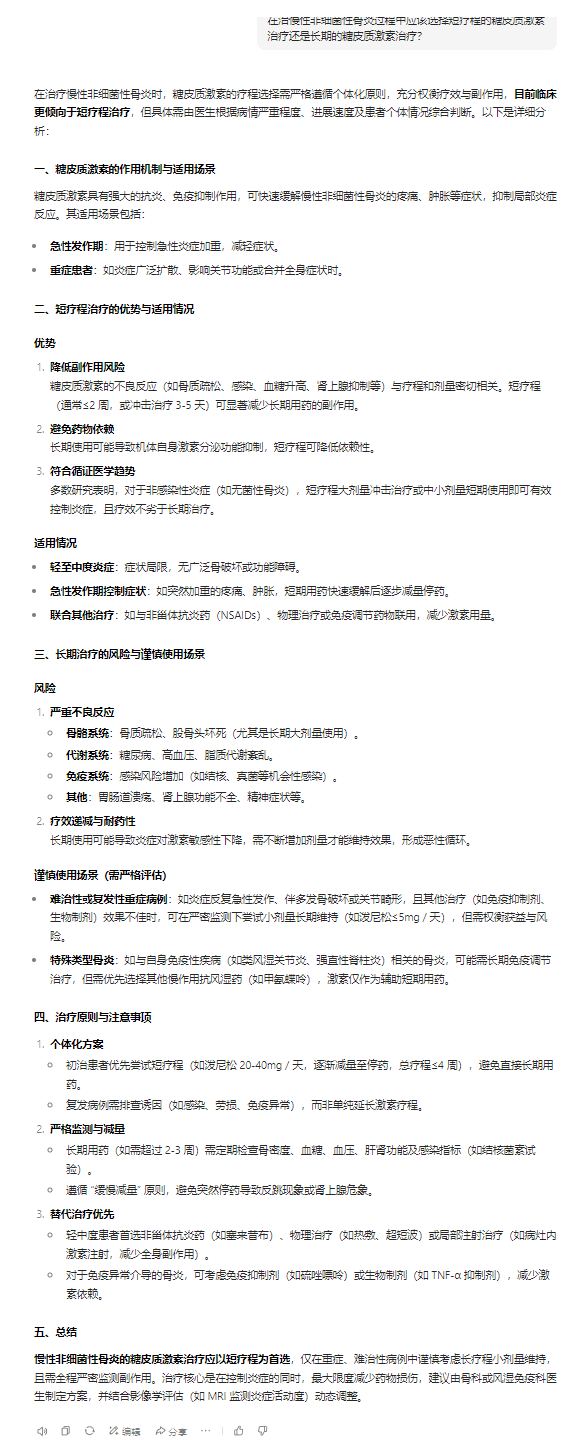


在治疗慢性非细菌性骨炎时，糖皮质激素的疗程选择需严格遵循个体化原则，充分权衡疗效与副作用，**目前临床更倾向于短疗程治疗**，但具体需由医生根据病情严重程度、进展速度及患者个体情况综合判断。以下是详细分析：

### **一、糖皮质激素的作用机制与适用场景**

糖皮质激素具有强大的抗炎、免疫抑制作用，可快速缓解慢性非细菌性骨炎的疼痛、肿胀等症状，抑制局部炎症反应。其适用场景包括：

- **急性发作期**：用于控制急性炎症加重，减轻症状。

- **重症患者**：如炎症广泛扩散、影响关节功能或合并全身症状时。

### **二、短疗程治疗的优势与适用情况**

#### **优势**

1. **降低副作用风险**

糖皮质激素的不良反应（如骨质疏松、感染、血糖升高、肾上腺抑制等）与疗程和剂量密切相关。短疗程（通常≤2周，或冲击治疗3-5天）可显著减少长期用药的副作用。

2. **避免药物依赖**

长期使用可能导致机体自身激素分泌功能抑制，短疗程可降低依赖性。

3. **符合循证医学趋势**

多数研究表明，对于非感染性炎症（如无菌性骨炎），短疗程大剂量冲击治疗或中小剂量短期使用即可有效控制炎症，且疗效不劣于长期治疗。

#### **适用情况**

- **轻至中度炎症**：症状局限，无广泛骨破坏或功能障碍。

- **急性发作期控制症状**：如突然加重的疼痛、肿胀，短期用药快速缓解后逐步减量停药。

- **联合其他治疗**：如与非甾体抗炎药（NSAIDs）、物理治疗或免疫调节药物联用，减少激素用量。

### **三、长期治疗的风险与谨慎使用场景**

#### **风险**

1. **严重不良反应**

- **骨骼系统**：骨质疏松、股骨头坏死（尤其是长期大剂量使用）。

- **代谢系统**：糖尿病、高血压、脂质代谢紊乱。

- **免疫系统**：感染风险增加（如结核、真菌等机会性感染）。

- **其他**：胃肠道溃疡、肾上腺功能不全、精神症状等。

2. **疗效递减与耐药性**

长期使用可能导致炎症对激素敏感性下降，需不断增加剂量才能维持效果，形成恶性循环。

#### **谨慎使用场景（需严格评估）**

- **难治性或复发性重症病例**：如炎症反复急性发作、伴多发骨破坏或关节畸形，且其他治疗（如免疫抑制剂、生物制剂）效果不佳时，可在严密监测下尝试小剂量长期维持（如泼尼松≤5mg/天），但需权衡获益与风险。

- **特殊类型骨炎**：如与自身免疫性疾病（如类风湿关节炎、强直性脊柱炎）相关的骨炎，可能需长期免疫调节治疗，但需优先选择其他慢作用抗风湿药（如甲氨蝶呤），激素仅作为辅助短期用药。

### **四、治疗原则与注意事项**

1. **个体化方案**

- 初治患者优先尝试短疗程（如泼尼松20-40mg/天，逐渐减量至停药，总疗程≤4周），避免直接长期用药。

- 复发病例需排查诱因（如感染、劳损、免疫异常），而非单纯延长激素疗程。

2. **严格监测与减量**

- 长期用药（如需超过2-3周）需定期检查骨密度、血糖、血压、肝肾功能及感染指标（如结核菌素试验）。

- 遵循“缓慢减量”原则，避免突然停药导致反跳现象或肾上腺危象。

3. **替代治疗优先**

- 轻中度患者首选非甾体抗炎药（如塞来昔布）、物理治疗（如热敷、超短波）或局部注射治疗（如病灶内激素注射，减少全身副作用）。

- 对于免疫异常介导的骨炎，可考虑免疫抑制剂（如硫唑嘌呤）或生物制剂（如TNF-α抑制剂），减少激素依赖。

### **五、总结**

**慢性非细菌性骨炎的糖皮质激素治疗应以短疗程为首选**，仅在重症、难治性病例中谨慎考虑长疗程小剂量维持，且需全程严密监测副作用。治疗核心是在控制炎症的同时，最大限度减少药物损伤，建议由骨科或风湿免疫科医生制定方案，并结合影像学评估（如MRI监测炎症活动度）动态调整。

13成人慢性非细菌性骨炎一线治疗方案是什么？


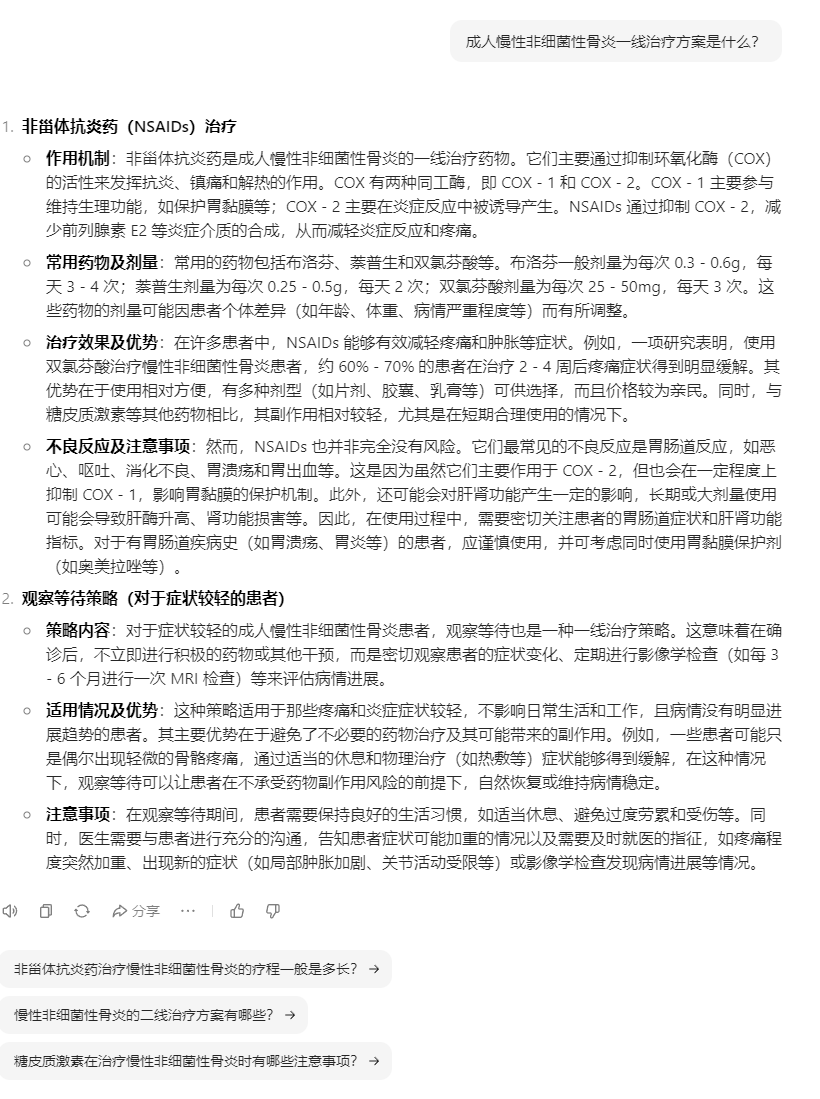
 1. **非甾体抗炎药（NSAIDs）治疗**

- **作用机制**：非甾体抗炎药是成人慢性非细菌性骨炎的一线治疗药物。它们主要通过抑制环氧化酶（COX）的活性来发挥抗炎、镇痛和解热的作用。COX有两种同工酶，即COX - 1和COX - 2。COX - 1主要参与维持生理功能，如保护胃黏膜等；COX - 2主要在炎症反应中被诱导产生。NSAIDs通过抑制COX - 2，减少前列腺素E2等炎症介质的合成，从而减轻炎症反应和疼痛。

- **常用药物及剂量**：常用的药物包括布洛芬、萘普生和双氯芬酸等。布洛芬一般剂量为每次0.3 - 0.6g，每天3 - 4次；萘普生剂量为每次0.25 - 0.5g，每天2次；双氯芬酸剂量为每次25 - 50mg，每天3次。这些药物的剂量可能因患者个体差异（如年龄、体重、病情严重程度等）而有所调整。

- **治疗效果及优势**：在许多患者中，NSAIDs能够有效减轻疼痛和肿胀等症状。例如，一项研究表明，使用双氯芬酸治疗慢性非细菌性骨炎患者，约60% - 70%的患者在治疗2 - 4周后疼痛症状得到明显缓解。其优势在于使用相对方便，有多种剂型（如片剂、胶囊、乳膏等）可供选择，而且价格较为亲民。同时，与糖皮质激素等其他药物相比，其副作用相对较轻，尤其是在短期合理使用的情况下。

- **不良反应及注意事项**：然而，NSAIDs也并非完全没有风险。它们最常见的不良反应是胃肠道反应，如恶心、呕吐、消化不良、胃溃疡和胃出血等。这是因为虽然它们主要作用于COX - 2，但也会在一定程度上抑制COX - 1，影响胃黏膜的保护机制。此外，还可能会对肝肾功能产生一定的影响，长期或大剂量使用可能会导致肝酶升高、肾功能损害等。因此，在使用过程中，需要密切关注患者的胃肠道症状和肝肾功能指标。对于有胃肠道疾病史（如胃溃疡、胃炎等）的患者，应谨慎使用，并可考虑同时使用胃黏膜保护剂（如奥美拉唑等）。

2. **观察等待策略（对于症状较轻的患者）**

- **策略内容**：对于症状较轻的成人慢性非细菌性骨炎患者，观察等待也是一种一线治疗策略。这意味着在确诊后，不立即进行积极的药物或其他干预，而是密切观察患者的症状变化、定期进行影像学检查（如每3 - 6个月进行一次MRI检查）等来评估病情进展。

- **适用情况及优势**：这种策略适用于那些疼痛和炎症症状较轻，不影响日常生活和工作，且病情没有明显进展趋势的患者。其主要优势在于避免了不必要的药物治疗及其可能带来的副作用。例如，一些患者可能只是偶尔出现轻微的骨骼疼痛，通过适当的休息和物理治疗（如热敷等）症状能够得到缓解，在这种情况下，观察等待可以让患者在不承受药物副作用风险的前提下，自然恢复或维持病情稳定。

- **注意事项**：在观察等待期间，患者需要保持良好的生活习惯，如适当休息、避免过度劳累和受伤等。同时，医生需要与患者进行充分的沟通，告知患者症状可能加重的情况以及需要及时就医的指征，如疼痛程度突然加重、出现新的症状（如局部肿胀加剧、关节活动受限等）或影像学检查发现病情进展等情况。

14无临床症状，也没有放射学疾病活动的慢性非细菌性骨炎的病人需要治疗吗？


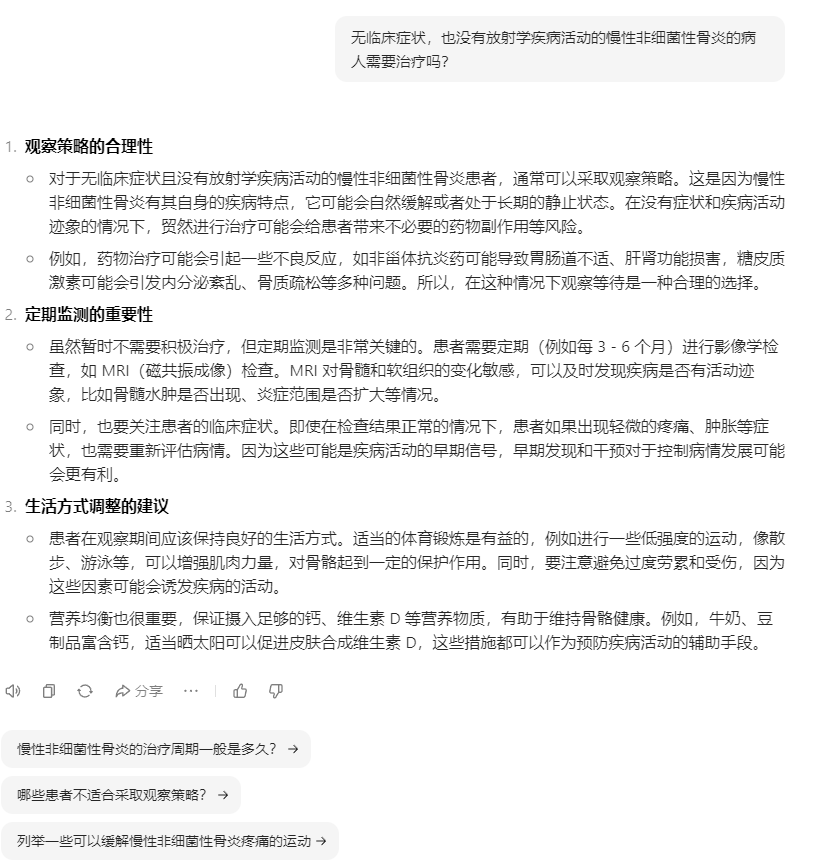
 1. **观察策略的合理性**

- 对于无临床症状且没有放射学疾病活动的慢性非细菌性骨炎患者，通常可以采取观察策略。这是因为慢性非细菌性骨炎有其自身的疾病特点，它可能会自然缓解或者处于长期的静止状态。在没有症状和疾病活动迹象的情况下，贸然进行治疗可能会给患者带来不必要的药物副作用等风险。

- 例如，药物治疗可能会引起一些不良反应，如非甾体抗炎药可能导致胃肠道不适、肝肾功能损害，糖皮质激素可能会引发内分泌紊乱、骨质疏松等多种问题。所以，在这种情况下观察等待是一种合理的选择。

2. **定期监测的重要性**

- 虽然暂时不需要积极治疗，但定期监测是非常关键的。患者需要定期（例如每3 - 6个月）进行影像学检查，如MRI（磁共振成像）检查。MRI对骨髓和软组织的变化敏感，可以及时发现疾病是否有活动迹象，比如骨髓水肿是否出现、炎症范围是否扩大等情况。

- 同时，也要关注患者的临床症状。即使在检查结果正常的情况下，患者如果出现轻微的疼痛、肿胀等症状，也需要重新评估病情。因为这些可能是疾病活动的早期信号，早期发现和干预对于控制病情发展可能会更有利。

3. **生活方式调整的建议**

- 患者在观察期间应该保持良好的生活方式。适当的体育锻炼是有益的，例如进行一些低强度的运动，像散步、游泳等，可以增强肌肉力量，对骨骼起到一定的保护作用。同时，要注意避免过度劳累和受伤，因为这些因素可能会诱发疾病的活动。

- 营养均衡也很重要，保证摄入足够的钙、维生素D等营养物质，有助于维持骨骼健康。例如，牛奶、豆制品富含钙，适当晒太阳可以促进皮肤合成维生素D，这些措施都可以作为预防疾病活动的辅助手段。

15有临床症状没有放射学疾病活动的慢性非细菌性骨炎的病人如何处理？


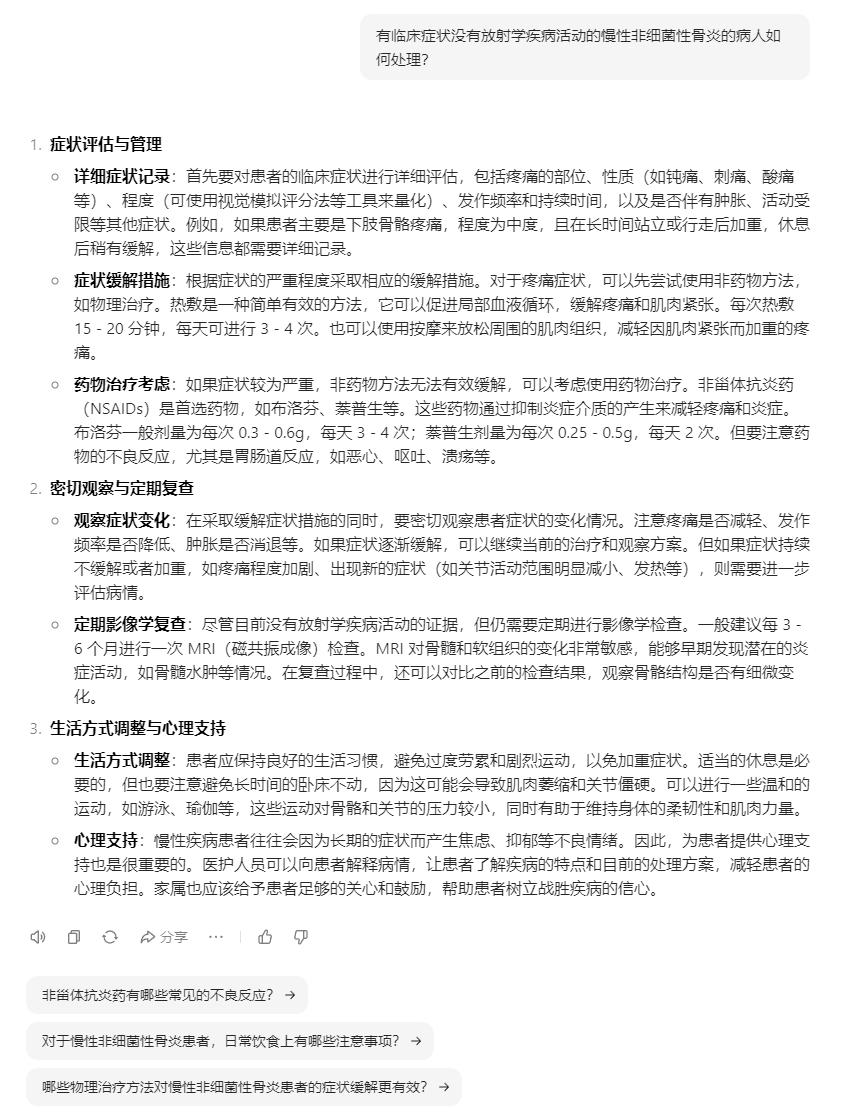
 1. **症状评估与管理**

- **详细症状记录**：首先要对患者的临床症状进行详细评估，包括疼痛的部位、性质（如钝痛、刺痛、酸痛等）、程度（可使用视觉模拟评分法等工具来量化）、发作频率和持续时间，以及是否伴有肿胀、活动受限等其他症状。例如，如果患者主要是下肢骨骼疼痛，程度为中度，且在长时间站立或行走后加重，休息后稍有缓解，这些信息都需要详细记录。

- **症状缓解措施**：根据症状的严重程度采取相应的缓解措施。对于疼痛症状，可以先尝试使用非药物方法，如物理治疗。热敷是一种简单有效的方法，它可以促进局部血液循环，缓解疼痛和肌肉紧张。每次热敷15 - 20分钟，每天可进行3 - 4次。也可以使用按摩来放松周围的肌肉组织，减轻因肌肉紧张而加重的疼痛。

- **药物治疗考虑**：如果症状较为严重，非药物方法无法有效缓解，可以考虑使用药物治疗。非甾体抗炎药（NSAIDs）是首选药物，如布洛芬、萘普生等。这些药物通过抑制炎症介质的产生来减轻疼痛和炎症。布洛芬一般剂量为每次0.3 - 0.6g，每天3 - 4次；萘普生剂量为每次0.25 - 0.5g，每天2次。但要注意药物的不良反应，尤其是胃肠道反应，如恶心、呕吐、溃疡等。

2. **密切观察与定期复查**

- **观察症状变化**：在采取缓解症状措施的同时，要密切观察患者症状的变化情况。注意疼痛是否减轻、发作频率是否降低、肿胀是否消退等。如果症状逐渐缓解，可以继续当前的治疗和观察方案。但如果症状持续不缓解或者加重，如疼痛程度加剧、出现新的症状（如关节活动范围明显减小、发热等），则需要进一步评估病情。

- **定期影像学复查**：尽管目前没有放射学疾病活动的证据，但仍需要定期进行影像学检查。一般建议每3 - 6个月进行一次MRI（磁共振成像）检查。MRI对骨髓和软组织的变化非常敏感，能够早期发现潜在的炎症活动，如骨髓水肿等情况。在复查过程中，还可以对比之前的检查结果，观察骨骼结构是否有细微变化。

3. **生活方式调整与心理支持**

- **生活方式调整**：患者应保持良好的生活习惯，避免过度劳累和剧烈运动，以免加重症状。适当的休息是必要的，但也要注意避免长时间的卧床不动，因为这可能会导致肌肉萎缩和关节僵硬。可以进行一些温和的运动，如游泳、瑜伽等，这些运动对骨骼和关节的压力较小，同时有助于维持身体的柔韧性和肌肉力量。

- **心理支持**：慢性疾病患者往往会因为长期的症状而产生焦虑、抑郁等不良情绪。因此，为患者提供心理支持也是很重要的。医护人员可以向患者解释病情，让患者了解疾病的特点和目前的处理方案，减轻患者的心理负担。家属也应该给予患者足够的关心和鼓励，帮助患者树立战胜疾病的信心。

16有椎体塌陷风险的脊柱骨病变的慢性非细菌性骨炎患者和出现明显骨骼累积损伤的慢性非细菌性骨炎患者如何治疗？


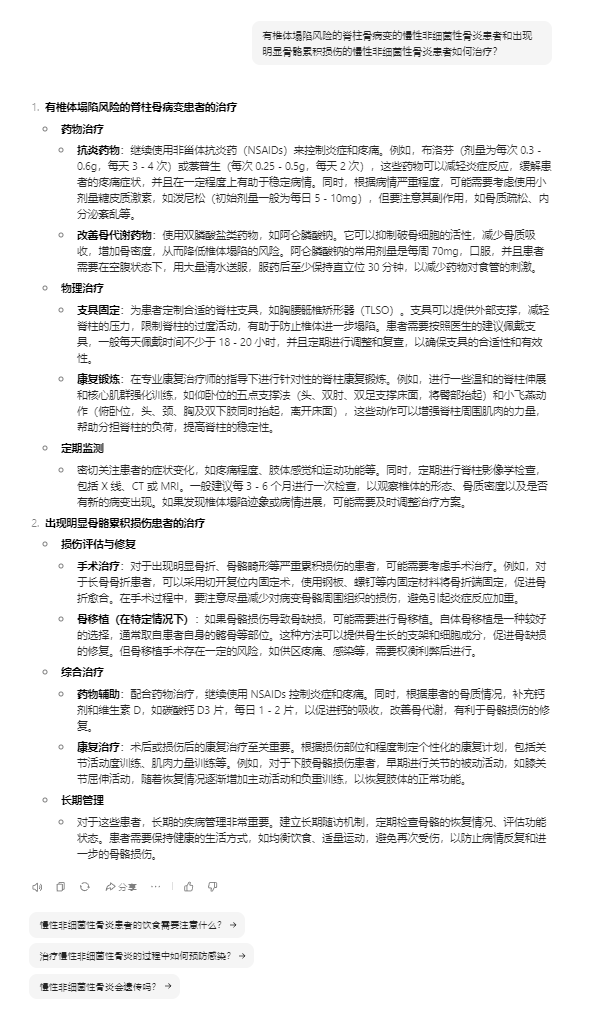
 1. **有椎体塌陷风险的脊柱骨病变患者的治疗**

- **药物治疗**

- **抗炎药物**：继续使用非甾体抗炎药（NSAIDs）来控制炎症和疼痛。例如，布洛芬（剂量为每次0.3 - 0.6g，每天3 - 4次）或萘普生（每次0.25 - 0.5g，每天2次），这些药物可以减轻炎症反应，缓解患者的疼痛症状，并且在一定程度上有助于稳定病情。同时，根据病情严重程度，可能需要考虑使用小剂量糖皮质激素，如泼尼松（初始剂量一般为每日5 - 10mg），但要注意其副作用，如骨质疏松、内分泌紊乱等。

- **改善骨代谢药物**：使用双膦酸盐类药物，如阿仑膦酸钠。它可以抑制破骨细胞的活性，减少骨质吸收，增加骨密度，从而降低椎体塌陷的风险。阿仑膦酸钠的常用剂量是每周70mg，口服，并且患者需要在空腹状态下，用大量清水送服，服药后至少保持直立位30分钟，以减少药物对食管的刺激。

- **物理治疗**

- **支具固定**：为患者定制合适的脊柱支具，如胸腰骶椎矫形器（TLSO）。支具可以提供外部支撑，减轻脊柱的压力，限制脊柱的过度活动，有助于防止椎体进一步塌陷。患者需要按照医生的建议佩戴支具，一般每天佩戴时间不少于18 - 20小时，并且定期进行调整和复查，以确保支具的合适性和有效性。

- **康复锻炼**：在专业康复治疗师的指导下进行针对性的脊柱康复锻炼。例如，进行一些温和的脊柱伸展和核心肌群强化训练，如仰卧位的五点支撑法（头、双肘、双足支撑床面，将臀部抬起）和小飞燕动作（俯卧位，头、颈、胸及双下肢同时抬起，离开床面），这些动作可以增强脊柱周围肌肉的力量，帮助分担脊柱的负荷，提高脊柱的稳定性。

- **定期监测**

- 密切关注患者的症状变化，如疼痛程度、肢体感觉和运动功能等。同时，定期进行脊柱影像学检查，包括X线、CT或MRI。一般建议每3 - 6个月进行一次检查，以观察椎体的形态、骨质密度以及是否有新的病变出现。如果发现椎体塌陷迹象或病情进展，可能需要及时调整治疗方案。

2. **出现明显骨骼累积损伤患者的治疗**

- **损伤评估与修复**

- **手术治疗**：对于出现明显骨折、骨骼畸形等严重累积损伤的患者，可能需要考虑手术治疗。例如，对于长骨骨折患者，可以采用切开复位内固定术，使用钢板、螺钉等内固定材料将骨折端固定，促进骨折愈合。在手术过程中，要注意尽量减少对病变骨骼周围组织的损伤，避免引起炎症反应加重。

- **骨移植（在特定情况下）**：如果骨骼损伤导致骨缺损，可能需要进行骨移植。自体骨移植是一种较好的选择，通常取自患者自身的髂骨等部位。这种方法可以提供骨生长的支架和细胞成分，促进骨缺损的修复。但骨移植手术存在一定的风险，如供区疼痛、感染等，需要权衡利弊后进行。

- **综合治疗**

- **药物辅助**：配合药物治疗，继续使用NSAIDs控制炎症和疼痛。同时，根据患者的骨质情况，补充钙剂和维生素D，如碳酸钙D3片，每日1 - 2片，以促进钙的吸收，改善骨代谢，有利于骨骼损伤的修复。

- **康复治疗**：术后或损伤后的康复治疗至关重要。根据损伤部位和程度制定个性化的康复计划，包括关节活动度训练、肌肉力量训练等。例如，对于下肢骨骼损伤患者，早期进行关节的被动活动，如膝关节屈伸活动，随着恢复情况逐渐增加主动活动和负重训练，以恢复肢体的正常功能。

- **长期管理**

- 对于这些患者，长期的疾病管理非常重要。建立长期随访机制，定期检查骨骼的恢复情况、评估功能状态。患者需要保持健康的生活方式，如均衡饮食、适量运动，避免再次受伤，以防止病情反复和进一步的骨骼损伤。
