# Supplementary material for: Evaluation of the accuracy and repeatability of Deepseek V3, Doubao, and Kimi1.5 in answering knowledge-related queries about chronic non-bacterial osteitis
Source: Front Artif Intell. 2025 Sep 29;8:1629149. doi: 10.3389/frai.2025.1629149 (PMC12515971; doi:10.3389/frai.2025.1629149)
Supplement: Supplementary file 1 [file Data_Sheet_1.zip › day28/day28-Kimi.docx]

1成人慢性非细菌性骨炎的定义是什么？


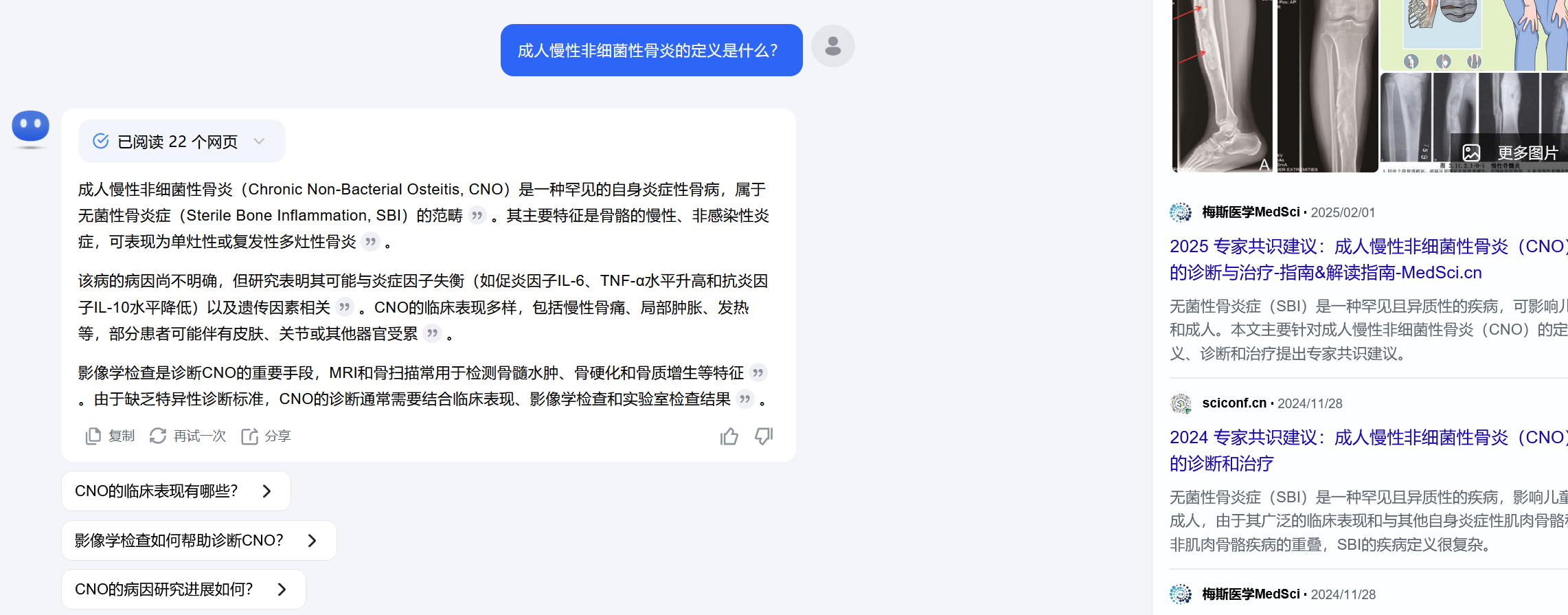


成人慢性非细菌性骨炎（Chronic Non-Bacterial Osteitis, CNO）是一种罕见的自身炎症性骨病，属于无菌性骨炎症（Sterile Bone Inflammation, SBI）的范畴。其主要特征是骨骼的慢性、非感染性炎症，可表现为单灶性或复发性多灶性骨炎。

该病的病因尚不明确，但研究表明其可能与炎症因子失衡（如促炎因子IL-6、TNF-α水平升高和抗炎因子IL-10水平降低）以及遗传因素相关。CNO的临床表现多样，包括慢性骨痛、局部肿胀、发热等，部分患者可能伴有皮肤、关节或其他器官受累。

影像学检查是诊断CNO的重要手段，MRI和骨扫描常用于检测骨髓水肿、骨硬化和骨质增生等特征。由于缺乏特异性诊断标准，CNO的诊断通常需要结合临床表现、影像学检查和实验室检查结果。

2成人慢性非细菌性骨炎的典型影像学特征是什么？


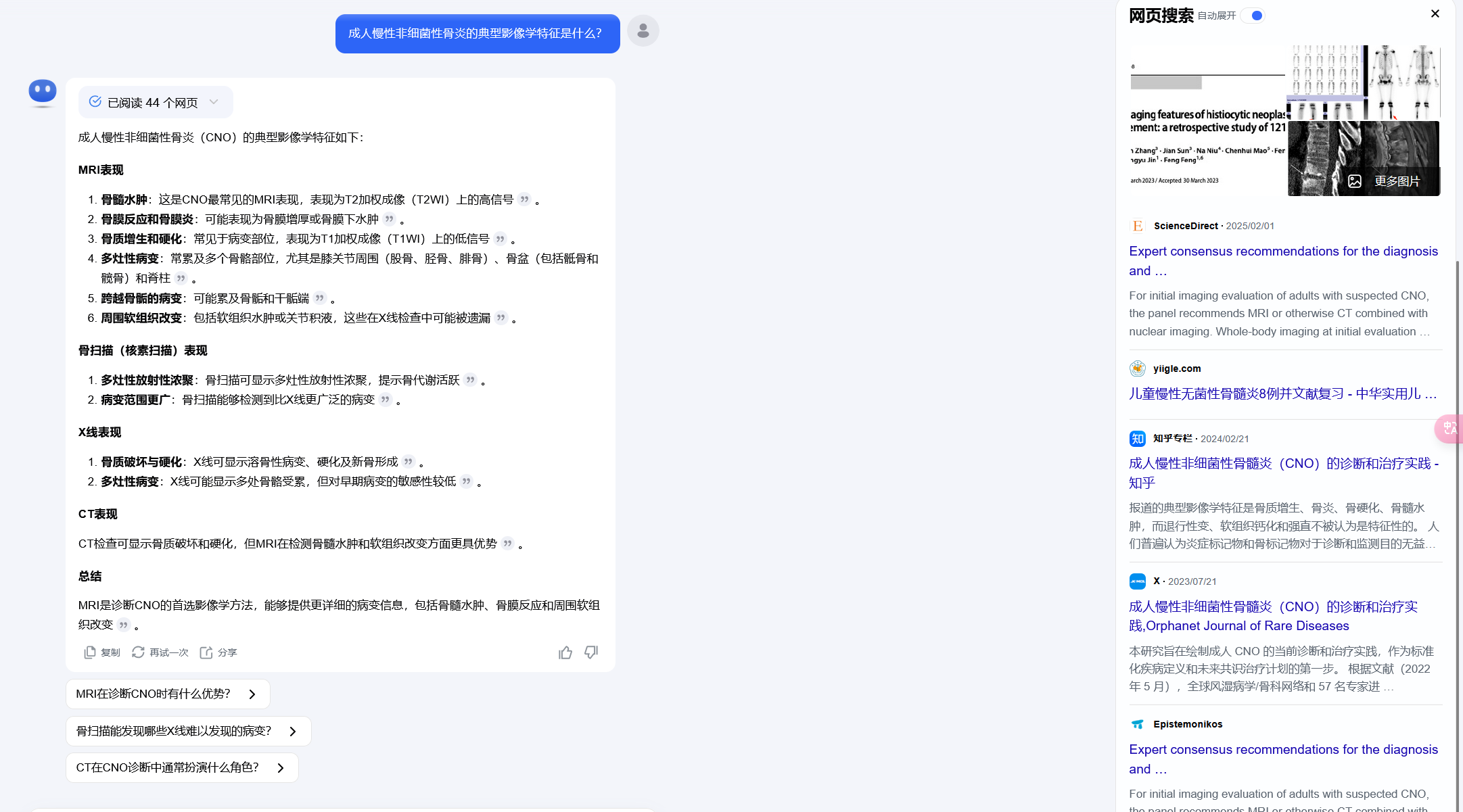


成人慢性非细菌性骨炎（CNO）的典型影像学特征如下：

### MRI表现

1. **骨髓水肿**：这是CNO最常见的MRI表现，表现为T2加权成像（T2WI）上的高信号。

2. **骨膜反应和骨膜炎**：可能表现为骨膜增厚或骨膜下水肿。

3. **骨质增生和硬化**：常见于病变部位，表现为T1加权成像（T1WI）上的低信号。

4. **多灶性病变**：常累及多个骨骼部位，尤其是膝关节周围（股骨、胫骨、腓骨）、骨盆（包括骶骨和髋骨）和脊柱。

5. **跨越骨骺的病变**：可能累及骨骺和干骺端。

6. **周围软组织改变**：包括软组织水肿或关节积液，这些在X线检查中可能被遗漏。

### 骨扫描（核素扫描）表现

1. **多灶性放射性浓聚**：骨扫描可显示多灶性放射性浓聚，提示骨代谢活跃。

2. **病变范围更广**：骨扫描能够检测到比X线更广泛的病变。

### X线表现

1. **骨质破坏与硬化**：X线可显示溶骨性病变、硬化及新骨形成。

2. **多灶性病变**：X线可能显示多处骨骼受累，但对早期病变的敏感性较低。

### CT表现

CT检查可显示骨质破坏和硬化，但MRI在检测骨髓水肿和软组织改变方面更具优势。

### 总结

MRI是诊断CNO的首选影像学方法，能够提供更详细的病变信息，包括骨髓水肿、骨膜反应和周围软组织改变。

3成人慢性非细菌性骨炎最常见的表现是什么？


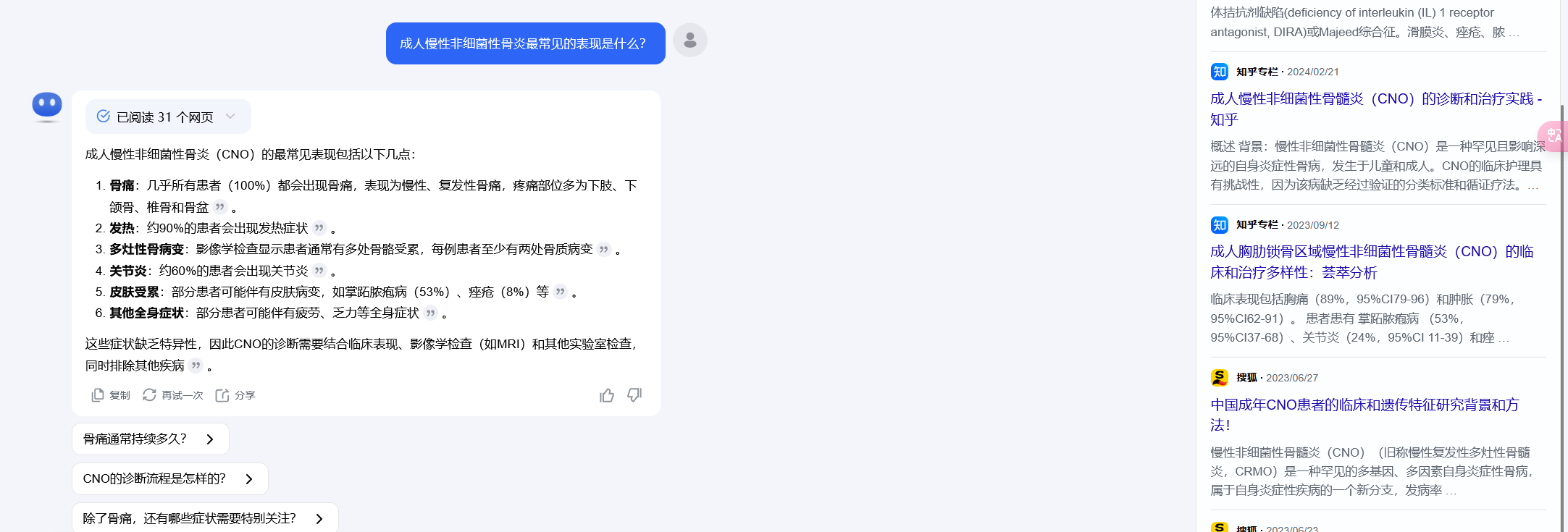


成人慢性非细菌性骨炎（CNO）的最常见表现包括以下几点：

1. **骨痛**：几乎所有患者（100%）都会出现骨痛，表现为慢性、复发性骨痛，疼痛部位多为下肢、下颌骨、椎骨和骨盆。

2. **发热**：约90%的患者会出现发热症状。

3. **多灶性骨病变**：影像学检查显示患者通常有多处骨骼受累，每例患者至少有两处骨质病变。

4. **关节炎**：约60%的患者会出现关节炎。

5. **皮肤受累**：部分患者可能伴有皮肤病变，如掌跖脓疱病（53%）、痤疮（8%）等。

6. **其他全身症状**：部分患者可能伴有疲劳、乏力等全身症状。

这些症状缺乏特异性，因此CNO的诊断需要结合临床表现、影像学检查（如MRI）和其他实验室检查，同时排除其他疾病。

4成人慢性非细菌性骨炎最常受累的部位是那些？


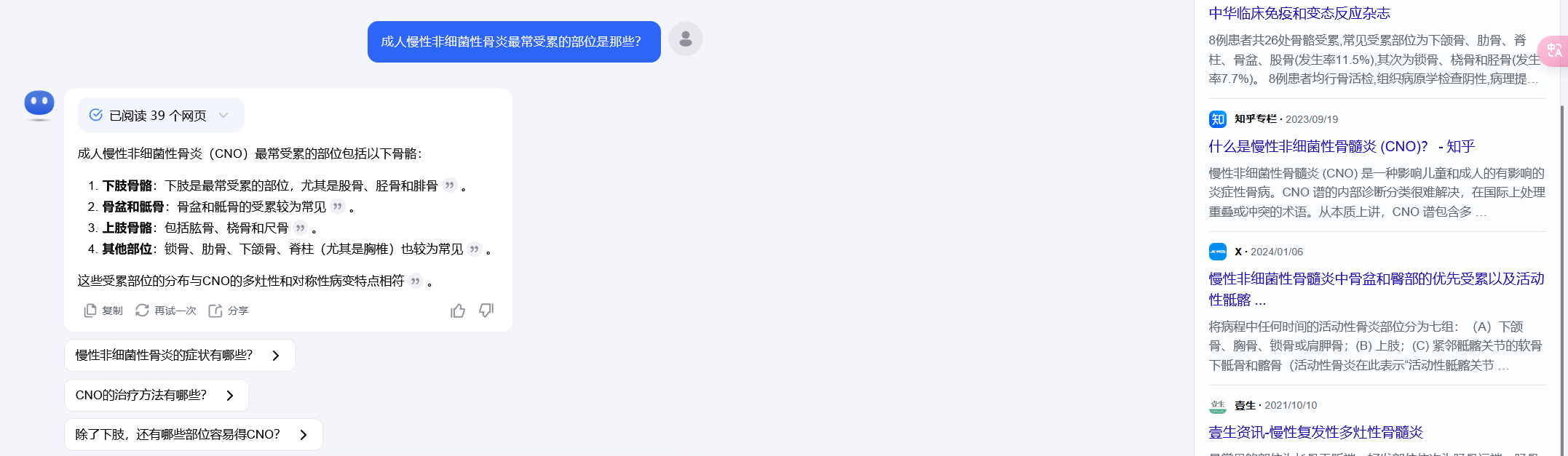


成人慢性非细菌性骨炎（CNO）最常受累的部位包括以下骨骼：

1. **下肢骨骼**：下肢是最常受累的部位，尤其是股骨、胫骨和腓骨。

2. **骨盆和骶骨**：骨盆和骶骨的受累较为常见。

3. **上肢骨骼**：包括肱骨、桡骨和尺骨。

4. **其他部位**：锁骨、肋骨、下颌骨、脊柱（尤其是胸椎）也较为常见。

这些受累部位的分布与CNO的多灶性和对称性病变特点相符。

5成人慢性非细菌性骨炎有没有特异性的炎症标志物？


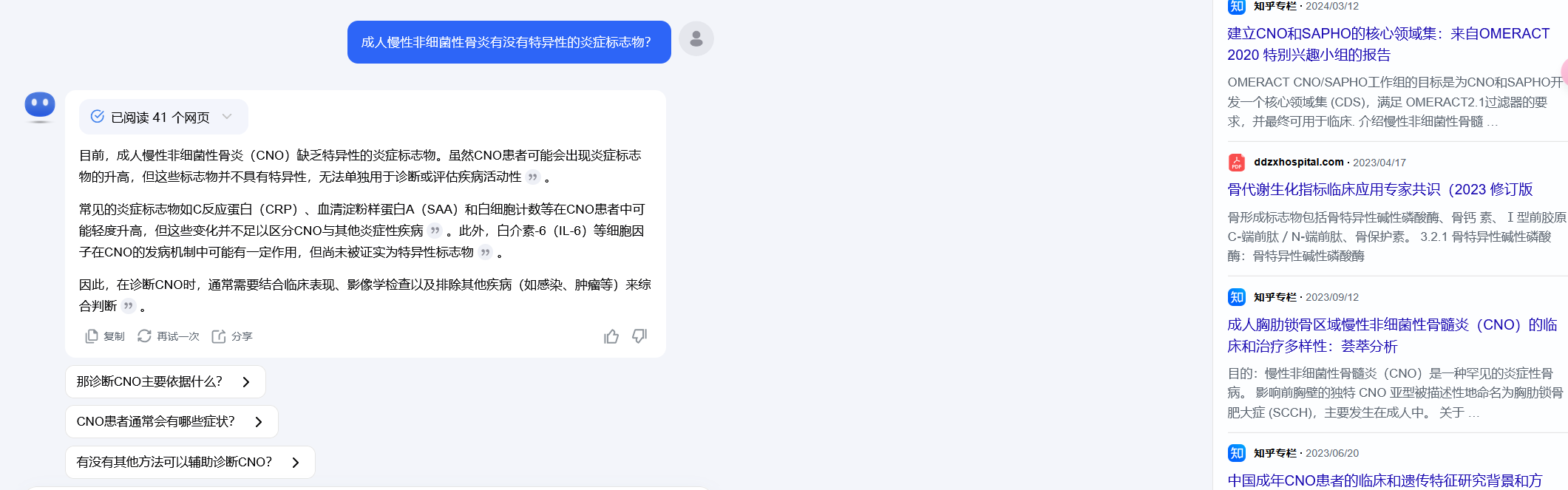


目前，成人慢性非细菌性骨炎（CNO）缺乏特异性的炎症标志物。虽然CNO患者可能会出现炎症标志物的升高，但这些标志物并不具有特异性，无法单独用于诊断或评估疾病活动性。

常见的炎症标志物如C反应蛋白（CRP）、血清淀粉样蛋白A（SAA）和白细胞计数等在CNO患者中可能轻度升高，但这些变化并不足以区分CNO与其他炎症性疾病。此外，白介素-6（IL-6）等细胞因子在CNO的发病机制中可能有一定作用，但尚未被证实为特异性标志物。

因此，在诊断CNO时，通常需要结合临床表现、影像学检查以及排除其他疾病（如感染、肿瘤等）来综合判断。

6慢性非细菌性骨炎首选的影像学检查是什么？


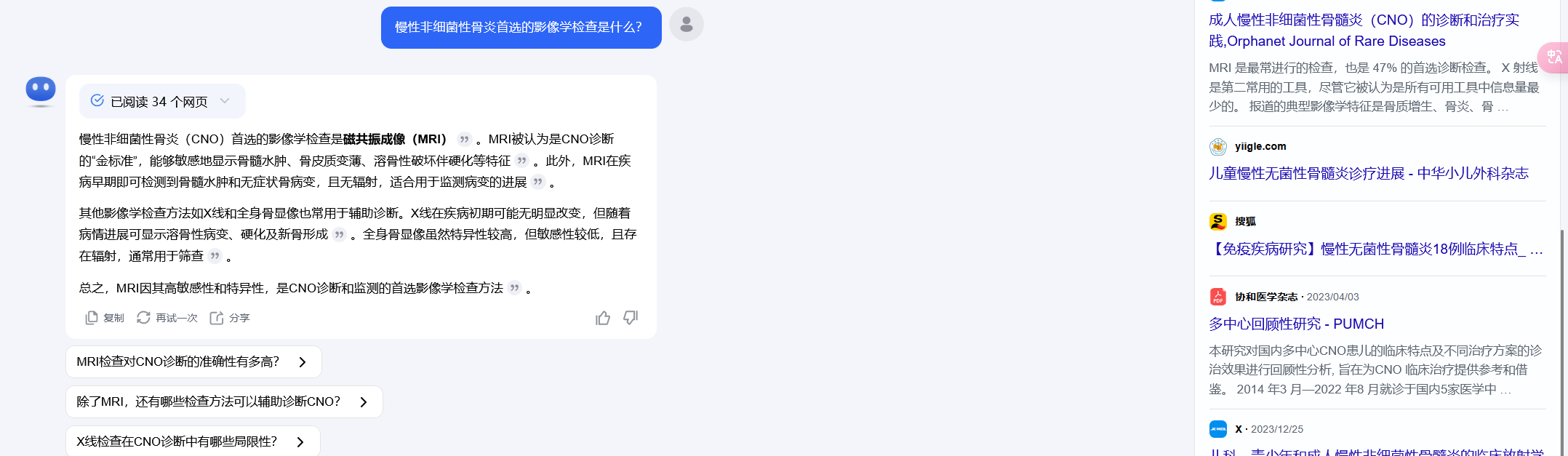


慢性非细菌性骨炎（CNO）首选的影像学检查是**磁共振成像（MRI）**。MRI被认为是CNO诊断的“金标准”，能够敏感地显示骨髓水肿、骨皮质变薄、溶骨性破坏伴硬化等特征。此外，MRI在疾病早期即可检测到骨髓水肿和无症状骨病变，且无辐射，适合用于监测病变的进展。

其他影像学检查方法如X线和全身骨显像也常用于辅助诊断。X线在疾病初期可能无明显改变，但随着病情进展可显示溶骨性病变、硬化及新骨形成。全身骨显像虽然特异性较高，但敏感性较低，且存在辐射，通常用于筛查。

总之，MRI因其高敏感性和特异性，是CNO诊断和监测的首选影像学检查方法。

7全身成像检查是不是诊断成人慢性非细菌性骨炎的先决条件？


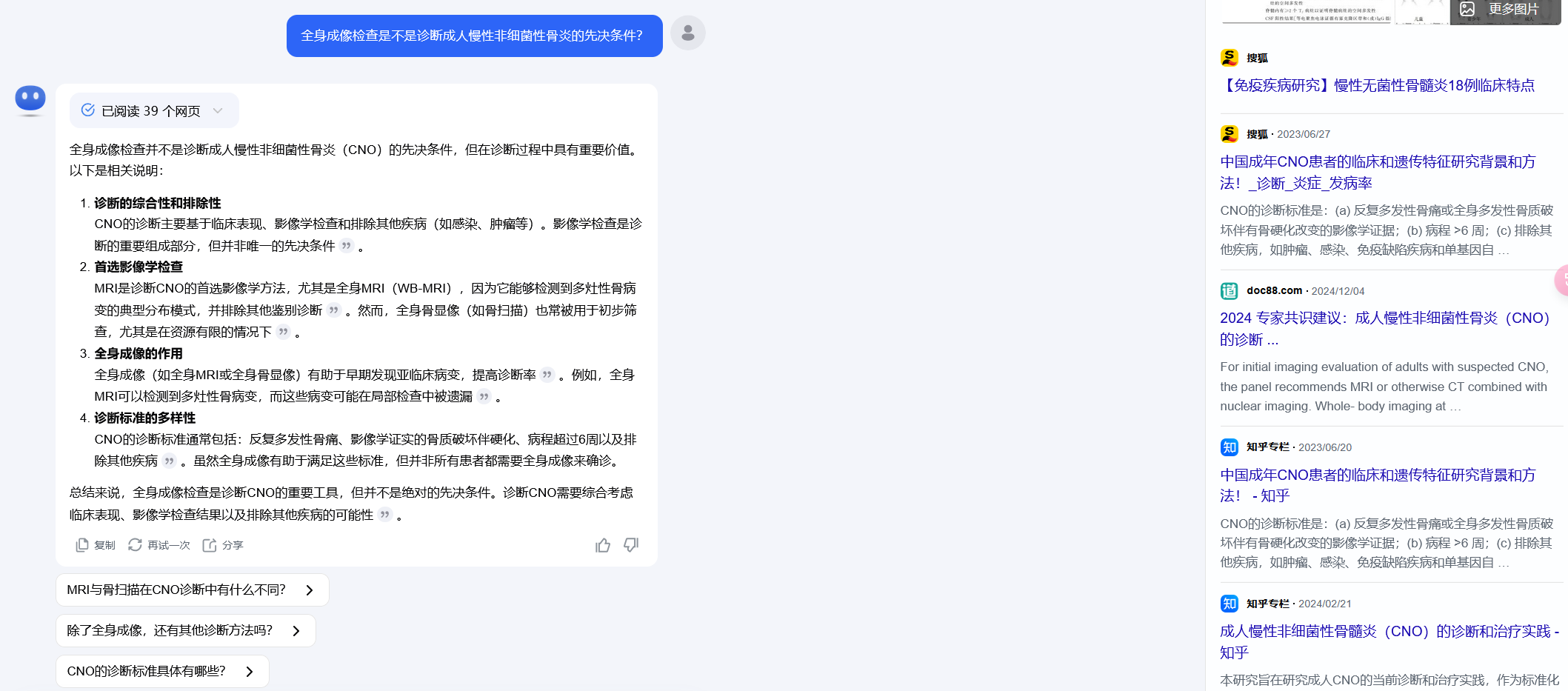


全身成像检查并不是诊断成人慢性非细菌性骨炎（CNO）的先决条件，但在诊断过程中具有重要价值。以下是相关说明：

1. **诊断的综合性和排除性**

CNO的诊断主要基于临床表现、影像学检查和排除其他疾病（如感染、肿瘤等）。影像学检查是诊断的重要组成部分，但并非唯一的先决条件。

2. **首选影像学检查**

MRI是诊断CNO的首选影像学方法，尤其是全身MRI（WB-MRI），因为它能够检测到多灶性骨病变的典型分布模式，并排除其他鉴别诊断。然而，全身骨显像（如骨扫描）也常被用于初步筛查，尤其是在资源有限的情况下。

3. **全身成像的作用**

全身成像（如全身MRI或全身骨显像）有助于早期发现亚临床病变，提高诊断率。例如，全身MRI可以检测到多灶性骨病变，而这些病变可能在局部检查中被遗漏。

4. **诊断标准的多样性**

CNO的诊断标准通常包括：反复多发性骨痛、影像学证实的骨质破坏伴硬化、病程超过6周以及排除其他疾病。虽然全身成像有助于满足这些标准，但并非所有患者都需要全身成像来确诊。

总结来说，全身成像检查是诊断CNO的重要工具，但并不是绝对的先决条件。诊断CNO需要综合考虑临床表现、影像学检查结果以及排除其他疾病的可能性。

8诊断成人慢性非细菌性骨炎有必要常规骨活检吗？


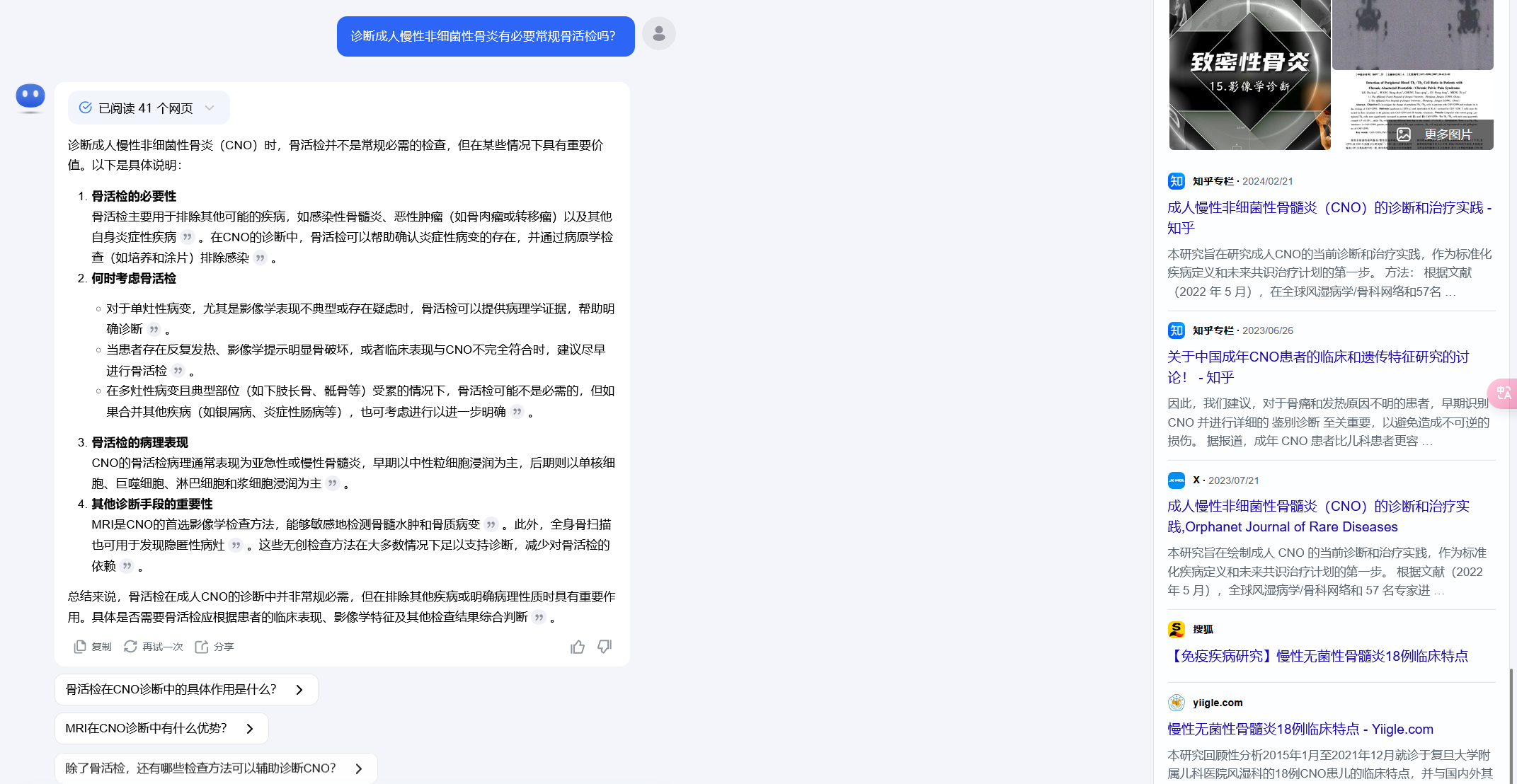


诊断成人慢性非细菌性骨炎（CNO）时，骨活检并不是常规必需的检查，但在某些情况下具有重要价值。以下是具体说明：

1. **骨活检的必要性**

骨活检主要用于排除其他可能的疾病，如感染性骨髓炎、恶性肿瘤（如骨肉瘤或转移瘤）以及其他自身炎症性疾病。在CNO的诊断中，骨活检可以帮助确认炎症性病变的存在，并通过病原学检查（如培养和涂片）排除感染。

2. **何时考虑骨活检**

- 对于单灶性病变，尤其是影像学表现不典型或存在疑虑时，骨活检可以提供病理学证据，帮助明确诊断。

- 当患者存在反复发热、影像学提示明显骨破坏，或者临床表现与CNO不完全符合时，建议尽早进行骨活检。

- 在多灶性病变且典型部位（如下肢长骨、骶骨等）受累的情况下，骨活检可能不是必需的，但如果合并其他疾病（如银屑病、炎症性肠病等），也可考虑进行以进一步明确。

3. **骨活检的病理表现**

CNO的骨活检病理通常表现为亚急性或慢性骨髓炎，早期以中性粒细胞浸润为主，后期则以单核细胞、巨噬细胞、淋巴细胞和浆细胞浸润为主。

4. **其他诊断手段的重要性**

MRI是CNO的首选影像学检查方法，能够敏感地检测骨髓水肿和骨质病变。此外，全身骨扫描也可用于发现隐匿性病灶。这些无创检查方法在大多数情况下足以支持诊断，减少对骨活检的依赖。

总结来说，骨活检在成人CNO的诊断中并非常规必需，但在排除其他疾病或明确病理性质时具有重要作用。具体是否需要骨活检应根据患者的临床表现、影像学特征及其他检查结果综合判断。

9慢性非细菌性骨炎如何与恶性骨肿瘤鉴别？


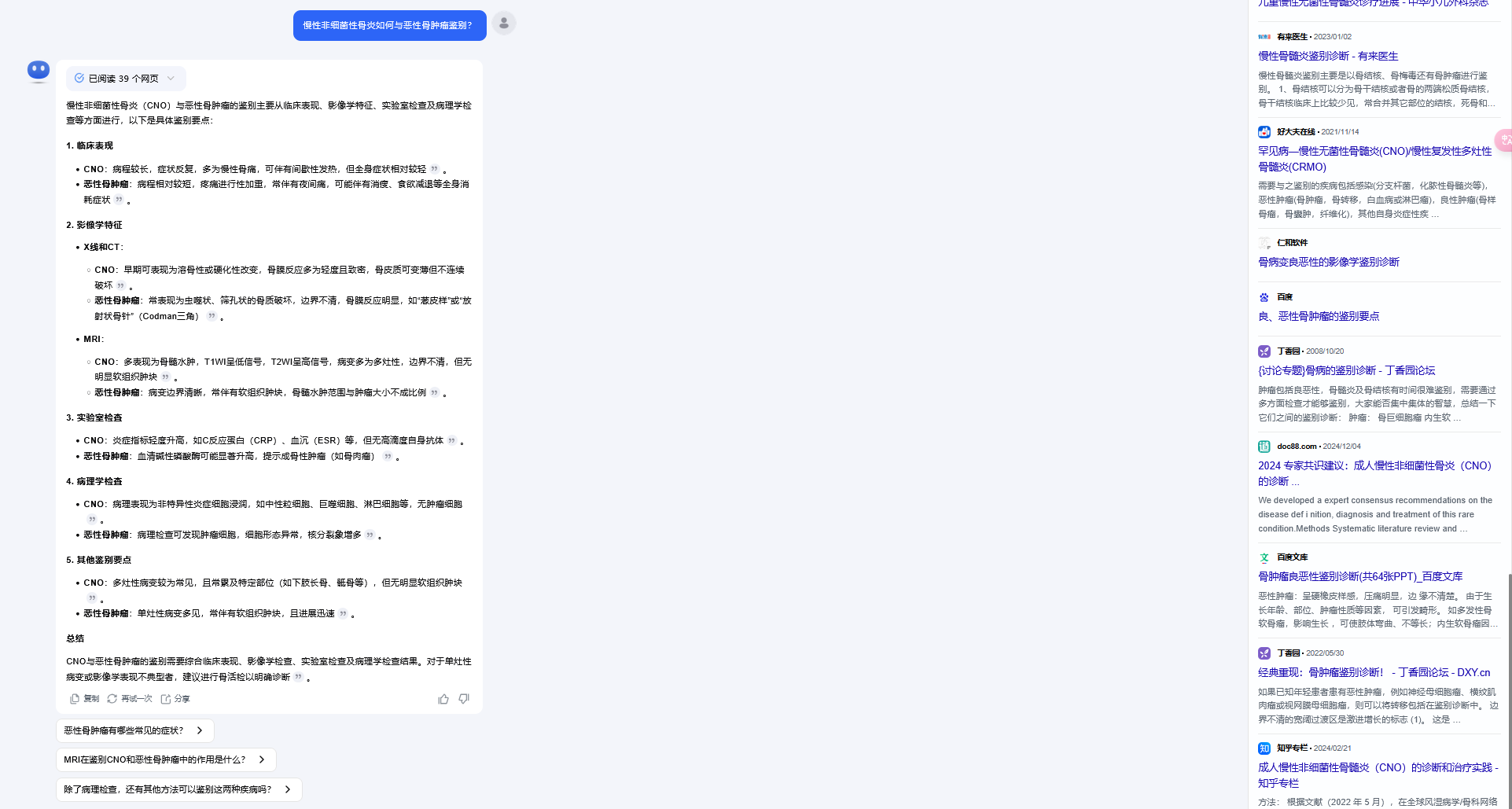


慢性非细菌性骨炎（CNO）与恶性骨肿瘤的鉴别主要从临床表现、影像学特征、实验室检查及病理学检查等方面进行，以下是具体鉴别要点：

### 1. **临床表现**

- **CNO**：病程较长，症状反复，多为慢性骨痛，可伴有间歇性发热，但全身症状相对较轻。

- **恶性骨肿瘤**：病程相对较短，疼痛进行性加重，常伴有夜间痛，可能伴有消瘦、食欲减退等全身消耗症状。

### 2. **影像学特征**

- **X线和CT**：

- **CNO**：早期可表现为溶骨性或硬化性改变，骨膜反应多为轻度且致密，骨皮质可变薄但不连续破坏。

- **恶性骨肿瘤**：常表现为虫噬状、筛孔状的骨质破坏，边界不清，骨膜反应明显，如“葱皮样”或“放射状骨针”（Codman三角）。

- **MRI**：

- **CNO**：多表现为骨髓水肿，T1WI呈低信号，T2WI呈高信号，病变多为多灶性，边界不清，但无明显软组织肿块。

- **恶性骨肿瘤**：病变边界清晰，常伴有软组织肿块，骨髓水肿范围与肿瘤大小不成比例。

### 3. **实验室检查**

- **CNO**：炎症指标轻度升高，如C反应蛋白（CRP）、血沉（ESR）等，但无高滴度自身抗体。

- **恶性骨肿瘤**：血清碱性磷酸酶可能显著升高，提示成骨性肿瘤（如骨肉瘤）。

### 4. **病理学检查**

- **CNO**：病理表现为非特异性炎症细胞浸润，如中性粒细胞、巨噬细胞、淋巴细胞等，无肿瘤细胞。

- **恶性骨肿瘤**：病理检查可发现肿瘤细胞，细胞形态异常，核分裂象增多。

### 5. **其他鉴别要点**

- **CNO**：多灶性病变较为常见，且常累及特定部位（如下肢长骨、骶骨等），但无明显软组织肿块。

- **恶性骨肿瘤**：单灶性病变多见，常伴有软组织肿块，且进展迅速。

### 总结

CNO与恶性骨肿瘤的鉴别需要综合临床表现、影像学检查、实验室检查及病理学检查结果。对于单灶性病变或影像学表现不典型者，建议进行骨活检以明确诊断。

10慢性非细菌性骨炎如何与骨关节炎鉴别？


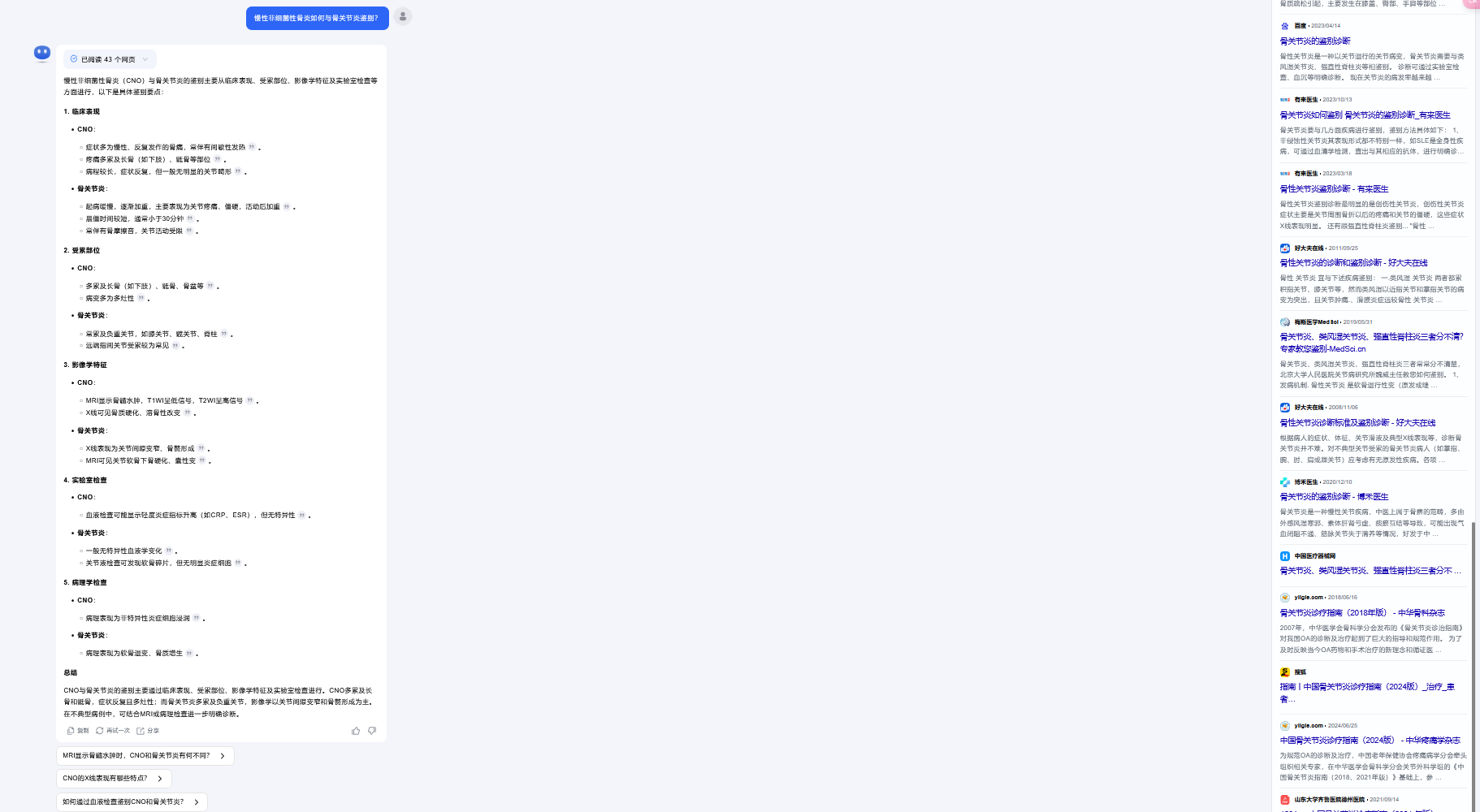


慢性非细菌性骨炎（CNO）与骨关节炎的鉴别主要从临床表现、受累部位、影像学特征及实验室检查等方面进行，以下是具体鉴别要点：

### 1. **临床表现**

- **CNO**：

- 症状多为慢性、反复发作的骨痛，常伴有间歇性发热。

- 疼痛多累及长骨（如下肢）、骶骨等部位。

- 病程较长，症状反复，但一般无明显的关节畸形。

- **骨关节炎**：

- 起病缓慢，逐渐加重，主要表现为关节疼痛、僵硬，活动后加重。

- 晨僵时间较短，通常小于30分钟。

- 常伴有骨摩擦音，关节活动受限。

### 2. **受累部位**

- **CNO**：

- 多累及长骨（如下肢）、骶骨、骨盆等。

- 病变多为多灶性。

- **骨关节炎**：

- 常累及负重关节，如膝关节、髋关节、脊柱。

- 远端指间关节受累较为常见。

### 3. **影像学特征**

- **CNO**：

- MRI显示骨髓水肿，T1WI呈低信号，T2WI呈高信号。

- X线可见骨质硬化、溶骨性改变。

- **骨关节炎**：

- X线表现为关节间隙变窄、骨赘形成。

- MRI可见关节软骨下骨硬化、囊性变。

### 4. **实验室检查**

- **CNO**：

- 血液检查可能显示轻度炎症指标升高（如CRP、ESR），但无特异性。

- **骨关节炎**：

- 一般无特异性血液学变化。

- 关节液检查可发现软骨碎片，但无明显炎症细胞。

### 5. **病理学检查**

- **CNO**：

- 病理表现为非特异性炎症细胞浸润。

- **骨关节炎**：

- 病理表现为软骨退变、骨质增生。

### 总结

CNO与骨关节炎的鉴别主要通过临床表现、受累部位、影像学特征及实验室检查进行。CNO多累及长骨和骶骨，症状反复且多灶性；而骨关节炎多累及负重关节，影像学以关节间隙变窄和骨赘形成为主。在不典型病例中，可结合MRI或病理检查进一步明确诊断。

11慢性非细菌性骨炎如何与纤维发育不良鉴别？


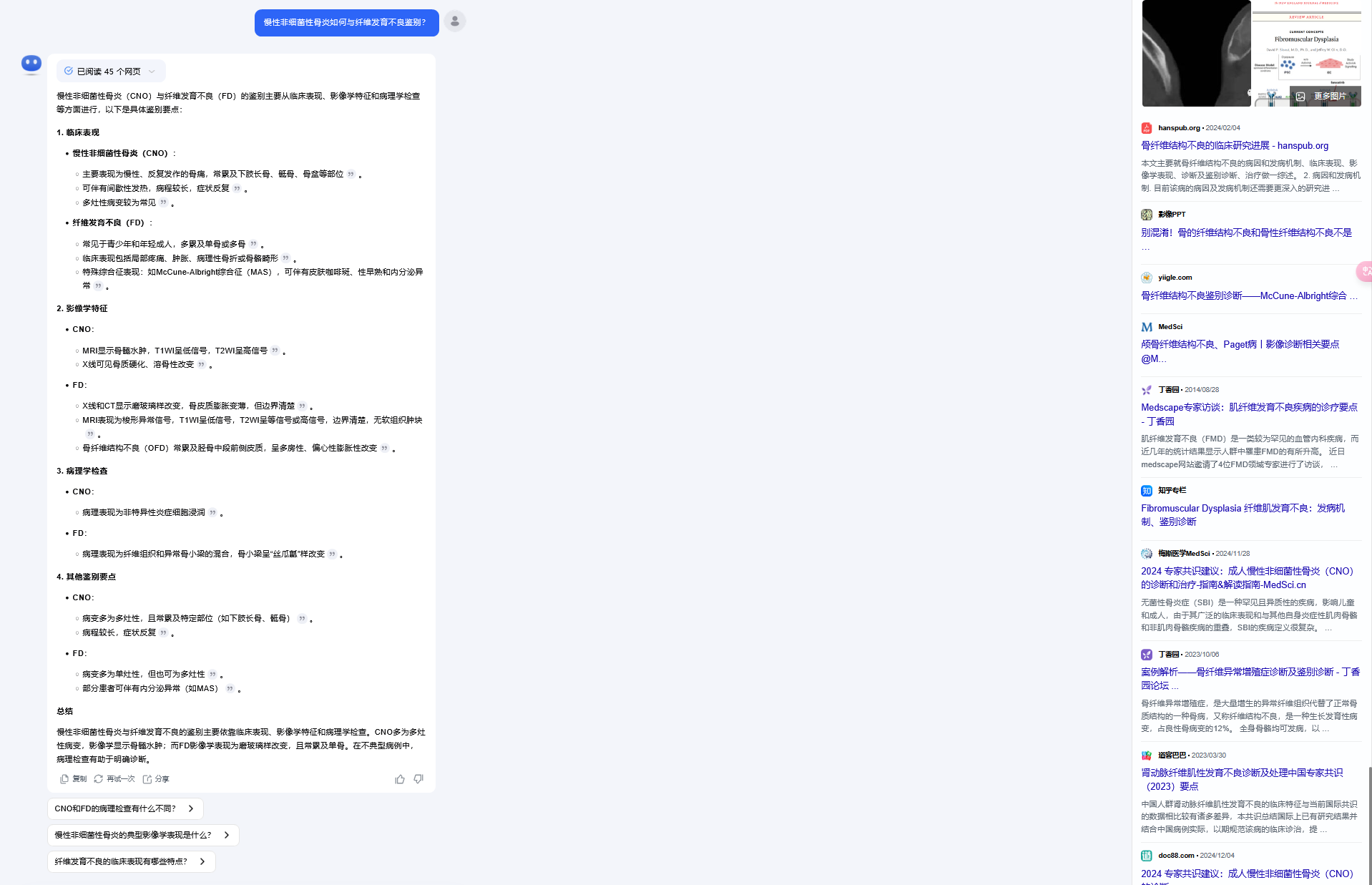


慢性非细菌性骨炎（CNO）与纤维发育不良（FD）的鉴别主要从临床表现、影像学特征和病理学检查等方面进行，以下是具体鉴别要点：

### 1. **临床表现**

- **慢性非细菌性骨炎（CNO）**：

- 主要表现为慢性、反复发作的骨痛，常累及下肢长骨、骶骨、骨盆等部位。

- 可伴有间歇性发热，病程较长，症状反复。

- 多灶性病变较为常见。

- **纤维发育不良（FD）**：

- 常见于青少年和年轻成人，多累及单骨或多骨。

- 临床表现包括局部疼痛、肿胀、病理性骨折或骨骼畸形。

- 特殊综合征表现：如McCune-Albright综合征（MAS），可伴有皮肤咖啡斑、性早熟和内分泌异常。

### 2. **影像学特征**

- **CNO**：

- MRI显示骨髓水肿，T1WI呈低信号，T2WI呈高信号。

- X线可见骨质硬化、溶骨性改变。

- **FD**：

- X线和CT显示磨玻璃样改变，骨皮质膨胀变薄，但边界清楚。

- MRI表现为梭形异常信号，T1WI呈低信号，T2WI呈等信号或高信号，边界清楚，无软组织肿块。

- 骨纤维结构不良（OFD）常累及胫骨中段前侧皮质，呈多房性、偏心性膨胀性改变。

### 3. **病理学检查**

- **CNO**：

- 病理表现为非特异性炎症细胞浸润。

- **FD**：

- 病理表现为纤维组织和异常骨小梁的混合，骨小梁呈“丝瓜瓤”样改变。

### 4. **其他鉴别要点**

- **CNO**：

- 病变多为多灶性，且常累及特定部位（如下肢长骨、骶骨）。

- 病程较长，症状反复。

- **FD**：

- 病变多为单灶性，但也可为多灶性。

- 部分患者可伴有内分泌异常（如MAS）。

### 总结

慢性非细菌性骨炎与纤维发育不良的鉴别主要依靠临床表现、影像学特征和病理学检查。CNO多为多灶性病变，影像学显示骨髓水肿；而FD影像学表现为磨玻璃样改变，且常累及单骨。在不典型病例中，病理检查有助于明确诊断。

12在治慢性非细菌性骨炎过程中应该选择短疗程的糖皮质激素治疗还是长期的糖皮质激素治疗？


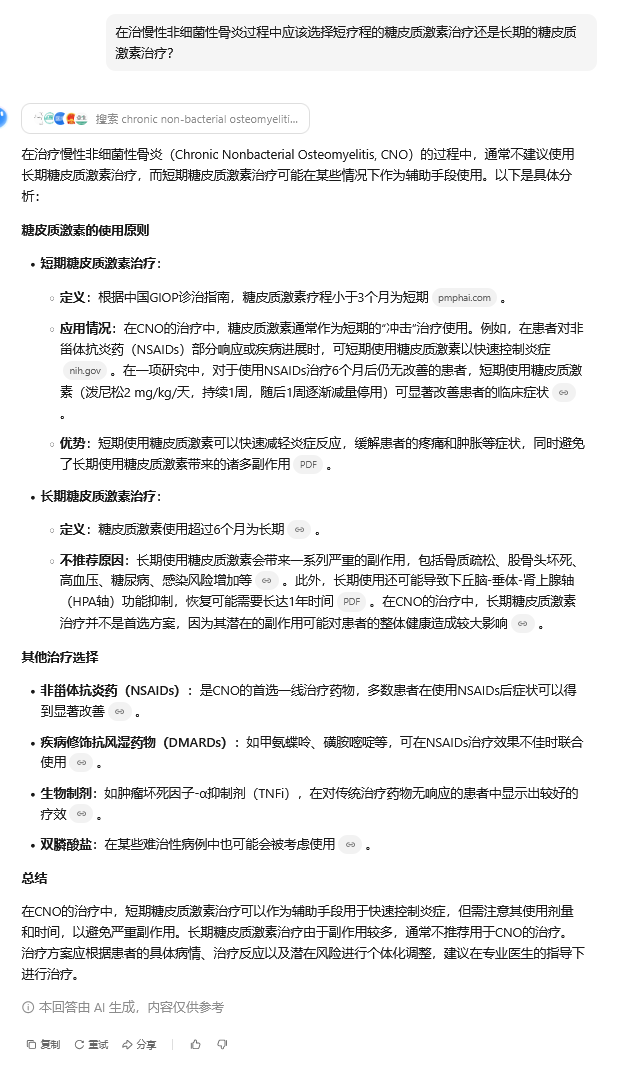
在治疗慢性非细菌性骨炎（Chronic Nonbacterial Osteomyelitis, CNO）的过程中，通常不建议使用长期糖皮质激素治疗，而短期糖皮质激素治疗可能在某些情况下作为辅助手段使用。以下是具体分析：

### 糖皮质激素的使用原则

- **短期糖皮质激素治疗**：

- **定义**：根据中国GIOP诊治指南，糖皮质激素疗程小于3个月为短期。

- **应用情况**：在CNO的治疗中，糖皮质激素通常作为短期的“冲击”治疗使用。例如，在患者对非甾体抗炎药（NSAIDs）部分响应或疾病进展时，可短期使用糖皮质激素以快速控制炎症。在一项研究中，对于使用NSAIDs治疗6个月后仍无改善的患者，短期使用糖皮质激素（泼尼松2 mg/kg/天，持续1周，随后1周逐渐减量停用）可显著改善患者的临床症状。

- **优势**：短期使用糖皮质激素可以快速减轻炎症反应，缓解患者的疼痛和肿胀等症状，同时避免了长期使用糖皮质激素带来的诸多副作用。

- **长期糖皮质激素治疗**：

- **定义**：糖皮质激素使用超过6个月为长期。

- **不推荐原因**：长期使用糖皮质激素会带来一系列严重的副作用，包括骨质疏松、股骨头坏死、高血压、糖尿病、感染风险增加等。此外，长期使用还可能导致下丘脑-垂体-肾上腺轴（HPA轴）功能抑制，恢复可能需要长达1年时间。在CNO的治疗中，长期糖皮质激素治疗并不是首选方案，因为其潜在的副作用可能对患者的整体健康造成较大影响。

### 其他治疗选择

- **非甾体抗炎药（NSAIDs）**：是CNO的首选一线治疗药物，多数患者在使用NSAIDs后症状可以得到显著改善。

- **疾病修饰抗风湿药物（DMARDs）**：如甲氨蝶呤、磺胺嘧啶等，可在NSAIDs治疗效果不佳时联合使用。

- **生物制剂**：如肿瘤坏死因子-α抑制剂（TNFi），在对传统治疗药物无响应的患者中显示出较好的疗效。

- **双膦酸盐**：在某些难治性病例中也可能会被考虑使用。

### 总结

在CNO的治疗中，短期糖皮质激素治疗可以作为辅助手段用于快速控制炎症，但需注意其使用剂量和时间，以避免严重副作用。长期糖皮质激素治疗由于副作用较多，通常不推荐用于CNO的治疗。治疗方案应根据患者的具体病情、治疗反应以及潜在风险进行个体化调整，建议在专业医生的指导下进行治疗。

13成人慢性非细菌性骨炎一线治疗方案是什么？


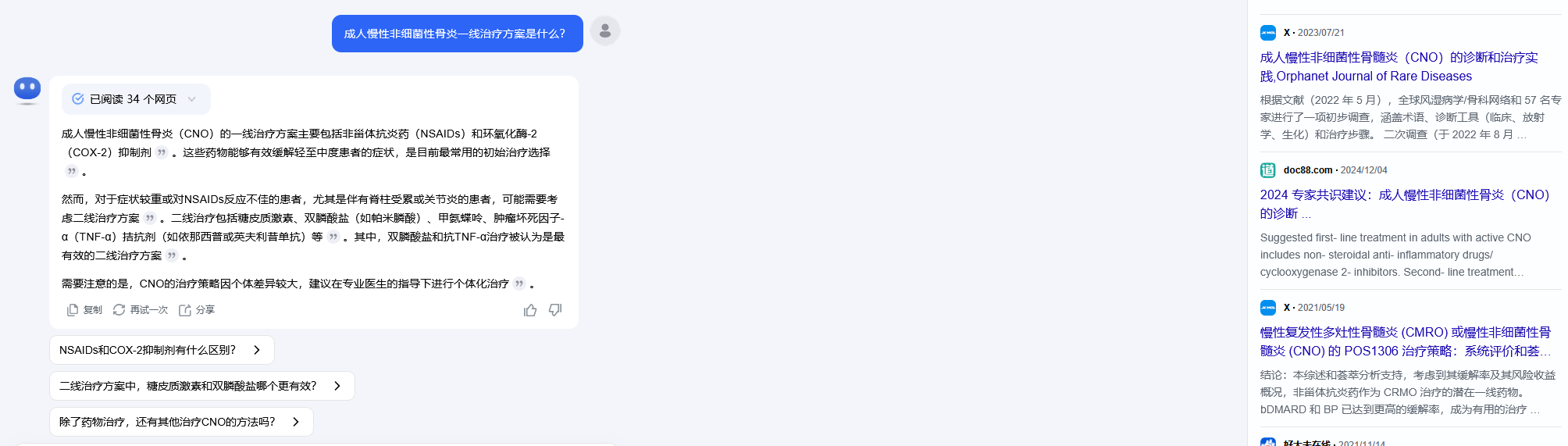


成人慢性非细菌性骨炎（CNO）的一线治疗方案主要包括非甾体抗炎药（NSAIDs）和环氧化酶-2（COX-2）抑制剂。这些药物能够有效缓解轻至中度患者的症状，是目前最常用的初始治疗选择。

然而，对于症状较重或对NSAIDs反应不佳的患者，尤其是伴有脊柱受累或关节炎的患者，可能需要考虑二线治疗方案。二线治疗包括糖皮质激素、双膦酸盐（如帕米膦酸）、甲氨蝶呤、肿瘤坏死因子-α（TNF-α）拮抗剂（如依那西普或英夫利昔单抗）等。其中，双膦酸盐和抗TNF-α治疗被认为是最有效的二线治疗方案。

需要注意的是，CNO的治疗策略因个体差异较大，建议在专业医生的指导下进行个体化治疗。

14无临床症状，也没有放射学疾病活动的慢性非细菌性骨炎的病人需要治疗吗？


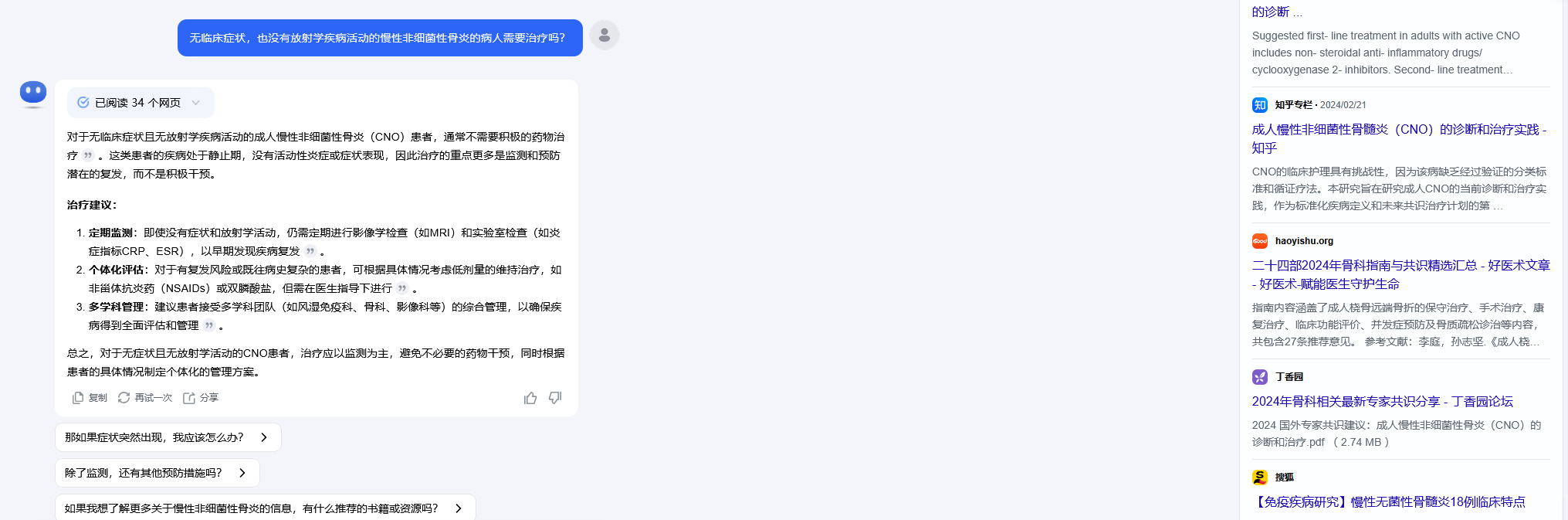


对于无临床症状且无放射学疾病活动的成人慢性非细菌性骨炎（CNO）患者，通常不需要积极的药物治疗。这类患者的疾病处于静止期，没有活动性炎症或症状表现，因此治疗的重点更多是监测和预防潜在的复发，而不是积极干预。

### 治疗建议：

1. **定期监测**：即使没有症状和放射学活动，仍需定期进行影像学检查（如MRI）和实验室检查（如炎症指标CRP、ESR），以早期发现疾病复发。

2. **个体化评估**：对于有复发风险或既往病史复杂的患者，可根据具体情况考虑低剂量的维持治疗，如非甾体抗炎药（NSAIDs）或双膦酸盐，但需在医生指导下进行。

3. **多学科管理**：建议患者接受多学科团队（如风湿免疫科、骨科、影像科等）的综合管理，以确保疾病得到全面评估和管理。

总之，对于无症状且无放射学活动的CNO患者，治疗应以监测为主，避免不必要的药物干预，同时根据患者的具体情况制定个体化的管理方案。

15有临床症状没有放射学疾病活动的慢性非细菌性骨炎的病人如何处理？


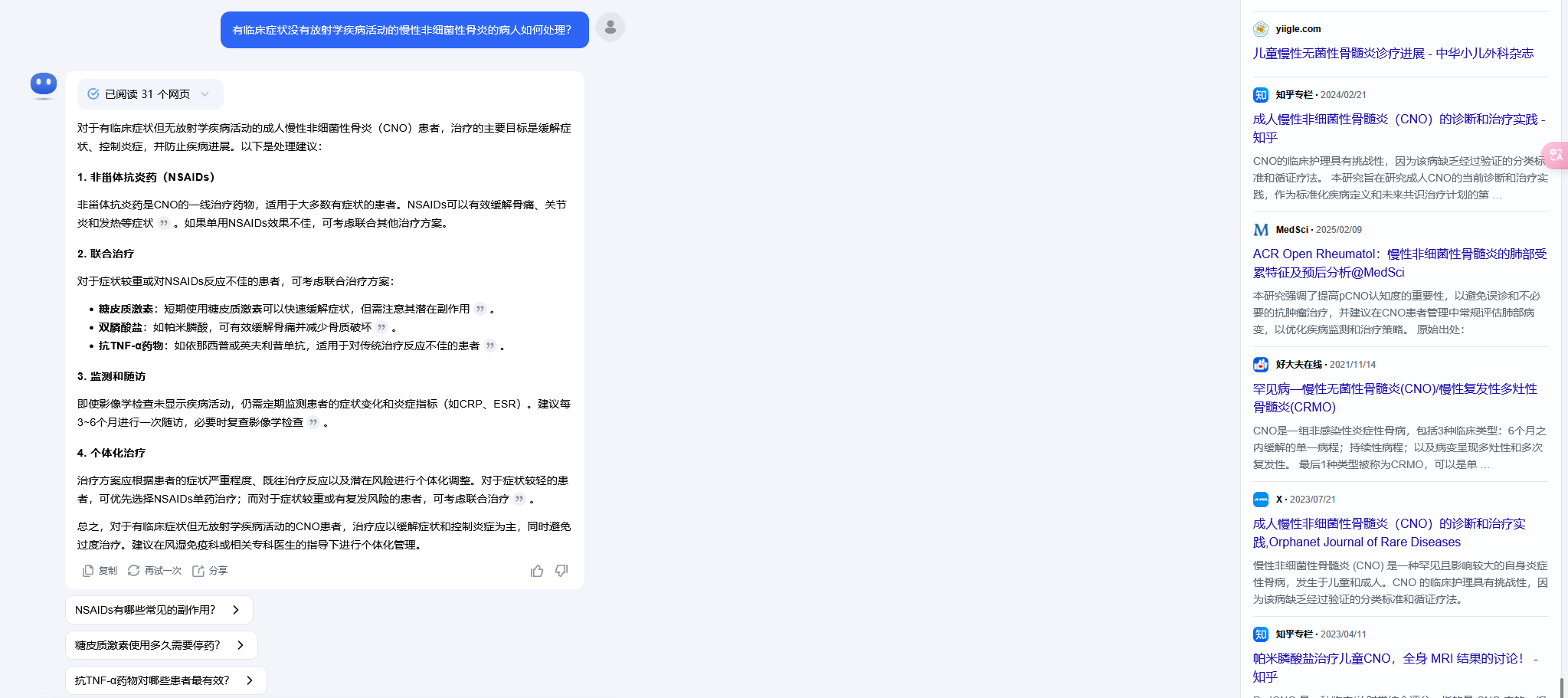


对于有临床症状但无放射学疾病活动的成人慢性非细菌性骨炎（CNO）患者，治疗的主要目标是缓解症状、控制炎症，并防止疾病进展。以下是处理建议：

### 1. **非甾体抗炎药（NSAIDs）**

非甾体抗炎药是CNO的一线治疗药物，适用于大多数有症状的患者。NSAIDs可以有效缓解骨痛、关节炎和发热等症状。如果单用NSAIDs效果不佳，可考虑联合其他治疗方案。

### 2. **联合治疗**

对于症状较重或对NSAIDs反应不佳的患者，可考虑联合治疗方案：

- **糖皮质激素**：短期使用糖皮质激素可以快速缓解症状，但需注意其潜在副作用。

- **双膦酸盐**：如帕米膦酸，可有效缓解骨痛并减少骨质破坏。

- **抗TNF-α药物**：如依那西普或英夫利昔单抗，适用于对传统治疗反应不佳的患者。

### 3. **监测和随访**

即使影像学检查未显示疾病活动，仍需定期监测患者的症状变化和炎症指标（如CRP、ESR）。建议每3~6个月进行一次随访，必要时复查影像学检查。

### 4. **个体化治疗**

治疗方案应根据患者的症状严重程度、既往治疗反应以及潜在风险进行个体化调整。对于症状较轻的患者，可优先选择NSAIDs单药治疗；而对于症状较重或有复发风险的患者，可考虑联合治疗。

总之，对于有临床症状但无放射学疾病活动的CNO患者，治疗应以缓解症状和控制炎症为主，同时避免过度治疗。建议在风湿免疫科或相关专科医生的指导下进行个体化管理。

16有椎体塌陷风险的脊柱骨病变的慢性非细菌性骨炎患者和出现明显骨骼累积损伤的慢性非细菌性骨炎患者如何治疗？


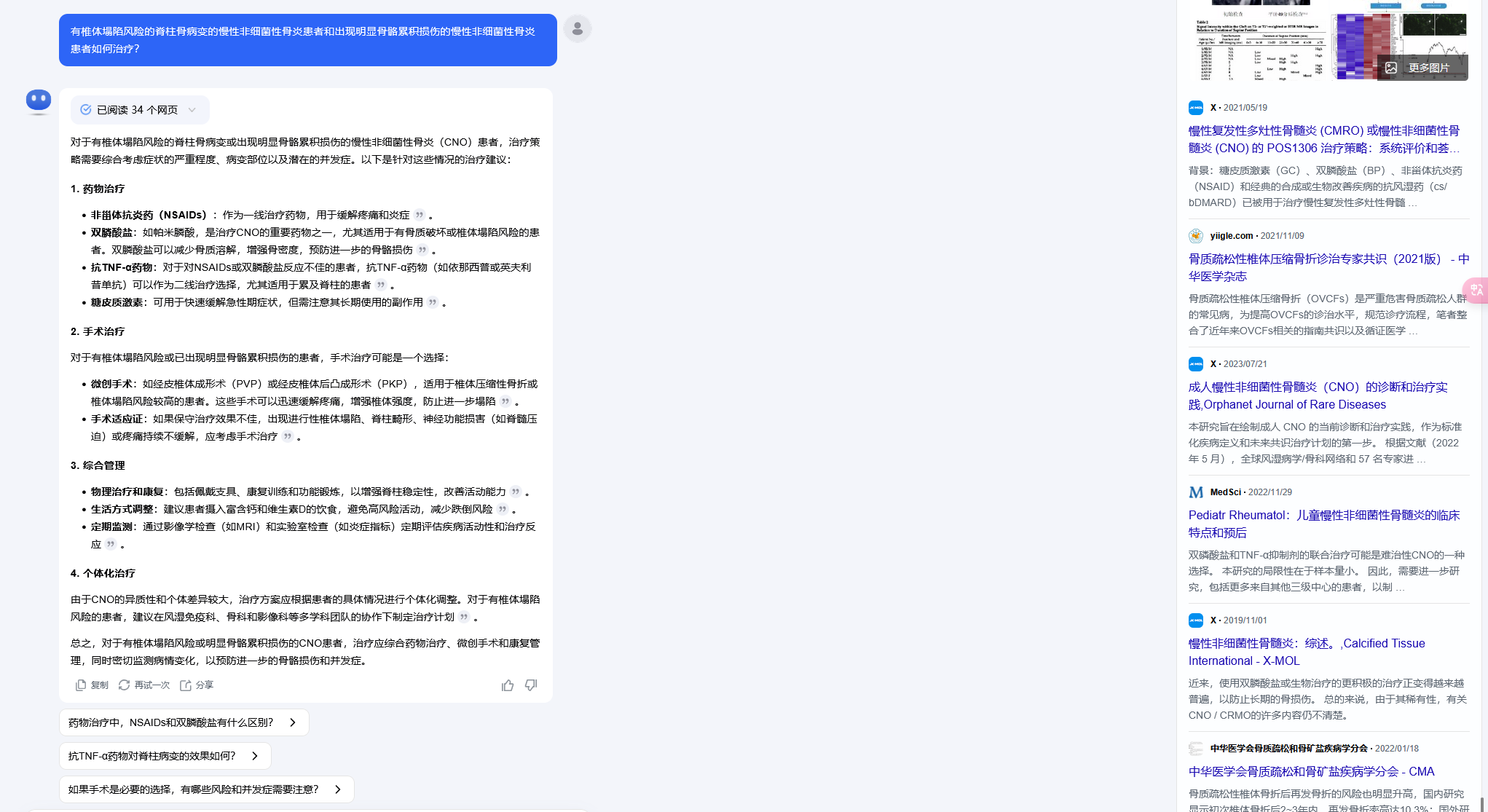


对于有椎体塌陷风险的脊柱骨病变或出现明显骨骼累积损伤的慢性非细菌性骨炎（CNO）患者，治疗策略需要综合考虑症状的严重程度、病变部位以及潜在的并发症。以下是针对这些情况的治疗建议：

### 1. **药物治疗**

- **非甾体抗炎药（NSAIDs）**：作为一线治疗药物，用于缓解疼痛和炎症。

- **双膦酸盐**：如帕米膦酸，是治疗CNO的重要药物之一，尤其适用于有骨质破坏或椎体塌陷风险的患者。双膦酸盐可以减少骨质溶解，增强骨密度，预防进一步的骨骼损伤。

- **抗TNF-α药物**：对于对NSAIDs或双膦酸盐反应不佳的患者，抗TNF-α药物（如依那西普或英夫利昔单抗）可以作为二线治疗选择，尤其适用于累及脊柱的患者。

- **糖皮质激素**：可用于快速缓解急性期症状，但需注意其长期使用的副作用。

### 2. **手术治疗**

对于有椎体塌陷风险或已出现明显骨骼累积损伤的患者，手术治疗可能是一个选择：

- **微创手术**：如经皮椎体成形术（PVP）或经皮椎体后凸成形术（PKP），适用于椎体压缩性骨折或椎体塌陷风险较高的患者。这些手术可以迅速缓解疼痛，增强椎体强度，防止进一步塌陷。

- **手术适应证**：如果保守治疗效果不佳，出现进行性椎体塌陷、脊柱畸形、神经功能损害（如脊髓压迫）或疼痛持续不缓解，应考虑手术治疗。

### 3. **综合管理**

- **物理治疗和康复**：包括佩戴支具、康复训练和功能锻炼，以增强脊柱稳定性，改善活动能力。

- **生活方式调整**：建议患者摄入富含钙和维生素D的饮食，避免高风险活动，减少跌倒风险。

- **定期监测**：通过影像学检查（如MRI）和实验室检查（如炎症指标）定期评估疾病活动性和治疗反应。

### 4. **个体化治疗**

由于CNO的异质性和个体差异较大，治疗方案应根据患者的具体情况进行个体化调整。对于有椎体塌陷风险的患者，建议在风湿免疫科、骨科和影像科等多学科团队的协作下制定治疗计划。

总之，对于有椎体塌陷风险或明显骨骼累积损伤的CNO患者，治疗应综合药物治疗、微创手术和康复管理，同时密切监测病情变化，以预防进一步的骨骼损伤和并发症。
