# Supplementary material for: Evaluation of the accuracy and repeatability of Deepseek V3, Doubao, and Kimi1.5 in answering knowledge-related queries about chronic non-bacterial osteitis
Source: Front Artif Intell. 2025 Sep 29;8:1629149. doi: 10.3389/frai.2025.1629149 (PMC12515971; doi:10.3389/frai.2025.1629149)
Supplement: Supplementary file 2 [file Data_Sheet_2.zip › Supplementary Material/Table_2.DOCX]

**Table S2** the scores given by the two reviewers and the more experienced expert in the second rounds of questioning.

| Number | Question | Reviewer1 Grades (Deeseek,Doubao,Kimi) | Reviewer2 Grades (Deeseek,Doubao,Kimi) |
| --- | --- | --- | --- |
| 1 | What is the definition of adult chronic nonbacterial osteomyelitis? | 3,4,4 | 4,4,4 |
| 2 | What are the typical imaging features of adult chronic nonbacterial osteomyelitis? | 4,4,4 | 4,4,4 |
| 3 | What are the most common manifestations of adult chronic nonbacterial osteomyelitis? | 4,4,4 | 4,4,4 |
| 4 | What are the most commonly affected sites in adult chronic nonbacterial osteomyelitis? | 2,2,2 | 2,3,2 |
| 5 | Are there any specific inflammatory markers for adult chronic nonbacterial osteomyelitis? | 4,4,4 | 4,4,4 |
| 6 | What is the preferred imaging examination for chronic nonbacterial osteomyelitis? | 4,4,4 | 4,4,4 |
| 7 | Is whole-body imaging examination a prerequisite for diagnosing adult chronic nonbacterial osteomyelitis? | 4,4,4 | 4,4,4 |
| 8 | Is routine bone biopsy necessary for the diagnosis of adult chronic nonbacterial osteomyelitis? | 4,4,4 | 4,4,4 |
| 9 | How to differentiate chronic nonbacterial osteomyelitis from malignant bone tumors? | 4,4,4 | 3,4,4 |
| 10 | How to differentiate chronic nonbacterial osteomyelitis from osteoarthritis? | 4,4,4 | 3,4,4 |
| 11 | How to differentiate chronic nonbacterial osteomyelitis from fibrous dysplasia? | 4,4,4 | 4,3,3 |
| 12 | During the treatment of chronic nonbacterial osteomyelitis, should a short-course or long-term glucocorticoid therapy be chosen? | 4,4,4 | 3,3,4 |
| 13 | What is the first-line treatment option for adult chronic nonbacterial osteomyelitis? | 4,4,4 | 3,4,3 |
| 14 | Do patients with chronic nonbacterial osteomyelitis who have no clinical symptoms and no radiological evidence of disease activity require treatment? | 4,4,4 | 4,4,4 |
| 15 | How should patients with chronic nonbacterial osteomyelitis who have clinical symptoms but no radiological evidence of disease activity be managed? | 4,4,4 | 4,4,4 |
| 16 | How should patients with chronic nonbacterial osteomyelitis involving spinal bone lesions at risk of vertebral collapse and those with significant cumulative skeletal damage be treated? | 3,4,4 | 3,4,4 |
